# Genome-wide associated variants of subclinical atherosclerosis among young people with HIV and gene-environment interactions

**DOI:** 10.1186/s12967-022-03817-6

**Published:** 2022-12-20

**Authors:** Jiayu He, Haijiang Lin, Yingying Ding, Xing Liu, Kelin Xu, Xiaoxiao Chen, Weiwei Shen, Sujuan Zhou, Miaochen Wang, Jingjing Xia, Na He

**Affiliations:** 1 Department of Epidemiology, School of Public Health, and Key Laboratory of Public Health Safety of Ministry of Education, Fudan University, Shanghai, China; 2 Yi-Wu Research Institute, Fudan University, Shanghai, China; 3 Shanghai Institute of Infectious Diseases and Biosecurity, Fudan University, Shanghai, China; 4 Taizhou City Center for Disease Control and Prevention, Taizhou, Zhejiang China; 5 Department of Biostatistics, School of Public Health, Fudan University, Shanghai, China

**Keywords:** Subclinical atherosclerosis, HIV, GWAS, Interaction, Chinese

## Abstract

**Background:**

Genome-wide association studies (GWAS) have identified some variants associated with subclinical atherosclerosis (SCA) in general population but lacking sufficient validation. Besides traditional risk factors, whether and how would genetic variants associate with SCA among people with HIV (PWH) remains to be elucidated.

**Method:**

A large original GWAS and gene-environment interaction analysis of SCA were conducted among Chinese PWH (n = 2850) and age/sex-matched HIV-negative controls (n = 5410). Subgroup analyses by age and functional annotations of variants were also performed.

**Results:**

Different from HIV-negative counterparts, host genome had a greater impact on young PWH rather than the elders: one genome-wide significant variant (rs77741796, *P* = 2.20 × 10^−9^) and eight suggestively significant variants (*P* < 1 × 10^−6^) were identified to be specifically associated with SCA among PWH younger than 45 years. Seven genomic loci and 15 genes were mapped to play a potential role on SCA among young PWH, which were enriched in the biological processes of atrial cardiac muscle cell membrane repolarization and molecular function of protein kinase A subunit binding. Furthermore, genome-wide interaction analyses revealed significant HIV-gene interactions overall as well as gene-environment interactions with alcohol consumption, tobacco use and obesity among PWH. The identified gene-environment interaction on SCA among PWH might be useful for discovering high-risk individuals for the prevention of SCA, particularly among those with tobacco use and alcohol consumption.

**Conclusion:**

The present study provides new clues for the genetic contribution of SCA among young PWH and is the starting point of precision intervention targeting HIV-related atherosclerosis.

**Supplementary Information:**

The online version contains supplementary material available at 10.1186/s12967-022-03817-6.

## Background

Cardiovascular diseases (CVDs) have been identified as a major cause of death among people with HIV (PWH) in the antiretroviral therapy (ART) era [1]. Most forms of CVDs originate from atherosclerosis, a chronic inflammatory disease of blood vessels among elderly population [2]. Of note, there is an increase in incidental atherosclerosis among PWH [3], and HIV infection appears to increase the risk of carotid plaque [4, 5]. Atherosclerosis starts early in life and progresses silently [2] and thus it is necessary to identify atherosclerosis from early subclinical stages. We previously observed a disproportionally higher risk and earlier onset of subclinical atherosclerosis (SCA) among young PWH than HIV-negative counterparts in the Comparative HIV and Aging Research in Taizhou (CHART) cohort [6]. This age-specific association between HIV and SCA is independent of traditional risk factors of CVDs and suggestive of unrecognized unique mechanisms linking HIV infection with SCA [6].

Atherosclerosis is a complex disease with the involvement of multiple factors such as smoking, alcohol use and genetics [7]. It has been reported that genetics plays a vital role in atherosclerosis development [8], accounting for 30–50% of the variance in SCA [9]. Genome-wide association studies (GWAS) and meta-analyses have identified a number of genetic variants that contribute to the risk of SCA in the general population [10–12]. However, whether and how would the genetic variants associate with SCA differentially among PWH remains to be elucidated, especially in Asian people. The contributing effect of HIV infection could involve different sets of genes and biological pathways in SCA development [9]. A GWAS study conducted in 2010 reported two SNPs (rs2229116 and rs7177922) in tight linkage disequilibrium (LD) in the *RYR3* gene associated with SCA in 171 White HIV-infected men, which was also the only GWAS in relation to SCA among PWH [13].

Therefore, in the present study, we conducted a large GWAS of SCA among Chinese PWH and HIV-negative counterparts based on the CHART cohort in an attempt to compare the differences of genetic associations with SCA between these two groups. The possible underlying mechanism of earlier onset of SCA among PWH as previously revealed [6] was explored by age-specific stratified analyses. Furthermore, genome-wide gene-environment interaction analyses of SCA that incorporate HIV infection, alcohol consumption, tobacco use and obesity were also performed.

## Methods

### Study design and participants

Participants were enrolled from the CHART cohort, which is an ongoing prospective cohort study specifically designed to facilitate epidemiological and pathophysiological understandings of aging-related comorbidities among Chinese PWH and comparative HIV-negative individuals [14]. The present cross-sectional study was based on the baseline survey of CHART conducted in 2017–2020. Details about the CHART cohort have been described elsewhere [6].

As of Jan. 2020, an aggregate of 8260 including 2850 PWH and 5410 HIV-negative individuals were enrolled. Eventually included in the analyses were 7904 (95.7%) without missing data on cIMT and after genotyping quality control. Written informed consent was obtained from all study participants. The study was approved by the Institutional Review Board of Fudan University School of Public Health, Shanghai, China.

### Data collection and measurements

#### Questionnaire interview and physical examination

A standardized structured questionnaire was administered face-to-face by trained health staffs to collect information on age, sex, tobacco and regular alcohol use, physical activities and history of non-communicable diseases (NCDs). Regular alcohol use was defined as alcohol use at least 3 times per week. Smoking status was classified as “never”, “previous” or “current”, with current smoking defined as having smoked at least one cigarette in the past 30 days. Physical examinations of waist circumference, hip circumference, height, weight and blood pressures (BP) were carried out. Body mass index (BMI) was calculated and general obesity was defined as BMI ≥ 24 kg/m^2^. The cutoff of waist to hip ratio (WHR) for abdominal obesity was defined as 0.90 for men and 0.85 for women [14].

Hypertension was defined as systolic BP ≥ 140 mmHg or diastolic BP ≥ 90 mmHg, or prior clinical diagnosis of hypertension [15]. Diabetes was defined as HbA1c ≥ 6.5% or a prior clinical diagnosis. Metabolic syndrome (MS) was defined according to standardized protocol [16]. Dyslipidemia was defined as TC ≥ 6.2 mmol/L, LDL ≥ 4.1 mmol/L, HDL < 1.0 mmol/L or TG ≥ 2.3 mmol/L [17]. HIV-related variables were extracted from the national HIV/AIDS Comprehensive Response Information Management System (CRIMS) [18]. Nadir CD4 count was defined as the lowest CD4 count as recorded.

#### SCA measurements and outcome definition

One of the reliable and valid measure of SCA is carotid intima-media thickness (cIMT) [6, 19]. Intima-media thickness (IMT) of the left common carotid artery was measured by trained sonographers using a high-resolution B-mode ultrasound imager (LOGIQ P5 pro, GE, Indianapolis, USA), in accordance with standard procedures. Briefly, an IMT image was obtained on about 10 mm of the longitudinal carotid length which is free of plaque with an identified double-line pattern.

Subclinical carotid atherosclerosis was defined as a cIMT of 780 μm or more, according to our previous published study [6]. The average cIMT values were also categorized into < 780, 780–1000 and > 1000 μm [20]. We assigned two SCA-related phenotypes: one quantitative, using continuous cIMT values and the other categorical, termed “binary-cIMT” with the cutoff of 780 μm.

### Genotyping and quality control

Genomic DNA was extracted from whole peripheral blood samples using a commercial DNA extraction kit (Qiagen) and was quantified using PicoGreen reagent (Invitrogen). We genotyped study samples for 664,165 SNPs on the Infinium™ Chinese Genotyping Array-24 v1.0 BeadChip. We then performed quality control using PLINK 1.9 [21] at sample level and at SNP level according to the following criteria: (1) individual level: call rate < 95%, gender discrepancies checking, heterozygosity rate outliers (> 6 sd.), and unexpected duplicates; (2) SNP level: missing data > 5%, minor allele frequencies (MAF) < 0.05, and deviated from Hardy–Weinberg equilibrium (HWE) (*P* < 10^–6^). Principal component analysis (PCA) was done in PLINK 1.9 for the remaining 372,728 SNPs and the first five principal components (PCs) were extracted and employed in further association analyses.

### Genome-wide association (GWA) analyses

GWA analyses were performed under additive genetic effects assumption. For continuous phenotypes, linear mixed model (LMM) was applied; for dichotomous phenotypes, generalized linear mixed model (GLMM) was used. LMM-based methods are usually preferred over linear regression-based methods largely because they can account for population stratification [22, 23] and relatedness without the need to remove related individuals [24]. LMM was conducted through fastGWA model which is an extremely resource-efficient approach implemented in the GCTA software package [25, 26]. GLMM was conducted through fastGWA-GLMM which is a resource-efficient tool for GLMM based GWAS analysis for binary traits in biobank-scale data such as the UK Biobank [24]. For all analyses, we adjusted for following parameters as covariates: age (continuous variable), sex, regular alcohol use, current smoking status, BMI, and the first five PCs.

We also created quantile–quantile (QQ) plot and Manhattan plot using the R package “CMplot”. A QQ plot was used to evaluate the overall significance of the GWAS, and the deviation of the observed versus the expected distribution of the *P* values was represented by the inflation factor (λ_GC_). We further performed age-specific stratified analyses both in PWH and HIV-negative counterparts. The genome-wide significance threshold was considered at *P* value less than 5 × 10^–8^, and *P* value less than 1 × 10^–6^ indicated a suggestive significance threshold [27, 28]. Plots of representative SNPs were generated using LocusZoom online software [29].

### Genome-wide interaction analyses

In order to test the interaction between environmental factors and genetic variants, we conducted a genome-wide interaction analysis by including a two-way interaction parameter based on the equation: $g\left(Y\right)={\beta }_{0}+{\beta }_{1}\times SNP+{\beta }_{2}\times environmental\, factors+{\beta }_{3}\times \left(SNP\times environmental\, factors\right)+{\beta }_{4}\times {X}_{C}$. Here, *Y* is the vector of the observed cIMT measurement, *β*_*0*_ is a constant, *β*_*1*_ and *β*_*2*_ are the main effects of SNP and environmental factors, respectively, *β*_*4*_ is the main effects of other covariates and *β*_*3*_ is the interaction term to be tested. Environmental factors included HIV infection, alcohol consumption, tobacco use and obesity, respectively.

Age-specific interaction effects of HIV infection and genetic variants on SCA were measured based on the same equation among participants under or above 45 years old (at and above 45 years old).

### Statistical analyses

Comparisons of baseline characteristics, stratified by HIV serostatus, were performed using Student’s t test and analysis of variance (ANOVA) for normally distributed continuous variables, Mann Whitney U test for continuous variables with skewed distributions, and chi-square test for categorical variables. Distribution of cIMT was also analyzed. Logistic regressions were conducted to examine the association of baseline characteristics and SCA.

We also calculated unweighted and weighted genetic risk scores (GRS) of selected risk variants (*P* < 1 × 10^–6^) for SCA. To calculate GRS for the *i*th subject from the selected risk variants, the following formula was used [30]:

$${GRS}_{i}=\sum_{j=1}^{n}{w}_{j}{x}_{ij}$$

Here ${x}_{ij}$ is the number of risk alleles for the *j*th SNP in the *i*th subject (${x}_{ij}=\mathrm{0,1},or 2$) and ${w}_{j}$ is the weight or coefficient of the *j*th SNP. Unweighted genetic risk scores simply counted the number of alleles associated with SCA an individual carried across all potential risk variants, thus giving an equal weight to all risk alleles (${w}_{j}$=1). Weighted genetic risk scores were calculated likewise, with the associated beta estimates as ${w}_{j}$ for each selected SNP allele count. Weighting normally results in higher specificity of the GRS by assigning more weights to variants with stronger effects. A *P* value less than 0.05 served as statistical significance. Data were analyzed with SAS 9.4 software (SAS Institute, Cary, NC, USA).

### Functional annotation, gene mapping and gene set analysis

Functional annotation was performed with Functional Mapping and Annotation (FUMA) [31], an online platform for the functional mapping of genetic variants. We first defined ‘independent significant SNPs’ as those surpassing a predefined threshold *P* value (1 × 10^–6^) and showing moderate to low LD (r^2^ < 0.6). We further defined ‘lead SNPs’ as the subset of independent SNPs (r^2^ < 0.1). In addition, we defined genomic risk loci by merging LD blocks of independent significant SNPs that have close physical position (< 250 kb).

SNPs in genomic risk loci were mapped to genes in FUMA using three strategies: position mapping, expression quantitative trait loci (eQTL) mapping and chromatin interaction mapping. Genes implicated by mapping of GWAS SNPs were further investigated using the GENE2FUNC procedure in FUMA, which provides enrichment of the list of mapped genes in MSigDB gene sets, Kyoto Encyclopedia of Genes and Genomes (KEGG), and Geno Oncology (GO). Details are presented in Additional files 2, 3, 4, 5, 6 and 7.

## Results

### Demographic characteristics and risk factors of SCA

Finally included in the analyses were 2583 PWH and 5321 HIV-negative individuals. Of them, 74.2% were male and 52.4% (4139/7904) aged less than 45 years old. The cIMT phenotype subordinated an approximately normal distribution. Demographic characteristics of participants by HIV serostatus and SCA were summarized in Table 1. PWH had a higher prevalence of SCA than HIV negative counterparts in different categorial groups. PWH who had an older age, general/abdominal obesity, regular alcohol use, current/previous smoking status, hypertension, diabetes or MS, had a higher prevalence of SCA (all *P* < 0.05).

**Table 1:** Sociodemographic characteristics and prevalence of subclinical atherosclerosis among study participants

Characteristics	PWH (n = 2583)			HIV-negative counterparts (n = 5321)			*P* value^c^
	SCA+	SCA−	*P* value^a^	SCA+	SCA−	*P* value^b^	
Overall	940 (36.4)	1643 (63.6)		1516 (28.5)	3805 (71.5)		< 0.001^d^
Age (years)			< 0.001			< 0.001	< 0.001
18–29	71 (14.1)	431 (85.9)		36 (3.6)	971 (96.4)		
30–44	195 (23.1)	651 (76.9)		238 (13.3)	1546 (86.7)		
45–59	332 (43.9)	424 (56.1)		557 (36.2)	980 (63.8)		
60–89	342 (71.4)	137 (28.6)		685 (69.0)	308 (31.0)		
Male	748 (37.1)	1269 (62.9)	0.167	1214 (31.5)	2636 (68.5)	< 0.001	0.762
BMI (kg/m^3^) (n = 7900)			0.017			< 0.001	< 0.001
< 18.5	87 (33.7)	171 (66.3)		41 (14.2)	247 (85.8)		
18.5–24.0	594 (35.0)	1104 (65.0)		573 (23.8)	1830 (76.2)		
> 24	256 (41.1)	367 (58.9)		902 (34.3)	1728 (65.7)		
Smoking status			< 0.001			< 0.001	< 0.001
Never	513 (32.8)	1049 (67.2)		688 (22.9)	2316 (77.1)		
Previous	144 (46.3)	167 (53.7)		272 (52.2)	249 (47.8)		
Current	283 (39.9)	427 (60.1)		556 (31.0)	1240 (69.0)		
Regular alcohol use (n = 7888)			< 0.001			< 0.001	< 0.001
Yes	66 (55.0)	54 (45.0)		316 (44.1)	400 (55.9)		
No	874 (35.5)	1589 (64.5)		1196 (26.2)	3393 (73.9)		
Dyslipidemia			0.095			< 0.001	0.487
Yes	565 (37.8)	931 (62.2)		894 (32.6)	1846 (67.4)		
No	370 (34.6)	701 (65.4)		621 (24.1)	1958 (75.9)		
Total cholesterol (mmol/L, mean ± SD)	4.80 ± 1.10	4.65 ± 1.00	< 0.001	5.19 ± 1.03	5.03 ± 0.98	< 0.001	< 0.001
LDL cholesterol (mmol/L, mean ± SD)	2.52 ± 0.81	2.44 ± 0.71	0.018	3.00 ± 0.86	2.86 ± 0.81	< 0.001	< 0.001
HDL cholesterol (mmol/L, median, IQR)	1.0 (0.9, 1.3)	1.0 (0.9, 1.2)	0.905	1.1 (0.9, 1.3)	1.1 (1.0, 1.4)	< 0.001	< 0.001
Triglycerides (mmol/L, median, IQR)	1.7 (1.2, 2.5)	1.6 (1.1, 2.4)	0.004	2.0 (1.3, 2.9)	1.7 (1.1, 2.6)	< 0.001	< 0.001
Abdominal obesity, measured as WHR (n = 7900)	439 (41.7)	614 (58.3)	< 0.001	974 (38.0)	1589 (62.0)	< 0.001	< 0.001
Hypertension			< 0.001			< 0.001	< 0.001
Yes	307 (53.9)	262 (46.1)		821 (46.8)	934 (53.2)		
No	633 (31.4)	1381 (68.6)		695 (19.5)	2871 (80.5)		
Diabetes			< 0.001			< 0.001	< 0.001
Yes	109 (56.5)	84 (43.5)		307 (53.7)	265 (46.3)		
No	831 (34.8)	1559 (65.2)		1209 (25.5)	3540 (74.5)		
Metabolic syndrome			< 0.001			< 0.001	< 0.001
Yes	337 (45.5)	403 (54.5)		796 (41.0)	1147 (59.0)		
No	600 (32.6)	1239 (67.4)		716 (21.3)	2646 (78.7)		
Baseline CD4 (cells/μl) (n = 2563)			0.024				
≤ 200	177 (38.2)	286 (61.8)					
201–350	255 (40.0)	382 (60.0)					
> 350	500 (34.2)	963 (65.8)					
Nadir CD4 < 200 (cells/μl) (n = 2578)	436 (37.8)	719 (62.2)	0.182				
Years since HIV diagnosis (median, IQR)	1.2 (0.2, 4.4)	1.6 (0.3, 4.5)	0.059				

^a^Compared between SCA+ and SCA− among PWH, assessed by chi-square test, student t test and Mann Whitney U test in appropriate

^b^Compared between SCA+ and SCA− among HIV-negative counterparts, assessed by chi-square test, student t test and Mann Whitney U test in appropriate

^c^Compared between PWH and HIV-negative counterparts among those with SCA, assessed by chi-square test, student t test and Mann Whitney U test in appropriate

^d^Compared the prevalence of SCA between PWH and HIV negative counterparts, assessed by chi-square test

PWH: people with HIV; BMI: body mass index; LDL: low-density lipoprotein; HDL: high-density lipoprotein; SCA: subclinical atherosclerosis

The prevalence of SCA was significantly higher among PWH than HIV-negative counterparts (36.39% vs. 28.49%, *P* < 0.001). Compared with the 18–29 age group, the age groups of 30–44 years (a*OR* = 2.55, 95% CI 2.03–3.20, *P* < 0.001), 45–59 years (a*OR* = 8.22, 95% CI 6.58–10.26, *P* < 0.001) and 60–89 years (a*OR* = 29.83, 95% CI 23.57–37.75, *P* < 0.001) were significantly associated with SCA which was also positively correlated to general obesity (a*OR* = 1.38, 95% CI 1.22–1.55, *P* < 0.001), previous (a*OR* = 1.25, 95% CI 1.04–1.50, *P* = 0.020) or current smoking status (a*OR* = 1.17, 95% CI 1.02–1.35, *P* = 0.024) and HIV infection (a*OR* = 1.77, 95% CI 1.56–2.00, *P* < 0.001), according to multiple logistic regression analysis (Additional file 1: Table S1).

### Genetic variants associated with SCA

**Fig. 1:**
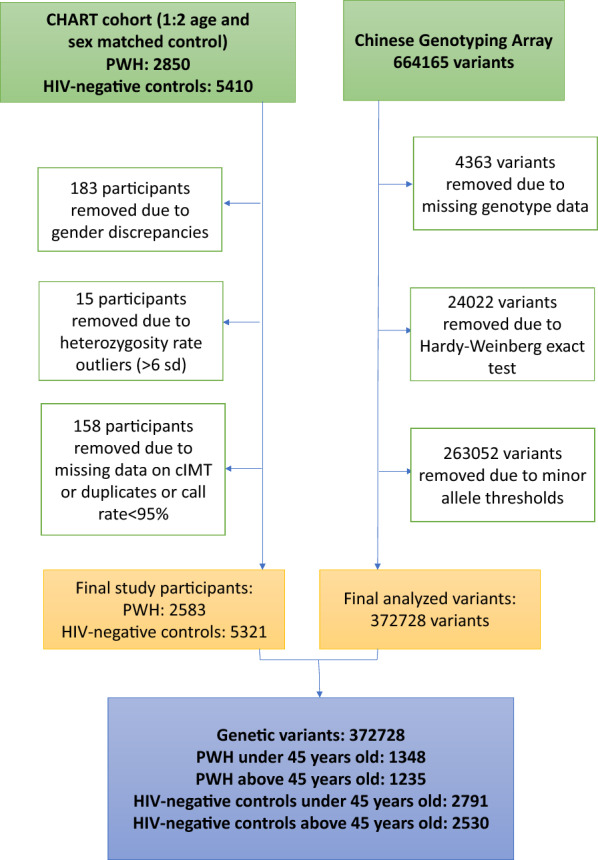
Flow chart of quality control. PWH: people with HIV

A total of 7904 participants and 372,728 SNPs were subject to final association analyses (Fig. 1). The association analyses with SCA were conducted for all participants, PWH and HIV-negative individuals, respectively. Manhattan plots and QQ plots were shown in Additional file 1: Figs. S1–6.

Two variants at *EMC3* (rs3732968, *P* = 1.39 × 10^–7^; rs6786636, *P* = 7.64 × 10^–7^) and one variant at *CRELD1* (rs2302786, *P* = 7.21 × 10^–7^) were potentially associated with cIMT among all participants (Additional file 1: Table S2). Among HIV-negative individuals, three variants at three genes reached the genome-wide association threshold for cIMT (rs2302786, *P* = 3.65 × 10^–8^, *CRELD1*; rs13096737, *P* = 4.73 × 10^–8^, *EMC1*; rs13146599, *P* = 4.80 × 10^–8^, *CCSER1*) and six variants at four genes reached suggestive evidence for cIMT (rs3774207, *P* = 1.33 × 10^–7^, *CRELD1*; rs6786636, *P* = 1.40 × 10^–7^, *EMC3-AS1*; rs3732968, *P* = 2.43 × 10^–7^, *EMC3*; rs8058808, *P* = 2.58 × 10^–7^, *SLC7A5*; rs3755783, *P* = 4.15 × 10^–7^, *EMC3*; rs10856885, *P* = 5.00 × 10^–7^, *CCSER1*) (Additional file 1: Table S2). Among PWH, no variant met the significant level and the most significant variant was rs6772280 (*P* = 3.66 × 10^–6^) located in the gene region of *KCNAB1* on chromosome 3 (Table 2). Another genetic variant, located at the same gene, was also found among the top 4 most significant variants (rs78012168, *P* = 7.98 × 10^–6^) (Table 2).

**Table 2:** Association of SNPs with cIMT among PWH in different groups

SNP	CHR	Position (GRCh37)	Gene	Location	MAF	Minor allele	Major allele	*β* value^a^	Adjusted *P* value^a^
*All PWH*									
rs6772280	3	156,162,567	KCNAB1	Intronic	0.109	G	A	0.07	3.66E−06
rs77741796	12	80,815,650	PTPRQ	Intergenic	0.054	T	C	0.09	5.83E−06
rs77595573	7	32,927,011	KBTBD2	Intronic	0.115	A	C	0.07	6.19E−06
rs78012168	3	156,150,321	KCNAB1	Intronic	0.094	A	G	0.07	7.98E−06
*PWH under 45 years old*									
rs77741796	12	80,815,650	PTPRQ	Intergenic	0.054	T	C	0.14	2.20E−09
rs111815403	11	2,553,341	KCNQ1	Intronic	0.061	A	G	0.11	1.50E−07
rs35812497	5	108,563,812	FER;PJA2	Intergenic	0.089	A	G	0.09	3.27E−07
rs2507941	3	37,536,056	ITGA9	Synonymous	0.064	T	C	0.11	3.85E−07
rs148420952	4	171,482,271	LINC01612	Intergenic	0.051	T	C	0.12	3.94E−07
rs6762348	3	96,683,649	EPHA6	Intronic	0.058	G	A	0.11	4.42E−07
rs62263680	3	96,627,491	EPHA6	Intronic	0.057	C	T	0.12	4.50E−07
rs62262941	3	96,570,987	EPHA6	Intronic	0.057	C	T	0.11	4.80E−07
rs10847321	12	127,782,099	LINC02376	Intergenic	0.056	A	C	0.11	8.91E−07
*PWH above 45 years old*									
rs11948504	5	166,997,147	TENM2	intronic	0.068	G	T	0.16	1.11E−06
rs9851984	3	193,090,806	ATP13A5	intronic	0.382	G	A	− 0.07	6.51E−06
rs36130341	7	343,194	FAM20C	Intergenic	0.273	A	G	0.07	7.70E−06
rs35129955	4	181,960,818	LINC00290	Intergenic	0.054	C	T	0.15	7.90E−06
rs2430722	12	15,122,111	PDE6H	Intergenic	0.255	C	T	− 0.08	1.14E−05
rs6858162	4	40,555,674	RBM47	Intronic	0.050	T	C	0.15	1.35E−05

^a^Assessed by linear mixed model, adjusted for age, sex, smoking status, regular alcohol use, BMI and the first five principal components of PCA

For binary cIMT, no variant reached the potential significance level among all participants, PWH and HIV negative participants (Additional file 1: Table S3).

### Age-specific genetic variants associated with SCA

We further conducted stratified genetic association analyses among PWH and HIV-negative counterparts under or above 45 years old.

**Fig. 2:**
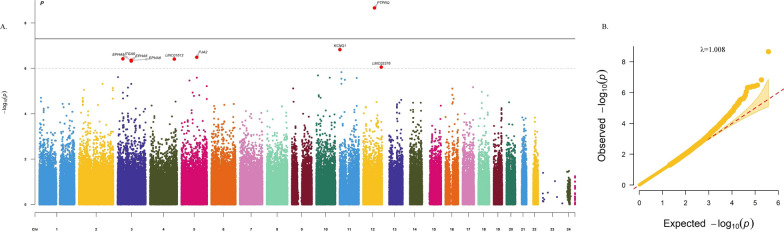
Manhattan plot (**A**) and qqplot (**B**) of SNPs associated with cIMT among PWH under 45 years old. Manhattan plot showing the −log_10_ transformed two-tailed *P* value of each SNP from the GWAS on the y axis and base-pair positions along the chromosomes on the x axis. The red dots indicate SNPs surpassing Bonferroni-corrected suggestive significance threshold (*P* < 1 × 10^–6^). (n = 1348 individuals)

**Fig. 3:**
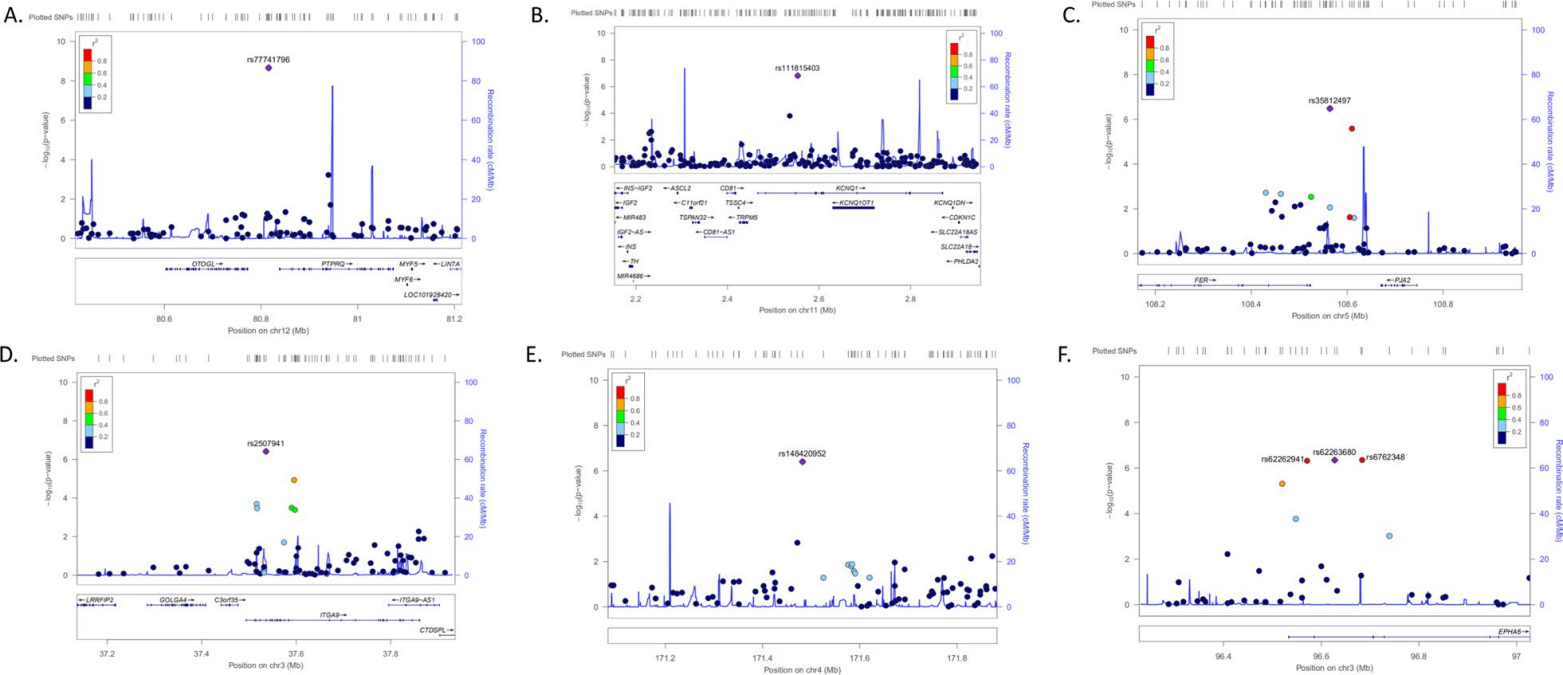
Regional plots of the association results for the significant susceptibility loci associated with cIMT among PWH under 45 years old. **A** rs77741796; **B** rs111815403; **C** rs35812497; **D** rs2507941; **E** rs148420952; **F** rs62263680, rs62262941, rs6762348. SNPs shown in red, orange, green, light blue and blue have r^2^ ≥ 0.8, r^2^ ≥ 0.6, r^2^ ≥ 0.4, r^2^ ≥ 0.2 and r^2^ < 0.2 with the tag SNP, respectively. Purple diamonds represent associations of tag SNPs identified in the GWAS stage

Manhattan plot and QQ plot in Fig. 2 showed the genetic association with cIMT among PWH under 45 years old. As shown in Table 2, among PWH under 45 years old, one variant at chromosome 12 (rs77741796, *P* = 2.20 × 10^–9^) reached genome-wide significance level with cIMT (Fig. 3A). Eight suggestively significant variants to cIMT were also identified, including variants located at *KCNQ1* of chromosome 11 (rs111815403, *P* = 1.50 × 10^–7^) (Fig. 3B), *FER*/*PJA2* of chromosome 5 (rs35812497, *P* = 3.27 × 10^–7^) (Fig. 3C), *ITGA9* genes of chromosome 3 (rs2507941, *P* = 3.85 × 10^–7^) (Fig. 3D), and two variants located at chromosome 4 and 12, respectively (rs148420952, *P* = 3.94 × 10^–7^; rs10847321, *P* = 8.91 × 10^–7^) (Fig. 3E). Three of the eight variants were located at the *EPHA6* of chromosome 3 (rs6762348, *P* = 4.42 × 10^–7^; rs62263680, *P* = 4.50 × 10^–7^; rs62262941, *P* = 4.80 × 10^–7^) (Fig. 3F). However, there was no significant or potentially significant variant associated with cIMT among PWH above 45 years old (Table 2).

Among HIV-negative individuals under 45 years old, three variants were significantly associated with cIMT, two located at *GRIP2* (rs34527568, *P* = 2.81 × 10^–9^; rs9863287, *P* = 7.04 × 10^–9^) and another at *RBFOX1* (rs9932976, *P* = 4.41 × 10^–8^). Five variants were potentially related to cIMT (rs9938274, *P* = 7.13 × 10^–8^; rs10255973, *P* = 6.57 × 10^–7^; rs9385488, *P* = 9.71 × 10^–7^; rs118148069, *P* = 4.50 × 10^–7^; rs4485261, *P* = 4.77 × 10^–7^). Among HIV negative individuals above 45 years old, three variants (rs62487045, *P* = 4.53 × 10^–9^; rs8058808, *P* = 2.97 × 10^–8^; rs4661575, *P* = 3.63 × 10^–8^) reached genome-wide significance level with cIMT and six variants reached potential significance level (rs16891400, *P* = 7.43 × 10^–8^; rs587741, *P* = 1.83 × 10^–7^; rs28412203, *P* = 1.88 × 10^–7^; rs76462900, *P* = 3.65 × 10^–7^; rs1788783, *P* = 6.08 × 10^–7^; rs11625012, *P* = 8.84 × 10^–7^) (Additional file 1: Table S2).

For binary cIMT, no variant reached the potential significance level among PWH and HIV negative participants under or above 45 years old (Additional file 1: Table S3).

### HIV-Gene interaction on SCA

Genome-wide interaction analyses were conducted among all participants and the most significant interaction effect was observed for rs78012168 at *KCNAB1* (*β* = 0.09, *P*_*interact*_ = 7.49 × 10^–6^)) (Table 3). Negative interactions with HIV infection were observed for the potential risk variants of cIMT to HIV-negative individuals (for three variants reaching the genome-wide association threshold: rs2302786, *β* = − 0.05, *P*_*interact*_ = 0.001; rs13096737, *β* = − 0.07, *P*_*interact*_ = 0.001; rs13146599, *β* = − 0.04, *P*_*interact*_ = 0.022; and for six variants reaching suggestive association threshold: rs3774207, *β* = − 0.04, *P*_*interact*_ = 0.002; rs6786636, *β* = − 0.04, *P*_*interact*_ = 0.006; rs3732968, *β* = − 0.04, *P*_*interact*_ = 0.017; rs8058808, *β* = − 0.05, *P*_*interact*_ = 0.035; rs3755783, *β* = − 0.04, *P*_*interact*_ = 0.006; rs10856885, *β* = − 0.03, *P*_*interact*_ = 0.043) (Table 3). Both HIV infection and genetic variants remained significantly associated with cIMT in interaction analyses (all *P* < 0.001).

**Table 3:** Association of SNP × HIV infection with cIMT among different groups

SNP	CHR	Position (GRCh37)	MAF	Gene	Location	Minor allele	SNP × HIV		SNP		HIV	
							*β* value^a^	Adjusted *P* value^a^	*β* value^a^	Adjusted *P* value^a^	*β* value^a^	Adjusted *P* value^a^
*Among all participants*												
rs78012168	3	156,150,321	0.098	KCNAB1	Intronic	A	0.09	7.49E−06	− 0.1	1.69E−04	0.04	3.48E−05
rs1194716	10	54,196,773	0.199	DKK1	Intergenic	T	0.06	1.17E−05	− 0.08	4.74E−05	0.03	4.10E−03
rs4814734	20	1,889,477	0.333	SIRPA	Intronic	T	0.05	1.33E−05	− 0.06	1.98E−04	0.02	8.50E−02
rs13096737	3	10,016,911	0.073	EMC3	Intronic	C	− 0.07	9.95E−04	0.14	5.38E−06	0.07	5.58E−13
rs2302786	3	9,979,660	0.199	CRELD1	Intronic	G	− 0.05	1.26E−03	0.09	8.71E−06	0.07	9.41E−13
rs3774207	3	9,985,656	0.200	CRELD1	Synonymous	T	− 0.04	2.20E−03	0.08	2.45E−05	0.07	1.91E−12
rs3755783	3	10,029,289	0.167	EMC3	Intronic	G	− 0.04	5.90E−03	0.09	6.36E−05	0.07	3.60E−12
rs6786636	3	10,045,703	0.170	EMC3	3′-UTR	C	− 0.04	6.44E−03	0.09	4.89E−05	0.07	2.67E−12
rs3732968	3	10,013,273	0.149	EMC3	Intronic	G	− 0.04	1.72E−02	0.09	2.35E−04	0.06	1.63E−11
rs13146599	4	92,348,857	0.114	CCSER1	Intronic	A	− 0.04	2.21E−02	0.09	2.35E−04	0.06	1.63E−11
rs8058808	16	87,839,943	0.052	SLC7A5	Intergenic	A	− 0.05	3.45E−02	0.12	4.17E−04	0.06	1.94E−11
rs10856885	4	92,309,543	0.130	CCSER1	Intronic	C	− 0.03	4.29E−02	0.08	1.06E−03	0.06	5.64E−11
*Among participants under 45 years old*												
rs9932976	16	6,505,665	0.119	RBFOX1	Intronic	C	− 0.09	3.47E−07	0.14	1.20E−08	0.1	1.38E−27
rs9938274	16	6,505,208	0.118	RBFOX1	Intronic	T	− 0.09	4.00E−07	0.14	1.67E−08	0.1	9.96E−28
rs1345439	16	48,751,012	0.133	CBLN1	Intergenic	A	0.08	6.33E−07	− 0.1	1.94E−05	0.06	6.29E−10
rs2507941	3	37,536,056	0.064	ITGA9	Synonymous	T	0.12	8.42E−07	− 0.14	7.37E−05	0.07	7.07E−15
rs6762348	3	96,683,649	0.058	EPHA6	Intronic	G	0.12	1.93E−06	− 0.12	2.80E−04	0.07	3.48E−15
rs62263680	3	96,627,491	0.057	EPHA6	Intronic	C	0.12	2.45E−06	− 0.12	3.71E−04	0.07	2.98E−15
rs62262941	3	96,570,987	0.057	EPHA6	Intronic	C	0.12	3.13E−06	− 0.12	4.77E−04	0.07	2.15E−15
rs77741796	12	80,815,650	0.054	PTPRQ	Intergenic	T	0.11	8.89E−06	− 0.09	1.18E−02	0.07	2.67E−16
rs148420952	4	171,482,271	0.051	LINC01612	Intergenic	T	0.11	4.05E−05	− 0.09	1.21E−02	0.07	1.53E−16
rs35812497	5	108,563,812	0.089	FER, PJA2	Intergenic	A	0.08	1.14E−04	− 0.07	1.53E−02	0.07	1.04E−13
rs10847321	12	127,782,099	0.056	LINC02376	Intergenic	A	0.09	1.69E−04	− 0.07	3.06E−02	0.07	4.96E−16
rs111815403	11	2,553,341	0.061	KCNQ1	Intronic	A	0.07	2.11E−03	− 0.04	2.65E−01	0.07	1.65E−16
*Among participants above 45 years old*												
rs11948504	5	166,997,147	0.068	TENM2	Intronic	G	0.19	7.16E−06	− 0.21	2.72E−04	− 0.01	9.34E−01
rs2430722	12	15,122,111	0.255	PDE6H	Intergenic	C	− 0.07	3.53E−03	0.08	1.37E−02	0.06	4.32E−03
rs35129955	4	181,960,818	0.054	LINC00290	Intergenic	C	0.12	6.55E−03	− 0.11	6.05E−02	0.01	6.22E−01
rs36130341	7	343,194	0.273	FAM20C	Intergenic	A	0.05	1.60E−02	− 0.06	6.79E−02	− 0.01	6.80E−01
rs6858162	4	40,555,674	0.050	RBM47	Intronic	T	0.11	1.76E−02	− 0.09	1.72E−01	0.01	5.00E−01
rs9851984	3	193,090,806	0.382	ATP13A5	Intronic	G	− 0.04	5.62E−02	0.03	3.18E−01	0.05	2.23E−02

^a^Assessed by generalized linear model, adjusted for age, sex, smoking status, regular alcohol use, BMI

### Age-specific HIV-gene interactions on SCA

We then conducted genome-wide interaction analyses among participants under or above 45 years old. Among participants under 45 years old, four variants had a suggestive significant interaction effect with HIV infection (rs9932976, *β* = − 0.09, *P*_*interact*_ = 3.47 × 10^–7^, *RBFOX1*; rs9938274, *β* = − 0.09, *P*_*interact*_ = 4.00 × 10^–7^, *RBFOX1*; rs1345439, *β* = 0.08, *P*_*interact*_ = 6.33 × 10^–7^, *CBLN1*; rs2507941, *β* = 0.12, *P*_*interact*_ = 8.42 × 10^–7^, *ITGA9*) (Table 3).

Moreover, for potential risk variants of cIMT to young PWH, positive interactions of those variants with HIV infection were also observed among participants under 45 years old (rs2507941, *β* = 0.12, *P*_*interact*_ = 8.42 × 10^–7^; rs6762348, *β* = 0.12, *P*_*interact*_ = 1.93 × 10^–6^; rs62263680, *β* = 0.12, *P*_*interact*_ = 2.45 × 10^–6^; rs62262941, *β* = 0.12, *P*_*interact*_ = 3.13 × 10^–6^; rs77741796, *β* = 0.12, *P*_*interact*_ = 8.89 × 10^–6^; rs148420952, *β* = 0.11, *P*_*interact*_ = 4.05 × 10^–5^; rs35812497, *β* = 0.08, *P*_*interact*_ = 1.14 × 10^–4^; rs10847321, *β* = 0.09, *P*_*interact*_ = 1.69 × 10^–4^; rs111815403, *β* = 0.07, *P*_*interact*_ = 0.002) (Table 3).

Among participants above 45 years old, no interaction term reached the suggestive significant level and the most significant interaction effect was observed in rs11948504 (*β* = 0.19, *P*_*interact*_ = 7.16 × 10^–6^) (Table 3).

### Gene interaction with potential risk factors of SCA among PWH

We also measured the interaction effect of genetic variants and traditional risk factors of SCA including alcohol consumption, tobacco use and increasing BMI category on cIMT among PWH, respectively. One variant at *NKAIN2* (rs9375288, *β* = 0.09, *P*_*interact*_ = 2.31 × 10^–8^) and six variants (rs817856, *β* = 0.39, *P*_*interact*_ = 1.03 × 10^–9^; rs78096022, *β* = 0.41, *P*_*interact*_ = 3.70 × 10^–9^; rs6513469, *β* = 0.50, *P*_*interact*_ = 9.53 × 10^–9^; rs17392147, *β* = 0.83, *P*_*interact*_ = 1.89 × 10^–8^; rs186333, *β* = 0.43, *P*_*interact*_ = 2.70 × 10^–8^; rs78752139, *β* = 0.40, *P*_*interact*_ = 4.28 × 10^–8^) had genome-wide significant interaction with alcohol consumption and tobacco use to cIMT among PWH, respectively (Table 4).

**Table 4:** Association of SNP × traditional risk factors with cIMT among PWH

CHR	SNP	Position (GRCh37)	MAF	Gene	Location	Minor allele	SNP × covariate		SNP		Covariate	
							*β* value^a^	*P* interaction	*β* value^a^	*P *value	*β* value^a^	*P* value
*SNP × alcohol interaction*												
6	rs9375288	124,194,087	0.123	NKAIN2	Intronic	G	0.09	2.31E−08	0.03	8.10E−02	0.01	7.70E−01
12	rs77741796	80,815,650	0.054	PTPRQ	Intergenic	T	0.10	6.45E−06	0.03	2.31E−01	− 0.01	7.68E−01
3	rs6762348	96,683,649	0.058	EPHA6	Intronic	G	0.10	2.23E−05	− 0.01	7.37E−01	− 0.01	8.61E–01
3	rs62262941	96,570,987	0.057	EPHA6	Intronic	C	0.10	3.40E−05	− 0.01	8.26E−01	− 0.01	8.90E−01
3	rs62263680	96,627,491	0.057	EPHA6	Intronic	C	0.10	3.83E−05	− 0.01	8.22E−01	− 0.01	8.81E−01
4	rs148420952	171,482,271	0.051	LINC01612	Intergenic	T	0.09	1.74E−04	0.01	9.23E−01	− 0.01	8.86E−01
12	rs10847321	127,782,099	0.056	LINC02376	Intergenic	A	0.05	1.97E−02	0.01	9.53E−01	0.01	8.51E−01
*SNP × tobacco interaction*												
9	rs817856	110,118,754	0.115	RAD23B	Intergenic	G	0.39	1.03E−09	− 0.02	2.15E−01	− 0.04	1.81E−01
8	rs78096022	132,359,092	0.107	ADCY8	Intergenic	T	0.41	3.70E−09	− 0.03	4.55E−02	− 0.04	2.58E−01
20	rs6513469	58,588,895	0.059	CDH26	3′-UTR	C	0.50	9.53E−09	0.01	7.63E−01	− 0.03	3.73E−01
1	rs17392147	9,060,210	0.054	SLC2A7	Intronic	C	0.83	1.89E−08	0.02	4.43E−01	0.01	9.39E−01
5	rs186333	23,202,461	0.074	CDH12	Intergenic	G	0.43	2.70E−08	− 0.01	6.68E−01	− 0.02	5.47E−01
5	rs78752139	103,593,227	0.092	NUDT12	Intergenic	T	0.40	4.28E−08	− 0.01	9.25E−01	− 0.04	2.88E−01
11	rs111815403	2,553,341	0.061	KCNQ1	Intronic	A	0.22	3.67E−03	0.04	2.64E−02	0.01	8.74E−01
4	rs148420952	171,482,271	0.051	LINC01612	Intergenic	T	− 0.24	1.39E−02	0.08	1.64E−04	0.06	4.87E−02
3	rs2507941	37,536,056	0.064	ITGA9	Synonymous	T	0.30	1.79E−02	0.05	7.73E−03	0.03	4.29E−01
*SNP × BMI category interaction*												
3	rs2507941	37,536,056	0.064	ITGA9	Synonymous	T	0.12	1.87E−04	− 0.21	5.03E−03	0.01	5.19E−01
12	rs10847321	127,782,099	0.056	LINC02376	Intergenic	A	0.11	1.48E−03	− 0.20	1.01E−02	0.01	3.62E−01
11	rs111815403	2,553,341	0.061	KCNQ1	Intronic	A	0.10	2.91E−03	− 0.16	3.41E−02	0.01	3.77E−01
4	rs148420952	171,482,271	0.051	LINC01612	Intergenic	T	0.08	1.69E−02	− 0.11	1.50E−01	0.01	2.86E−01
5	rs35812497	108,563,812	0.089	FER, PJA2	Intergenic	A	0.06	3.58E−02	− 0.09	1.46E−01	0.01	3.63E−01
3	rs6762348	96,683,649	0.058	EPHA6	Intronic	G	0.06	4.18E−02	− 0.09	2.20E−01	0.01	2.24E−01

^a^Assessed by generalized linear model, adjusted for age, sex, smoking status, regular alcohol use, BMI category

For the risk variants of cIMT to young PWH, six of them had a positive interaction effect with alcohol consumption (rs77741796, *β* = 0.10, *P*_*interact*_ = 6.45 × 10^–6^; rs6762348, *β* = 0.10, *P*_*interact*_ = 2.23 × 10^–5^; rs62262941, *β* = 0.10, *P*_*interact*_ = 3.40 × 10^–5^; rs62263680, *β* = 0.10, *P*_*interact*_ = 3.83 × 10^–5^; rs148420952, *β* = 0.09, *P*_*interact*_ = 1.74 × 10^–4^; rs10847321, *β* = 0.05, *P*_*interact*_ = 1.97 × 10^–2^); three of them had a significant interaction with tobacco use (rs111815403, *β* = 0.22, *P*_*interact*_ = 3.67 × 10^–3^; rs148420952, *β* = -0.24, *P*_*interact*_ = 1.39 × 10^–2^; rs2507941, *β* = 0.30, *P*_*interact*_ = 1.79 × 10^–2^) and six of them had a positive interaction effect with increasing BMI category (rs2507941, *β* = 0.12, *P*_*interact*_ = 1.87 × 10^–4^; rs10847321, *β* = 0.11, *P*_*interact*_ = 1.48 × 10^–3^; rs111815403, *β* = 0.10, *P*_*interact*_ = 2.91 × 10^–3^; rs148420952, *β* = 0.08, *P*_*interact*_ = 1.69 × 10^–2^; rs35812497, *β* = 0.06, *P*_*interact*_ = 3.58 × 10^–2^; rs6762348, *β* = 0.06, *P*_*interact*_ = 4.18 × 10^–2^) (Table 4).

Summary statistics results of GWA analyses can be seen in Additional files 2, 3, 4, 5, 6 and 7.

### Association of GRS with SCA

Genetic variants potentially associated with cIMT among PWH under 45 years old were selected to calculated for the unweighted and weighted GRS, and associations with cIMT and binary-cIMT were tested by GLM and logistic regression models, respectively. Both univariable and multivariable regression models were fitted adjusting for age, sex, regular alcohol use, current smoking status and BMI.

The unweighted GRS was significantly associated with cIMT level (*β* = 0.06, *P* < 2 × 10^–16^) and binary-cIMT (*OR* = 1.15, 95%*CI*: 1.04–1.25, *P* = 0.006) among PWH under 45 years old after adjustment (Additional file 1: Table S4). The weighted GRS demonstrated greater specificity for the cIMT level (*β* = 0.51, *P* = 2 × 10^–16^) and binary-cIMT (*OR* = 2.72, 95%*CI*: 1.15–6.42, *P* = 0.023) with greater evidence for association between genetic variants and SCA risk among PWH under 45 years old (Additional file 1: Table S5).

### Functional annotation, gene mapping and gene set analysis

Using three gene mapping strategies in FUMA, we identified 7 genomic risk loci and 15 mapped genes associated with cIMT among PWH under 45 years old (Additional file 1: Tables S6, 7), 6 genomic risk loci and 11 mapped genes among HIV-negative counterparts under 45 years old (Additional file 1: Tables S8, 9).

Among PWH under 45 years old, positional gene mapping aligned SNPs to 5 genes by genomic location, eQTL gene mapping matched SNPs to 8 genes by expression levels they influence, and chromatin interaction mapping annotated SNPs to 4 genes on the basis of 3D DNA-DNA interactions (Additional file 1: Table S7, S10–11). Of note, the variant rs2507941 was also mapped to *SCN5A* gene through chromatin interaction mapping (Additional file 1: Table S7). Eleven genes were notable as they were linked via eQTL mapping or chromatin interactions between two independent genomic risk loci (Additional file 1: Figs. S7–9). Circos plot of chromatin interactions among HIV negative counterparts under 45 years old can be seen in Additional file 1: Figs. S10–13.

Gene-set based analysis was performed to further evaluate the underlying disease mechanisms responsible for the genetic signals. The 2 significant GO biological processes and 6 significant GO molecular functions were identified among PWH under 45 years old (Table 5). Among those gene sets, there were 2 GO gene sets involved in pathogenesis of SCA, including regulation of atrial cardiac muscle cell membrane repolarization (*FDR* = 0.034) and molecular function of protein kinase A (PKA) subunit binding (*FDR* = 0.018). Gene-set results among HIV-negative individuals under 45 years old were also shown in Table 5.

**Table 5:** The significant gene-set analyses for cIMT among participants under 45 years old

Category	GeneSet	N	n	*P*-value	Adjusted *P*	Genes
*PWH under 45 years old*						
GO_bp	regulation of atrial cardiac muscle cell membrane repolarization	8	2	4.75E−06	0.034	KCNQ1, SCN5A
GO_bp	atrial cardiac muscle cell membrane repolarization	11	2	9.33E−06	0.034	KCNQ1, SCN5A
GO_mf	protein kinase A catalytic subunit binding	13	2	1.32E−05	0.018	KCNQ1, PJA2
GO_mf	protein kinase A regulatory subunit binding	20	2	3.22E−05	0.018	KCNQ1, PJA2
GO_mf	protein phosphatase 1 binding	20	2	3.22E−05	0.018	KCNQ1, FER
GO_mf	protein kinase activity	588	4	9.15E−05	0.038	DCLK3, ACVR2B, EPHA6, FER
*HIV-negative participants under 45 years old*						
GWAS catalog reported genes	Hyperopia	6	2	1.09E−06	1.98E−03	RBFOX1, LAMA2
GWAS catalog reported genes	Urinary albumin-to-creatinine ratio in non-diabetics	19	2	1.24E−05	6.44E−03	SOGA3, SOGA3, C6orf58
GWAS catalog reported genes	Spherical equivalent (joint analysis main effects and education interaction)	20	2	1.38E−05	6.44E−03	RBFOX1, LAMA2
GWAS catalog reported genes	Spherical equivalent or myopia (age of diagnosis)	175	3	1.42E−05	6.44E−03	RBFOX1, LAMA2, DPP6
GWAS catalog reported genes	Refractive error	29	2	2.95E−05	1.07E−02	RBFOX1, LAMA2
GWAS catalog reported genes	Waist-to-hip ratio adjusted for BMI	403	3	1.69E−04	4.30E−02	ECHDC1, SOGA3, SOGA3, C6orf58
GWAS catalog reported genes	Intracranial aneurysm	72	2	1.84E−04	4.30E−02	RBFOX1, CARHSP1
GWAS catalog reported genes	Myopia	73	2	1.89E−04	4.30E−02	RBFOX1, LAMA2

Results of gene-set analyses for cIMT among PWH and HIV-negative counterparts under 45 years old. Gene-set analysis used the results from genes mapped in SNP-based analysis as input. N: Genes in Gene Set; n: Genes in Overlap.GO: gene ontology

## Discussion

This study for the first time comprehensively compared and evaluated the genome-wide associated variants and gene-environment interaction in relation to SCA among PWH and HIV-negative individuals in Chinese population, indicating that the host genome had a greater impact on SCA among young PWH than the elder PWH. Nine novel genetic variants, seven genomic loci and 15 mapped genes were identified to be associated with SCA among PWH under 45 years old. Genetic variants had a significant interaction with HIV infection, tobacco use, alcohol use and obesity on the development of SCA. Aggregations of the identified genetic variants were highly associated with SCA among young PWH, as predicted by GRS. Using gene-set analyses, we demonstrated that genetic variants of SCA among PWH under 45 years old pointed towards a role of genes enriched in the biological process of cardiac muscle cell repolarization and molecular function of PKA subunit binding.

We previously reported that SCA could occur early in young HIV-infected adults in the CHART cohort [6]. Based on the same cohort, we found in the present study that one significant variant rs77741796 near *PTPRQ* gene and eight suggestive significant variants at *KCNQ1*/*FER*/*PJA2*/*ITGA9*/*EPHA6* genes were associated with SCA among PWH under 45 years old (Table 2). There was no significant variant associated with SCA among PWH above 45 years old but significant variants can be found among HIV-negative individuals both under and above 45 years old. These results indicated that genetic predisposition may play a crucial role in the development of SCA among young HIV-infected adults instead of old PWH. Elderly PWH population usually have a higher prevalence of multimorbidity and traditional risk factors of CVDs, such as hypertension and metabolic syndrome [6, 32], and thus the role of genetics may be overshadowed. On the contrary, among young PWH with less traditional risk factors and accordingly lower prevalence of CVDs, the role of genetics may become prominent in the debut and progression of atherosclerosis. To what extent and how will the genetic variants impact on the development of SCA among PWH remains to be addressed in longitudinal prospective cohort studies.

In genome-wide interaction analyses among participants under 45 years old, we identified four genetic variants at *RBFOX1*/*CBLN1*/*ITGA9* that had a suggestively significant interaction with HIV infection, indicating an age-specific interaction effect of HIV infection and genetic variant on the development of SCA. rs2507941 at *ITGA9* was associated with cIMT both in GWA among young PWH and genome-wide interaction analyses. The protein that *ITGA9* encodes can improve cell migration and regulate various cellular biological functions [33]. It was also reported that human *ITGA9* was associated with blood pressure and linked to cardiovascular phenotypes [34]. The protein that *RBFOX1* encodes is a muscle-specific isoform of an RNA splicing regulator and previous study identified that regulation of RNA splicing by *RBFOX1* played a crucial role in transcriptome reprogramming during heart failure [35]. *CBLN1* encodes a cerebellum-specific precursor protein that establishes parallel fiber-Purkinje cell synapses [36] but its role in SCA development was firstly reported.

For risk variants of cIMT to young PWH, a positive interaction effect with HIV infection was also identified among all young participants. This might be partially owing to a mixture of accelerated aging due to HIV infection and host genomic effects in the HIV-infected youngsters who had less traditional risk factors for SCA. In addition, risk variants of cIMT to HIV-negative individuals also had a negative interaction with HIV infection among all participants. The significant variants related to SCA among PWH and HIV-negative counterparts under 45 years old were also different. The underlying mechanism might be attributable to the integration of HIV proviral DNA into host genome, which could affect expression of host genes, influence basal and inducible transcription [37, 38], and thus manifest differential associations of genetic variants with SCA between comparable PWH and HIV-negative counterparts.

Genome-wide interaction analyses with traditional risk factors of SCA were also performed among PWH. These analyses identified variants at *NKAIN2/RAD23B/ADCY8/CDH26/SLC2A7/CDH12/NUDT12* had a genome-wide significant interaction with alcohol consumption or tobacco use (Table 4). Previous study also revealed the strong association of *NKAIN2* with alcohol dependence [39] and nicotine dependence [40]. The protein that *RAD23B* encodes is shown to elevate the nucleotide excision activity of 3-methyladenine-DNA glycosylase and plays a role in DNA damage recognition in base excision repair [41], the latter of which was usually caused by tobacco usage [42]. It was also reported that overexpression of a neuronal *ADCY8* in sinoatrial node markedly impacted on heart rate and rhythm [43]. Cadherins (CDHs) formed adherens junctions and were known stabilizers of atherosclerotic plaques [44]. Overexpression of *CDH12* and *CDH26* might be related to myocardial infarction and progression of atherosclerosis [44]. *SLC2A7* encodes a protein that catalyzes the uptake of sugars [45] through facilitated diffusion while *NUDT12* regulates the concentrations of individual nucleotides [46], but their links to SCA were first reported in our study. Potential risk variants of cIMT to young PWH also had an interaction with alcohol consumption, tobacco use and obesity. These results strongly highlight the importance of controlling traditional risk factors of SCA, such as reducing alcohol use, smoking cessation and maintaining a good weight among PWH carrying high-risk alleles in an attempt to reduce SCA risk.

Using functional annotation of associated genetic variants, we found variants at *KCNQ1* and *SCN5A* were associated with SCA among PWH under 45 years old. The *KCNQ1* gene encodes a voltage-gated potassium channel required for repolarization phase of the cardiac action potential [47]. A cohort study in Japan has reported that SNPs at *KCNQ1* were significantly associated with coronary epicardial endothelial dysfunction [48]. Animal experiment has confirmed that an imprinted antisense IncRNA in the *KCNQ1* gene promotes macrophage lipid accumulation and accelerates the development of atherosclerosis through the miR-452-3p/HDAC3/ABCA1 pathway [8]. Protein encoded by *SCN5A* was primarily found in cardiac muscle and defects in this gene have been associated with atrial fibrillation (AF) and cardiomyopathy [49]. Previous study also indicated variants at *SCN5A* were related to increased AF risk and PR interval [50] but its relation to SCA was firstly reported.

Moreover, three SNPs-rs6762348, rs62263680 and rs62262941 located at *EPHA6* on chromosome 3 were identified to be associated with SCA among PWH under 45 years old. *EPHA6* gene is predicted to enable transmembrane-ephrin receptor activity and is found to be associated with insulin signaling [51] and blood pressure phenotype [52], which are the known risk factors of atherosclerosis. We also identified that genetic variants at *ITGA9*, *FER*, *PJA2*, *PTPRQ* genes were significantly associated with SCA among PWH under 45 years old. Variants near *PTPRQ* reached genome-wide significance to cIMT among young PWH; this gene encodes a member of the type III receptor-like protein-tyrosine phosphatase family, playing roles in cellular proliferation and differentiation [53], which might have a link to cardiovascular disease [54]. *FER* regulated cell–cell adhesion and absence of *FER* protein tyrosine kinase could induce epithelial barrier dysfunction [55] which was regarded as a hallmark of many human panvascular diseases, including atherosclerosis, hypertension and diabetes [56]. One study demonstrated the association of *PJA2* with atherosclerosis through protein–protein interaction network analysis [57]. The unweighted and weighted GRSs were significantly associated with SCA among PWH under 45 years old, which might be used as the predictive biomarker panel of SCA among young HIV-infected adults.

The gene set analyses revealed that genes related to SCA among PWH under 45 years old were enriched in regulation of atrial cardiac muscle cell membrane repolarization and molecular function of protein kinase A (PKA) catalytic subunit binding. *KCNQ1* and *SCN5A* participated in the regulation of atrial cardiac muscle cell membrane repolarization which was involved in the process that modulates the establishment or extent of a membrane potential in the polarizing direction towards the resting potential in an atrial cardiomyocyte [58]. Dysregulation of atrial cardiac muscle cell membrane repolarization is related to long QT syndrome, sudden cardiac death, cardiac death and death from any cause [59–61]. *KCNQ1* and *PJA2* were involved in the catalytic subunit binding of PKA which is one of the master regulatory molecules in the heart. It has been reported that persistent activation of PKA signaling was linked to pathological hypertrophy and the progression to heart failure [62].

To our knowledge, this is the largest GWAS of SCA among comparative PWH and HIV-negative counterparts in Asia, and is the first that measured the genome-wide interaction effect of environmental factors and genetic variants on SCA. Nevertheless, our study has several limitations. First, replication study was not conducted, which may reduce the robustness of our results to some extent. However, using the stringent *P*-value could reduce the false discovery rate and candidate SNPs were presented for future validation. Second, since all genetic data were available within one cohort and were obtained using a single chip, no imputation of SNP genotypes was performed. Results of imputation analyses will also be reported in future work. Last, sample size for PWH under 45 years old was relatively small, although genome-wide significant variants were still identified. Future studies with a larger sample size are needed to validate these results.

## Conclusion

In summary, the present GWAS indicated a greater impact of host genome on SCA among young Chinese PWH, as well as the interaction effects between genetic variants and environmental factors on HIV-related SCA development. Nine genetic variants, seven genomic loci and 15 mapped genes were identified to be associated with SCA among PWH under 45 years old. Pathways related to biological processes of atrial cardiac muscle cell membrane repolarization and molecular function of PKA subunit binding were implicated in pathogenesis of SCA in HIV-infected youngsters. Furthermore, the identified gene-environment interaction on SCA among PWH might be useful for discovering high-risk individuals for the prevention of SCA, particularly among those with tobacco use and alcohol consumption. The current study provides new clues for the causal mechanism of SCA among young Chinese HIV-infected adults, and is the starting point of precision intervention targeting HIV-related atherosclerosis.

## Supplementary Information

**Additional file 1**: Supplemental material.

**Additional file 2**: Summary statistics of GWAS in relation to cIMT among all HIV negative control.

**Additional file 3**: Summary statistics of GWAS in relation to cIMT among all PWH.

**Additional file 4**: Summary statistics of GWAS in relation to cIMT among HIV negative control above 45 years old.

**Additional file 5**: Summary statistics of GWAS in relation to cIMT among HIV negative control under 45 years old.

**Additional file 6**: Summary statistics of GWAS in relation to cIMT among PWH above 45 years old.

**Additional file 7**: Summary statistics of GWAS in relation to cIMT among PWH under 45 years old.

## Data Availability

All the data in the paper or in the supplementary materials are free to obtain. Raw data that support the findings of this study are available from the corresponding author upon reasonable request.

## Abbreviations

GWAS: Genome-wide association study
SCA: Subclinical atherosclerosis
PWH: People with HIV
CVDs: Cardiovascular diseases
ART: Antiretroviral therapy
SNP: Single nucleotide polymorphism
LD: Linkage disequilibrium
NCDs: Non-communicable diseases
BP: Blood pressures
BMI: Body mass index
WHR: Waist to hip ratio
SBP: Systolic blood pressure
DBP: Diastolic blood pressure
TC: Total cholesterol
TG: Triglyceride
HDL: High density lipoprotein
LDL: Low density lipoprotein
MS: Metabolic syndrome
cIMT: Carotid intima-media thickness
PCA: Principal component analysis
PCs: Principal components
LMM: Linear mixed model
GLMM: Generalized linear mixed model
QQ plot: Quantile–quantile plot
GRS: Genetic risk scores
FUMA: Functional mapping and annotation
eQTL: Expression quantitative trait loci
KEGG: Kyoto Encyclopedia of Genes and Genomes
GO: Geno oncology
aOR: Adjusted odds ratio
CI: Confidence interval
