# Supplementary material for: Genome-wide associated variants of subclinical atherosclerosis among young people with HIV and gene-environment interactions
Source: J Transl Med. 2022 Dec 20;20:609. doi: 10.1186/s12967-022-03817-6 (PMC9764595; doi:10.1186/s12967-022-03817-6)
Supplement: Supplementary file 1 — Additional file 1: Supplemental material. [file 12967_2022_3817_MOESM1_ESM.docx]

***Additional file***

**Methods**

**Functional annotation**

All known SNPs in the 1000 genome data that have (r^2^>0.6) with any of the independent significant SNP were included for annotation, and the region containing all of these ‘candidate SNPs’ was considered to be a single independent genomic locus. All LD information was calculated from 1000G phase3 East Asian population^1^.

Functional consequences for the SNPs were obtained by performing ANNOVAR gene-based annotation using Ensembl genes^2^. SNPs were matched according to chromosome, position, reference, and alternative alleles, and were annotated by CADD scores (scores>12.37 indicate deleterious SNP)^3^, RegulomeDB scores^4^ (lower scores indicate higher potentiality of regulatory function), and by chromatin states predicted by hidden Markov model based on 5 chromatin marks for 127 epigenomes in the Roadmap Epigenomics Project (lower scores≤7 represent higher accessibility of the genomic regions). CADD scores integrate diverse annotations into a single measure that correlates with pathogenicity, disease severity, experimentally measured regulatory effects and complex trait associations.

**Gene mapping**

Significant loci obtained by the GWAS were mapped to genes in FUMA using three strategies:

1. Positional mapping mapped SNPs to genes based on physical distance (within a 10-kb window) from known protein-coding genes in the human reference assembly (GRCh37/hg19);
2. eQTL mapping mapped SNPs to genes with which they showed a significant eQTL association (i.e., allelic variation at the SNP is associated with the expression level of that gene). eQTL mapping uses information from 45 tissue types in 3 data repositories (GTEx^5^, Blood eQTL brower^6^, BIOS QTL browser^7^) and is based on cis-eQTLs that can map SNPs to genes up to 1Mb away. We used a false discovery rate (FDR) of 0.05 to define significant eQTL associations;
3. Chromatin interaction mapping mapped SNPs to the promoter regions of genes based on significant chromatin interactions. This type of mapping is a 3D DNA-DNA interaction between the SNP region and a gene region, without a distance boundary. FUMA currently contains Hi-C data for 21 tissue/cell types from the study^8^. Because chromatin interactions are often defined in a certain resolution, such as 40 kb, an interacting region can span multiple genes. If a SNP is located in a region that interacts with a region containing multiple genes, it will be mapped to each of those genes. To prioritize candidate genes, we integrated predicted enhancers and promoters in the heart tissue from the Roadmap Epigenomics Project^9^. Using the information, FUMA selected chromatin interactions for which one region involved in the interaction overlapped with predicted enhancers and the other overlapped with predicted promoters 250 bp upstream and 500 bp downstream of the TSS of a gene. We used a false discovery rate of 1×10^-6^ to define significant interactions.

References

1. Genomes Project, C. *et al.* A global reference for human genetic variation. *Nature* **526**, 68-74 (2015).

2. Wang, K., Li, M. & Hakonarson, H. ANNOVAR: functional annotation of genetic variants from high-throughput sequencing data. *Nucleic Acids Res* **38**, e164 (2010).

3. Kircher, M. *et al.* A general framework for estimating the relative pathogenicity of human genetic variants. *Nat Genet* **46**, 310-5 (2014).

4. Boyle, A.P. *et al.* Annotation of functional variation in personal genomes using RegulomeDB. *Genome Res* **22**, 1790-7 (2012).

5. Consortium, G.T. Human genomics. The Genotype-Tissue Expression (GTEx) pilot analysis: multitissue gene regulation in humans. *Science* **348**, 648-60 (2015).

6. Westra, H.J. *et al.* Systematic identification of trans eQTLs as putative drivers of known disease associations. *Nat Genet* **45**, 1238-1243 (2013).

7. Zhernakova, D.V. *et al.* Identification of context-dependent expression quantitative trait loci in whole blood. *Nat Genet* **49**, 139-145 (2017).

8. Schmitt, A.D. *et al.* A Compendium of Chromatin Contact Maps Reveals Spatially Active Regions in the Human Genome. *Cell Rep* **17**, 2042-2059 (2016).

9. Roadmap Epigenomics, C. *et al.* Integrative analysis of 111 reference human epigenomes. *Nature* **518**, 317-30 (2015).

**Results**

**Summary statistics of association analyses**

The results can be seen in the files “association analyses results”. We provided GWA summary statistics of cIMT conducted among all PWH, all HIV negative controls, PWH above 45 years old, PWH under 45 years old, HIV negative control above 45 years old and HIV negative control under 45 years old. The files of GWAS had the suffix of “.fastGWA”.

We also provided summary statistics of genome-wide interaction analyses with HIV infection conducted among all participants, among participants under 45 years old and among participants above 45 years old. The summary statistics of genome-wide interaction analyses with alcohol consumption, tobacco use and BMI among PWH were also provided. The files of genome-wide interaction analyses had the suffix of “.assoc.linear”.


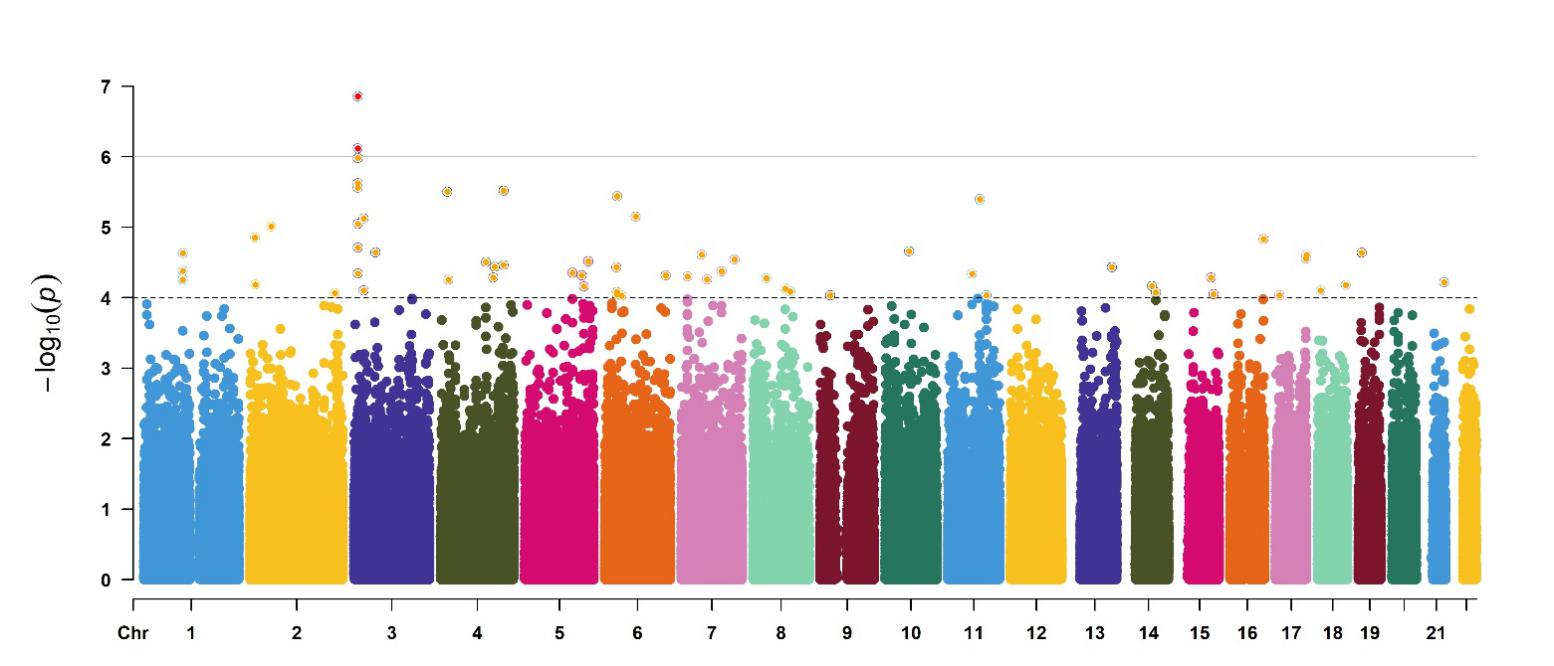


Figure S1. Manhattan plot of SNPs associated with cIMT among CHART cohort


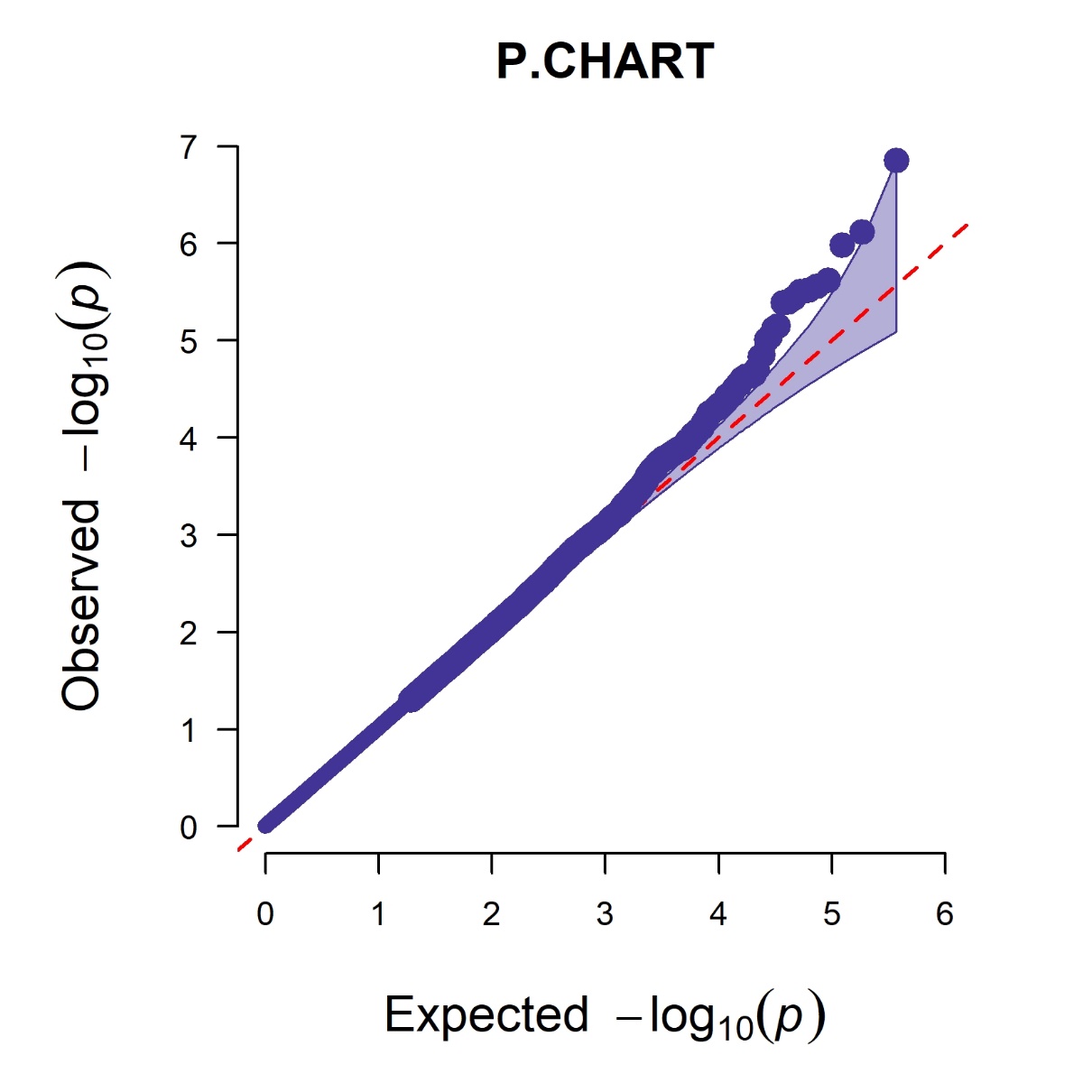


λ_CHART_=1.013

Figure S2. qq plot of SNPs associated with cIMT among CHART cohort


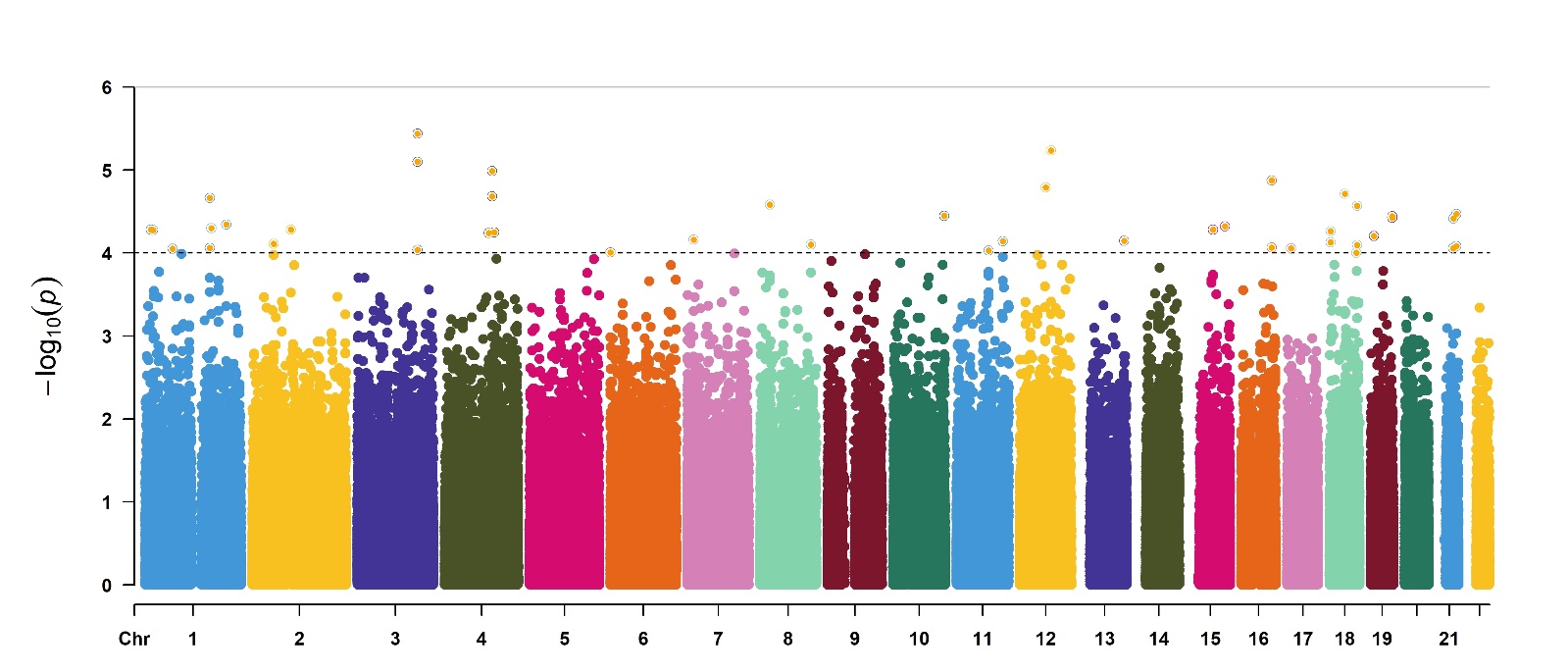


Figure S3. Manhattan plot of SNPs associated with cIMT among people living HIV


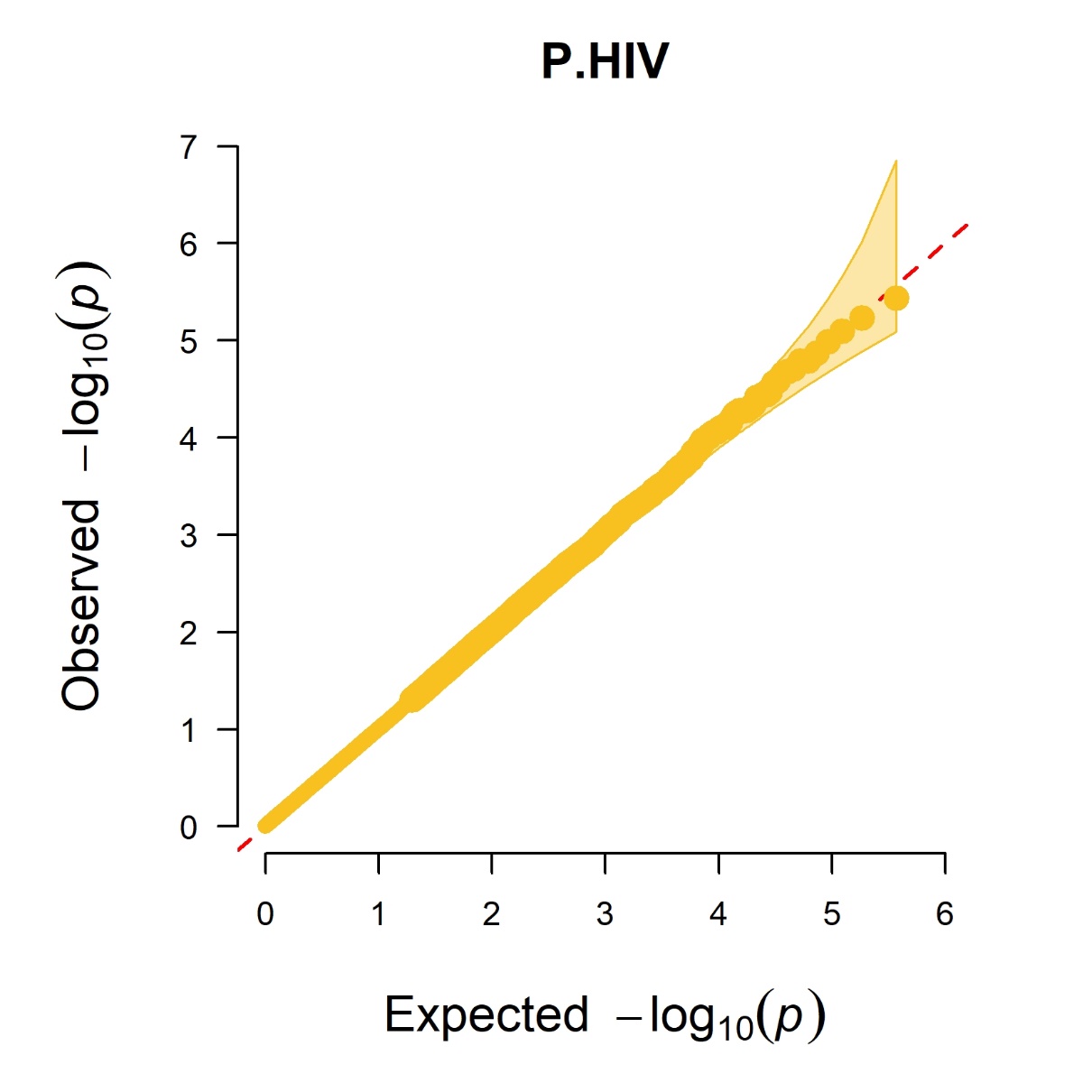


λ_HIV_ =1.029

Figure S4. qq plot of SNPs associated with cIMT among people living HIV


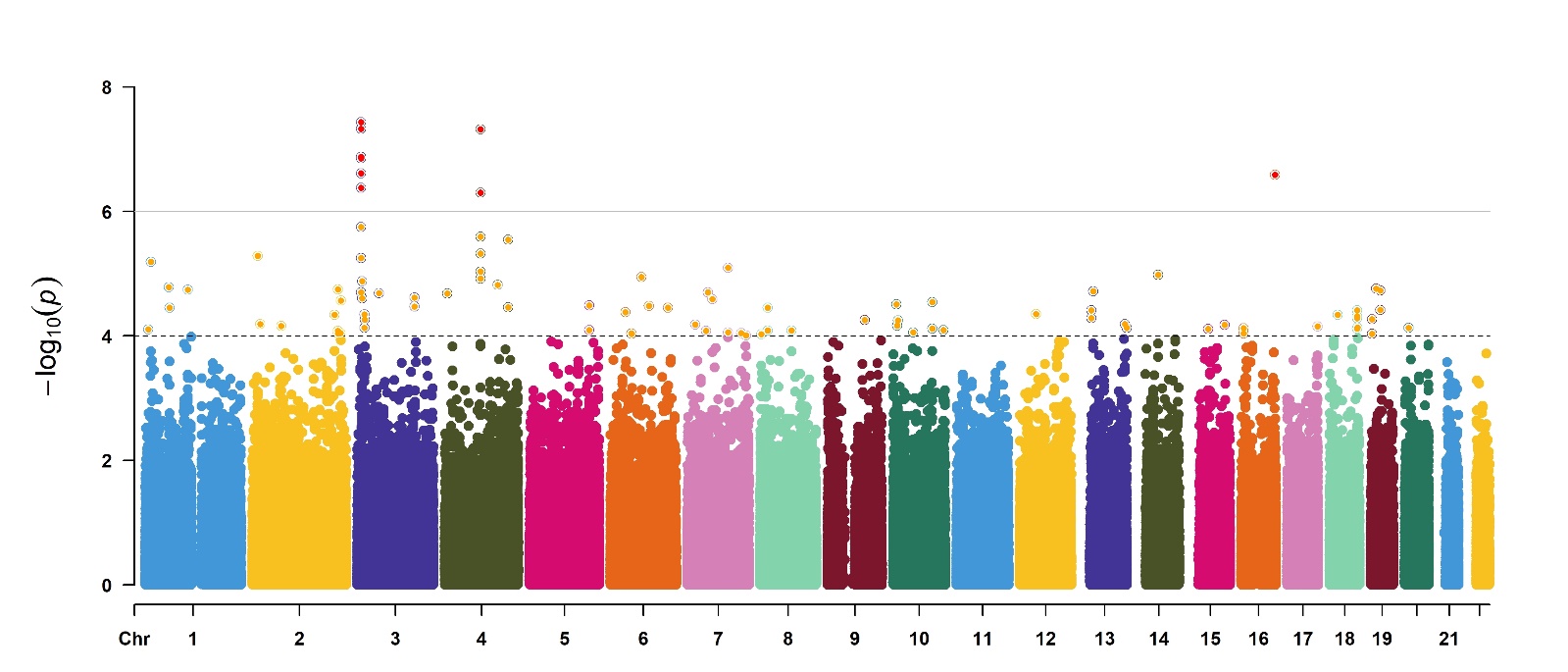


Figure S5. Manhattan plot of SNPs associated with cIMT among HIV-negative controls


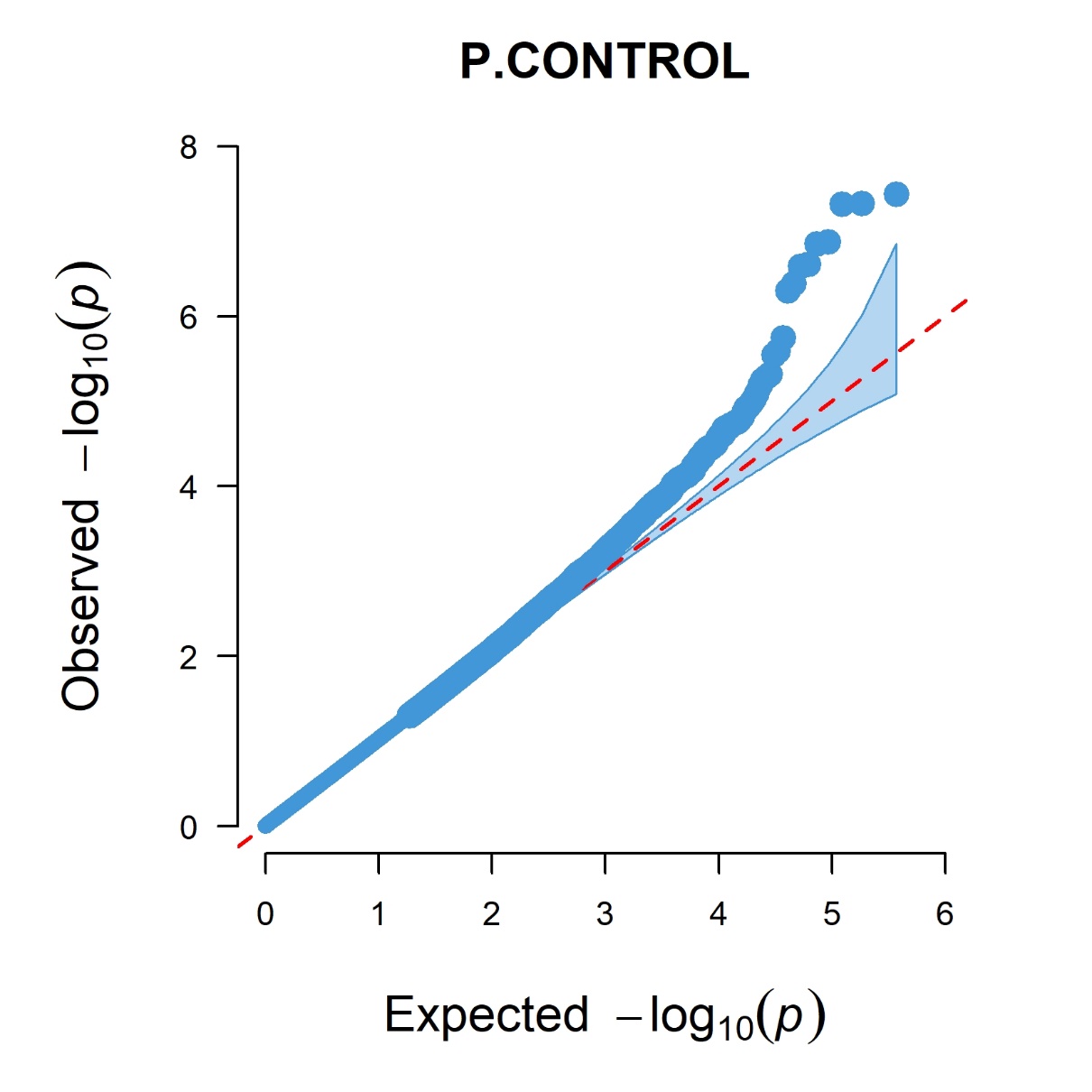


λ_CONTROL_=1.046

Figure S6. qq plot of SNPs associated with cIMT among HIV-negative controls


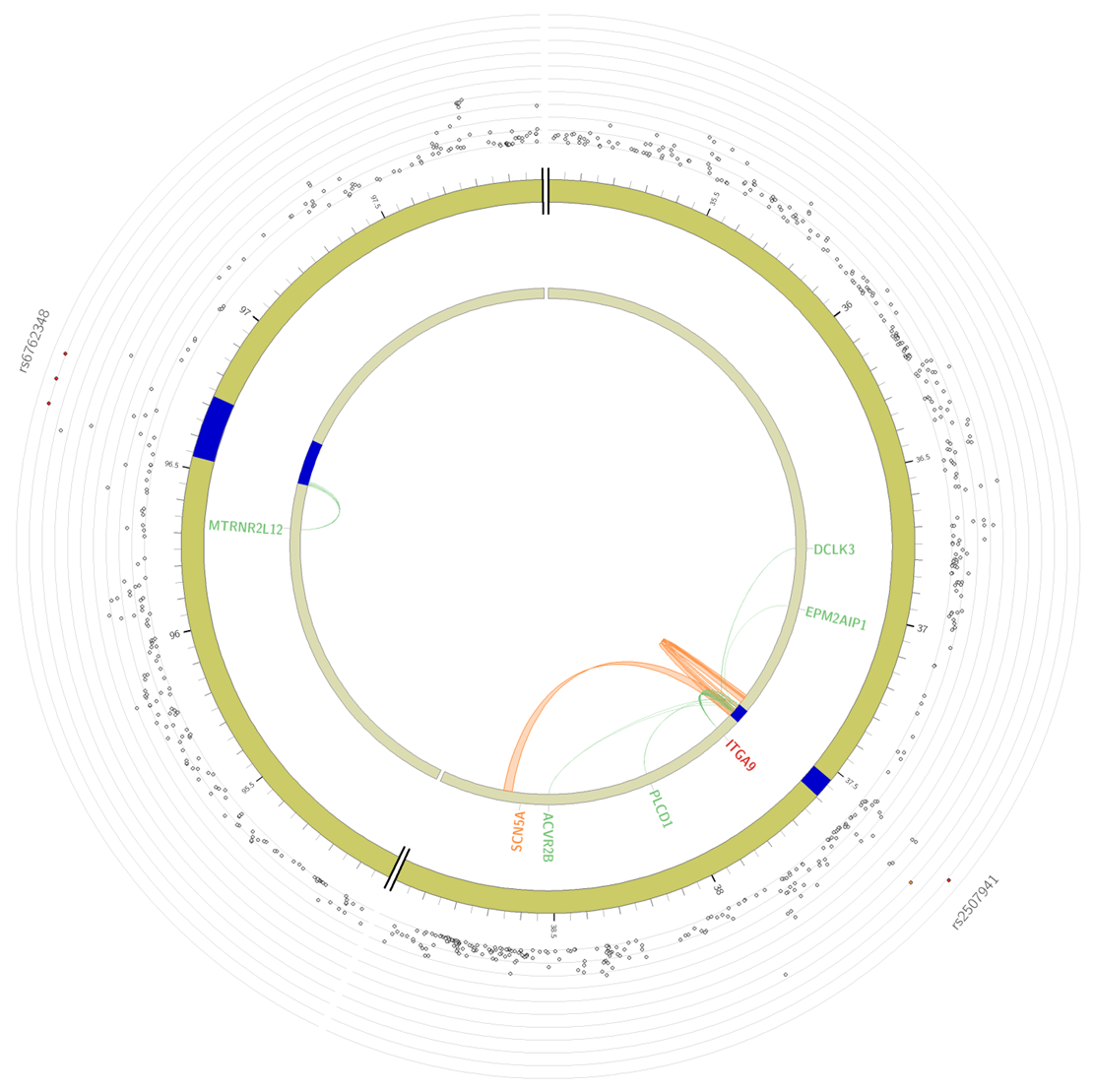


Figure S7. Genomic risk loci, eQTL associations and chromatin interactions identified in GWAS of cIMT among PWH under 45 years old. Circos plots showing genes on chromosomes 3 that were implicated through the genomic risk loci (blue areas) in GWAS by chromatin interaction mapping (orange font and orange lines connecting two interacting regions), eQTL mapping (green font and green lines connecting an eQTL SNP to its associated gene), or by both chromatin interaction and eQTL mapping (red font). The outer layer shows a Manhattan plot containing the -log_10_P-value of each SNP in the GWAS analysis of cIMT, with genome-wide significant SNPs colored according to LD patterns with the lead SNP. (n=1348 individuals)


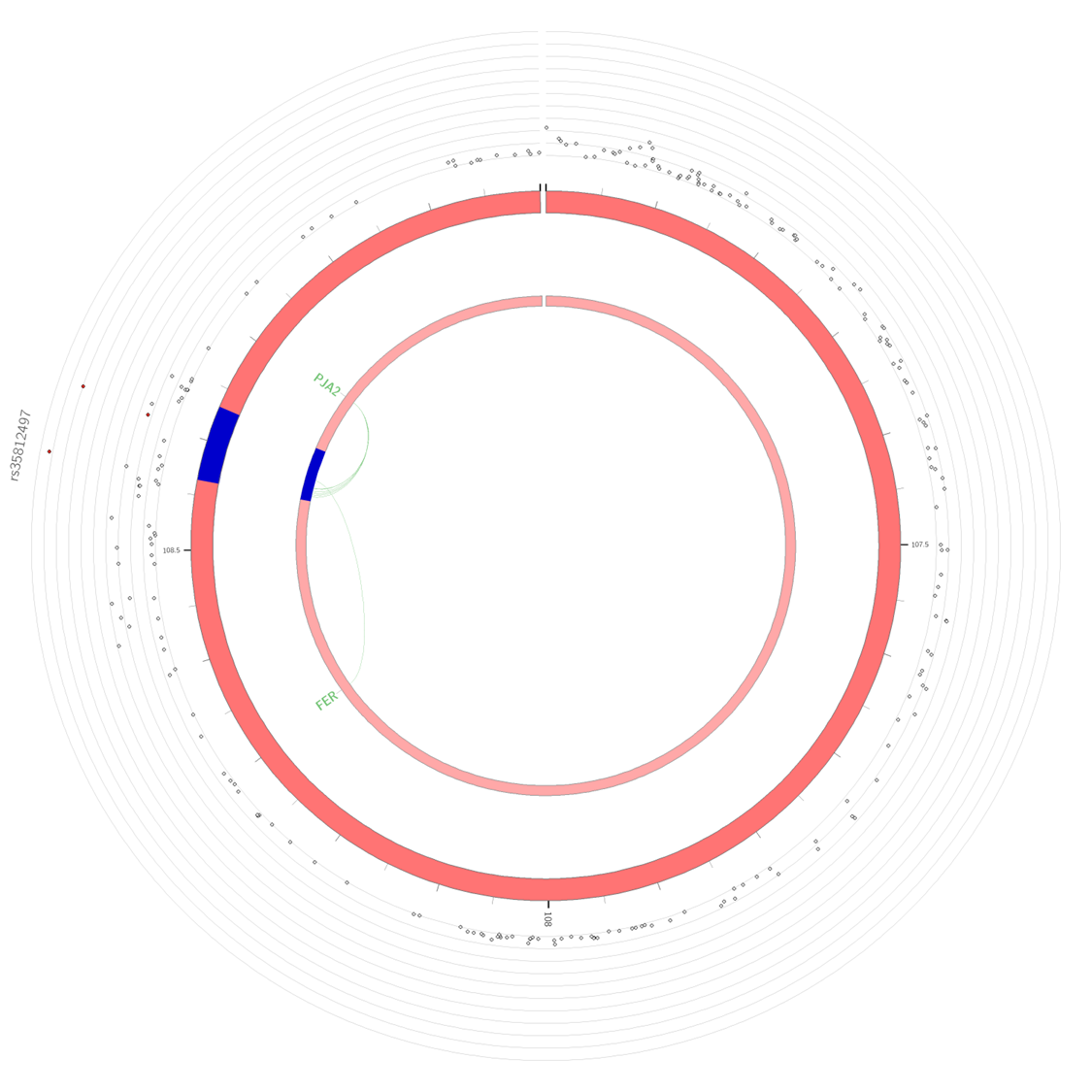


Figure S8. Genomic risk loci, eQTL associations and chromatin interactions identified in GWAS of cIMT among PWH under 45 years old. Circos plots showing genes on chromosomes 5 that were implicated through the genomic risk loci (blue areas) in GWAS by chromatin interaction mapping (orange font and orange lines connecting two interacting regions), eQTL mapping (green font and green lines connecting an eQTL SNP to its associated gene), or by both chromatin interaction and eQTL mapping (red font). The outer layer shows a Manhattan plot containing the -log_10_P-value of each SNP in the GWAS analysis of cIMT, with genome-wide significant SNPs colored according to LD patterns with the lead SNP. (n=1348 individuals)


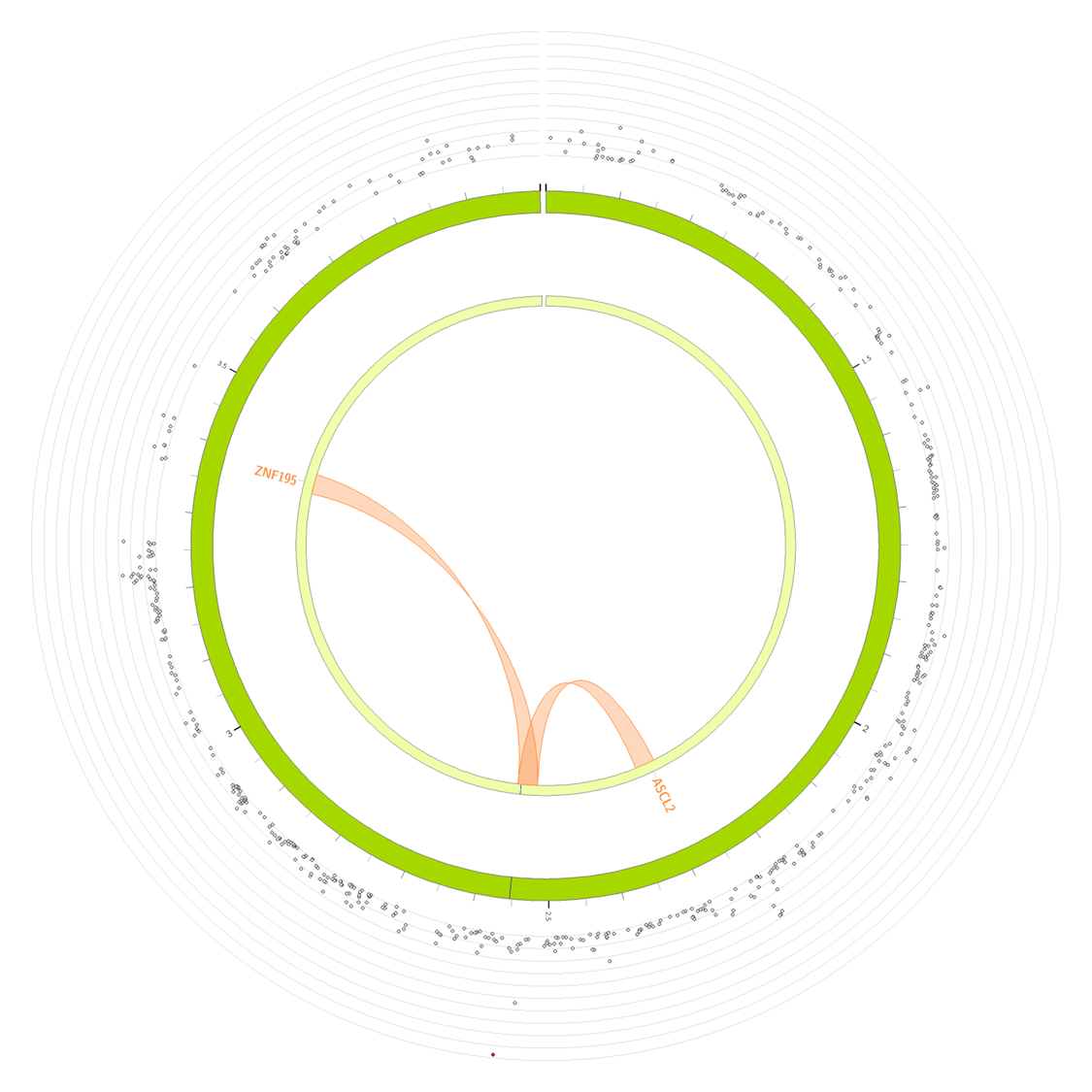


Figure S9. Genomic risk loci, eQTL associations and chromatin interactions identified in GWAS of cIMT among PWH under 45 years old. Circos plots showing genes on chromosomes 11 that were implicated through the genomic risk loci (blue areas) in GWAS by chromatin interaction mapping (orange font and orange lines connecting two interacting regions), eQTL mapping (green font and green lines connecting an eQTL SNP to its associated gene), or by both chromatin interaction and eQTL mapping (red font). The outer layer shows a Manhattan plot containing the -log_10_P-value of each SNP in the GWAS analysis of cIMT, with genome-wide significant SNPs colored according to LD patterns with the lead SNP. (n=1348 individuals)


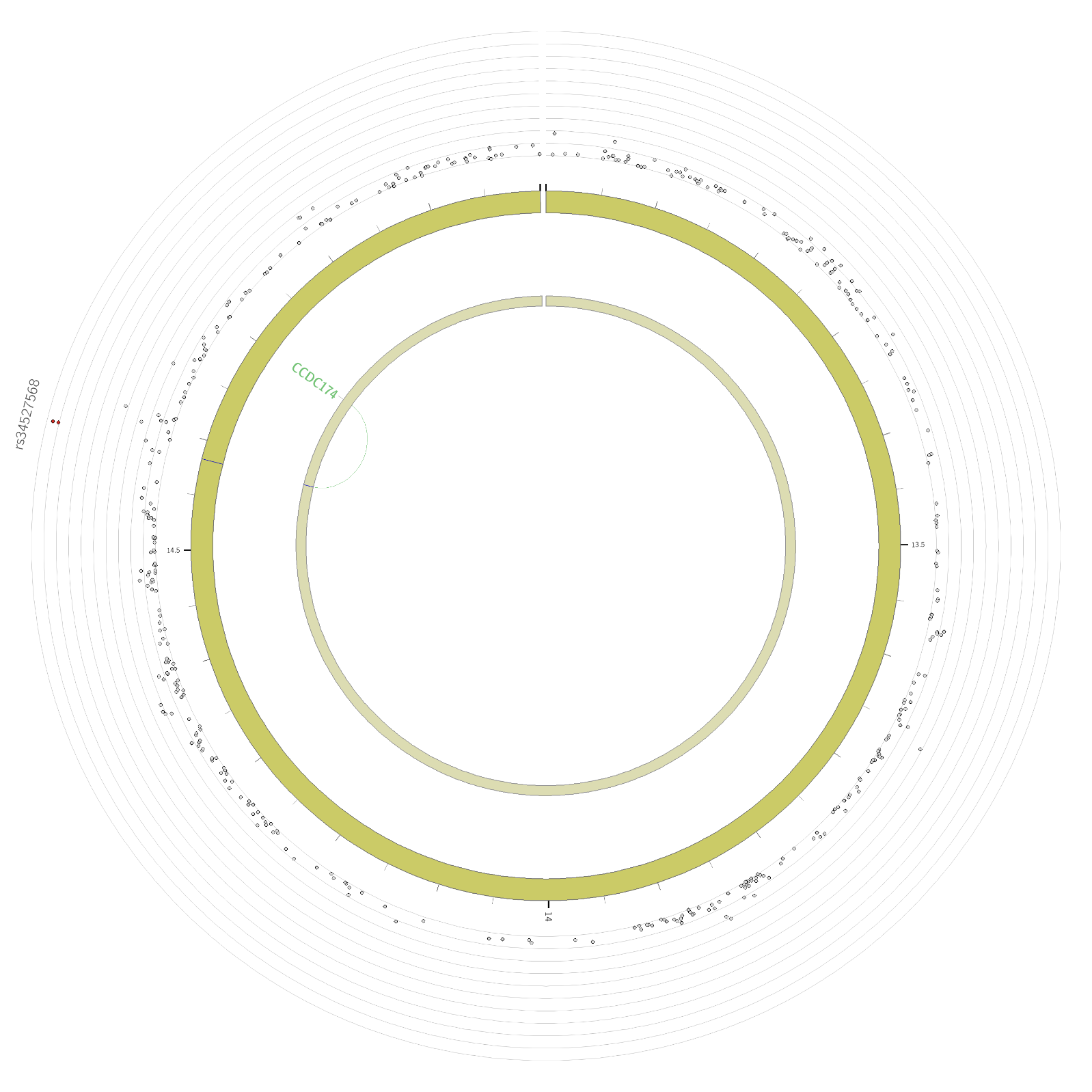


Figure S10. Genomic risk loci, eQTL associations and chromatin interactions identified in GWAS of cIMT among HIV negative participants under 45 years old. Circos plots showing genes on chromosomes 3 that were implicated through the genomic risk loci (blue areas) in GWAS by chromatin interaction mapping (orange font and orange lines connecting two interacting regions), eQTL mapping (green font and green lines connecting an eQTL SNP to its associated gene), or by both chromatin interaction and eQTL mapping (red font). The outer layer shows a Manhattan plot containing the -log_10_P-value of each SNP in the GWAS analysis of cIMT, with genome-wide significant SNPs colored according to LD patterns with the lead SNP. (n=2791 individuals)


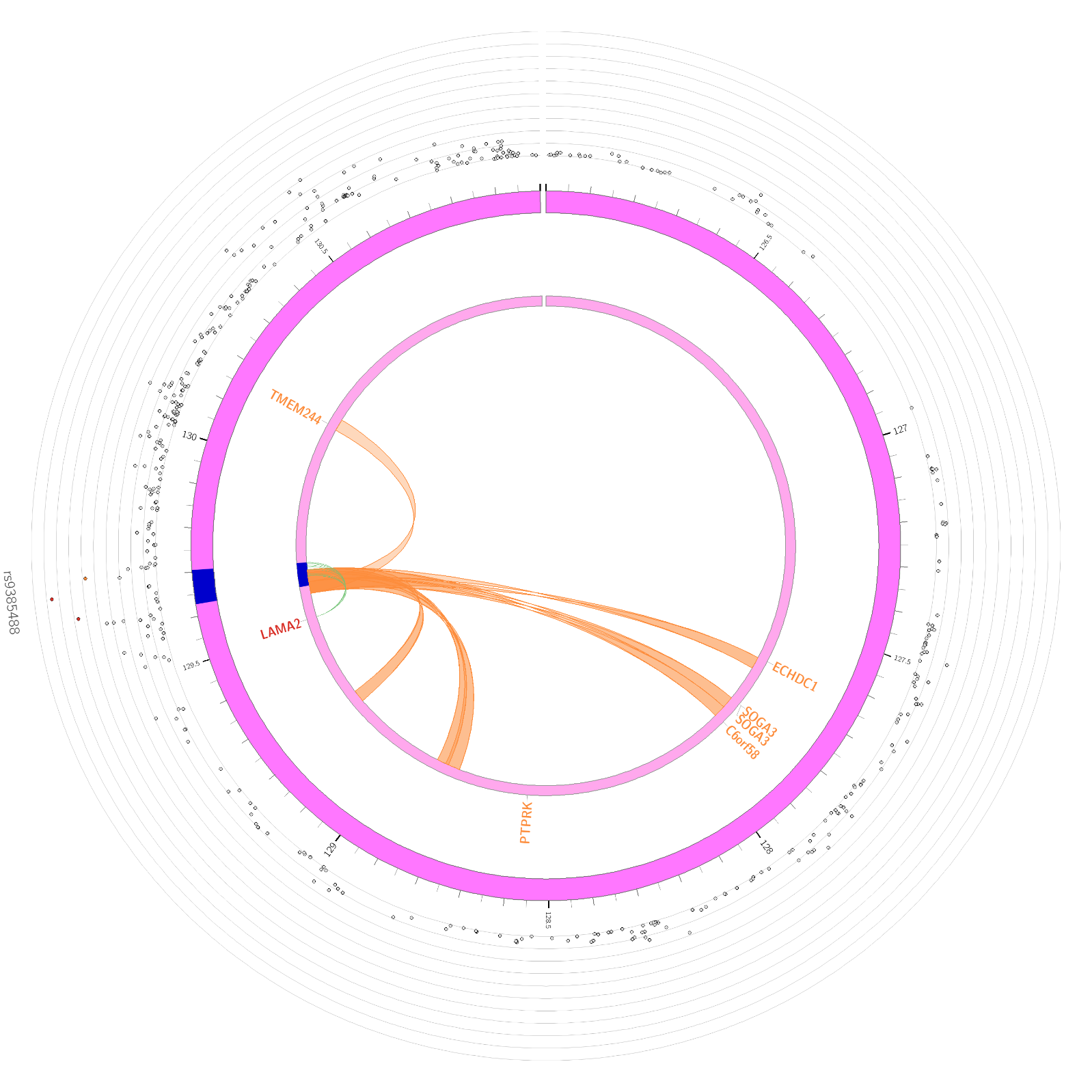


Figure S11. Genomic risk loci, eQTL associations and chromatin interactions identified in GWAS of cIMT among HIV negative participants under 45 years old. Circos plots showing genes on chromosomes 6 that were implicated through the genomic risk loci (blue areas) in GWAS by chromatin interaction mapping (orange font and orange lines connecting two interacting regions), eQTL mapping (green font and green lines connecting an eQTL SNP to its associated gene), or by both chromatin interaction and eQTL mapping (red font). The outer layer shows a Manhattan plot containing the -log_10_P-value of each SNP in the GWAS analysis of cIMT, with genome-wide significant SNPs colored according to LD patterns with the lead SNP.(n=2791 individuals)


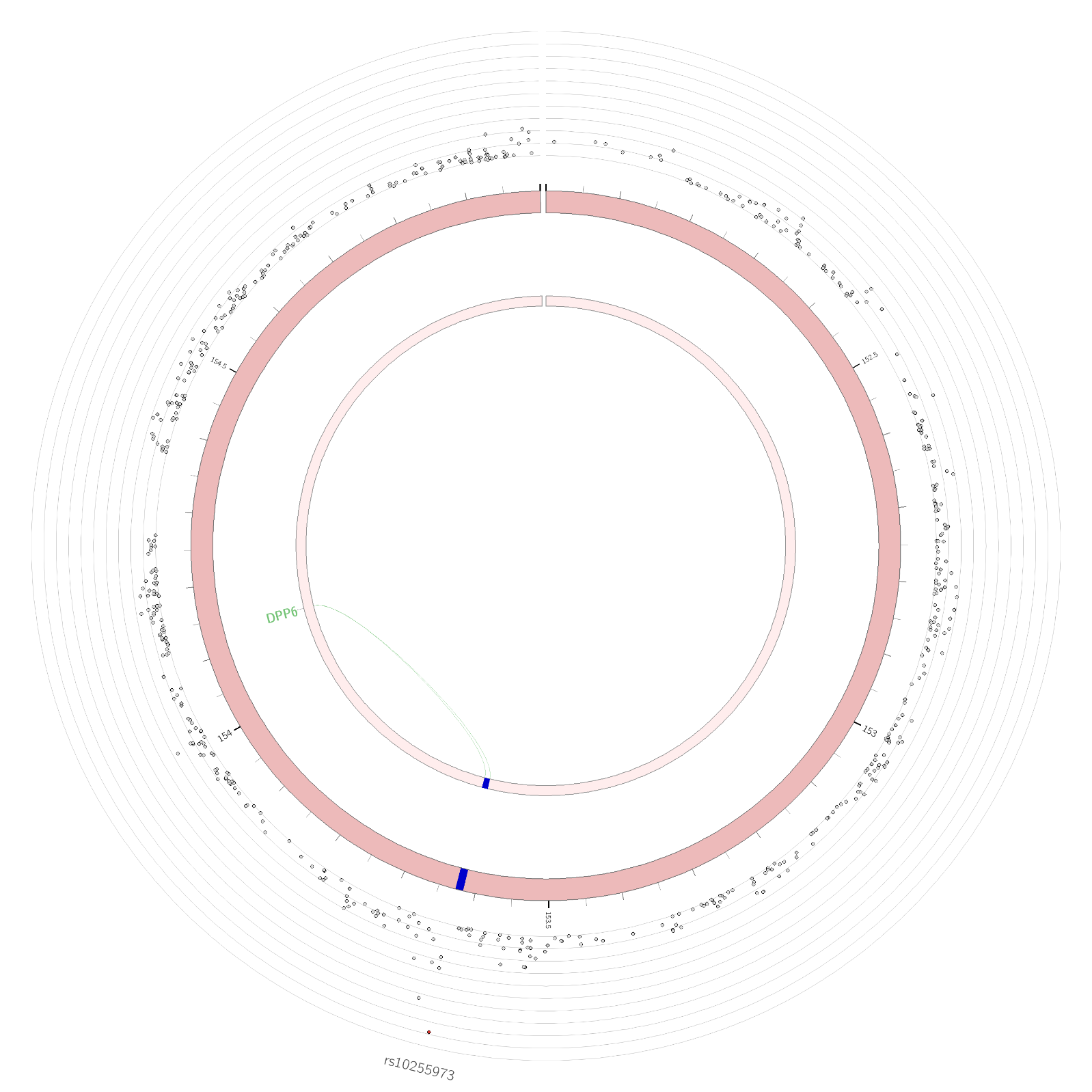


Figure S12. Genomic risk loci, eQTL associations and chromatin interactions identified in GWAS of cIMT among HIV negative participants under 45 years old. Circos plots showing genes on chromosomes 7 that were implicated through the genomic risk loci (blue areas) in GWAS by chromatin interaction mapping (orange font and orange lines connecting two interacting regions), eQTL mapping (green font and green lines connecting an eQTL SNP to its associated gene), or by both chromatin interaction and eQTL mapping (red font). The outer layer shows a Manhattan plot containing the -log_10_P-value of each SNP in the GWAS analysis of cIMT, with genome-wide significant SNPs colored according to LD patterns with the lead SNP. (n=2791 individuals)


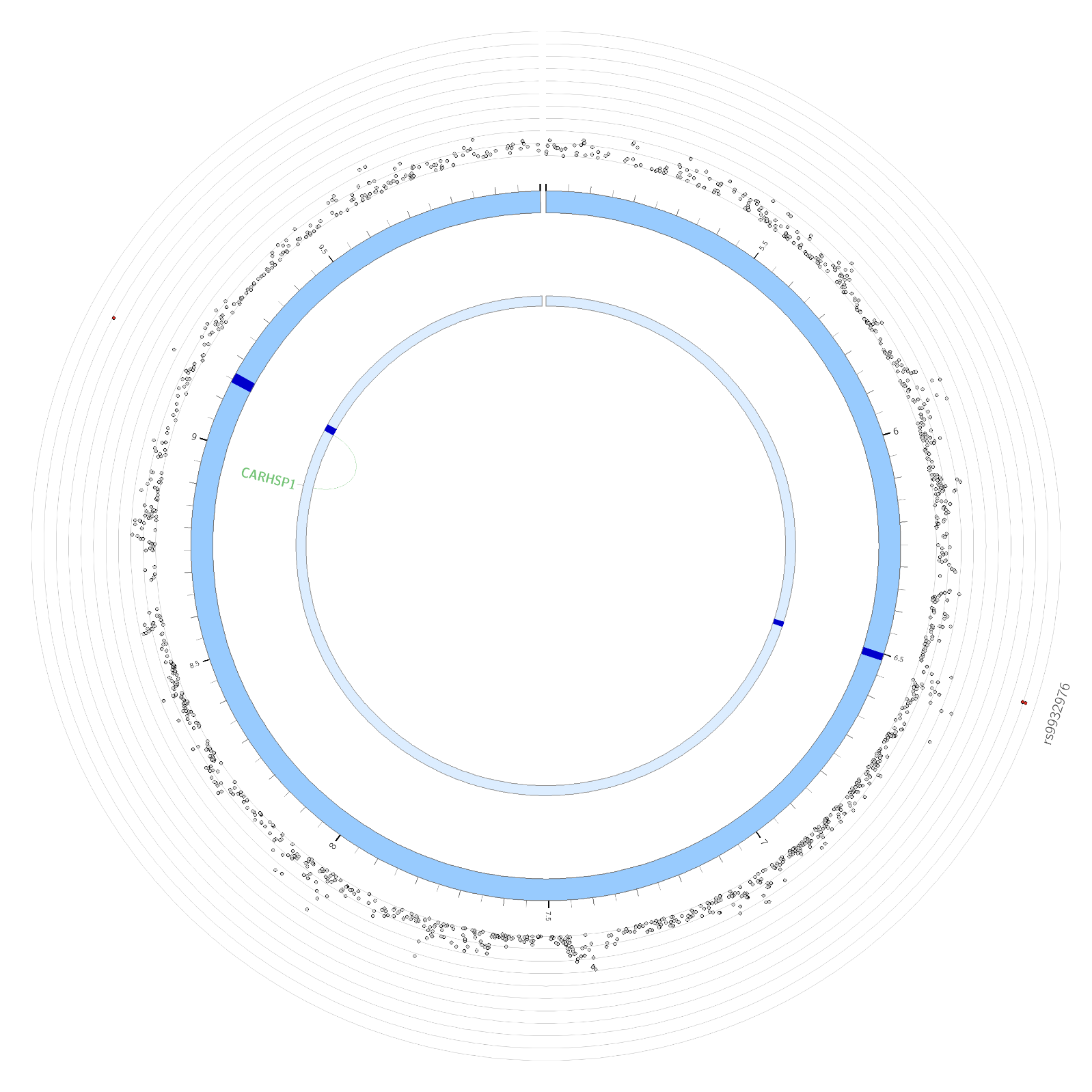


Figure S13. Genomic risk loci, eQTL associations and chromatin interactions identified in GWAS of cIMT among HIV negative participants under 45 years old. Circos plots showing genes on chromosomes 16 that were implicated through the genomic risk loci (blue areas) in GWAS by chromatin interaction mapping (orange font and orange lines connecting two interacting regions), eQTL mapping (green font and green lines connecting an eQTL SNP to its associated gene), or by both chromatin interaction and eQTL mapping (red font). The outer layer shows a Manhattan plot containing the -log_10_P-value of each SNP in the GWAS analysis of cIMT, with genome-wide significant SNPs colored according to LD patterns with the lead SNP. (n=2791 individuals)

| **Table S1. Association of baseline characteristics and subclinical atherosclerosis among CHART** | | | | | |
| --- | --- | --- | --- | --- | --- |
| **Characteristics** | **crude OR (95%CI)** | **P value^a^** |  | **adjusted OR (95%CI)** | **P value^b^** |
| Age (years) |  |  |  |  |  |
| 18~29 | 1.00 |  |  | 1.00 |  |
| 30~44 | 2.58 (2.07, 3.22) | <0.001 |  | 2.55 (2.03, 3.20) | <0.001 |
| 45~59 | 8.30 (6.70, 10.27) | <0.001 |  | 8.22 (6.58, 10.26) | <0.001 |
| 60~89 | 30.24 (24.13, 37.90) | <0.001 |  | 29.83 (23.57, 37.75) | <0.001 |
| Male | 1.57 (1.40, 1.76) | <0.001 |  | 1.51 (1.30, 1.76) | <0.001 |
| BMI (kg/m3) (n=7900) |  |  |  |  |  |
| <18.5 | 0.77 (0.63, 0.95) | 0.014 |  | 0.98 (0.77, 1.26) | 0.889 |
| 18.5-24.0 | 1.00 |  |  | 1.00 |  |
| >24 | 1.39 (1.26, 1.53) | <0.001 |  | 1.38 (1.22, 1.55) | <0.001 |
| Smoking status |  |  |  |  |  |
| Never | 1.00 |  |  | 1.00 |  |
| Previous | 2.80 (2.41, 3.26) | <0.001 |  | 1.25 (1.04, 1.50) | 0.020 |
| Current | 1.41 (1.27, 1.57) | <0.001 |  | 1.17 (1.02, 1.35) | 0.024 |
| Regular alcohol use (n=7888) |  |  |  |  |  |
| Yes | 2.03 (1.75, 2.34) | <0.001 |  | 1.16 (0.97, 1.37) | 0.102 |
| No | 1.00 |  |  | 1.00 |  |
| HIV infection |  |  |  |  |  |
| Yes | 1.44 (1.30, 1.59) | <0.001 |  | 1.77 (1.56, 2.00) | <0.001 |
| No | 1.00 |  |  | 1.00 |  |
| Dyslipidemia |  |  |  |  |  |
| Yes | 1.41 (1.28, 1.55) | <0.001 |  | / |  |
| No | 1.00 |  |  |  |  |
| Total cholesterol (mmol/L) | 1.12 (1.07, 1.17) | <0.001 |  | / |  |
| LDL cholesterol (mmol/L) | 1.13 (1.06, 1.19) | <0.001 |  | / |  |
| HDL cholesterol (mmol/L) | 0.76 (0.65, 0.89) | <0.001 |  | / |  |
| Triglycerides (mmol/L) | 1.07 (1.04, 1.10) | <0.001 |  | / |  |
| Waist-hip ratio above the cutoff (n=7900) | 2.00 (1.81, 2.20) | <0.001 |  | / |  |
| Hypertension | 3.02 (2.73, 3.34) | <0.001 |  | / |  |
| Diabetes | 2.98 (2.56, 3.47) | <0.001 |  | / |  |
| Metabolic syndrome | 2.16 (1.96, 2.38) | <0.001 |  | / |  |
| Baseline CD4 (cells/μl) (n=2563) |  |  |  |  |  |
| ≤200 | 1.19 (0.96, 1.48) | 0.112 |  | / |  |
| 201-350 | 1.29 (1.06, 1.56) | 0.010 |  | / |  |
| >350 | 1.00 |  |  | / |  |
| Nadir CD4<200 (cells/μl) (n=2578) | 1.12 (0.95, 1.31) | 0.182 |  | / |  |
| Years since HIV diagnosis (mean±SD) | 0.99 (0.97, 1.02) | 0.680 |  | / |  |
| a assessed by univariable logistic regression | | |  |  |  |
| b assessed by multivariable logistic regression | | | | |  |
| Abbreviation: PWH, people with HIV; BMI, body mass index; LDL, low-density lipoprotein; HDL, high-density lipoprotein; ART, antiretroviral therapy. | | | | | |
|  |

| **Table S2. Association analysis of SNPs potentially associated with the level of cIMT among different participants** | | | | | | | | |
| --- | --- | --- | --- | --- | --- | --- | --- | --- |
| **SNP** | **CHR** | **position** | **Gene** | **MAF** | **Minor allele** | **Major allele** | **β value^a^** | **adjusted P value^a^** |
| *All participants* |  |  |  |  |  |  |  |  |
| rs3732968 | 3 | 10013273 | EMC3 | 0.147 | G | A | 0.04 | 8.95E-08 |
| rs6786636 | 3 | 10045703 | EMC3 | 0.169 | C | T | 0.04 | 4.59E-07 |
| rs2302786 | 3 | 9979660 | CRELD1 | 0.199 | G | A | 0.03 | 7.21E-07 |
| *All participants under 45 years old* | | |  |  |  |  |  |  |
| rs10914347 | 1 | 31773496 | ZCCHC17 | 0.201 | T | G | 0.04 | 3.90E-08 |
| rs3766295 | 1 | 31820225 | ZCCHC17 | 0.201 | A | C | 0.04 | 5.89E-08 |
| rs77603390 | 16 | 49830172 | ZNF423 | 0.075 | A | G | 0.05 | 3.43E-07 |
| rs111815403 | 11 | 2553341 | KCNQ1 | 0.061 | A | G | 0.06 | 3.93E-07 |
| rs13194498 | 6 | 49620573 | - | 0.071 | C | T | 0.05 | 5.73E-07 |
| rs12122184 | 1 | 31858784 | LOC105378623 | 0.229 | C | T | 0.03 | 8.79E-07 |
| *All participants above 45 years old* |  |  |  |  |  |  |  |  |
| rs8058808 | 16 | 87839943 | / | 0.054 | A | G | 0.11 | 5.46E-08 |
| rs4661575 | 1 | 15414413 | KAZN | 0.088 | C | T | 0.09 | 8.03E-08 |
| rs16891400 | 6 | 80713722 | TTK | 0.054 | A | G | 0.11 | 8.74E-08 |
| rs7466234 | 9 | 8143737 | / | 0.107 | A | G | 0.08 | 2.84E-07 |
| rs62487045 | 7 | 105342787 | ATXN7L1 | 0.072 | T | C | 0.09 | 3.61E-07 |
| *HIV negative participants* | | |  |  |  |  |  |  |
| rs2302786 | 3 | 9979660 | CRELD1 | 0.199 | G | A | 0.04 | 3.65E-08 |
| rs13096737 | 3 | 10016911 | EMC3,LOC442075 | 0.073 | C | A | 0.07 | 4.73E-08 |
| rs13146599 | 4 | 92348857 | CCSER1 | 0.114 | A | G | 0.05 | 4.80E-08 |
| rs3774207 | 3 | 9985656 | CRELD1,PRRT3 | 0.200 | T | C | 0.04 | 1.33E-07 |
| rs6786636 | 3 | 10045703 | EMC3-AS1,LOC442075 | 0.170 | C | T | 0.05 | 1.40E-07 |
| rs3732968 | 3 | 10013273 | EMC3,LOC442075 | 0.149 | G | A | 0.05 | 2.43E-07 |
| rs8058808 | 16 | 87839943 | SLC7A5 | 0.052 | A | G | 0.07 | 2.58E-07 |
| rs3755783 | 3 | 10029289 | EMC3,EMC3-AS1,LOC442075 | 0.167 | G | A | 0.04 | 4.15E-07 |
| rs10856885 | 4 | 92309543 | CCSER1 | 0.130 | C | T | 0.05 | 5.00E-07 |
| *HIV-negative individuals under 45 years old* |  |  |  |  |  |  |  |  |
| rs34527568 | 3 | 14582785 | GRIP2 | 0.072 | G | T | 0.07 | 2.81E-09 |
| rs9863287 | 3 | 14582939 | GRIP2 | 0.073 | T | C | 0.07 | 7.04E-09 |
| rs9932976 | 16 | 6505665 | RBFOX1 | 0.119 | C | T | 0.05 | 4.41E-08 |
| rs9938274 | 16 | 6505208 | RBFOX1 | 0.118 | T | C | 0.05 | 7.13E-08 |
| rs118148069 | 16 | 9146749 | - | 0.06 | A | G | 0.06 | 4.50E-07 |
| rs4485261 | 14 | 38637716 | - | 0.054 | A | G | 0.07 | 4.77E-07 |
| rs10255973 | 7 | 153616857 | DPP6 | 0.215 | A | G | 0.04 | 6.57E-07 |
| rs9385488 | 6 | 129673689 | LAMA2 | 0.176 | C | T | 0.04 | 9.71E-07 |
| *HIV-negative individuals above 45 years old* |  |  |  |  |  |  |  |  |
| rs62487045 | 7 | 105342787 | ATXN7L1 | 0.072 | T | C | 0.13 | 4.53E-09 |
| rs8058808 | 16 | 87839943 | / | 0.054 | A | G | 0.14 | 2.97E-08 |
| rs4661575 | 1 | 15414413 | KAZN | 0.088 | C | T | 0.11 | 3.63E-08 |
| rs16891400 | 6 | 80713722 | TTK | 0.054 | A | G | 0.14 | 7.43E-08 |
| rs587741 | 7 | 105386461 | ATXN7L1 | 0.074 | C | T | 0.11 | 1.83E-07 |
| rs28412203 | 7 | 105384160 | ATXN7L1 | 0.073 | T | C | 0.11 | 1.88E-07 |
| rs76462900 | 7 | 19738751 | POLR1F | 0.058 | C | T | 0.13 | 3.65E-07 |
| rs1788783 | 18 | 21161134 | NPC1 | 0.070 | C | T | 0.12 | 6.08E-07 |
| rs11625012 | 14 | 51804089 | LINC00640 | 0.093 | G | T | 0.10 | 8.84E-07 |
| a assessed by linear mixed model, adjusted for age, sex, smoking status, regular alcohol use, BMI and first five principal components of PCA | | | | | | | | |

| **Table S3. Association analysis of SNPs potentially associated with the level of cIMT among different participants** | | | | | | | | |
| --- | --- | --- | --- | --- | --- | --- | --- | --- |
| **SNP** | **CHR** | **position** | **Gene** | **MAF** | **Minor allele** | **Major allele** | **β value^a^** | **adjusted P value^a^** |
| *All participants* |  |  |  |  |  |  |  |  |
| rs3732968 | 3 | 10013273 | EMC3 | 0.147 | G | A | 0.04 | 8.95E-08 |
| rs6786636 | 3 | 10045703 | EMC3 | 0.169 | C | T | 0.04 | 4.59E-07 |
| rs2302786 | 3 | 9979660 | CRELD1 | 0.199 | G | A | 0.03 | 7.21E-07 |
| *All participants under 45 years old* | | |  |  |  |  |  |  |
| rs10914347 | 1 | 31773496 | ZCCHC17 | 0.201 | T | G | 0.04 | 3.90E-08 |
| rs3766295 | 1 | 31820225 | ZCCHC17 | 0.201 | A | C | 0.04 | 5.89E-08 |
| rs77603390 | 16 | 49830172 | ZNF423 | 0.075 | A | G | 0.05 | 3.43E-07 |
| rs111815403 | 11 | 2553341 | KCNQ1 | 0.061 | A | G | 0.06 | 3.93E-07 |
| rs13194498 | 6 | 49620573 | - | 0.071 | C | T | 0.05 | 5.73E-07 |
| rs12122184 | 1 | 31858784 | LOC105378623 | 0.229 | C | T | 0.03 | 8.79E-07 |
| *All participants above 45 years old* |  |  |  |  |  |  |  |  |
| rs8058808 | 16 | 87839943 | / | 0.054 | A | G | 0.11 | 5.46E-08 |
| rs4661575 | 1 | 15414413 | KAZN | 0.088 | C | T | 0.09 | 8.03E-08 |
| rs16891400 | 6 | 80713722 | TTK | 0.054 | A | G | 0.11 | 8.74E-08 |
| rs7466234 | 9 | 8143737 | / | 0.107 | A | G | 0.08 | 2.84E-07 |
| rs62487045 | 7 | 105342787 | ATXN7L1 | 0.072 | T | C | 0.09 | 3.61E-07 |
| *HIV negative participants* | | |  |  |  |  |  |  |
| rs2302786 | 3 | 9979660 | CRELD1 | 0.199 | G | A | 0.04 | 3.65E-08 |
| rs13096737 | 3 | 10016911 | EMC3,LOC442075 | 0.073 | C | A | 0.07 | 4.73E-08 |
| rs13146599 | 4 | 92348857 | CCSER1 | 0.114 | A | G | 0.05 | 4.80E-08 |
| rs3774207 | 3 | 9985656 | CRELD1,PRRT3 | 0.200 | T | C | 0.04 | 1.33E-07 |
| rs6786636 | 3 | 10045703 | EMC3-AS1,LOC442075 | 0.170 | C | T | 0.05 | 1.40E-07 |
| rs3732968 | 3 | 10013273 | EMC3,LOC442075 | 0.149 | G | A | 0.05 | 2.43E-07 |
| rs8058808 | 16 | 87839943 | SLC7A5 | 0.052 | A | G | 0.07 | 2.58E-07 |
| rs3755783 | 3 | 10029289 | EMC3,EMC3-AS1,LOC442075 | 0.167 | G | A | 0.04 | 4.15E-07 |
| rs10856885 | 4 | 92309543 | CCSER1 | 0.130 | C | T | 0.05 | 5.00E-07 |
| *HIV-negative individuals under 45 years old* |  |  |  |  |  |  |  |  |
| rs34527568 | 3 | 14582785 | GRIP2 | 0.072 | G | T | 0.07 | 2.81E-09 |
| rs9863287 | 3 | 14582939 | GRIP2 | 0.073 | T | C | 0.07 | 7.04E-09 |
| rs9932976 | 16 | 6505665 | RBFOX1 | 0.119 | C | T | 0.05 | 4.41E-08 |
| rs9938274 | 16 | 6505208 | RBFOX1 | 0.118 | T | C | 0.05 | 7.13E-08 |
| rs118148069 | 16 | 9146749 | - | 0.06 | A | G | 0.06 | 4.50E-07 |
| rs4485261 | 14 | 38637716 | - | 0.054 | A | G | 0.07 | 4.77E-07 |
| rs10255973 | 7 | 153616857 | DPP6 | 0.215 | A | G | 0.04 | 6.57E-07 |
| rs9385488 | 6 | 129673689 | LAMA2 | 0.176 | C | T | 0.04 | 9.71E-07 |
| *HIV-negative individuals above 45 years old* |  |  |  |  |  |  |  |  |
| rs62487045 | 7 | 105342787 | ATXN7L1 | 0.072 | T | C | 0.13 | 4.53E-09 |
| rs8058808 | 16 | 87839943 | / | 0.054 | A | G | 0.14 | 2.97E-08 |
| rs4661575 | 1 | 15414413 | KAZN | 0.088 | C | T | 0.11 | 3.63E-08 |
| rs16891400 | 6 | 80713722 | TTK | 0.054 | A | G | 0.14 | 7.43E-08 |
| rs587741 | 7 | 105386461 | ATXN7L1 | 0.074 | C | T | 0.11 | 1.83E-07 |
| rs28412203 | 7 | 105384160 | ATXN7L1 | 0.073 | T | C | 0.11 | 1.88E-07 |
| rs76462900 | 7 | 19738751 | POLR1F | 0.058 | C | T | 0.13 | 3.65E-07 |
| rs1788783 | 18 | 21161134 | NPC1 | 0.070 | C | T | 0.12 | 6.08E-07 |
| rs11625012 | 14 | 51804089 | LINC00640 | 0.093 | G | T | 0.10 | 8.84E-07 |
| a assessed by linear mixed model, adjusted for age, sex, smoking status, regular alcohol use, BMI and first five principal components of PCA | | | | | | | | |

| **Table S4. The association of unweighted genetic risk score with cIMT level or subclinical atherosclerosis among different groups** | | | | | | | | | | | |
| --- | --- | --- | --- | --- | --- | --- | --- | --- | --- | --- | --- |
|  | cIMT level | | | | |  | Subclinical atherosclerosis | | | | |
|  | Model 1 | |  | Model 2 | |  | Model 3 | |  | Model 4 | |
|  | β (SE) | P value |  | β (SE) | P value |  | OR (95%CI) | P value |  | OR (95%CI) | P value |
| All participants | 0.01 (0.01) | 0.001 |  | 0.01 (0.01) | 2.26E-04 |  | 1.01 (0.97, 1.04) | 0.759 |  | 1.02 (0.97, 1.06) | 0.482 |
| under 45 years old | 0.02 (0.01) | 2.82E-15 |  | 0.02 (0.01) | 3.74E-16 |  | 1.07 (1.00, 1.14) | 0.042 |  | 1.09 (1.02, 1.16) | 0.013 |
| above 45 years old | -0.01 (0.01) | 0.215 |  | -0.01 (0.01) | 0.388 |  | 0.97 (0.92, 1.02) | 0.166 |  | 0.97 (0.92, 1.03) | 0.341 |
| PWH | 0.03 (0.01) | 8.93E-11 |  | 0.03 (0.01) | 3.93E-13 |  | 1.03 (0.97, 1.09) | 0.425 |  | 1.04 (0.97, 1.11) | 0.248 |
| under 45 years old | 0.06 (0.01) | <2.00E-16 |  | 0.06 (0.01) | <2.00E-16 |  | 1.12 (1.02, 1.22) | 0.017 |  | 1.14 (1.04, 1.25) | 0.006 |
| above 45 years old | -0.01 (0.01) | 0.645 |  | 0.01 (0.01) | 0.844 |  | 0.92 (0.84, 1.01) | 0.084 |  | 0.95 (0.87, 1.05) | 0.327 |
| HIV-negative individuals | -0.01 (0.01) | 0.876 |  | -0.01 (0.01) | 0.799 |  | 1.00 (0.96, 1.05) | 0.975 |  | 1.00 (0.95, 1.06) | 0.974 |
| under 45 years old | 0.01 (0.01) | 0.074 |  | 0.01 (0.01) | 0.123 |  | 1.05 (0.96, 1.14) | 0.341 |  | 1.03 (0.94, 1.14) | 0.536 |
| above 45 years old | -0.01 (0.01) | 0.255 |  | -0.01 (0.01) | 0.221 |  | 0.99 (0.93, 1.05) | 0.639 |  | 0.98 (0.92, 1.05) | 0.600 |
| Model 1: univariable analysis, assessed by generalized linear regression model | | | | | | | | | | | |
| Model 2: adjusted for age, sex, regular alcohol use, current smoking status, BMI and HIV serostatus; assessed by generalized linear regression model | | | | | | | | | | | |
| Model 3: univariable analysis, assessed by logistic regression model | | | | | | | | | | | |
| Model 4: adjusted for age, sex, regular alcohol use, current smoking status, BMI and HIV serostatus; assessed by logistic regression model | | | | | | | | | | | |

| **Table S5. The association of weighted genetic risk score with cIMT level or subclinical atherosclerosis among different groups** | | | | | | | | | | | |
| --- | --- | --- | --- | --- | --- | --- | --- | --- | --- | --- | --- |
|  | cIMT level | | | | |  | Subclinical atherosclerosis | | | | |
|  | Model 1 | |  | Model 2 | |  | Model 3 | |  | Model 4 | |
|  | β (SE) | P value |  | β (SE) | P value |  | OR (95%CI) | P value |  | OR (95%CI) | P value |
| All participants | 0.09 (0.03) | 2.00E-03 |  | 0.09 (0.03) | 7.94E-04 |  | 1.06 (0.76, 1.47) | 0.746 |  | 1.10 (0.75, 1.62) | 0.630 |
| under 45 years old | 0.18 (0.03) | 4.99E-12 |  | 0.19 (0.03) | 1.27E-12 |  | 1.63 (0.91, 2.94) | 0.102 |  | 1.88 (1.02, 3.46) | **0.043** |
| above 45 years old | -0.04 (0.05) | 0.424 |  | -0.03 (0.05) | 0.553 |  | 0.77 (0.49, 1.22) | 0.261 |  | 0.80 (0.49, 1.31) | 0.382 |
| PWH | 0.30 (0.05) | **5.14E-10** |  | 0.30 (0.04) | **6.90E-12** |  | 1.21 (0.69, 2.14) | 0.511 |  | 1.33 (0.70, 2.51) | 0.385 |
| under 45 years old | 0.50 (0.05) | **<2.00E-16** |  | 0.51 (0.05) | **<2.00E-16** |  | 2.30 (0.99, 5.33) | 0.053 |  | 2.72 (1.15, 6.42) | **0.023** |
| above 45 years old | -0.02 (0.08) | 0.760 |  | 0.02 (0.08) | 0.817 |  | 0.52 (0.22, 1.21) | 0.128 |  | 0.66 (0.27, 1.63) | 0.369 |
| HIV-negative individuals | -0.01 (0.04) | 0.956 |  | -0.01 (0.03) | 0.770 |  | 1.04 (0.69, 1.57) | 0.853 |  | 0.98 (0.60, 1.61) | 0.948 |
| under 45 years old | 0.04 (0.03) | 0.229 |  | 0.03 (0.03) | 0.382 |  | 1.47 (0.65, 3.37) | 0.357 |  | 1.24 (0.52, 2.98) | 0.629 |
| above 45 years old | -0.04 (0.06) | 0.474 |  | -0.05 (0.06) | 0.360 |  | 0.92 (0.54, 1.58) | 0.764 |  | 0.86 (0.48, 1.55) | 0.619 |
| Model 1: univariable analysis, assessed by generalized linear regression model | | | | | | | | | | | |
| Model 2: adjusted for age, sex, regular alcohol use, current smoking status, BMI and HIV serostatus; assessed by generalized linear regression model | | | | | | | | | | | |
| Model 3: univariable analysis, assessed by logistic regression model | | | | | | | | | | | |
| Model 4: adjusted for age, sex, regular alcohol use, current smoking status, BMI and HIV serostatus; assessed by logistic regression model | | | | | | | | | | | |

| **Table S6. Association P-values for all lead SNPs for cIMT among PWH under 45 years old.** | | | | | | | | | | | | | |
| --- | --- | --- | --- | --- | --- | --- | --- | --- | --- | --- | --- | --- | --- |
| **GenomicLocus** | **uniqID** | **rsID** | **chr** | **pos** | **p** | **start** | **end** | **nSNPs** | **nGWASSNPs** | **nIndSigSNPs** | **IndSigSNPs** | **nLeadSNPs** | **LeadSNPs** |
| 1 | 3:37536056:C:T | rs2507941 | 3 | 37536056 | 3.85E-07 | 37532209 | 37595373 | 32 | 2 | 1 | rs2507941 | 1 | rs2507941 |
| 2 | 3:96683649:A:G | rs6762348 | 3 | 96683649 | 4.42E-07 | 96537223 | 96731529 | 87 | 3 | 1 | rs6762348 | 1 | rs6762348 |
| 3 | 4:171482271:C:T | rs148420952 | 4 | 171482271 | 3.94E-07 | 171465318 | 171501997 | 3 | 1 | 1 | rs148420952 | 1 | rs148420952 |
| 4 | 5:108563812:A:G | rs35812497 | 5 | 108563812 | 3.27E-07 | 108563812 | 108632237 | 115 | 3 | 1 | rs35812497 | 1 | rs35812497 |
| 5 | 11:2553341:A:G | rs111815403 | 11 | 2553341 | 1.50E-07 | 2553341 | 2553341 | 1 | 1 | 1 | rs111815403 | 1 | rs111815403 |
| 6 | 12:80815650:C:T | rs77741796 | 12 | 80815650 | 2.20E-09 | 80678719 | 80846133 | 5 | 1 | 1 | rs77741796 | 1 | rs77741796 |
| 7 | 12:127782099:A:C | rs10847321 | 12 | 127782099 | 8.91E-07 | 127763567 | 127788056 | 21 | 1 | 1 | rs10847321 | 1 | rs10847321 |
| Note: Genomic risk loci were defined by independent lead SNPs and maximum distance between their LD block. Genomic locus = locus ID; Unique SNP ID = ID variable formatted as 'chromosome:base pair position:alleles in alphabetic order'; rsID = rs number of the SNP; chr = Chromosome; BP = base pair position; P-value = P-value of top lead SNP for cIMT (the threhold of 1×10^-6^); Start/End = start and end position of the genomic locus; No. of SNPs = number of unique candidate SNPs in the genomic locus, including non-GWAS-tagged SNPs (which are extracted from 1000G); No. of lead SNPs = number of lead SNPs in the genomic locus (r^2^ < 0.1); Lead SNPs = rsID's of lead SNPs; No. of IndSigSNPs = number of the independent significant SNPs in the genomic locus (r^2^ < 0.6); IndSigSNPs = rsID's of independent significant SNPs; | | | | | | | | | | | |  |  |
|  |  |
|  |  |
|  |  |
|  |  |
|  |  |
|  |  |

| **Table S7. All genes mapped in SNP-based (FUMA) for cIMT among PWH under 45 years old** | | | | | | | | | | | | | | | | | | | | | | | | | | |
| --- | --- | --- | --- | --- | --- | --- | --- | --- | --- | --- | --- | --- | --- | --- | --- | --- | --- | --- | --- | --- | --- | --- | --- | --- | --- | --- |
| **ensg** | **symbol** | **chr** | **start** | **end** | **strand** | **type** | **entrezID** | **HUGO** | **pLI** | **ncRVIS** | **posMapSNPs** | **posMapMaxCADD** | **eqtlMapSNPs** | **eqtlMapminP** | **eqtlMapminQ** | **eqtlMapts** | **eqtlDirection** | **ciMap** | **ciMapts** | **minGwasP** | **IndSigSNPs** | **GenomicLocus** | **positional mapping** | **eQTL mapping** | **CI mapping** | **No. of ways gene is mapped** |
| ENSG00000163673 | DCLK3 | 3 | 36753913 | 36781352 | -1 | protein_coding | 85443 | DCLK3 | 0.439315818 | 1.518414455 | 0 | 0 | 2 | 1.55E-05 | 8.15E-11 | GTEx/v8/Nerve_Tibial | - | No | NA | 1.18E-05 | rs2507941 | 1 | FALSE | TRUE | FALSE | 1 |
| ENSG00000178567 | EPM2AIP1 | 3 | 37027357 | 37034795 | -1 | protein_coding | 9852 | EPM2AIP1 | 0.164415268 | 0.811082225 | 0 | 0 | 1 | 1.37E-05 | 0.038271166 | eQTLGen_cis_eQTLs | NA | No | NA | NA | rs2507941 | 1 | FALSE | TRUE | FALSE | 1 |
| ENSG00000144668 | ITGA9 | 3 | 37493606 | 37865005 | 1 | protein_coding | 3680 | ITGA9 | 0.096191748 | -0.021472053 | 32 | 13.94 | 29 | 5.19E-11 | 0 | eQTLGen_cis_eQTLs:GTEx/v8/Brain_Cerebellar_Hemisphere:GTEx/v8/Brain_Cerebellum:GTEx/v7/Brain_Cerebellar_Hemisphere:GTEx/v7/Brain_Cerebellum | + | Yes | Adult_Cortex:Fetal_Cortex:IMR90:Mesendoderm:hESC | 3.85E-07 | rs2507941 | 1 | TRUE | TRUE | TRUE | 3 |
| ENSG00000187091 | PLCD1 | 3 | 38048987 | 38071253 | -1 | protein_coding | 5333 | PLCD1 | 2.55E-09 | -0.675212094 | 0 | 0 | 2 | 1.44E-06 | 0.004360022 | eQTLGen_cis_eQTLs | NA | No | NA | NA | rs2507941 | 1 | FALSE | TRUE | FALSE | 1 |
| ENSG00000114739 | ACVR2B | 3 | 38495342 | 38534633 | 1 | protein_coding | 93 | ACVR2B | 0.993110896 | NA | 0 | 0 | 2 | 6.60E-06 | 0.000134833 | GTEx/v7/Whole_Blood:GTEx/v6/Whole_Blood | NA | No | NA | NA | rs2507941 | 1 | FALSE | TRUE | FALSE | 1 |
| ENSG00000183873 | SCN5A | 3 | 38589548 | 38691164 | -1 | protein_coding | 6331 | SCN5A | 0.995747527 | -0.493253335 | 0 | 0 | 0 | NA | NA | NA | NA | Yes | IMR90 | NA | rs2507941 | 1 | FALSE | FALSE | TRUE | 1 |
| ENSG00000269028 | MTRNR2L12 | 3 | 96335981 | 96337000 | -1 | protein_coding | 100463498 | MTRNR2L12 | NA | NA | 0 | 0 | 8 | 0.000123071 | 0.030362 | GTEx/v8/Breast_Mammary_Tissue | NA | No | NA | NA | rs6762348 | 2 | FALSE | TRUE | FALSE | 1 |
| ENSG00000080224 | EPHA6 | 3 | 96533425 | 97471304 | 1 | protein_coding | 285220 | EPHA6 | 0.954513147 | -0.193013583 | 87 | 16.2 | 0 | NA | NA | NA | NA | No | NA | 4.42E-07 | rs6762348 | 2 | TRUE | FALSE | FALSE | 1 |
| ENSG00000151422 | FER | 5 | 108083523 | 108532542 | 1 | protein_coding | 2241 | FER | 0.968420467 | 1.734071518 | 0 | 0 | 1 | 4.05E-06 | 0.025282482 | GTEx/v6/Cells_EBV-transformed_lymphocytes | NA | No | NA | NA | rs35812497 | 4 | FALSE | TRUE | FALSE | 1 |
| ENSG00000198961 | PJA2 | 5 | 108670410 | 108745695 | -1 | protein_coding | 9867 | PJA2 | 0.466603862 | -0.019617374 | 0 | 0 | 6 | 7.02E-06 | 0.020039469 | eQTLGen_cis_eQTLs | NA | No | NA | NA | rs35812497 | 4 | FALSE | TRUE | FALSE | 1 |
| ENSG00000183734 | ASCL2 | 11 | 2289725 | 2292182 | -1 | protein_coding | 430 | ASCL2 | 0.38890041 | NA | 0 | 0 | 0 | NA | NA | NA | NA | Yes | IMR90:Mesenchymal_Stem_Cell | 1.50E-07 | rs111815403 | 5 | FALSE | FALSE | TRUE | 1 |
| ENSG00000053918 | KCNQ1 | 11 | 2465914 | 2870339 | 1 | protein_coding | 3784 | KCNQ1 | 2.45E-05 | 1.153237047 | 1 | 0.043 | 0 | NA | NA | NA | NA | No | NA | 1.50E-07 | rs111815403 | 5 | TRUE | FALSE | FALSE | 1 |
| ENSG00000005801 | ZNF195 | 11 | 3360491 | 3400448 | -1 | protein_coding | 7748 | ZNF195 | 0.731668925 | 0.495026993 | 0 | 0 | 0 | NA | NA | NA | NA | Yes | IMR90:Mesenchymal_Stem_Cell | 1.50E-07 | rs111815403 | 5 | FALSE | FALSE | TRUE | 1 |
| ENSG00000165899 | OTOGL | 12 | 80603233 | 80772870 | 1 | protein_coding | 283310 | OTOGL | 3.73E-31 | NA | 2 | 2.164 | 0 | NA | NA | NA | NA | No | NA | NA | rs77741796 | 6 | TRUE | FALSE | FALSE | 1 |
| ENSG00000139304 | PTPRQ | 12 | 80799774 | 81072802 | 1 | protein_coding | 374462 | PTPRQ | NA | NA | 3 | 4.937 | 0 | NA | NA | NA | NA | No | NA | 2.20E-09 | rs77741796 | 6 | TRUE | FALSE | FALSE | 1 |
| Note: SNPs were identified by a genome-wide association (P<1×10-6) among PWH under 45 years old, mapped to genes using positional mapping, eQTL maping, and chromatin interaction strategies.Genomic locus = index of genomic locus; Symbol = gene symbol; Entrez ID = Entrez gene ID; Chr = chromosome; Start/End = start and end position of the genomic locus; Strand =coding strand; pLI = pLI score from ExAC database, The probability of being loss-of-function intolerant. The higher the score is, the more intolerant to loss-of-function mutations the gene is; ncRVIS = non-coding residual variation intolerance score. The higher the score is, the more intolerant to non-coding variation the gene is; posMapSNPs = number of SNPs mapped to gene based on positional mapping (after functional filtering if parameters are given); posMapMaxCADD = maximum CADD score of mapped SNPs by positional mapping; eqtlMapSNPs = number of SNPs mapped to the gene based on eQTL mapping; eqtlMapminP = minimum eQTL P-value of mapped SNPs; eqtlMapminQ = minimum eQTL FDR of mapped SNPs; eqtlMapts = tissue types of mapped eQTL SNPs; eqtlDirection = consecutive direction of mapped eQTL SNPs after aligning risk increasing alleles in GWAS and tested alleles in eQTL data source; ciMap = "Yes" if the gene is mapped by chromatin interaction mapping, "No" otherwise; ciMapts = tissue/cell types of mapped chromatin interactions; minGwasP = The minimum P-value of mapped SNPs; IndSigSNPs = rsID of the all independent significant SNPs of mapped SNPs; NA values in any column indicate that the given gene was maped not in SNP-based analyses (FUMA). | | | | | | | | | | | | | | | | | | | | | | | | | | |
|  |
|  |
|  |
|  |
|  |
|  |
|  |
|  |

| **Table S8. Association P-values for all lead SNPs for cIMT among HIV-negative controls under 45 years old.** | | | | | | | | | | | | | |
| --- | --- | --- | --- | --- | --- | --- | --- | --- | --- | --- | --- | --- | --- |
| **GenomicLocus** | **uniqID** | **rsID** | **chr** | **pos** | **p** | **start** | **end** | **nSNPs** | **nGWASSNPs** | **nIndSigSNPs** | **IndSigSNPs** | **nLeadSNPs** | **LeadSNPs** |
| 1 | 3:14582785:G:T | rs34527568 | 3 | 14582785 | 2.81E-09 | 14582785 | 14582986 | 3 | 2 | 1 | rs34527568 | 1 | rs34527568 |
| 2 | 6:129673689:C:T | rs9385488 | 6 | 129673689 | 9.71E-07 | 129628090 | 129704589 | 24 | 3 | 1 | rs9385488 | 1 | rs9385488 |
| 3 | 7:153616857:A:G | rs10255973 | 7 | 153616857 | 6.58E-07 | 153616857 | 153627177 | 5 | 1 | 1 | rs10255973 | 1 | rs10255973 |
| 4 | 14:38637716:A:G | rs4485261 | 14 | 38637716 | 4.78E-07 | 38637716 | 38658493 | 5 | 1 | 1 | rs4485261 | 1 | rs4485261 |
| 5 | 16:6505665:C:T | rs9932976 | 16 | 6505665 | 4.41E-08 | 6499391 | 6515493 | 13 | 2 | 1 | rs9932976 | 1 | rs9932976 |
| 6 | 16:9146749:A:G | rs118148069 | 16 | 9146749 | 4.50E-07 | 9142725 | 9164733 | 4 | 1 | 1 | rs118148069 | 1 | rs118148069 |
| Note: Genomic risk loci were defined by independent lead SNPs and maximum distance between their LD block. Genomic locus = locus ID; Unique SNP ID = ID variable formatted as 'chromosome:base pair position:alleles in alphabetic order'; rsID = rs number of the SNP; chr = Chromosome; BP = base pair position; P-value = P-value of top lead SNP for cIMT (the threhold of 1×10-6); Start/End = start and end position of the genomic locus; No. of SNPs = number of unique candidate SNPs in the genomic locus, including non-GWAS-tagged SNPs (which are extracted from 1000G); No. of lead SNPs = number of lead SNPs in the genomic locus (r2 < 0.1); Lead SNPs = rsID's of lead SNPs; No. of IndSigSNPs = number of the independent significant SNPs in the genomic locus (r2 < 0.6); IndSigSNPs = rsID's of independent significant SNPs; | | | | | | | | | | | | | |
|  |
|  |
|  |
|  |
|  |
|  |

| **Table S9. All genes mapped in SNP-based (FUMA) for cIMT among HIV-negative controls under 45 years old** | | | | | | | | | | | | | | | | | | | | | | |
| --- | --- | --- | --- | --- | --- | --- | --- | --- | --- | --- | --- | --- | --- | --- | --- | --- | --- | --- | --- | --- | --- | --- |
| **ensg** | **symbol** | **chr** | **start** | **end** | **strand** | **type** | **entrezID** | **HUGO** | **pLI** | **ncRVIS** | **posMapSNPs** | **posMapMaxCADD** | **eqtlMapSNPs** | **eqtlMapminP** | **eqtlMapminQ** | **eqtlMapts** | **eqtlDirection** | **ciMap** | **ciMapts** | **minGwasP** | **IndSigSNPs** | **GenomicLocus** |
| ENSG00000154781 | CCDC174 | 3 | 14693271 | 14714166 | 1 | protein_coding | 51244 | CCDC174 | 2.95E-05 | 0.321126987 | 0 | 0 | 2 | 2.35E-08 | 0.000114745 | eQTLGen_cis_eQTLs | + | No | NA | 7.04E-09 | rs34527568 | 1 |
| ENSG00000093144 | ECHDC1 | 6 | 127609855 | 127664754 | -1 | protein_coding | 55862 | ECHDC1 | 6.03E-05 | 0.227821385 | 0 | 0 | 0 | NA | NA | NA | NA | Yes | Left_Ventricle:IMR90:Mesenchymal_Stem_Cell:Mesendoderm:hESC | 2.10E-05 | rs9385488 | 2 |
| ENSG00000214338 | SOGA3 | 6 | 127759551 | 127840500 | -1 | protein_coding | 387104 | SOGA3 | NA | 0.147040609 | 0 | 0 | 0 | NA | NA | NA | NA | Yes | Liver:IMR90:Mesenchymal_Stem_Cell:Mesendoderm:Trophoblast-like_Cell:hESC | 2.10E-05 | rs9385488 | 2 |
| ENSG00000255330 | SOGA3 | 6 | 127759551 | 127840146 | -1 | protein_coding | 387104 | SOGA3 | NA | 0.147040609 | 0 | 0 | 0 | NA | NA | NA | NA | Yes | Liver:IMR90:Mesenchymal_Stem_Cell:Mesendoderm:Trophoblast-like_Cell:hESC | 2.10E-05 | rs9385488 | 2 |
| ENSG00000184530 | C6orf58 | 6 | 127840600 | 127912962 | 1 | protein_coding | 352999 | C6orf58 | 1.61E-10 | 0.187402208 | 0 | 0 | 0 | NA | NA | NA | NA | Yes | Liver:IMR90:Mesenchymal_Stem_Cell:Mesendoderm:Trophoblast-like_Cell:hESC | 2.10E-05 | rs9385488 | 2 |
| ENSG00000152894 | PTPRK | 6 | 128289924 | 128841870 | -1 | protein_coding | 5796 | PTPRK | 0.980388546 | -0.479546584 | 0 | 0 | 0 | NA | NA | NA | NA | Yes | Fetal_Cortex:IMR90:Mesenchymal_Stem_Cell:Mesendoderm:hESC | 2.10E-05 | rs9385488 | 2 |
| ENSG00000196569 | LAMA2 | 6 | 129204342 | 129837714 | 1 | protein_coding | 3908 | LAMA2 | 1.18E-24 | 0.02365523 | 24 | 22.4 | 13 | 7.26E-10 | 6.59E-06 | eQTLGen_cis_eQTLs:BIOSQTL/BIOS_eQTL_geneLevel | + | Yes | Left_Ventricle:IMR90:Mesenchymal_Stem_Cell:Mesendoderm:Neural_Progenitor_Cell:Trophoblast-like_Cell:hESC | 9.71E-07 | rs9385488 | 2 |
| ENSG00000203756 | TMEM244 | 6 | 130152389 | 130182692 | -1 | protein_coding | 253582 | TMEM244 | 0.037087536 | -0.246645988 | 0 | 0 | 0 | NA | NA | NA | NA | Yes | Mesenchymal_Stem_Cell | 2.10E-05 | rs9385488 | 2 |
| ENSG00000130226 | DPP6 | 7 | 153584182 | 154685995 | 1 | protein_coding | 1804 | DPP6 | 0.967691107 | NA | 5 | 1.001 | 2 | 3.45E-06 | 5.38E-07 | GTEx/v8/Nerve_Tibial | + | No | NA | 6.58E-07 | rs10255973 | 3 |
| ENSG00000078328 | RBFOX1 | 16 | 6069095 | 7763340 | 1 | protein_coding | 54715 | RBFOX1 | 0.935961018 | 1.09605486 | 13 | 6.253 | 0 | NA | NA | NA | NA | No | NA | 4.41E-08 | rs9932976 | 5 |
| ENSG00000153048 | CARHSP1 | 16 | 8946799 | 8962866 | -1 | protein_coding | 23589 | CARHSP1 | NA | 1.977761735 | 0 | 0 | 1 | 3.14E-07 | 0.001017244 | eQTLGen_cis_eQTLs | NA | No | NA | NA | rs118148069 | 6 |
| Note: SNPs were identified by a genome-wide association (P<1×10-6) among HIV-negative controls under 45 years old, mapped to genes using positional mapping, eQTL maping, and chromatin interaction strategies.Genomic locus = index of genomic locus; Symbol = gene symbol; Entrez ID = Entrez gene ID; Chr = chromosome; Start/End = start and end position of the genomic locus; Strand =coding strand; pLI = pLI score from ExAC database, The probability of being loss-of-function intolerant. The higher the score is, the more intolerant to loss-of-function mutations the gene is; ncRVIS = non-coding residual variation intolerance score. The higher the score is, the more intolerant to non-coding variation the gene is; posMapSNPs = number of SNPs mapped to gene based on positional mapping (after functional filtering if parameters are given); posMapMaxCADD = maximum CADD score of mapped SNPs by positional mapping; eqtlMapSNPs = number of SNPs mapped to the gene based on eQTL mapping; eqtlMapminP = minimum eQTL P-value of mapped SNPs; eqtlMapminQ = minimum eQTL FDR of mapped SNPs; eqtlMapts = tissue types of mapped eQTL SNPs; eqtlDirection = consecutive direction of mapped eQTL SNPs after aligning risk increasing alleles in GWAS and tested alleles in eQTL data source; ciMap = "Yes" if the gene is mapped by chromatin interaction mapping, "No" otherwise; ciMapts = tissue/cell types of mapped chromatin interactions; minGwasP = The minimum P-value of mapped SNPs; IndSigSNPs = rsID of the all independent significant SNPs of mapped SNPs; NA values in any column indicate that the given gene was maped not in SNP-based analyses (FUMA). | | | | | | | | | | | | | | | | | | | | | | |
|  |
|  |
|  |
|  |
|  |
|  |
|  |
|  |

| **Table S10. eQTL linking GWAS risk SNPs of cIMT to mapped genes.** | | | | | | | | | | | | | |
| --- | --- | --- | --- | --- | --- | --- | --- | --- | --- | --- | --- | --- | --- |
| **uniqID** | **db** | **tissue** | **gene** | **testedAllele** | **p** | **signed_stats** | **FDR** | **RiskIncAllele** | **alignedDirection** | **chr** | **pos** | **symbol** | **eqtlMapFilt** |
| 3:37550382:A:G | eQTLGen | eQTLGen_cis_eQTLs | ENSG00000144668 | A | 1.46E-09 | -6.0484 | 1.31E-05 | NA | NA | 3 | 37550382 | ITGA9 | 1 |
| 3:37550382:A:G | eQTLGen | eQTLGen_cis_eQTLs | ENSG00000178567 | A | 1.37E-05 | 4.3491 | 0.038271166 | NA | NA | 3 | 37550382 | EPM2AIP1 | 1 |
| 3:37550382:A:G | eQTLGen | eQTLGen_cis_eQTLs | ENSG00000187091 | A | 5.66E-06 | 4.5388 | 0.016161919 | NA | NA | 3 | 37550382 | PLCD1 | 1 |
| 3:37558775:C:T | eQTLGen | eQTLGen_cis_eQTLs | ENSG00000144668 | C | 5.19E-11 | -6.5654 | 0 | NA | NA | 3 | 37558775 | ITGA9 | 1 |
| 3:37571865:A:G | eQTLGen | eQTLGen_cis_eQTLs | ENSG00000144668 | G | 8.46E-09 | -5.7589 | 5.80E-05 | NA | NA | 3 | 37571865 | ITGA9 | 1 |
| 3:37571865:A:G | eQTLGen | eQTLGen_cis_eQTLs | ENSG00000187091 | G | 1.44E-06 | 4.8192 | 0.004360022 | NA | NA | 3 | 37571865 | PLCD1 | 1 |
| 5:108569849:C:G | eQTLGen | eQTLGen_cis_eQTLs | ENSG00000198961 | G | 7.02E-06 | 4.493 | 0.020039469 | NA | NA | 5 | 108569849 | PJA2 | 1 |
| 5:108572649:A:G | eQTLGen | eQTLGen_cis_eQTLs | ENSG00000198961 | G | 1.02E-05 | 4.4135 | 0.028632606 | NA | NA | 5 | 108572649 | PJA2 | 1 |
| 5:108574769:C:T | eQTLGen | eQTLGen_cis_eQTLs | ENSG00000198961 | T | 9.62E-06 | 4.4257 | 0.027262989 | NA | NA | 5 | 108574769 | PJA2 | 1 |
| 5:108577416:C:T | eQTLGen | eQTLGen_cis_eQTLs | ENSG00000198961 | T | 1.78E-05 | 4.2915 | 0.04869258 | NA | NA | 5 | 108577416 | PJA2 | 1 |
| 5:108581219:C:T | eQTLGen | eQTLGen_cis_eQTLs | ENSG00000198961 | C | 1.34E-05 | 4.3531 | 0.037514064 | NA | NA | 5 | 108581219 | PJA2 | 1 |
| 5:108581474:C:T | eQTLGen | eQTLGen_cis_eQTLs | ENSG00000198961 | C | 1.20E-05 | 4.3775 | 0.03380975 | NA | NA | 5 | 108581474 | PJA2 | 1 |
| 3:37532209:A:AT | GTEx/v8 | Brain_Cerebellar_Hemisphere | ENSG00000144668 | A | 3.82E-06 | 0.842025 | 1.96E-09 | NA | NA | 3 | 37532209 | ITGA9 | 1 |
| 3:37536056:C:T | GTEx/v8 | Brain_Cerebellar_Hemisphere | ENSG00000144668 | T | 4.39E-06 | 0.895653 | 1.96E-09 | T | + | 3 | 37536056 | ITGA9 | 1 |
| 3:37537638:A:G | GTEx/v8 | Brain_Cerebellar_Hemisphere | ENSG00000144668 | A | 4.39E-06 | 0.895653 | 1.96E-09 | NA | NA | 3 | 37537638 | ITGA9 | 1 |
| 3:37542234:A:G | GTEx/v8 | Brain_Cerebellar_Hemisphere | ENSG00000144668 | A | 4.39E-06 | 0.895653 | 1.96E-09 | NA | NA | 3 | 37542234 | ITGA9 | 1 |
| 3:37548780:C:T | GTEx/v8 | Brain_Cerebellar_Hemisphere | ENSG00000144668 | C | 5.53E-06 | 0.870868 | 1.96E-09 | NA | NA | 3 | 37548780 | ITGA9 | 1 |
| 3:37549402:C:T | GTEx/v8 | Brain_Cerebellar_Hemisphere | ENSG00000144668 | T | 5.53E-06 | 0.870868 | 1.96E-09 | NA | NA | 3 | 37549402 | ITGA9 | 1 |
| 3:37549983:A:G | GTEx/v8 | Brain_Cerebellar_Hemisphere | ENSG00000144668 | A | 5.53E-06 | 0.870868 | 1.96E-09 | NA | NA | 3 | 37549983 | ITGA9 | 1 |
| 3:37550382:A:G | GTEx/v8 | Brain_Cerebellar_Hemisphere | ENSG00000144668 | A | 4.43E-06 | 0.60157 | 1.96E-09 | NA | NA | 3 | 37550382 | ITGA9 | 1 |
| 3:37551323:A:G | GTEx/v8 | Brain_Cerebellar_Hemisphere | ENSG00000144668 | G | 5.53E-06 | 0.870868 | 1.96E-09 | NA | NA | 3 | 37551323 | ITGA9 | 1 |
| 3:37558775:C:T | GTEx/v8 | Brain_Cerebellar_Hemisphere | ENSG00000144668 | C | 3.62E-06 | 0.640085 | 1.96E-09 | NA | NA | 3 | 37558775 | ITGA9 | 1 |
| 3:37559481:C:T | GTEx/v8 | Brain_Cerebellar_Hemisphere | ENSG00000144668 | C | 5.53E-06 | 0.870868 | 1.96E-09 | NA | NA | 3 | 37559481 | ITGA9 | 1 |
| 3:37560114:A:T | GTEx/v8 | Brain_Cerebellar_Hemisphere | ENSG00000144668 | T | 5.53E-06 | 0.870868 | 1.96E-09 | NA | NA | 3 | 37560114 | ITGA9 | 1 |
| 3:37560708:A:G | GTEx/v8 | Brain_Cerebellar_Hemisphere | ENSG00000144668 | G | 5.53E-06 | 0.870868 | 1.96E-09 | NA | NA | 3 | 37560708 | ITGA9 | 1 |
| 3:37560965:A:C | GTEx/v8 | Brain_Cerebellar_Hemisphere | ENSG00000144668 | A | 5.53E-06 | 0.870868 | 1.96E-09 | NA | NA | 3 | 37560965 | ITGA9 | 1 |
| 3:37564853:C:T | GTEx/v8 | Brain_Cerebellar_Hemisphere | ENSG00000144668 | T | 5.53E-06 | 0.870868 | 1.96E-09 | NA | NA | 3 | 37564853 | ITGA9 | 1 |
| 3:37567033:A:G | GTEx/v8 | Brain_Cerebellar_Hemisphere | ENSG00000144668 | G | 5.53E-06 | 0.870868 | 1.96E-09 | NA | NA | 3 | 37567033 | ITGA9 | 1 |
| 3:37567896:A:G | GTEx/v8 | Brain_Cerebellar_Hemisphere | ENSG00000144668 | G | 3.58E-06 | 0.768343 | 1.96E-09 | NA | NA | 3 | 37567896 | ITGA9 | 1 |
| 3:37568727:A:G | GTEx/v8 | Brain_Cerebellar_Hemisphere | ENSG00000144668 | G | 5.53E-06 | 0.870868 | 1.96E-09 | NA | NA | 3 | 37568727 | ITGA9 | 1 |
| 3:37568777:A:G | GTEx/v8 | Brain_Cerebellar_Hemisphere | ENSG00000144668 | A | 5.53E-06 | 0.870868 | 1.96E-09 | NA | NA | 3 | 37568777 | ITGA9 | 1 |
| 3:37571337:A:G | GTEx/v8 | Brain_Cerebellar_Hemisphere | ENSG00000144668 | A | 5.53E-06 | 0.870868 | 1.96E-09 | NA | NA | 3 | 37571337 | ITGA9 | 1 |
| 3:37571865:A:G | GTEx/v8 | Brain_Cerebellar_Hemisphere | ENSG00000144668 | G | 2.41E-05 | 0.530436 | 1.96E-09 | NA | NA | 3 | 37571865 | ITGA9 | 1 |
| 3:37572997:C:T | GTEx/v8 | Brain_Cerebellar_Hemisphere | ENSG00000144668 | T | 5.53E-06 | 0.870868 | 1.96E-09 | NA | NA | 3 | 37572997 | ITGA9 | 1 |
| 3:37573193:A:G | GTEx/v8 | Brain_Cerebellar_Hemisphere | ENSG00000144668 | A | 5.53E-06 | 0.870868 | 1.96E-09 | NA | NA | 3 | 37573193 | ITGA9 | 1 |
| 3:37575785:G:T | GTEx/v8 | Brain_Cerebellar_Hemisphere | ENSG00000144668 | G | 5.53E-06 | 0.870868 | 1.96E-09 | NA | NA | 3 | 37575785 | ITGA9 | 1 |
| 3:37576600:G:T | GTEx/v8 | Brain_Cerebellar_Hemisphere | ENSG00000144668 | G | 5.53E-06 | 0.870868 | 1.96E-09 | NA | NA | 3 | 37576600 | ITGA9 | 1 |
| 3:37579577:G:T | GTEx/v8 | Brain_Cerebellar_Hemisphere | ENSG00000144668 | G | 5.53E-06 | 0.870868 | 1.96E-09 | NA | NA | 3 | 37579577 | ITGA9 | 1 |
| 3:37580075:A:G | GTEx/v8 | Brain_Cerebellar_Hemisphere | ENSG00000144668 | A | 5.53E-06 | 0.870868 | 1.96E-09 | NA | NA | 3 | 37580075 | ITGA9 | 1 |
| 3:37580144:A:G | GTEx/v8 | Brain_Cerebellar_Hemisphere | ENSG00000144668 | G | 5.53E-06 | 0.870868 | 1.96E-09 | NA | NA | 3 | 37580144 | ITGA9 | 1 |
| 3:37532209:A:AT | GTEx/v8 | Brain_Cerebellum | ENSG00000144668 | A | 2.92E-08 | 0.885303 | 6.50E-15 | NA | NA | 3 | 37532209 | ITGA9 | 1 |
| 3:37536056:C:T | GTEx/v8 | Brain_Cerebellum | ENSG00000144668 | T | 4.35E-08 | 0.931939 | 6.50E-15 | T | + | 3 | 37536056 | ITGA9 | 1 |
| 3:37537638:A:G | GTEx/v8 | Brain_Cerebellum | ENSG00000144668 | A | 4.35E-08 | 0.931939 | 6.50E-15 | NA | NA | 3 | 37537638 | ITGA9 | 1 |
| 3:37542234:A:G | GTEx/v8 | Brain_Cerebellum | ENSG00000144668 | A | 4.35E-08 | 0.931939 | 6.50E-15 | NA | NA | 3 | 37542234 | ITGA9 | 1 |
| 3:37548780:C:T | GTEx/v8 | Brain_Cerebellum | ENSG00000144668 | C | 1.12E-08 | 0.95804 | 6.50E-15 | NA | NA | 3 | 37548780 | ITGA9 | 1 |
| 3:37549402:C:T | GTEx/v8 | Brain_Cerebellum | ENSG00000144668 | T | 1.12E-08 | 0.95804 | 6.50E-15 | NA | NA | 3 | 37549402 | ITGA9 | 1 |
| 3:37549983:A:G | GTEx/v8 | Brain_Cerebellum | ENSG00000144668 | A | 1.12E-08 | 0.95804 | 6.50E-15 | NA | NA | 3 | 37549983 | ITGA9 | 1 |
| 3:37550382:A:G | GTEx/v8 | Brain_Cerebellum | ENSG00000144668 | A | 2.06E-05 | 0.530904 | 6.50E-15 | NA | NA | 3 | 37550382 | ITGA9 | 1 |
| 3:37551323:A:G | GTEx/v8 | Brain_Cerebellum | ENSG00000144668 | G | 1.12E-08 | 0.95804 | 6.50E-15 | NA | NA | 3 | 37551323 | ITGA9 | 1 |
| 3:37558775:C:T | GTEx/v8 | Brain_Cerebellum | ENSG00000144668 | C | 2.15E-05 | 0.564441 | 6.50E-15 | NA | NA | 3 | 37558775 | ITGA9 | 1 |
| 3:37559481:C:T | GTEx/v8 | Brain_Cerebellum | ENSG00000144668 | C | 1.12E-08 | 0.95804 | 6.50E-15 | NA | NA | 3 | 37559481 | ITGA9 | 1 |
| 3:37560114:A:T | GTEx/v8 | Brain_Cerebellum | ENSG00000144668 | T | 1.12E-08 | 0.95804 | 6.50E-15 | NA | NA | 3 | 37560114 | ITGA9 | 1 |
| 3:37560708:A:G | GTEx/v8 | Brain_Cerebellum | ENSG00000144668 | G | 1.12E-08 | 0.95804 | 6.50E-15 | NA | NA | 3 | 37560708 | ITGA9 | 1 |
| 3:37560965:A:C | GTEx/v8 | Brain_Cerebellum | ENSG00000144668 | A | 1.12E-08 | 0.95804 | 6.50E-15 | NA | NA | 3 | 37560965 | ITGA9 | 1 |
| 3:37564853:C:T | GTEx/v8 | Brain_Cerebellum | ENSG00000144668 | T | 1.12E-08 | 0.95804 | 6.50E-15 | NA | NA | 3 | 37564853 | ITGA9 | 1 |
| 3:37567033:A:G | GTEx/v8 | Brain_Cerebellum | ENSG00000144668 | G | 1.12E-08 | 0.95804 | 6.50E-15 | NA | NA | 3 | 37567033 | ITGA9 | 1 |
| 3:37567896:A:G | GTEx/v8 | Brain_Cerebellum | ENSG00000144668 | G | 5.33E-09 | 0.808777 | 6.50E-15 | NA | NA | 3 | 37567896 | ITGA9 | 1 |
| 3:37568727:A:G | GTEx/v8 | Brain_Cerebellum | ENSG00000144668 | G | 1.12E-08 | 0.95804 | 6.50E-15 | NA | NA | 3 | 37568727 | ITGA9 | 1 |
| 3:37568777:A:G | GTEx/v8 | Brain_Cerebellum | ENSG00000144668 | A | 1.12E-08 | 0.95804 | 6.50E-15 | NA | NA | 3 | 37568777 | ITGA9 | 1 |
| 3:37571337:A:G | GTEx/v8 | Brain_Cerebellum | ENSG00000144668 | A | 1.12E-08 | 0.95804 | 6.50E-15 | NA | NA | 3 | 37571337 | ITGA9 | 1 |
| 3:37571865:A:G | GTEx/v8 | Brain_Cerebellum | ENSG00000144668 | G | 1.40E-06 | 0.552662 | 6.50E-15 | NA | NA | 3 | 37571865 | ITGA9 | 1 |
| 3:37572997:C:T | GTEx/v8 | Brain_Cerebellum | ENSG00000144668 | T | 1.12E-08 | 0.95804 | 6.50E-15 | NA | NA | 3 | 37572997 | ITGA9 | 1 |
| 3:37573193:A:G | GTEx/v8 | Brain_Cerebellum | ENSG00000144668 | A | 1.12E-08 | 0.95804 | 6.50E-15 | NA | NA | 3 | 37573193 | ITGA9 | 1 |
| 3:37575785:G:T | GTEx/v8 | Brain_Cerebellum | ENSG00000144668 | G | 1.12E-08 | 0.95804 | 6.50E-15 | NA | NA | 3 | 37575785 | ITGA9 | 1 |
| 3:37576600:G:T | GTEx/v8 | Brain_Cerebellum | ENSG00000144668 | G | 1.12E-08 | 0.95804 | 6.50E-15 | NA | NA | 3 | 37576600 | ITGA9 | 1 |
| 3:37579577:G:T | GTEx/v8 | Brain_Cerebellum | ENSG00000144668 | G | 1.12E-08 | 0.95804 | 6.50E-15 | NA | NA | 3 | 37579577 | ITGA9 | 1 |
| 3:37580075:A:G | GTEx/v8 | Brain_Cerebellum | ENSG00000144668 | A | 1.12E-08 | 0.95804 | 6.50E-15 | NA | NA | 3 | 37580075 | ITGA9 | 1 |
| 3:37580144:A:G | GTEx/v8 | Brain_Cerebellum | ENSG00000144668 | G | 1.12E-08 | 0.95804 | 6.50E-15 | NA | NA | 3 | 37580144 | ITGA9 | 1 |
| 3:96537223:C:T | GTEx/v8 | Breast_Mammary_Tissue | ENSG00000269028 | C | 0.000123071 | 0.376588 | 0.030362 | NA | NA | 3 | 96537223 | MTRNR2L12 | 1 |
| 3:96540431:C:T | GTEx/v8 | Breast_Mammary_Tissue | ENSG00000269028 | T | 0.000123071 | 0.376588 | 0.030362 | NA | NA | 3 | 96540431 | MTRNR2L12 | 1 |
| 3:96540833:A:G | GTEx/v8 | Breast_Mammary_Tissue | ENSG00000269028 | A | 0.000123071 | 0.376588 | 0.030362 | NA | NA | 3 | 96540833 | MTRNR2L12 | 1 |
| 3:96544745:A:G | GTEx/v8 | Breast_Mammary_Tissue | ENSG00000269028 | G | 0.000123071 | 0.376588 | 0.030362 | NA | NA | 3 | 96544745 | MTRNR2L12 | 1 |
| 3:96545549:T:TATTTCCAAATATTTTTCCCAA | GTEx/v8 | Breast_Mammary_Tissue | ENSG00000269028 | T | 0.000123071 | 0.376588 | 0.030362 | NA | NA | 3 | 96545549 | MTRNR2L12 | 1 |
| 3:96547218:A:G | GTEx/v8 | Breast_Mammary_Tissue | ENSG00000269028 | A | 0.000123071 | 0.376588 | 0.030362 | NA | NA | 3 | 96547218 | MTRNR2L12 | 1 |
| 3:96552712:C:G | GTEx/v8 | Breast_Mammary_Tissue | ENSG00000269028 | G | 0.000123071 | 0.376588 | 0.030362 | NA | NA | 3 | 96552712 | MTRNR2L12 | 1 |
| 3:96554160:A:C | GTEx/v8 | Breast_Mammary_Tissue | ENSG00000269028 | A | 0.000123071 | 0.376588 | 0.030362 | NA | NA | 3 | 96554160 | MTRNR2L12 | 1 |
| 3:37588712:A:G | GTEx/v8 | Nerve_Tibial | ENSG00000163673 | G | 4.58E-05 | -0.211593 | 8.15E-11 | NA | NA | 3 | 37588712 | DCLK3 | 1 |
| 3:37595373:C:T | GTEx/v8 | Nerve_Tibial | ENSG00000163673 | C | 1.55E-05 | -0.220391 | 8.15E-11 | C | - | 3 | 37595373 | DCLK3 | 1 |
| 3:37532209:A:AT | GTEx/v7 | Whole_Blood | ENSG00000114739 | A | 1.97E-05 | -0.337829 | 0.000134833 | NA | NA | 3 | 37532209 | ACVR2B | 1 |
| 3:37567896:A:G | GTEx/v7 | Brain_Cerebellar_Hemisphere | ENSG00000144668 | G | 9.10E-06 | 0.834487 | 0.000231992 | NA | NA | 3 | 37567896 | ITGA9 | 1 |
| 3:37532209:A:AT | GTEx/v7 | Brain_Cerebellum | ENSG00000144668 | A | 7.99E-07 | 0.952318 | 2.97E-07 | NA | NA | 3 | 37532209 | ITGA9 | 1 |
| 3:37536056:C:T | GTEx/v7 | Brain_Cerebellum | ENSG00000144668 | T | 6.63E-07 | 1.02577 | 2.97E-07 | T | + | 3 | 37536056 | ITGA9 | 1 |
| 3:37537638:A:G | GTEx/v7 | Brain_Cerebellum | ENSG00000144668 | A | 6.63E-07 | 1.02577 | 2.97E-07 | NA | NA | 3 | 37537638 | ITGA9 | 1 |
| 3:37542234:A:G | GTEx/v7 | Brain_Cerebellum | ENSG00000144668 | A | 6.63E-07 | 1.02577 | 2.97E-07 | NA | NA | 3 | 37542234 | ITGA9 | 1 |
| 3:37548780:C:T | GTEx/v7 | Brain_Cerebellum | ENSG00000144668 | C | 6.63E-07 | 1.02577 | 2.97E-07 | NA | NA | 3 | 37548780 | ITGA9 | 1 |
| 3:37549402:C:T | GTEx/v7 | Brain_Cerebellum | ENSG00000144668 | T | 6.63E-07 | 1.02577 | 2.97E-07 | NA | NA | 3 | 37549402 | ITGA9 | 1 |
| 3:37549983:A:G | GTEx/v7 | Brain_Cerebellum | ENSG00000144668 | A | 6.63E-07 | 1.02577 | 2.97E-07 | NA | NA | 3 | 37549983 | ITGA9 | 1 |
| 3:37551323:A:G | GTEx/v7 | Brain_Cerebellum | ENSG00000144668 | G | 6.63E-07 | 1.02577 | 2.97E-07 | NA | NA | 3 | 37551323 | ITGA9 | 1 |
| 3:37551667:A:AT | GTEx/v7 | Brain_Cerebellum | ENSG00000144668 | AT | 5.53E-07 | 1.03339 | 2.97E-07 | NA | NA | 3 | 37551667 | ITGA9 | 1 |
| 3:37559481:C:T | GTEx/v7 | Brain_Cerebellum | ENSG00000144668 | C | 6.63E-07 | 1.02577 | 2.97E-07 | NA | NA | 3 | 37559481 | ITGA9 | 1 |
| 3:37560114:A:T | GTEx/v7 | Brain_Cerebellum | ENSG00000144668 | T | 6.63E-07 | 1.02577 | 2.97E-07 | NA | NA | 3 | 37560114 | ITGA9 | 1 |
| 3:37560708:A:G | GTEx/v7 | Brain_Cerebellum | ENSG00000144668 | G | 6.63E-07 | 1.02577 | 2.97E-07 | NA | NA | 3 | 37560708 | ITGA9 | 1 |
| 3:37560965:A:C | GTEx/v7 | Brain_Cerebellum | ENSG00000144668 | A | 6.63E-07 | 1.02577 | 2.97E-07 | NA | NA | 3 | 37560965 | ITGA9 | 1 |
| 3:37564853:C:T | GTEx/v7 | Brain_Cerebellum | ENSG00000144668 | T | 6.63E-07 | 1.02577 | 2.97E-07 | NA | NA | 3 | 37564853 | ITGA9 | 1 |
| 3:37567033:A:G | GTEx/v7 | Brain_Cerebellum | ENSG00000144668 | G | 6.63E-07 | 1.02577 | 2.97E-07 | NA | NA | 3 | 37567033 | ITGA9 | 1 |
| 3:37567896:A:G | GTEx/v7 | Brain_Cerebellum | ENSG00000144668 | G | 1.44E-07 | 0.889239 | 2.97E-07 | NA | NA | 3 | 37567896 | ITGA9 | 1 |
| 3:37568727:A:G | GTEx/v7 | Brain_Cerebellum | ENSG00000144668 | G | 6.63E-07 | 1.02577 | 2.97E-07 | NA | NA | 3 | 37568727 | ITGA9 | 1 |
| 3:37568777:A:G | GTEx/v7 | Brain_Cerebellum | ENSG00000144668 | A | 6.63E-07 | 1.02577 | 2.97E-07 | NA | NA | 3 | 37568777 | ITGA9 | 1 |
| 3:37571337:A:G | GTEx/v7 | Brain_Cerebellum | ENSG00000144668 | A | 6.63E-07 | 1.02577 | 2.97E-07 | NA | NA | 3 | 37571337 | ITGA9 | 1 |
| 3:37572997:C:T | GTEx/v7 | Brain_Cerebellum | ENSG00000144668 | T | 6.63E-07 | 1.02577 | 2.97E-07 | NA | NA | 3 | 37572997 | ITGA9 | 1 |
| 3:37573193:A:G | GTEx/v7 | Brain_Cerebellum | ENSG00000144668 | A | 6.63E-07 | 1.02577 | 2.97E-07 | NA | NA | 3 | 37573193 | ITGA9 | 1 |
| 3:37575785:G:T | GTEx/v7 | Brain_Cerebellum | ENSG00000144668 | G | 6.63E-07 | 1.02577 | 2.97E-07 | NA | NA | 3 | 37575785 | ITGA9 | 1 |
| 3:37576600:G:T | GTEx/v7 | Brain_Cerebellum | ENSG00000144668 | G | 6.63E-07 | 1.02577 | 2.97E-07 | NA | NA | 3 | 37576600 | ITGA9 | 1 |
| 3:37579577:G:T | GTEx/v7 | Brain_Cerebellum | ENSG00000144668 | G | 6.63E-07 | 1.02577 | 2.97E-07 | NA | NA | 3 | 37579577 | ITGA9 | 1 |
| 3:37580075:A:G | GTEx/v7 | Brain_Cerebellum | ENSG00000144668 | A | 6.63E-07 | 1.02577 | 2.97E-07 | NA | NA | 3 | 37580075 | ITGA9 | 1 |
| 3:37580144:A:G | GTEx/v7 | Brain_Cerebellum | ENSG00000144668 | G | 6.63E-07 | 1.02577 | 2.97E-07 | NA | NA | 3 | 37580144 | ITGA9 | 1 |
| 5:108591744:C:T | GTEx/v6 | Cells_EBV-transformed_lymphocytes | ENSG00000151422 | T | 4.05E-06 | -4.90289 | 0.025282482 | NA | NA | 5 | 108591744 | FER | 1 |
| 3:37571865:A:G | GTEx/v6 | Whole_Blood | ENSG00000114739 | G | 6.60E-06 | -4.58865 | 0.002468097 | NA | NA | 3 | 37571865 | ACVR2B | 1 |
| Note: uniqID = Unique ID of SNPs consists of chr:position:allele1:allele2 where alleles are alphabetically ordered; DB = Data source of eQTLs; tissue = Tissue type; Gene = ENSG ID; P-value = P-value of eQTLs; signed_stats = Signed test statistics, the actual value depends on the data source; FDR = FDR of eQTLs. Note that method to compute FDR differs between data sources; RiskIncAllele = Risk increasing allele obtained from input GWAS summary statistics. NA if signed effect is not provided in the input file. SNPs which are not in the input GWAS but included from reference panel are also encoded as "NA"; alignedDirection = The direction of effect to gene expression after aligning risk increasing allele of GWAS and tested allele of eQTLs. "+" when the risk increasing allele increase the expression of the gene, "-" when the risk increasing allele decrease the expression of the gene. NA if risk increasing allele is not defined; chr = chromosome; pos = position on hg19; symbol = Gene symbol; eqtlMapFilt = If the eQTL was used for eQTL mapping 1, otherwise 0. It can be 0 when the SNP has filtered by functional annotations such as CADD score. ID; P-value = P-value of eQTLs; signed_stats = Signed test statistics, the actual value depends on the data source; FDR = FDR of eQTLs. Note that method to compute FDR differs between data sources; RiskIncAllele = Risk increasing allele obtained from input GWAS summary statistics. NA if signed effect is not provided in the input file.SNPs which are not in the input GWAS but included from reference panel are also encoded as "NA"; alignedDirection = The direction of effect to gene expression after aligning risk increasing allele of GWAS and tested allele of eQTLs. "+" when the risk increasing allele increase the expression of the gene, "-" when the risk increasing allele decrease the expression of the gene. NA if risk increasing allele is not defined; chr = chromosome; pos = position on hg19; symbol = Gene symbol; eqtlMapFilt = If the eQTL was used for eQTL mapping 1, otherwise 0. It can be 0 when the SNP has filtered by functional annotations such as CADD score. | | | | | | | | | | | | | |
|  |
|  |
|  |
|  |
|  |
|  |
|  |
|  |
|  |
|  |
|  |
|  |

| **Table S11. Chromatin interaction regions linking GWAS risk loci of cIMT to mapped genes among PWH under 45 years old.** | | | | | | | | | | |
| --- | --- | --- | --- | --- | --- | --- | --- | --- | --- | --- |
| **GenomicLocus** | **region1** | **region2** | **FDR** | **type** | **DB** | **tissue/cell** | **inter/intra** | **SNPs** | **genes** | **ciMapFilt** |
| 4 | 5:108590000-108600000 | 5:107620000-107630000 | 0 | HiC | PsychENCODE | Promoter_anchored_loops | intra | 5:108591279:C:T;5:108591279:A:T;rs1363213;rs1862201;rs1862202;rs1862203;rs5870359 | NA | 0 |
| 4 | 5:108600000-108610000 | 5:107880000-107890000 | 0 | HiC | PsychENCODE | Promoter_anchored_loops | intra | rs11744353;rs79776200;rs76069623;rs76043709;rs78729571;rs36123121;rs11748450;rs80234158;rs78146542;rs185072862;rs137867205;rs189006725;rs4388251;rs4438924;rs4541698;rs4388252;rs4392675;rs200252534;rs145520315;rs147699420;rs77803982;rs79469094;rs75077625;rs74379173;rs78664536;rs549536839;rs570631944;rs78784808;rs78340649;rs201569786;rs75649455;rs78814720;rs77561644;rs76565008;rs74436342;rs1592807;rs1592810;rs1592811;rs7700458;rs6594365;rs6863893;rs140163909;rs146837743;rs7705919;rs201046346;rs202152005;rs199869120;rs111726541;rs373673987;rs113026830;rs148211606;rs201619508;rs201169921;rs146644914;rs116649742;rs79893293;rs57629433;rs60335509;rs58453996;rs59466242;rs60878839;rs11743452 | NA | 0 |
| 4 | 5:108610000-108620000 | 5:108000000-108010000 | 0 | HiC | PsychENCODE | Promoter_anchored_loops | intra | rs1833567;rs1833568;rs1833569;rs7721272;rs7721427;rs7721730;rs7721870;rs7722100;rs7704359;rs1895200;rs1895201;rs77974856;rs60905342;rs11746207;rs11739672;rs11749718;rs78518991;rs76243602;rs76383235 | NA | 0 |
| 4 | 5:108630000-108640000 | 5:107870000-107880000 | 0 | HiC | PsychENCODE | Promoter_anchored_loops | intra | rs10463599 | NA | 0 |
| 5 | 11:2550000-2560000 | 11:2890000-2900000 | 0 | HiC | PsychENCODE | Promoter_anchored_loops | intra | rs111815403 | NA | 0 |
| 5 | 11:2550000-2560000 | 11:3280000-3290000 | 0 | HiC | PsychENCODE | Promoter_anchored_loops | intra | rs111815403 | NA | 0 |
| 5 | 11:2550000-2560000 | 11:3420000-3430000 | 0 | HiC | PsychENCODE | Promoter_anchored_loops | intra | rs111815403 | NA | 0 |
| 6 | 12:80840000-80850000 | 12:80020000-80030000 | 0 | HiC | PsychENCODE | Promoter_anchored_loops | intra | rs200589835 | NA | 0 |
| 2 | 3:96650000-96660000 | 3:95920000-95930000 | 0 | HiC | PsychENCODE | Promoter_anchored_loops | intra | rs62263693;rs2318066;rs62263694;rs74563548;rs62263696;rs185127539;rs145486125;rs62263697;rs148690862 | NA | 0 |
| 2 | 3:96650000-96660000 | 3:96030000-96040000 | 0 | HiC | PsychENCODE | Promoter_anchored_loops | intra | rs62263693;rs2318066;rs62263694;rs74563548;rs62263696;rs185127539;rs145486125;rs62263697;rs148690862 | NA | 0 |
| 2 | 3:96650000-96660000 | 3:97330000-97340000 | 0 | HiC | PsychENCODE | Promoter_anchored_loops | intra | rs62263693;rs2318066;rs62263694;rs74563548;rs62263696;rs185127539;rs145486125;rs62263697;rs148690862 | NA | 0 |
| 2 | 3:96570000-96580000 | 3:97380000-97390000 | 0 | HiC | PsychENCODE | Promoter_anchored_loops | intra | rs62262940;rs62262941;rs62262942 | NA | 0 |
| 2 | 3:96570000-96580000 | 3:97420000-97430000 | 0 | HiC | PsychENCODE | Promoter_anchored_loops | intra | rs62262940;rs62262941;rs62262942 | NA | 0 |
| 2 | 3:96570000-96580000 | 3:97450000-97460000 | 0 | HiC | PsychENCODE | Promoter_anchored_loops | intra | rs62262940;rs62262941;rs62262942 | NA | 0 |
| 2 | 3:96570000-96580000 | 3:97480000-97490000 | 0 | HiC | PsychENCODE | Promoter_anchored_loops | intra | rs62262940;rs62262941;rs62262942 | ENSG00000113966 | 0 |
| 5 | 11:2550000-2560000 | 11:2650000-2660000 | 0 | HiC | PsychENCODE | Promoter_anchored_loops | intra | rs111815403 | NA | 0 |
| 5 | 11:2550000-2560000 | 11:2890000-2900000 | 0 | HiC | PsychENCODE | Promoter_anchored_loops | intra | rs111815403 | NA | 0 |
| 5 | 11:2550000-2560000 | 11:2910000-2920000 | 0 | HiC | PsychENCODE | Promoter_anchored_loops | intra | rs111815403 | NA | 0 |
| 5 | 11:2550000-2560000 | 11:2920000-2930000 | 0 | HiC | PsychENCODE | Promoter_anchored_loops | intra | rs111815403 | ENSG00000254827:ENSG00000110628 | 0 |
| 5 | 11:2550000-2560000 | 11:3280000-3290000 | 0 | HiC | PsychENCODE | Promoter_anchored_loops | intra | rs111815403 | NA | 0 |
| 5 | 11:2550000-2560000 | 11:3420000-3430000 | 0 | HiC | PsychENCODE | Promoter_anchored_loops | intra | rs111815403 | NA | 0 |
| 5 | 11:2550000-2560000 | 11:3540000-3550000 | 0 | HiC | PsychENCODE | Promoter_anchored_loops | intra | rs111815403 | NA | 0 |
| 5 | 11:2550000-2560000 | 11:3550000-3560000 | 0 | HiC | PsychENCODE | Promoter_anchored_loops | intra | rs111815403 | NA | 0 |
| 1 | 3:37540000-37550000 | 3:37810000-37820000 | 7.83E-28 | HiC | Giusti-Rodriguez_et_al_2019 | Adult_Cortex | intra | rs112038297;rs78544469;rs77422813;rs17228684 | NA | 0 |
| 1 | 3:37540000-37550000 | 3:38010000-38020000 | 2.40E-13 | HiC | Giusti-Rodriguez_et_al_2019 | Adult_Cortex | intra | rs112038297;rs78544469;rs77422813;rs17228684 | NA | 0 |
| 1 | 3:37540000-37550000 | 3:37490000-37500000 | 4.96E-35 | HiC | Giusti-Rodriguez_et_al_2019 | Adult_Cortex | intra | rs112038297;rs78544469;rs77422813;rs17228684 | ENSG00000144668 | 1 |
| 1 | 3:37560000-37570000 | 3:37490000-37500000 | 6.18E-15 | HiC | Giusti-Rodriguez_et_al_2019 | Adult_Cortex | intra | rs74985154;rs3733138;rs3733140;rs75619156;rs17229924;rs2162356;rs74786716;rs77820597 | ENSG00000144668 | 0 |
| 1 | 3:37570000-37580000 | 3:37490000-37500000 | 3.44E-19 | HiC | Giusti-Rodriguez_et_al_2019 | Fetal_Cortex | intra | rs112585731;rs915631;rs79149273;rs17230261;rs199607377;rs2162355;rs76952775;rs75469174 | ENSG00000144668 | 1 |
| 1 | 3:37520001-37560000 | 3:37760001-37800000 | 2.01E-07 | HiC | GSE87112 | Aorta | intra | rs113897538;rs2507941;rs17814364;rs112038297;rs78544469;rs77422813;rs17228684;rs928799;rs75425697;rs5848026;rs17814903;rs12054441 | NA | 0 |
| 1 | 3:37560001-37600000 | 3:37760001-37800000 | 5.15E-13 | HiC | GSE87112 | Aorta | intra | rs74985154;rs3733138;rs3733140;rs75619156;rs17229924;rs2162356;rs74786716;rs77820597;rs112585731;rs915631;rs79149273;rs17230261;rs199607377;rs2162355;rs76952775;rs75469174;rs78210776;rs77162103;rs75217006;rs78292414 | NA | 0 |
| 2 | 3:96600001-96640000 | 3:96680001-96720000 | 1.59E-07 | HiC | GSE87112 | Aorta | intra | rs62262952;rs62262953;rs116253310;rs76857958;rs111706803;rs62262955;rs62262957;rs62262958;rs62263676;rs187619159;rs62263677;rs62263678;rs574559070;rs534937763;rs62263680;rs62263682;rs62263684;rs16836982;rs16836986;rs112527011;rs62263685;rs62263686 | NA | 0 |
| 2 | 3:96680001-96720000 | 3:97280001-97320000 | 9.22E-07 | HiC | GSE87112 | Aorta | intra | rs62263705;rs62263706;rs6762348;rs62263709;rs137983052;rs62263734;rs2856474;rs2612274;rs553308194 | NA | 0 |
| 2 | 3:96680001-96720000 | 3:97400001-97440000 | 4.61E-08 | HiC | GSE87112 | Aorta | intra | rs62263705;rs62263706;rs6762348;rs62263709;rs137983052;rs62263734;rs2856474;rs2612274;rs553308194 | NA | 0 |
| 2 | 3:96680001-96720000 | 3:97720001-97760000 | 2.92E-11 | HiC | GSE87112 | Aorta | intra | rs62263705;rs62263706;rs6762348;rs62263709;rs137983052;rs62263734;rs2856474;rs2612274;rs553308194 | NA | 0 |
| 3 | 4:171440001-171480000 | 4:173040001-173080000 | 3.58E-09 | HiC | GSE87112 | Aorta | intra | rs147274196 | NA | 0 |
| 3 | 4:171440001-171480000 | 4:173600001-173640000 | 4.70E-07 | HiC | GSE87112 | Aorta | intra | rs147274196 | NA | 0 |
| 3 | 4:171480001-171520000 | 4:172480001-172520000 | 5.96E-07 | HiC | GSE87112 | Aorta | intra | rs148420952;rs76413646 | NA | 0 |
| 6 | 12:80800001-80840000 | 12:80920001-80960000 | 7.88E-15 | HiC | GSE87112 | Aorta | intra | rs117203215;rs77741796 | NA | 0 |
| 6 | 12:80800001-80840000 | 12:81000001-81040000 | 4.20E-16 | HiC | GSE87112 | Aorta | intra | rs117203215;rs77741796 | NA | 0 |
| 6 | 12:80800001-80840000 | 12:81080001-81120000 | 3.01E-11 | HiC | GSE87112 | Aorta | intra | rs117203215;rs77741796 | ENSG00000111046:ENSG00000111049 | 0 |
| 6 | 12:80800001-80840000 | 12:81120001-81160000 | 6.47E-16 | HiC | GSE87112 | Aorta | intra | rs117203215;rs77741796 | NA | 0 |
| 6 | 12:80800001-80840000 | 12:81160001-81200000 | 1.64E-09 | HiC | GSE87112 | Aorta | intra | rs117203215;rs77741796 | NA | 0 |
| 6 | 12:80800001-80840000 | 12:81240001-81280000 | 1.26E-07 | HiC | GSE87112 | Aorta | intra | rs117203215;rs77741796 | NA | 0 |
| 6 | 12:80800001-80840000 | 12:81280001-81320000 | 2.86E-10 | HiC | GSE87112 | Aorta | intra | rs117203215;rs77741796 | NA | 0 |
| 6 | 12:80800001-80840000 | 12:81320001-81360000 | 1.82E-11 | HiC | GSE87112 | Aorta | intra | rs117203215;rs77741796 | ENSG00000111052:ENSG00000111058 | 0 |
| 6 | 12:80800001-80840000 | 12:81480001-81520000 | 6.47E-11 | HiC | GSE87112 | Aorta | intra | rs117203215;rs77741796 | NA | 0 |
| 6 | 12:80800001-80840000 | 12:81640001-81680000 | 2.18E-10 | HiC | GSE87112 | Aorta | intra | rs117203215;rs77741796 | NA | 0 |
| 7 | 12:127760001-127800000 | 12:127800001-127840000 | 6.57E-16 | HiC | GSE87112 | Aorta | intra | rs1810088;rs367839627;rs146043604;rs143439170;rs10773382;rs10847307;rs35154640;rs1552386;rs7314186;rs1979065;rs5801722;rs10744302;12:127775513:A:C;rs10734938;rs35024927;rs10773387;rs978813;rs2348321;rs10847321;rs10847323;rs6489163 | NA | 0 |
| 7 | 12:127760001-127800000 | 12:128400001-128440000 | 2.22E-10 | HiC | GSE87112 | Aorta | intra | rs1810088;rs367839627;rs146043604;rs143439170;rs10773382;rs10847307;rs35154640;rs1552386;rs7314186;rs1979065;rs5801722;rs10744302;12:127775513:A:C;rs10734938;rs35024927;rs10773387;rs978813;rs2348321;rs10847321;rs10847323;rs6489163 | NA | 0 |
| 2 | 3:96520001-96560000 | 3:94000001-94040000 | 5.69E-09 | HiC | GSE87112 | Aorta | intra | rs62262901;rs62262902;rs62262903;rs62262930;rs151165295;rs62262931;rs62262933;rs62262934;rs74641574;rs79645223;rs2318156;rs2318155;rs2318154 | NA | 0 |
| 2 | 3:96600001-96640000 | 3:94000001-94040000 | 1.13E-11 | HiC | GSE87112 | Aorta | intra | rs62262952;rs62262953;rs116253310;rs76857958;rs111706803;rs62262955;rs62262957;rs62262958;rs62263676;rs187619159;rs62263677;rs62263678;rs574559070;rs534937763;rs62263680;rs62263682;rs62263684;rs16836982;rs16836986;rs112527011;rs62263685;rs62263686 | NA | 0 |
| 2 | 3:96680001-96720000 | 3:94000001-94040000 | 3.94E-11 | HiC | GSE87112 | Aorta | intra | rs62263705;rs62263706;rs6762348;rs62263709;rs137983052;rs62263734;rs2856474;rs2612274;rs553308194 | NA | 0 |
| 2 | 3:96520001-96560000 | 3:94040001-94080000 | 2.27E-07 | HiC | GSE87112 | Aorta | intra | rs62262901;rs62262902;rs62262903;rs62262930;rs151165295;rs62262931;rs62262933;rs62262934;rs74641574;rs79645223;rs2318156;rs2318155;rs2318154 | NA | 0 |
| 2 | 3:96680001-96720000 | 3:96600001-96640000 | 1.59E-07 | HiC | GSE87112 | Aorta | intra | rs62263705;rs62263706;rs6762348;rs62263709;rs137983052;rs62263734;rs2856474;rs2612274;rs553308194 | NA | 0 |
| 6 | 12:80800001-80840000 | 12:80680001-80720000 | 1.36E-10 | HiC | GSE87112 | Aorta | intra | rs117203215;rs77741796 | NA | 0 |
| 7 | 12:127760001-127800000 | 12:127800001-127840000 | 8.44E-07 | HiC | GSE87112 | Bladder | intra | rs1810088;rs367839627;rs146043604;rs143439170;rs10773382;rs10847307;rs35154640;rs1552386;rs7314186;rs1979065;rs5801722;rs10744302;12:127775513:A:C;rs10734938;rs35024927;rs10773387;rs978813;rs2348321;rs10847321;rs10847323;rs6489163 | NA | 0 |
| 1 | 3:37520001-37560000 | 3:37760001-37800000 | 3.87E-12 | HiC | GSE87112 | Left_Ventricle | intra | rs113897538;rs2507941;rs17814364;rs112038297;rs78544469;rs77422813;rs17228684;rs928799;rs75425697;rs5848026;rs17814903;rs12054441 | NA | 0 |
| 1 | 3:37520001-37560000 | 3:37800001-37840000 | 1.02E-15 | HiC | GSE87112 | Left_Ventricle | intra | rs113897538;rs2507941;rs17814364;rs112038297;rs78544469;rs77422813;rs17228684;rs928799;rs75425697;rs5848026;rs17814903;rs12054441 | NA | 0 |
| 1 | 3:37560001-37600000 | 3:37720001-37760000 | 8.14E-14 | HiC | GSE87112 | Left_Ventricle | intra | rs74985154;rs3733138;rs3733140;rs75619156;rs17229924;rs2162356;rs74786716;rs77820597;rs112585731;rs915631;rs79149273;rs17230261;rs199607377;rs2162355;rs76952775;rs75469174;rs78210776;rs77162103;rs75217006;rs78292414 | NA | 0 |
| 1 | 3:37560001-37600000 | 3:37760001-37800000 | 1.67E-08 | HiC | GSE87112 | Left_Ventricle | intra | rs74985154;rs3733138;rs3733140;rs75619156;rs17229924;rs2162356;rs74786716;rs77820597;rs112585731;rs915631;rs79149273;rs17230261;rs199607377;rs2162355;rs76952775;rs75469174;rs78210776;rs77162103;rs75217006;rs78292414 | NA | 0 |
| 1 | 3:37560001-37600000 | 3:37800001-37840000 | 7.48E-37 | HiC | GSE87112 | Left_Ventricle | intra | rs74985154;rs3733138;rs3733140;rs75619156;rs17229924;rs2162356;rs74786716;rs77820597;rs112585731;rs915631;rs79149273;rs17230261;rs199607377;rs2162355;rs76952775;rs75469174;rs78210776;rs77162103;rs75217006;rs78292414 | NA | 0 |
| 2 | 3:96600001-96640000 | 3:96680001-96720000 | 2.55E-20 | HiC | GSE87112 | Left_Ventricle | intra | rs62262952;rs62262953;rs116253310;rs76857958;rs111706803;rs62262955;rs62262957;rs62262958;rs62263676;rs187619159;rs62263677;rs62263678;rs574559070;rs534937763;rs62263680;rs62263682;rs62263684;rs16836982;rs16836986;rs112527011;rs62263685;rs62263686 | NA | 0 |
| 2 | 3:96600001-96640000 | 3:97120001-97160000 | 5.36E-08 | HiC | GSE87112 | Left_Ventricle | intra | rs62262952;rs62262953;rs116253310;rs76857958;rs111706803;rs62262955;rs62262957;rs62262958;rs62263676;rs187619159;rs62263677;rs62263678;rs574559070;rs534937763;rs62263680;rs62263682;rs62263684;rs16836982;rs16836986;rs112527011;rs62263685;rs62263686 | NA | 0 |
| 2 | 3:96600001-96640000 | 3:97280001-97320000 | 4.16E-07 | HiC | GSE87112 | Left_Ventricle | intra | rs62262952;rs62262953;rs116253310;rs76857958;rs111706803;rs62262955;rs62262957;rs62262958;rs62263676;rs187619159;rs62263677;rs62263678;rs574559070;rs534937763;rs62263680;rs62263682;rs62263684;rs16836982;rs16836986;rs112527011;rs62263685;rs62263686 | NA | 0 |
| 2 | 3:96600001-96640000 | 3:97360001-97400000 | 2.59E-07 | HiC | GSE87112 | Left_Ventricle | intra | rs62262952;rs62262953;rs116253310;rs76857958;rs111706803;rs62262955;rs62262957;rs62262958;rs62263676;rs187619159;rs62263677;rs62263678;rs574559070;rs534937763;rs62263680;rs62263682;rs62263684;rs16836982;rs16836986;rs112527011;rs62263685;rs62263686 | NA | 0 |
| 2 | 3:96600001-96640000 | 3:97400001-97440000 | 2.39E-07 | HiC | GSE87112 | Left_Ventricle | intra | rs62262952;rs62262953;rs116253310;rs76857958;rs111706803;rs62262955;rs62262957;rs62262958;rs62263676;rs187619159;rs62263677;rs62263678;rs574559070;rs534937763;rs62263680;rs62263682;rs62263684;rs16836982;rs16836986;rs112527011;rs62263685;rs62263686 | NA | 0 |
| 2 | 3:96680001-96720000 | 3:97000001-97040000 | 1.50E-10 | HiC | GSE87112 | Left_Ventricle | intra | rs62263705;rs62263706;rs6762348;rs62263709;rs137983052;rs62263734;rs2856474;rs2612274;rs553308194 | NA | 0 |
| 2 | 3:96680001-96720000 | 3:97200001-97240000 | 1.50E-17 | HiC | GSE87112 | Left_Ventricle | intra | rs62263705;rs62263706;rs6762348;rs62263709;rs137983052;rs62263734;rs2856474;rs2612274;rs553308194 | NA | 0 |
| 2 | 3:96680001-96720000 | 3:97360001-97400000 | 9.02E-10 | HiC | GSE87112 | Left_Ventricle | intra | rs62263705;rs62263706;rs6762348;rs62263709;rs137983052;rs62263734;rs2856474;rs2612274;rs553308194 | NA | 0 |
| 2 | 3:96680001-96720000 | 3:97720001-97760000 | 2.15E-09 | HiC | GSE87112 | Left_Ventricle | intra | rs62263705;rs62263706;rs6762348;rs62263709;rs137983052;rs62263734;rs2856474;rs2612274;rs553308194 | NA | 0 |
| 3 | 4:171440001-171480000 | 4:171640001-171680000 | 9.15E-07 | HiC | GSE87112 | Left_Ventricle | intra | rs147274196 | NA | 0 |
| 3 | 4:171440001-171480000 | 4:173280001-173320000 | 9.92E-08 | HiC | GSE87112 | Left_Ventricle | intra | rs147274196 | NA | 0 |
| 3 | 4:171440001-171480000 | 4:173600001-173640000 | 5.67E-09 | HiC | GSE87112 | Left_Ventricle | intra | rs147274196 | NA | 0 |
| 3 | 4:171480001-171520000 | 4:171520001-171560000 | 7.26E-09 | HiC | GSE87112 | Left_Ventricle | intra | rs148420952;rs76413646 | NA | 0 |
| 3 | 4:171480001-171520000 | 4:172680001-172720000 | 9.23E-11 | HiC | GSE87112 | Left_Ventricle | intra | rs148420952;rs76413646 | NA | 0 |
| 3 | 4:171480001-171520000 | 4:172840001-172880000 | 9.50E-07 | HiC | GSE87112 | Left_Ventricle | intra | rs148420952;rs76413646 | NA | 0 |
| 3 | 4:171480001-171520000 | 4:173080001-173120000 | 1.52E-14 | HiC | GSE87112 | Left_Ventricle | intra | rs148420952;rs76413646 | NA | 0 |
| 3 | 4:171480001-171520000 | 4:173360001-173400000 | 2.52E-07 | HiC | GSE87112 | Left_Ventricle | intra | rs148420952;rs76413646 | NA | 0 |
| 4 | 5:108560001-108600000 | 5:109840001-109880000 | 2.59E-07 | HiC | GSE87112 | Left_Ventricle | intra | rs35812497;rs75803484;rs75073283;rs76430100;rs75782477;rs77992445;rs61701247;rs17161655;rs115979287;rs17161659;rs151072743;rs17161665;rs6863688;5:108591279:C:T;5:108591279:A:T;rs1363213;rs1862201;rs1862202;rs1862203;rs5870359 | NA | 0 |
| 4 | 5:108600001-108640000 | 5:109760001-109800000 | 2.04E-07 | HiC | GSE87112 | Left_Ventricle | intra | rs11744353;rs79776200;rs76069623;rs76043709;rs78729571;rs36123121;rs11748450;rs80234158;rs78146542;rs185072862;rs137867205;rs189006725;rs4388251;rs4438924;rs4541698;rs4388252;rs4392675;rs200252534;rs145520315;rs147699420;rs77803982;rs79469094;rs75077625;rs74379173;rs78664536;rs549536839;rs570631944;rs78784808;rs78340649;rs201569786;rs75649455;rs78814720;rs77561644;rs76565008;rs74436342;rs1592807;rs1592810;rs1592811;rs7700458;rs6594365;rs6863893;rs140163909;rs146837743;rs7705919;rs201046346;rs202152005;rs199869120;rs111726541;rs373673987;rs113026830;rs148211606;rs201619508;rs201169921;rs146644914;rs116649742;rs79893293;rs57629433;rs60335509;rs58453996;rs59466242;rs60878839;rs11743452;rs1833567;rs1833568;rs1833569;rs7721272;rs7721427;rs7721730;rs7721870;rs7722100;rs7704359;rs1895200;rs1895201;rs77974856;rs60905342;rs11746207;rs11739672;rs11749718;rs78518991;rs76243602;rs76383235;rs11741980;rs35243960;rs116462975;rs139801813;rs113103584;rs74727230;rs75919325;rs79624714;rs2080858;rs138092354;rs148285468;rs114455395;rs74850689;rs10463599 | NA | 0 |
| 6 | 12:80800001-80840000 | 12:80880001-80920000 | 8.44E-09 | HiC | GSE87112 | Left_Ventricle | intra | rs117203215;rs77741796 | NA | 0 |
| 6 | 12:80800001-80840000 | 12:80920001-80960000 | 1.70E-20 | HiC | GSE87112 | Left_Ventricle | intra | rs117203215;rs77741796 | NA | 0 |
| 6 | 12:80800001-80840000 | 12:81000001-81040000 | 8.11E-09 | HiC | GSE87112 | Left_Ventricle | intra | rs117203215;rs77741796 | NA | 0 |
| 6 | 12:80800001-80840000 | 12:81040001-81080000 | 2.23E-18 | HiC | GSE87112 | Left_Ventricle | intra | rs117203215;rs77741796 | NA | 0 |
| 6 | 12:80800001-80840000 | 12:81080001-81120000 | 8.97E-15 | HiC | GSE87112 | Left_Ventricle | intra | rs117203215;rs77741796 | ENSG00000111046:ENSG00000111049 | 0 |
| 6 | 12:80800001-80840000 | 12:81120001-81160000 | 4.87E-17 | HiC | GSE87112 | Left_Ventricle | intra | rs117203215;rs77741796 | NA | 0 |
| 6 | 12:80800001-80840000 | 12:81160001-81200000 | 2.00E-09 | HiC | GSE87112 | Left_Ventricle | intra | rs117203215;rs77741796 | NA | 0 |
| 6 | 12:80800001-80840000 | 12:81240001-81280000 | 2.75E-15 | HiC | GSE87112 | Left_Ventricle | intra | rs117203215;rs77741796 | NA | 0 |
| 6 | 12:80800001-80840000 | 12:81320001-81360000 | 3.12E-10 | HiC | GSE87112 | Left_Ventricle | intra | rs117203215;rs77741796 | ENSG00000111052:ENSG00000111058 | 0 |
| 6 | 12:80800001-80840000 | 12:81400001-81440000 | 5.97E-07 | HiC | GSE87112 | Left_Ventricle | intra | rs117203215;rs77741796 | NA | 0 |
| 6 | 12:80800001-80840000 | 12:81440001-81480000 | 5.94E-12 | HiC | GSE87112 | Left_Ventricle | intra | rs117203215;rs77741796 | NA | 0 |
| 6 | 12:80800001-80840000 | 12:81480001-81520000 | 1.17E-15 | HiC | GSE87112 | Left_Ventricle | intra | rs117203215;rs77741796 | NA | 0 |
| 6 | 12:80800001-80840000 | 12:81600001-81640000 | 2.07E-10 | HiC | GSE87112 | Left_Ventricle | intra | rs117203215;rs77741796 | NA | 0 |
| 6 | 12:80800001-80840000 | 12:81760001-81800000 | 1.17E-10 | HiC | GSE87112 | Left_Ventricle | intra | rs117203215;rs77741796 | NA | 0 |
| 6 | 12:80800001-80840000 | 12:81920001-81960000 | 1.39E-10 | HiC | GSE87112 | Left_Ventricle | intra | rs117203215;rs77741796 | NA | 0 |
| 6 | 12:80800001-80840000 | 12:82200001-82240000 | 3.43E-08 | HiC | GSE87112 | Left_Ventricle | intra | rs117203215;rs77741796 | NA | 0 |
| 6 | 12:80840001-80880000 | 12:80920001-80960000 | 3.43E-15 | HiC | GSE87112 | Left_Ventricle | intra | rs200589835 | NA | 0 |
| 6 | 12:80840001-80880000 | 12:81000001-81040000 | 3.78E-15 | HiC | GSE87112 | Left_Ventricle | intra | rs200589835 | NA | 0 |
| 6 | 12:80840001-80880000 | 12:81080001-81120000 | 1.31E-07 | HiC | GSE87112 | Left_Ventricle | intra | rs200589835 | ENSG00000111046:ENSG00000111049 | 0 |
| 6 | 12:80840001-80880000 | 12:81160001-81200000 | 2.06E-09 | HiC | GSE87112 | Left_Ventricle | intra | rs200589835 | NA | 0 |
| 6 | 12:80840001-80880000 | 12:81600001-81640000 | 5.97E-07 | HiC | GSE87112 | Left_Ventricle | intra | rs200589835 | NA | 0 |
| 6 | 12:80840001-80880000 | 12:81640001-81680000 | 2.58E-22 | HiC | GSE87112 | Left_Ventricle | intra | rs200589835 | NA | 0 |
| 6 | 12:80840001-80880000 | 12:81760001-81800000 | 1.30E-08 | HiC | GSE87112 | Left_Ventricle | intra | rs200589835 | NA | 0 |
| 7 | 12:127760001-127800000 | 12:127800001-127840000 | 6.12E-42 | HiC | GSE87112 | Left_Ventricle | intra | rs1810088;rs367839627;rs146043604;rs143439170;rs10773382;rs10847307;rs35154640;rs1552386;rs7314186;rs1979065;rs5801722;rs10744302;12:127775513:A:C;rs10734938;rs35024927;rs10773387;rs978813;rs2348321;rs10847321;rs10847323;rs6489163 | NA | 0 |
| 7 | 12:127760001-127800000 | 12:127840001-127880000 | 1.29E-09 | HiC | GSE87112 | Left_Ventricle | intra | rs1810088;rs367839627;rs146043604;rs143439170;rs10773382;rs10847307;rs35154640;rs1552386;rs7314186;rs1979065;rs5801722;rs10744302;12:127775513:A:C;rs10734938;rs35024927;rs10773387;rs978813;rs2348321;rs10847321;rs10847323;rs6489163 | NA | 0 |
| 7 | 12:127760001-127800000 | 12:128400001-128440000 | 1.39E-07 | HiC | GSE87112 | Left_Ventricle | intra | rs1810088;rs367839627;rs146043604;rs143439170;rs10773382;rs10847307;rs35154640;rs1552386;rs7314186;rs1979065;rs5801722;rs10744302;12:127775513:A:C;rs10734938;rs35024927;rs10773387;rs978813;rs2348321;rs10847321;rs10847323;rs6489163 | NA | 0 |
| 7 | 12:127760001-127800000 | 12:129560001-129600000 | 8.20E-07 | HiC | GSE87112 | Left_Ventricle | intra | rs1810088;rs367839627;rs146043604;rs143439170;rs10773382;rs10847307;rs35154640;rs1552386;rs7314186;rs1979065;rs5801722;rs10744302;12:127775513:A:C;rs10734938;rs35024927;rs10773387;rs978813;rs2348321;rs10847321;rs10847323;rs6489163 | NA | 0 |
| 7 | 12:127760001-127800000 | 12:129800001-129840000 | 9.58E-08 | HiC | GSE87112 | Left_Ventricle | intra | rs1810088;rs367839627;rs146043604;rs143439170;rs10773382;rs10847307;rs35154640;rs1552386;rs7314186;rs1979065;rs5801722;rs10744302;12:127775513:A:C;rs10734938;rs35024927;rs10773387;rs978813;rs2348321;rs10847321;rs10847323;rs6489163 | NA | 0 |
| 7 | 12:127760001-127800000 | 12:130400001-130440000 | 1.82E-14 | HiC | GSE87112 | Left_Ventricle | intra | rs1810088;rs367839627;rs146043604;rs143439170;rs10773382;rs10847307;rs35154640;rs1552386;rs7314186;rs1979065;rs5801722;rs10744302;12:127775513:A:C;rs10734938;rs35024927;rs10773387;rs978813;rs2348321;rs10847321;rs10847323;rs6489163 | NA | 0 |
| 2 | 3:96680001-96720000 | 3:93800001-93840000 | 1.01E-11 | HiC | GSE87112 | Left_Ventricle | intra | rs62263705;rs62263706;rs6762348;rs62263709;rs137983052;rs62263734;rs2856474;rs2612274;rs553308194 | NA | 0 |
| 2 | 3:96600001-96640000 | 3:93840001-93880000 | 1.10E-07 | HiC | GSE87112 | Left_Ventricle | intra | rs62262952;rs62262953;rs116253310;rs76857958;rs111706803;rs62262955;rs62262957;rs62262958;rs62263676;rs187619159;rs62263677;rs62263678;rs574559070;rs534937763;rs62263680;rs62263682;rs62263684;rs16836982;rs16836986;rs112527011;rs62263685;rs62263686 | NA | 0 |
| 2 | 3:96520001-96560000 | 3:93880001-93920000 | 3.40E-07 | HiC | GSE87112 | Left_Ventricle | intra | rs62262901;rs62262902;rs62262903;rs62262930;rs151165295;rs62262931;rs62262933;rs62262934;rs74641574;rs79645223;rs2318156;rs2318155;rs2318154 | NA | 0 |
| 2 | 3:96560001-96600000 | 3:93880001-93920000 | 9.63E-09 | HiC | GSE87112 | Left_Ventricle | intra | rs16836353;rs62262935;rs62262936;rs62262937;rs62262938;rs62262940;rs62262941;rs62262942;rs62262944;rs62262946;rs62262947;rs62262948;rs62262949;rs62262950;rs62262951 | NA | 0 |
| 2 | 3:96680001-96720000 | 3:93880001-93920000 | 7.09E-18 | HiC | GSE87112 | Left_Ventricle | intra | rs62263705;rs62263706;rs6762348;rs62263709;rs137983052;rs62263734;rs2856474;rs2612274;rs553308194 | NA | 0 |
| 2 | 3:96560001-96600000 | 3:93960001-94000000 | 3.73E-18 | HiC | GSE87112 | Left_Ventricle | intra | rs16836353;rs62262935;rs62262936;rs62262937;rs62262938;rs62262940;rs62262941;rs62262942;rs62262944;rs62262946;rs62262947;rs62262948;rs62262949;rs62262950;rs62262951 | NA | 0 |
| 2 | 3:96520001-96560000 | 3:94000001-94040000 | 6.70E-16 | HiC | GSE87112 | Left_Ventricle | intra | rs62262901;rs62262902;rs62262903;rs62262930;rs151165295;rs62262931;rs62262933;rs62262934;rs74641574;rs79645223;rs2318156;rs2318155;rs2318154 | NA | 0 |
| 2 | 3:96560001-96600000 | 3:94000001-94040000 | 1.40E-07 | HiC | GSE87112 | Left_Ventricle | intra | rs16836353;rs62262935;rs62262936;rs62262937;rs62262938;rs62262940;rs62262941;rs62262942;rs62262944;rs62262946;rs62262947;rs62262948;rs62262949;rs62262950;rs62262951 | NA | 0 |
| 2 | 3:96600001-96640000 | 3:94000001-94040000 | 7.78E-28 | HiC | GSE87112 | Left_Ventricle | intra | rs62262952;rs62262953;rs116253310;rs76857958;rs111706803;rs62262955;rs62262957;rs62262958;rs62263676;rs187619159;rs62263677;rs62263678;rs574559070;rs534937763;rs62263680;rs62263682;rs62263684;rs16836982;rs16836986;rs112527011;rs62263685;rs62263686 | NA | 0 |
| 2 | 3:96640001-96680000 | 3:94000001-94040000 | 3.32E-13 | HiC | GSE87112 | Left_Ventricle | intra | rs62263687;rs62263693;rs2318066;rs62263694;rs74563548;rs62263696;rs185127539;rs145486125;rs62263697;rs148690862;rs62263699;rs149222683;rs111574505;rs62263701;rs62263702;rs62263703;rs75261331;rs62263704;rs189373463 | NA | 0 |
| 2 | 3:96680001-96720000 | 3:94000001-94040000 | 1.02E-30 | HiC | GSE87112 | Left_Ventricle | intra | rs62263705;rs62263706;rs6762348;rs62263709;rs137983052;rs62263734;rs2856474;rs2612274;rs553308194 | NA | 0 |
| 2 | 3:96600001-96640000 | 3:94040001-94080000 | 1.08E-09 | HiC | GSE87112 | Left_Ventricle | intra | rs62262952;rs62262953;rs116253310;rs76857958;rs111706803;rs62262955;rs62262957;rs62262958;rs62263676;rs187619159;rs62263677;rs62263678;rs574559070;rs534937763;rs62263680;rs62263682;rs62263684;rs16836982;rs16836986;rs112527011;rs62263685;rs62263686 | NA | 0 |
| 2 | 3:96520001-96560000 | 3:94120001-94160000 | 9.79E-08 | HiC | GSE87112 | Left_Ventricle | intra | rs62262901;rs62262902;rs62262903;rs62262930;rs151165295;rs62262931;rs62262933;rs62262934;rs74641574;rs79645223;rs2318156;rs2318155;rs2318154 | NA | 0 |
| 2 | 3:96680001-96720000 | 3:94120001-94160000 | 1.40E-07 | HiC | GSE87112 | Left_Ventricle | intra | rs62263705;rs62263706;rs6762348;rs62263709;rs137983052;rs62263734;rs2856474;rs2612274;rs553308194 | NA | 0 |
| 2 | 3:96680001-96720000 | 3:94160001-94200000 | 5.06E-11 | HiC | GSE87112 | Left_Ventricle | intra | rs62263705;rs62263706;rs6762348;rs62263709;rs137983052;rs62263734;rs2856474;rs2612274;rs553308194 | NA | 0 |
| 2 | 3:96520001-96560000 | 3:94240001-94280000 | 1.50E-11 | HiC | GSE87112 | Left_Ventricle | intra | rs62262901;rs62262902;rs62262903;rs62262930;rs151165295;rs62262931;rs62262933;rs62262934;rs74641574;rs79645223;rs2318156;rs2318155;rs2318154 | NA | 0 |
| 2 | 3:96560001-96600000 | 3:94480001-94520000 | 8.83E-07 | HiC | GSE87112 | Left_Ventricle | intra | rs16836353;rs62262935;rs62262936;rs62262937;rs62262938;rs62262940;rs62262941;rs62262942;rs62262944;rs62262946;rs62262947;rs62262948;rs62262949;rs62262950;rs62262951 | NA | 0 |
| 2 | 3:96680001-96720000 | 3:94480001-94520000 | 2.01E-08 | HiC | GSE87112 | Left_Ventricle | intra | rs62263705;rs62263706;rs6762348;rs62263709;rs137983052;rs62263734;rs2856474;rs2612274;rs553308194 | NA | 0 |
| 2 | 3:96600001-96640000 | 3:94520001-94560000 | 2.18E-07 | HiC | GSE87112 | Left_Ventricle | intra | rs62262952;rs62262953;rs116253310;rs76857958;rs111706803;rs62262955;rs62262957;rs62262958;rs62263676;rs187619159;rs62263677;rs62263678;rs574559070;rs534937763;rs62263680;rs62263682;rs62263684;rs16836982;rs16836986;rs112527011;rs62263685;rs62263686 | NA | 0 |
| 2 | 3:96680001-96720000 | 3:94520001-94560000 | 8.49E-12 | HiC | GSE87112 | Left_Ventricle | intra | rs62263705;rs62263706;rs6762348;rs62263709;rs137983052;rs62263734;rs2856474;rs2612274;rs553308194 | NA | 0 |
| 2 | 3:96560001-96600000 | 3:94600001-94640000 | 3.28E-08 | HiC | GSE87112 | Left_Ventricle | intra | rs16836353;rs62262935;rs62262936;rs62262937;rs62262938;rs62262940;rs62262941;rs62262942;rs62262944;rs62262946;rs62262947;rs62262948;rs62262949;rs62262950;rs62262951 | NA | 0 |
| 2 | 3:96680001-96720000 | 3:94640001-94680000 | 4.69E-15 | HiC | GSE87112 | Left_Ventricle | intra | rs62263705;rs62263706;rs6762348;rs62263709;rs137983052;rs62263734;rs2856474;rs2612274;rs553308194 | NA | 0 |
| 2 | 3:96520001-96560000 | 3:94680001-94720000 | 9.55E-18 | HiC | GSE87112 | Left_Ventricle | intra | rs62262901;rs62262902;rs62262903;rs62262930;rs151165295;rs62262931;rs62262933;rs62262934;rs74641574;rs79645223;rs2318156;rs2318155;rs2318154 | NA | 0 |
| 2 | 3:96600001-96640000 | 3:94840001-94880000 | 2.34E-07 | HiC | GSE87112 | Left_Ventricle | intra | rs62262952;rs62262953;rs116253310;rs76857958;rs111706803;rs62262955;rs62262957;rs62262958;rs62263676;rs187619159;rs62263677;rs62263678;rs574559070;rs534937763;rs62263680;rs62263682;rs62263684;rs16836982;rs16836986;rs112527011;rs62263685;rs62263686 | NA | 0 |
| 2 | 3:96520001-96560000 | 3:95000001-95040000 | 3.28E-10 | HiC | GSE87112 | Left_Ventricle | intra | rs62262901;rs62262902;rs62262903;rs62262930;rs151165295;rs62262931;rs62262933;rs62262934;rs74641574;rs79645223;rs2318156;rs2318155;rs2318154 | NA | 0 |
| 2 | 3:96520001-96560000 | 3:95120001-95160000 | 4.67E-07 | HiC | GSE87112 | Left_Ventricle | intra | rs62262901;rs62262902;rs62262903;rs62262930;rs151165295;rs62262931;rs62262933;rs62262934;rs74641574;rs79645223;rs2318156;rs2318155;rs2318154 | NA | 0 |
| 2 | 3:96600001-96640000 | 3:95120001-95160000 | 1.05E-08 | HiC | GSE87112 | Left_Ventricle | intra | rs62262952;rs62262953;rs116253310;rs76857958;rs111706803;rs62262955;rs62262957;rs62262958;rs62263676;rs187619159;rs62263677;rs62263678;rs574559070;rs534937763;rs62263680;rs62263682;rs62263684;rs16836982;rs16836986;rs112527011;rs62263685;rs62263686 | NA | 0 |
| 2 | 3:96680001-96720000 | 3:95120001-95160000 | 3.02E-09 | HiC | GSE87112 | Left_Ventricle | intra | rs62263705;rs62263706;rs6762348;rs62263709;rs137983052;rs62263734;rs2856474;rs2612274;rs553308194 | NA | 0 |
| 2 | 3:96520001-96560000 | 3:95200001-95240000 | 1.20E-11 | HiC | GSE87112 | Left_Ventricle | intra | rs62262901;rs62262902;rs62262903;rs62262930;rs151165295;rs62262931;rs62262933;rs62262934;rs74641574;rs79645223;rs2318156;rs2318155;rs2318154 | NA | 0 |
| 2 | 3:96680001-96720000 | 3:95400001-95440000 | 7.83E-16 | HiC | GSE87112 | Left_Ventricle | intra | rs62263705;rs62263706;rs6762348;rs62263709;rs137983052;rs62263734;rs2856474;rs2612274;rs553308194 | NA | 0 |
| 2 | 3:96680001-96720000 | 3:96600001-96640000 | 2.55E-20 | HiC | GSE87112 | Left_Ventricle | intra | rs62263705;rs62263706;rs6762348;rs62263709;rs137983052;rs62263734;rs2856474;rs2612274;rs553308194 | NA | 0 |
| 3 | 4:171440001-171480000 | 4:171400001-171440000 | 5.15E-10 | HiC | GSE87112 | Left_Ventricle | intra | rs147274196 | NA | 0 |
| 4 | 5:108560001-108600000 | 5:107960001-108000000 | 3.53E-21 | HiC | GSE87112 | Left_Ventricle | intra | rs35812497;rs75803484;rs75073283;rs76430100;rs75782477;rs77992445;rs61701247;rs17161655;rs115979287;rs17161659;rs151072743;rs17161665;rs6863688;5:108591279:C:T;5:108591279:A:T;rs1363213;rs1862201;rs1862202;rs1862203;rs5870359 | NA | 0 |
| 4 | 5:108600001-108640000 | 5:107960001-108000000 | 4.12E-12 | HiC | GSE87112 | Left_Ventricle | intra | rs11744353;rs79776200;rs76069623;rs76043709;rs78729571;rs36123121;rs11748450;rs80234158;rs78146542;rs185072862;rs137867205;rs189006725;rs4388251;rs4438924;rs4541698;rs4388252;rs4392675;rs200252534;rs145520315;rs147699420;rs77803982;rs79469094;rs75077625;rs74379173;rs78664536;rs549536839;rs570631944;rs78784808;rs78340649;rs201569786;rs75649455;rs78814720;rs77561644;rs76565008;rs74436342;rs1592807;rs1592810;rs1592811;rs7700458;rs6594365;rs6863893;rs140163909;rs146837743;rs7705919;rs201046346;rs202152005;rs199869120;rs111726541;rs373673987;rs113026830;rs148211606;rs201619508;rs201169921;rs146644914;rs116649742;rs79893293;rs57629433;rs60335509;rs58453996;rs59466242;rs60878839;rs11743452;rs1833567;rs1833568;rs1833569;rs7721272;rs7721427;rs7721730;rs7721870;rs7722100;rs7704359;rs1895200;rs1895201;rs77974856;rs60905342;rs11746207;rs11739672;rs11749718;rs78518991;rs76243602;rs76383235;rs11741980;rs35243960;rs116462975;rs139801813;rs113103584;rs74727230;rs75919325;rs79624714;rs2080858;rs138092354;rs148285468;rs114455395;rs74850689;rs10463599 | NA | 0 |
| 4 | 5:108560001-108600000 | 5:108240001-108280000 | 5.29E-08 | HiC | GSE87112 | Left_Ventricle | intra | rs35812497;rs75803484;rs75073283;rs76430100;rs75782477;rs77992445;rs61701247;rs17161655;rs115979287;rs17161659;rs151072743;rs17161665;rs6863688;5:108591279:C:T;5:108591279:A:T;rs1363213;rs1862201;rs1862202;rs1862203;rs5870359 | NA | 0 |
| 4 | 5:108560001-108600000 | 5:108360001-108400000 | 9.14E-08 | HiC | GSE87112 | Left_Ventricle | intra | rs35812497;rs75803484;rs75073283;rs76430100;rs75782477;rs77992445;rs61701247;rs17161655;rs115979287;rs17161659;rs151072743;rs17161665;rs6863688;5:108591279:C:T;5:108591279:A:T;rs1363213;rs1862201;rs1862202;rs1862203;rs5870359 | NA | 0 |
| 6 | 12:80800001-80840000 | 12:78000001-78040000 | 1.94E-08 | HiC | GSE87112 | Left_Ventricle | intra | rs117203215;rs77741796 | NA | 0 |
| 6 | 12:80800001-80840000 | 12:78680001-78720000 | 6.86E-11 | HiC | GSE87112 | Left_Ventricle | intra | rs117203215;rs77741796 | NA | 0 |
| 6 | 12:80800001-80840000 | 12:78720001-78760000 | 2.87E-09 | HiC | GSE87112 | Left_Ventricle | intra | rs117203215;rs77741796 | NA | 0 |
| 6 | 12:80800001-80840000 | 12:79320001-79360000 | 2.29E-09 | HiC | GSE87112 | Left_Ventricle | intra | rs117203215;rs77741796 | NA | 0 |
| 6 | 12:80800001-80840000 | 12:79760001-79800000 | 2.25E-15 | HiC | GSE87112 | Left_Ventricle | intra | rs117203215;rs77741796 | NA | 0 |
| 6 | 12:80640001-80680000 | 12:79920001-79960000 | 8.93E-10 | HiC | GSE87112 | Left_Ventricle | intra | rs201272756;rs202070984 | NA | 0 |
| 6 | 12:80800001-80840000 | 12:80720001-80760000 | 8.64E-07 | HiC | GSE87112 | Left_Ventricle | intra | rs117203215;rs77741796 | NA | 0 |
| 6 | 12:80800001-80840000 | 12:80760001-80800000 | 7.31E-08 | HiC | GSE87112 | Left_Ventricle | intra | rs117203215;rs77741796 | ENSG00000139304 | 0 |
| 7 | 12:127760001-127800000 | 12:125760001-125800000 | 5.97E-09 | HiC | GSE87112 | Left_Ventricle | intra | rs1810088;rs367839627;rs146043604;rs143439170;rs10773382;rs10847307;rs35154640;rs1552386;rs7314186;rs1979065;rs5801722;rs10744302;12:127775513:A:C;rs10734938;rs35024927;rs10773387;rs978813;rs2348321;rs10847321;rs10847323;rs6489163 | NA | 0 |
| 7 | 12:127760001-127800000 | 12:125960001-126000000 | 1.65E-11 | HiC | GSE87112 | Left_Ventricle | intra | rs1810088;rs367839627;rs146043604;rs143439170;rs10773382;rs10847307;rs35154640;rs1552386;rs7314186;rs1979065;rs5801722;rs10744302;12:127775513:A:C;rs10734938;rs35024927;rs10773387;rs978813;rs2348321;rs10847321;rs10847323;rs6489163 | NA | 0 |
| 7 | 12:127760001-127800000 | 12:126040001-126080000 | 3.76E-17 | HiC | GSE87112 | Left_Ventricle | intra | rs1810088;rs367839627;rs146043604;rs143439170;rs10773382;rs10847307;rs35154640;rs1552386;rs7314186;rs1979065;rs5801722;rs10744302;12:127775513:A:C;rs10734938;rs35024927;rs10773387;rs978813;rs2348321;rs10847321;rs10847323;rs6489163 | NA | 0 |
| 7 | 12:127760001-127800000 | 12:126080001-126120000 | 1.79E-07 | HiC | GSE87112 | Left_Ventricle | intra | rs1810088;rs367839627;rs146043604;rs143439170;rs10773382;rs10847307;rs35154640;rs1552386;rs7314186;rs1979065;rs5801722;rs10744302;12:127775513:A:C;rs10734938;rs35024927;rs10773387;rs978813;rs2348321;rs10847321;rs10847323;rs6489163 | NA | 0 |
| 7 | 12:127760001-127800000 | 12:126120001-126160000 | 3.61E-11 | HiC | GSE87112 | Left_Ventricle | intra | rs1810088;rs367839627;rs146043604;rs143439170;rs10773382;rs10847307;rs35154640;rs1552386;rs7314186;rs1979065;rs5801722;rs10744302;12:127775513:A:C;rs10734938;rs35024927;rs10773387;rs978813;rs2348321;rs10847321;rs10847323;rs6489163 | NA | 0 |
| 7 | 12:127760001-127800000 | 12:126280001-126320000 | 2.08E-16 | HiC | GSE87112 | Left_Ventricle | intra | rs1810088;rs367839627;rs146043604;rs143439170;rs10773382;rs10847307;rs35154640;rs1552386;rs7314186;rs1979065;rs5801722;rs10744302;12:127775513:A:C;rs10734938;rs35024927;rs10773387;rs978813;rs2348321;rs10847321;rs10847323;rs6489163 | NA | 0 |
| 7 | 12:127760001-127800000 | 12:126440001-126480000 | 2.91E-11 | HiC | GSE87112 | Left_Ventricle | intra | rs1810088;rs367839627;rs146043604;rs143439170;rs10773382;rs10847307;rs35154640;rs1552386;rs7314186;rs1979065;rs5801722;rs10744302;12:127775513:A:C;rs10734938;rs35024927;rs10773387;rs978813;rs2348321;rs10847321;rs10847323;rs6489163 | NA | 0 |
| 7 | 12:127760001-127800000 | 12:126680001-126720000 | 1.97E-07 | HiC | GSE87112 | Left_Ventricle | intra | rs1810088;rs367839627;rs146043604;rs143439170;rs10773382;rs10847307;rs35154640;rs1552386;rs7314186;rs1979065;rs5801722;rs10744302;12:127775513:A:C;rs10734938;rs35024927;rs10773387;rs978813;rs2348321;rs10847321;rs10847323;rs6489163 | NA | 0 |
| 7 | 12:127760001-127800000 | 12:126800001-126840000 | 5.94E-08 | HiC | GSE87112 | Left_Ventricle | intra | rs1810088;rs367839627;rs146043604;rs143439170;rs10773382;rs10847307;rs35154640;rs1552386;rs7314186;rs1979065;rs5801722;rs10744302;12:127775513:A:C;rs10734938;rs35024927;rs10773387;rs978813;rs2348321;rs10847321;rs10847323;rs6489163 | NA | 0 |
| 7 | 12:127760001-127800000 | 12:127240001-127280000 | 3.41E-07 | HiC | GSE87112 | Left_Ventricle | intra | rs1810088;rs367839627;rs146043604;rs143439170;rs10773382;rs10847307;rs35154640;rs1552386;rs7314186;rs1979065;rs5801722;rs10744302;12:127775513:A:C;rs10734938;rs35024927;rs10773387;rs978813;rs2348321;rs10847321;rs10847323;rs6489163 | NA | 0 |
| 7 | 12:127760001-127800000 | 12:127360001-127400000 | 9.64E-08 | HiC | GSE87112 | Left_Ventricle | intra | rs1810088;rs367839627;rs146043604;rs143439170;rs10773382;rs10847307;rs35154640;rs1552386;rs7314186;rs1979065;rs5801722;rs10744302;12:127775513:A:C;rs10734938;rs35024927;rs10773387;rs978813;rs2348321;rs10847321;rs10847323;rs6489163 | NA | 0 |
| 7 | 12:127760001-127800000 | 12:127440001-127480000 | 2.06E-09 | HiC | GSE87112 | Left_Ventricle | intra | rs1810088;rs367839627;rs146043604;rs143439170;rs10773382;rs10847307;rs35154640;rs1552386;rs7314186;rs1979065;rs5801722;rs10744302;12:127775513:A:C;rs10734938;rs35024927;rs10773387;rs978813;rs2348321;rs10847321;rs10847323;rs6489163 | NA | 0 |
| 7 | 12:127760001-127800000 | 12:127480001-127520000 | 4.46E-10 | HiC | GSE87112 | Left_Ventricle | intra | rs1810088;rs367839627;rs146043604;rs143439170;rs10773382;rs10847307;rs35154640;rs1552386;rs7314186;rs1979065;rs5801722;rs10744302;12:127775513:A:C;rs10734938;rs35024927;rs10773387;rs978813;rs2348321;rs10847321;rs10847323;rs6489163 | NA | 0 |
| 7 | 12:127760001-127800000 | 12:127560001-127600000 | 1.12E-17 | HiC | GSE87112 | Left_Ventricle | intra | rs1810088;rs367839627;rs146043604;rs143439170;rs10773382;rs10847307;rs35154640;rs1552386;rs7314186;rs1979065;rs5801722;rs10744302;12:127775513:A:C;rs10734938;rs35024927;rs10773387;rs978813;rs2348321;rs10847321;rs10847323;rs6489163 | NA | 0 |
| 7 | 12:127760001-127800000 | 12:127600001-127640000 | 6.05E-14 | HiC | GSE87112 | Left_Ventricle | intra | rs1810088;rs367839627;rs146043604;rs143439170;rs10773382;rs10847307;rs35154640;rs1552386;rs7314186;rs1979065;rs5801722;rs10744302;12:127775513:A:C;rs10734938;rs35024927;rs10773387;rs978813;rs2348321;rs10847321;rs10847323;rs6489163 | NA | 0 |
| 7 | 12:127760001-127800000 | 12:127680001-127720000 | 9.15E-08 | HiC | GSE87112 | Left_Ventricle | intra | rs1810088;rs367839627;rs146043604;rs143439170;rs10773382;rs10847307;rs35154640;rs1552386;rs7314186;rs1979065;rs5801722;rs10744302;12:127775513:A:C;rs10734938;rs35024927;rs10773387;rs978813;rs2348321;rs10847321;rs10847323;rs6489163 | NA | 0 |
| 7 | 12:127760001-127800000 | 12:127720001-127760000 | 1.18E-28 | HiC | GSE87112 | Left_Ventricle | intra | rs1810088;rs367839627;rs146043604;rs143439170;rs10773382;rs10847307;rs35154640;rs1552386;rs7314186;rs1979065;rs5801722;rs10744302;12:127775513:A:C;rs10734938;rs35024927;rs10773387;rs978813;rs2348321;rs10847321;rs10847323;rs6489163 | NA | 0 |
| 2 | 3:96600001-96640000 | 3:96680001-96720000 | 5.74E-11 | HiC | GSE87112 | Liver | intra | rs62262952;rs62262953;rs116253310;rs76857958;rs111706803;rs62262955;rs62262957;rs62262958;rs62263676;rs187619159;rs62263677;rs62263678;rs574559070;rs534937763;rs62263680;rs62263682;rs62263684;rs16836982;rs16836986;rs112527011;rs62263685;rs62263686 | NA | 0 |
| 2 | 3:96600001-96640000 | 3:97280001-97320000 | 9.48E-07 | HiC | GSE87112 | Liver | intra | rs62262952;rs62262953;rs116253310;rs76857958;rs111706803;rs62262955;rs62262957;rs62262958;rs62263676;rs187619159;rs62263677;rs62263678;rs574559070;rs534937763;rs62263680;rs62263682;rs62263684;rs16836982;rs16836986;rs112527011;rs62263685;rs62263686 | NA | 0 |
| 2 | 3:96600001-96640000 | 3:97360001-97400000 | 3.01E-08 | HiC | GSE87112 | Liver | intra | rs62262952;rs62262953;rs116253310;rs76857958;rs111706803;rs62262955;rs62262957;rs62262958;rs62263676;rs187619159;rs62263677;rs62263678;rs574559070;rs534937763;rs62263680;rs62263682;rs62263684;rs16836982;rs16836986;rs112527011;rs62263685;rs62263686 | NA | 0 |
| 2 | 3:96680001-96720000 | 3:97120001-97160000 | 6.65E-10 | HiC | GSE87112 | Liver | intra | rs62263705;rs62263706;rs6762348;rs62263709;rs137983052;rs62263734;rs2856474;rs2612274;rs553308194 | NA | 0 |
| 2 | 3:96680001-96720000 | 3:97200001-97240000 | 2.69E-07 | HiC | GSE87112 | Liver | intra | rs62263705;rs62263706;rs6762348;rs62263709;rs137983052;rs62263734;rs2856474;rs2612274;rs553308194 | NA | 0 |
| 2 | 3:96680001-96720000 | 3:97280001-97320000 | 2.67E-13 | HiC | GSE87112 | Liver | intra | rs62263705;rs62263706;rs6762348;rs62263709;rs137983052;rs62263734;rs2856474;rs2612274;rs553308194 | NA | 0 |
| 2 | 3:96680001-96720000 | 3:97360001-97400000 | 1.34E-07 | HiC | GSE87112 | Liver | intra | rs62263705;rs62263706;rs6762348;rs62263709;rs137983052;rs62263734;rs2856474;rs2612274;rs553308194 | NA | 0 |
| 3 | 4:171440001-171480000 | 4:171520001-171560000 | 1.82E-08 | HiC | GSE87112 | Liver | intra | rs147274196 | NA | 0 |
| 6 | 12:80800001-80840000 | 12:80920001-80960000 | 1.32E-10 | HiC | GSE87112 | Liver | intra | rs117203215;rs77741796 | NA | 0 |
| 6 | 12:80800001-80840000 | 12:81000001-81040000 | 9.98E-13 | HiC | GSE87112 | Liver | intra | rs117203215;rs77741796 | NA | 0 |
| 6 | 12:80800001-80840000 | 12:81600001-81640000 | 1.76E-07 | HiC | GSE87112 | Liver | intra | rs117203215;rs77741796 | NA | 0 |
| 6 | 12:80800001-80840000 | 12:81640001-81680000 | 6.77E-10 | HiC | GSE87112 | Liver | intra | rs117203215;rs77741796 | NA | 0 |
| 7 | 12:127760001-127800000 | 12:127800001-127840000 | 3.52E-11 | HiC | GSE87112 | Liver | intra | rs1810088;rs367839627;rs146043604;rs143439170;rs10773382;rs10847307;rs35154640;rs1552386;rs7314186;rs1979065;rs5801722;rs10744302;12:127775513:A:C;rs10734938;rs35024927;rs10773387;rs978813;rs2348321;rs10847321;rs10847323;rs6489163 | NA | 0 |
| 2 | 3:96600001-96640000 | 3:93880001-93920000 | 3.57E-08 | HiC | GSE87112 | Liver | intra | rs62262952;rs62262953;rs116253310;rs76857958;rs111706803;rs62262955;rs62262957;rs62262958;rs62263676;rs187619159;rs62263677;rs62263678;rs574559070;rs534937763;rs62263680;rs62263682;rs62263684;rs16836982;rs16836986;rs112527011;rs62263685;rs62263686 | NA | 0 |
| 2 | 3:96680001-96720000 | 3:93880001-93920000 | 4.75E-07 | HiC | GSE87112 | Liver | intra | rs62263705;rs62263706;rs6762348;rs62263709;rs137983052;rs62263734;rs2856474;rs2612274;rs553308194 | NA | 0 |
| 2 | 3:96520001-96560000 | 3:94000001-94040000 | 2.15E-10 | HiC | GSE87112 | Liver | intra | rs62262901;rs62262902;rs62262903;rs62262930;rs151165295;rs62262931;rs62262933;rs62262934;rs74641574;rs79645223;rs2318156;rs2318155;rs2318154 | NA | 0 |
| 2 | 3:96680001-96720000 | 3:94000001-94040000 | 3.09E-10 | HiC | GSE87112 | Liver | intra | rs62263705;rs62263706;rs6762348;rs62263709;rs137983052;rs62263734;rs2856474;rs2612274;rs553308194 | NA | 0 |
| 2 | 3:96520001-96560000 | 3:94120001-94160000 | 1.06E-10 | HiC | GSE87112 | Liver | intra | rs62262901;rs62262902;rs62262903;rs62262930;rs151165295;rs62262931;rs62262933;rs62262934;rs74641574;rs79645223;rs2318156;rs2318155;rs2318154 | NA | 0 |
| 2 | 3:96520001-96560000 | 3:94640001-94680000 | 3.50E-09 | HiC | GSE87112 | Liver | intra | rs62262901;rs62262902;rs62262903;rs62262930;rs151165295;rs62262931;rs62262933;rs62262934;rs74641574;rs79645223;rs2318156;rs2318155;rs2318154 | NA | 0 |
| 2 | 3:96600001-96640000 | 3:94640001-94680000 | 5.15E-07 | HiC | GSE87112 | Liver | intra | rs62262952;rs62262953;rs116253310;rs76857958;rs111706803;rs62262955;rs62262957;rs62262958;rs62263676;rs187619159;rs62263677;rs62263678;rs574559070;rs534937763;rs62263680;rs62263682;rs62263684;rs16836982;rs16836986;rs112527011;rs62263685;rs62263686 | NA | 0 |
| 2 | 3:96680001-96720000 | 3:94680001-94720000 | 3.60E-07 | HiC | GSE87112 | Liver | intra | rs62263705;rs62263706;rs6762348;rs62263709;rs137983052;rs62263734;rs2856474;rs2612274;rs553308194 | NA | 0 |
| 2 | 3:96680001-96720000 | 3:96600001-96640000 | 5.74E-11 | HiC | GSE87112 | Liver | intra | rs62263705;rs62263706;rs6762348;rs62263709;rs137983052;rs62263734;rs2856474;rs2612274;rs553308194 | NA | 0 |
| 6 | 12:80800001-80840000 | 12:80680001-80720000 | 1.73E-08 | HiC | GSE87112 | Liver | intra | rs117203215;rs77741796 | NA | 0 |
| 6 | 12:80800001-80840000 | 12:80720001-80760000 | 5.10E-08 | HiC | GSE87112 | Liver | intra | rs117203215;rs77741796 | NA | 0 |
| 7 | 12:127760001-127800000 | 12:127360001-127400000 | 1.52E-07 | HiC | GSE87112 | Liver | intra | rs1810088;rs367839627;rs146043604;rs143439170;rs10773382;rs10847307;rs35154640;rs1552386;rs7314186;rs1979065;rs5801722;rs10744302;12:127775513:A:C;rs10734938;rs35024927;rs10773387;rs978813;rs2348321;rs10847321;rs10847323;rs6489163 | NA | 0 |
| 7 | 12:127760001-127800000 | 12:127560001-127600000 | 1.40E-20 | HiC | GSE87112 | Liver | intra | rs1810088;rs367839627;rs146043604;rs143439170;rs10773382;rs10847307;rs35154640;rs1552386;rs7314186;rs1979065;rs5801722;rs10744302;12:127775513:A:C;rs10734938;rs35024927;rs10773387;rs978813;rs2348321;rs10847321;rs10847323;rs6489163 | NA | 0 |
| 7 | 12:127760001-127800000 | 12:127720001-127760000 | 5.47E-07 | HiC | GSE87112 | Liver | intra | rs1810088;rs367839627;rs146043604;rs143439170;rs10773382;rs10847307;rs35154640;rs1552386;rs7314186;rs1979065;rs5801722;rs10744302;12:127775513:A:C;rs10734938;rs35024927;rs10773387;rs978813;rs2348321;rs10847321;rs10847323;rs6489163 | NA | 0 |
| 7 | 12:127760001-127800000 | 12:127800001-127840000 | 2.13E-08 | HiC | GSE87112 | Lung | intra | rs1810088;rs367839627;rs146043604;rs143439170;rs10773382;rs10847307;rs35154640;rs1552386;rs7314186;rs1979065;rs5801722;rs10744302;12:127775513:A:C;rs10734938;rs35024927;rs10773387;rs978813;rs2348321;rs10847321;rs10847323;rs6489163 | NA | 0 |
| 7 | 12:127760001-127800000 | 12:127800001-127840000 | 1.30E-16 | HiC | GSE87112 | Pancreas | intra | rs1810088;rs367839627;rs146043604;rs143439170;rs10773382;rs10847307;rs35154640;rs1552386;rs7314186;rs1979065;rs5801722;rs10744302;12:127775513:A:C;rs10734938;rs35024927;rs10773387;rs978813;rs2348321;rs10847321;rs10847323;rs6489163 | NA | 0 |
| 7 | 12:127760001-127800000 | 12:127720001-127760000 | 4.51E-09 | HiC | GSE87112 | Pancreas | intra | rs1810088;rs367839627;rs146043604;rs143439170;rs10773382;rs10847307;rs35154640;rs1552386;rs7314186;rs1979065;rs5801722;rs10744302;12:127775513:A:C;rs10734938;rs35024927;rs10773387;rs978813;rs2348321;rs10847321;rs10847323;rs6489163 | NA | 0 |
| 7 | 12:127760001-127800000 | 12:127800001-127840000 | 1.74E-11 | HiC | GSE87112 | Right_Ventricle | intra | rs1810088;rs367839627;rs146043604;rs143439170;rs10773382;rs10847307;rs35154640;rs1552386;rs7314186;rs1979065;rs5801722;rs10744302;12:127775513:A:C;rs10734938;rs35024927;rs10773387;rs978813;rs2348321;rs10847321;rs10847323;rs6489163 | NA | 0 |
| 2 | 3:96520001-96560000 | 3:93880001-93920000 | 3.61E-07 | HiC | GSE87112 | Right_Ventricle | intra | rs62262901;rs62262902;rs62262903;rs62262930;rs151165295;rs62262931;rs62262933;rs62262934;rs74641574;rs79645223;rs2318156;rs2318155;rs2318154 | NA | 0 |
| 2 | 3:96520001-96560000 | 3:94000001-94040000 | 6.29E-07 | HiC | GSE87112 | Right_Ventricle | intra | rs62262901;rs62262902;rs62262903;rs62262930;rs151165295;rs62262931;rs62262933;rs62262934;rs74641574;rs79645223;rs2318156;rs2318155;rs2318154 | NA | 0 |
| 5 | 11:2520001-2560000 | 11:2880001-2920000 | 5.75E-15 | HiC | GSE87112 | Spleen | intra | rs111815403 | ENSG00000129757 | 0 |
| 5 | 11:2520001-2560000 | 11:2960001-3000000 | 4.89E-08 | HiC | GSE87112 | Spleen | intra | rs111815403 | NA | 0 |
| 7 | 12:127760001-127800000 | 12:127800001-127840000 | 3.04E-19 | HiC | GSE87112 | Spleen | intra | rs1810088;rs367839627;rs146043604;rs143439170;rs10773382;rs10847307;rs35154640;rs1552386;rs7314186;rs1979065;rs5801722;rs10744302;12:127775513:A:C;rs10734938;rs35024927;rs10773387;rs978813;rs2348321;rs10847321;rs10847323;rs6489163 | NA | 0 |
| 7 | 12:127760001-127800000 | 12:127840001-127880000 | 8.42E-07 | HiC | GSE87112 | Spleen | intra | rs1810088;rs367839627;rs146043604;rs143439170;rs10773382;rs10847307;rs35154640;rs1552386;rs7314186;rs1979065;rs5801722;rs10744302;12:127775513:A:C;rs10734938;rs35024927;rs10773387;rs978813;rs2348321;rs10847321;rs10847323;rs6489163 | NA | 0 |
| 4 | 5:108560001-108600000 | 5:107960001-108000000 | 2.57E-08 | HiC | GSE87112 | Spleen | intra | rs35812497;rs75803484;rs75073283;rs76430100;rs75782477;rs77992445;rs61701247;rs17161655;rs115979287;rs17161659;rs151072743;rs17161665;rs6863688;5:108591279:C:T;5:108591279:A:T;rs1363213;rs1862201;rs1862202;rs1862203;rs5870359 | NA | 0 |
| 5 | 11:2520001-2560000 | 11:2240001-2280000 | 1.11E-07 | HiC | GSE87112 | Spleen | intra | rs111815403 | NA | 0 |
| 7 | 12:127760001-127800000 | 12:127720001-127760000 | 1.67E-13 | HiC | GSE87112 | Spleen | intra | rs1810088;rs367839627;rs146043604;rs143439170;rs10773382;rs10847307;rs35154640;rs1552386;rs7314186;rs1979065;rs5801722;rs10744302;12:127775513:A:C;rs10734938;rs35024927;rs10773387;rs978813;rs2348321;rs10847321;rs10847323;rs6489163 | NA | 0 |
| 6 | 12:80800001-80840000 | 12:81240001-81280000 | 1.44E-08 | HiC | GSE87112 | GM12878 | intra | rs117203215;rs77741796 | NA | 0 |
| 6 | 12:80800001-80840000 | 12:81480001-81520000 | 4.76E-08 | HiC | GSE87112 | GM12878 | intra | rs117203215;rs77741796 | NA | 0 |
| 2 | 3:96520001-96560000 | 3:94000001-94040000 | 1.96E-12 | HiC | GSE87112 | GM12878 | intra | rs62262901;rs62262902;rs62262903;rs62262930;rs151165295;rs62262931;rs62262933;rs62262934;rs74641574;rs79645223;rs2318156;rs2318155;rs2318154 | NA | 0 |
| 2 | 3:96520001-96560000 | 3:94120001-94160000 | 7.98E-07 | HiC | GSE87112 | GM12878 | intra | rs62262901;rs62262902;rs62262903;rs62262930;rs151165295;rs62262931;rs62262933;rs62262934;rs74641574;rs79645223;rs2318156;rs2318155;rs2318154 | NA | 0 |
| 4 | 5:108560001-108600000 | 5:107960001-108000000 | 8.40E-09 | HiC | GSE87112 | GM12878 | intra | rs35812497;rs75803484;rs75073283;rs76430100;rs75782477;rs77992445;rs61701247;rs17161655;rs115979287;rs17161659;rs151072743;rs17161665;rs6863688;5:108591279:C:T;5:108591279:A:T;rs1363213;rs1862201;rs1862202;rs1862203;rs5870359 | NA | 0 |
| 4 | 5:108600001-108640000 | 5:107960001-108000000 | 2.06E-10 | HiC | GSE87112 | GM12878 | intra | rs11744353;rs79776200;rs76069623;rs76043709;rs78729571;rs36123121;rs11748450;rs80234158;rs78146542;rs185072862;rs137867205;rs189006725;rs4388251;rs4438924;rs4541698;rs4388252;rs4392675;rs200252534;rs145520315;rs147699420;rs77803982;rs79469094;rs75077625;rs74379173;rs78664536;rs549536839;rs570631944;rs78784808;rs78340649;rs201569786;rs75649455;rs78814720;rs77561644;rs76565008;rs74436342;rs1592807;rs1592810;rs1592811;rs7700458;rs6594365;rs6863893;rs140163909;rs146837743;rs7705919;rs201046346;rs202152005;rs199869120;rs111726541;rs373673987;rs113026830;rs148211606;rs201619508;rs201169921;rs146644914;rs116649742;rs79893293;rs57629433;rs60335509;rs58453996;rs59466242;rs60878839;rs11743452;rs1833567;rs1833568;rs1833569;rs7721272;rs7721427;rs7721730;rs7721870;rs7722100;rs7704359;rs1895200;rs1895201;rs77974856;rs60905342;rs11746207;rs11739672;rs11749718;rs78518991;rs76243602;rs76383235;rs11741980;rs35243960;rs116462975;rs139801813;rs113103584;rs74727230;rs75919325;rs79624714;rs2080858;rs138092354;rs148285468;rs114455395;rs74850689;rs10463599 | NA | 0 |
| 1 | 3:37520001-37560000 | 3:37720001-37760000 | 2.38E-12 | HiC | GSE87112 | IMR90 | intra | rs113897538;rs2507941;rs17814364;rs112038297;rs78544469;rs77422813;rs17228684;rs928799;rs75425697;rs5848026;rs17814903;rs12054441 | NA | 0 |
| 1 | 3:37520001-37560000 | 3:37760001-37800000 | 3.15E-16 | HiC | GSE87112 | IMR90 | intra | rs113897538;rs2507941;rs17814364;rs112038297;rs78544469;rs77422813;rs17228684;rs928799;rs75425697;rs5848026;rs17814903;rs12054441 | NA | 0 |
| 1 | 3:37520001-37560000 | 3:37800001-37840000 | 2.09E-16 | HiC | GSE87112 | IMR90 | intra | rs113897538;rs2507941;rs17814364;rs112038297;rs78544469;rs77422813;rs17228684;rs928799;rs75425697;rs5848026;rs17814903;rs12054441 | NA | 0 |
| 1 | 3:37560001-37600000 | 3:37640001-37680000 | 8.54E-18 | HiC | GSE87112 | IMR90 | intra | rs74985154;rs3733138;rs3733140;rs75619156;rs17229924;rs2162356;rs74786716;rs77820597;rs112585731;rs915631;rs79149273;rs17230261;rs199607377;rs2162355;rs76952775;rs75469174;rs78210776;rs77162103;rs75217006;rs78292414 | NA | 0 |
| 1 | 3:37560001-37600000 | 3:37680001-37720000 | 4.97E-10 | HiC | GSE87112 | IMR90 | intra | rs74985154;rs3733138;rs3733140;rs75619156;rs17229924;rs2162356;rs74786716;rs77820597;rs112585731;rs915631;rs79149273;rs17230261;rs199607377;rs2162355;rs76952775;rs75469174;rs78210776;rs77162103;rs75217006;rs78292414 | NA | 0 |
| 1 | 3:37560001-37600000 | 3:37760001-37800000 | 1.93E-27 | HiC | GSE87112 | IMR90 | intra | rs74985154;rs3733138;rs3733140;rs75619156;rs17229924;rs2162356;rs74786716;rs77820597;rs112585731;rs915631;rs79149273;rs17230261;rs199607377;rs2162355;rs76952775;rs75469174;rs78210776;rs77162103;rs75217006;rs78292414 | NA | 0 |
| 1 | 3:37560001-37600000 | 3:37800001-37840000 | 6.83E-16 | HiC | GSE87112 | IMR90 | intra | rs74985154;rs3733138;rs3733140;rs75619156;rs17229924;rs2162356;rs74786716;rs77820597;rs112585731;rs915631;rs79149273;rs17230261;rs199607377;rs2162355;rs76952775;rs75469174;rs78210776;rs77162103;rs75217006;rs78292414 | NA | 0 |
| 1 | 3:37560001-37600000 | 3:37840001-37880000 | 7.05E-08 | HiC | GSE87112 | IMR90 | intra | rs74985154;rs3733138;rs3733140;rs75619156;rs17229924;rs2162356;rs74786716;rs77820597;rs112585731;rs915631;rs79149273;rs17230261;rs199607377;rs2162355;rs76952775;rs75469174;rs78210776;rs77162103;rs75217006;rs78292414 | NA | 0 |
| 1 | 3:37560001-37600000 | 3:38680001-38720000 | 1.18E-07 | HiC | GSE87112 | IMR90 | intra | rs74985154;rs3733138;rs3733140;rs75619156;rs17229924;rs2162356;rs74786716;rs77820597;rs112585731;rs915631;rs79149273;rs17230261;rs199607377;rs2162355;rs76952775;rs75469174;rs78210776;rs77162103;rs75217006;rs78292414 | ENSG00000183873 | 1 |
| 2 | 3:96680001-96720000 | 3:97720001-97760000 | 6.10E-09 | HiC | GSE87112 | IMR90 | intra | rs62263705;rs62263706;rs6762348;rs62263709;rs137983052;rs62263734;rs2856474;rs2612274;rs553308194 | NA | 0 |
| 3 | 4:171440001-171480000 | 4:172520001-172560000 | 1.06E-10 | HiC | GSE87112 | IMR90 | intra | rs147274196 | NA | 0 |
| 3 | 4:171440001-171480000 | 4:172600001-172640000 | 1.32E-07 | HiC | GSE87112 | IMR90 | intra | rs147274196 | NA | 0 |
| 3 | 4:171440001-171480000 | 4:172840001-172880000 | 2.85E-11 | HiC | GSE87112 | IMR90 | intra | rs147274196 | NA | 0 |
| 3 | 4:171440001-171480000 | 4:172920001-172960000 | 3.64E-09 | HiC | GSE87112 | IMR90 | intra | rs147274196 | NA | 0 |
| 3 | 4:171440001-171480000 | 4:173040001-173080000 | 1.08E-08 | HiC | GSE87112 | IMR90 | intra | rs147274196 | NA | 0 |
| 3 | 4:171440001-171480000 | 4:173080001-173120000 | 5.79E-11 | HiC | GSE87112 | IMR90 | intra | rs147274196 | NA | 0 |
| 3 | 4:171440001-171480000 | 4:173440001-173480000 | 2.16E-11 | HiC | GSE87112 | IMR90 | intra | rs147274196 | NA | 0 |
| 3 | 4:171440001-171480000 | 4:173560001-173600000 | 3.85E-08 | HiC | GSE87112 | IMR90 | intra | rs147274196 | NA | 0 |
| 3 | 4:171440001-171480000 | 4:173600001-173640000 | 2.92E-08 | HiC | GSE87112 | IMR90 | intra | rs147274196 | NA | 0 |
| 3 | 4:171440001-171480000 | 4:173680001-173720000 | 1.50E-09 | HiC | GSE87112 | IMR90 | intra | rs147274196 | NA | 0 |
| 3 | 4:171440001-171480000 | 4:173760001-173800000 | 6.10E-11 | HiC | GSE87112 | IMR90 | intra | rs147274196 | NA | 0 |
| 3 | 4:171440001-171480000 | 4:173880001-173920000 | 3.50E-07 | HiC | GSE87112 | IMR90 | intra | rs147274196 | NA | 0 |
| 3 | 4:171440001-171480000 | 4:174200001-174240000 | 4.96E-09 | HiC | GSE87112 | IMR90 | intra | rs147274196 | NA | 0 |
| 3 | 4:171480001-171520000 | 4:171520001-171560000 | 1.02E-07 | HiC | GSE87112 | IMR90 | intra | rs148420952;rs76413646 | NA | 0 |
| 3 | 4:171480001-171520000 | 4:172080001-172120000 | 3.25E-07 | HiC | GSE87112 | IMR90 | intra | rs148420952;rs76413646 | NA | 0 |
| 3 | 4:171480001-171520000 | 4:172440001-172480000 | 1.90E-10 | HiC | GSE87112 | IMR90 | intra | rs148420952;rs76413646 | NA | 0 |
| 3 | 4:171480001-171520000 | 4:172520001-172560000 | 3.05E-09 | HiC | GSE87112 | IMR90 | intra | rs148420952;rs76413646 | NA | 0 |
| 3 | 4:171480001-171520000 | 4:172600001-172640000 | 2.60E-08 | HiC | GSE87112 | IMR90 | intra | rs148420952;rs76413646 | NA | 0 |
| 3 | 4:171480001-171520000 | 4:172840001-172880000 | 7.91E-07 | HiC | GSE87112 | IMR90 | intra | rs148420952;rs76413646 | NA | 0 |
| 3 | 4:171480001-171520000 | 4:172880001-172920000 | 8.95E-12 | HiC | GSE87112 | IMR90 | intra | rs148420952;rs76413646 | NA | 0 |
| 3 | 4:171480001-171520000 | 4:172920001-172960000 | 6.41E-08 | HiC | GSE87112 | IMR90 | intra | rs148420952;rs76413646 | NA | 0 |
| 3 | 4:171480001-171520000 | 4:173040001-173080000 | 5.92E-08 | HiC | GSE87112 | IMR90 | intra | rs148420952;rs76413646 | NA | 0 |
| 3 | 4:171480001-171520000 | 4:173080001-173120000 | 2.50E-07 | HiC | GSE87112 | IMR90 | intra | rs148420952;rs76413646 | NA | 0 |
| 3 | 4:171480001-171520000 | 4:173160001-173200000 | 2.40E-07 | HiC | GSE87112 | IMR90 | intra | rs148420952;rs76413646 | NA | 0 |
| 3 | 4:171480001-171520000 | 4:173240001-173280000 | 3.41E-09 | HiC | GSE87112 | IMR90 | intra | rs148420952;rs76413646 | NA | 0 |
| 3 | 4:171480001-171520000 | 4:173280001-173320000 | 2.10E-09 | HiC | GSE87112 | IMR90 | intra | rs148420952;rs76413646 | NA | 0 |
| 3 | 4:171480001-171520000 | 4:173360001-173400000 | 8.83E-09 | HiC | GSE87112 | IMR90 | intra | rs148420952;rs76413646 | NA | 0 |
| 3 | 4:171480001-171520000 | 4:173520001-173560000 | 5.30E-11 | HiC | GSE87112 | IMR90 | intra | rs148420952;rs76413646 | NA | 0 |
| 3 | 4:171480001-171520000 | 4:173560001-173600000 | 3.69E-11 | HiC | GSE87112 | IMR90 | intra | rs148420952;rs76413646 | NA | 0 |
| 3 | 4:171480001-171520000 | 4:173680001-173720000 | 6.28E-07 | HiC | GSE87112 | IMR90 | intra | rs148420952;rs76413646 | NA | 0 |
| 3 | 4:171480001-171520000 | 4:173760001-173800000 | 2.37E-11 | HiC | GSE87112 | IMR90 | intra | rs148420952;rs76413646 | NA | 0 |
| 3 | 4:171480001-171520000 | 4:173880001-173920000 | 1.03E-10 | HiC | GSE87112 | IMR90 | intra | rs148420952;rs76413646 | NA | 0 |
| 3 | 4:171480001-171520000 | 4:173960001-174000000 | 1.99E-15 | HiC | GSE87112 | IMR90 | intra | rs148420952;rs76413646 | NA | 0 |
| 3 | 4:171480001-171520000 | 4:174480001-174520000 | 4.11E-08 | HiC | GSE87112 | IMR90 | intra | rs148420952;rs76413646 | NA | 0 |
| 4 | 5:108560001-108600000 | 5:108800001-108840000 | 8.09E-15 | HiC | GSE87112 | IMR90 | intra | rs35812497;rs75803484;rs75073283;rs76430100;rs75782477;rs77992445;rs61701247;rs17161655;rs115979287;rs17161659;rs151072743;rs17161665;rs6863688;5:108591279:C:T;5:108591279:A:T;rs1363213;rs1862201;rs1862202;rs1862203;rs5870359 | NA | 0 |
| 4 | 5:108560001-108600000 | 5:109920001-109960000 | 4.10E-10 | HiC | GSE87112 | IMR90 | intra | rs35812497;rs75803484;rs75073283;rs76430100;rs75782477;rs77992445;rs61701247;rs17161655;rs115979287;rs17161659;rs151072743;rs17161665;rs6863688;5:108591279:C:T;5:108591279:A:T;rs1363213;rs1862201;rs1862202;rs1862203;rs5870359 | NA | 0 |
| 4 | 5:108560001-108600000 | 5:109960001-110000000 | 8.25E-15 | HiC | GSE87112 | IMR90 | intra | rs35812497;rs75803484;rs75073283;rs76430100;rs75782477;rs77992445;rs61701247;rs17161655;rs115979287;rs17161659;rs151072743;rs17161665;rs6863688;5:108591279:C:T;5:108591279:A:T;rs1363213;rs1862201;rs1862202;rs1862203;rs5870359 | NA | 0 |
| 4 | 5:108560001-108600000 | 5:110040001-110080000 | 3.42E-07 | HiC | GSE87112 | IMR90 | intra | rs35812497;rs75803484;rs75073283;rs76430100;rs75782477;rs77992445;rs61701247;rs17161655;rs115979287;rs17161659;rs151072743;rs17161665;rs6863688;5:108591279:C:T;5:108591279:A:T;rs1363213;rs1862201;rs1862202;rs1862203;rs5870359 | ENSG00000186952:ENSG00000164209 | 0 |
| 4 | 5:108600001-108640000 | 5:108800001-108840000 | 5.96E-08 | HiC | GSE87112 | IMR90 | intra | rs11744353;rs79776200;rs76069623;rs76043709;rs78729571;rs36123121;rs11748450;rs80234158;rs78146542;rs185072862;rs137867205;rs189006725;rs4388251;rs4438924;rs4541698;rs4388252;rs4392675;rs200252534;rs145520315;rs147699420;rs77803982;rs79469094;rs75077625;rs74379173;rs78664536;rs549536839;rs570631944;rs78784808;rs78340649;rs201569786;rs75649455;rs78814720;rs77561644;rs76565008;rs74436342;rs1592807;rs1592810;rs1592811;rs7700458;rs6594365;rs6863893;rs140163909;rs146837743;rs7705919;rs201046346;rs202152005;rs199869120;rs111726541;rs373673987;rs113026830;rs148211606;rs201619508;rs201169921;rs146644914;rs116649742;rs79893293;rs57629433;rs60335509;rs58453996;rs59466242;rs60878839;rs11743452;rs1833567;rs1833568;rs1833569;rs7721272;rs7721427;rs7721730;rs7721870;rs7722100;rs7704359;rs1895200;rs1895201;rs77974856;rs60905342;rs11746207;rs11739672;rs11749718;rs78518991;rs76243602;rs76383235;rs11741980;rs35243960;rs116462975;rs139801813;rs113103584;rs74727230;rs75919325;rs79624714;rs2080858;rs138092354;rs148285468;rs114455395;rs74850689;rs10463599 | NA | 0 |
| 5 | 11:2520001-2560000 | 11:2640001-2680000 | 3.38E-18 | HiC | GSE87112 | IMR90 | intra | rs111815403 | NA | 0 |
| 5 | 11:2520001-2560000 | 11:2680001-2720000 | 4.39E-21 | HiC | GSE87112 | IMR90 | intra | rs111815403 | NA | 0 |
| 5 | 11:2520001-2560000 | 11:2760001-2800000 | 2.29E-28 | HiC | GSE87112 | IMR90 | intra | rs111815403 | NA | 0 |
| 5 | 11:2520001-2560000 | 11:2840001-2880000 | 1.08E-08 | HiC | GSE87112 | IMR90 | intra | rs111815403 | NA | 0 |
| 5 | 11:2520001-2560000 | 11:2880001-2920000 | 2.76E-16 | HiC | GSE87112 | IMR90 | intra | rs111815403 | ENSG00000129757 | 0 |
| 5 | 11:2520001-2560000 | 11:2960001-3000000 | 1.06E-07 | HiC | GSE87112 | IMR90 | intra | rs111815403 | NA | 0 |
| 5 | 11:2520001-2560000 | 11:3360001-3400000 | 1.01E-08 | HiC | GSE87112 | IMR90 | intra | rs111815403 | ENSG00000005801 | 1 |
| 6 | 12:80800001-80840000 | 12:80880001-80920000 | 3.23E-11 | HiC | GSE87112 | IMR90 | intra | rs117203215;rs77741796 | NA | 0 |
| 6 | 12:80800001-80840000 | 12:80920001-80960000 | 1.22E-73 | HiC | GSE87112 | IMR90 | intra | rs117203215;rs77741796 | NA | 0 |
| 6 | 12:80800001-80840000 | 12:80960001-81000000 | 3.58E-14 | HiC | GSE87112 | IMR90 | intra | rs117203215;rs77741796 | NA | 0 |
| 6 | 12:80800001-80840000 | 12:81000001-81040000 | 6.09E-76 | HiC | GSE87112 | IMR90 | intra | rs117203215;rs77741796 | NA | 0 |
| 6 | 12:80800001-80840000 | 12:81040001-81080000 | 2.51E-45 | HiC | GSE87112 | IMR90 | intra | rs117203215;rs77741796 | NA | 0 |
| 6 | 12:80800001-80840000 | 12:81080001-81120000 | 1.20E-66 | HiC | GSE87112 | IMR90 | intra | rs117203215;rs77741796 | ENSG00000111046:ENSG00000111049 | 0 |
| 6 | 12:80800001-80840000 | 12:81120001-81160000 | 7.08E-59 | HiC | GSE87112 | IMR90 | intra | rs117203215;rs77741796 | NA | 0 |
| 6 | 12:80800001-80840000 | 12:81160001-81200000 | 4.83E-48 | HiC | GSE87112 | IMR90 | intra | rs117203215;rs77741796 | NA | 0 |
| 6 | 12:80800001-80840000 | 12:81200001-81240000 | 7.39E-10 | HiC | GSE87112 | IMR90 | intra | rs117203215;rs77741796 | NA | 0 |
| 6 | 12:80800001-80840000 | 12:81240001-81280000 | 2.04E-67 | HiC | GSE87112 | IMR90 | intra | rs117203215;rs77741796 | NA | 0 |
| 6 | 12:80800001-80840000 | 12:81280001-81320000 | 1.76E-61 | HiC | GSE87112 | IMR90 | intra | rs117203215;rs77741796 | NA | 0 |
| 6 | 12:80800001-80840000 | 12:81320001-81360000 | 5.14E-31 | HiC | GSE87112 | IMR90 | intra | rs117203215;rs77741796 | ENSG00000111052:ENSG00000111058 | 0 |
| 6 | 12:80800001-80840000 | 12:81360001-81400000 | 1.41E-07 | HiC | GSE87112 | IMR90 | intra | rs117203215;rs77741796 | NA | 0 |
| 6 | 12:80800001-80840000 | 12:81400001-81440000 | 2.09E-13 | HiC | GSE87112 | IMR90 | intra | rs117203215;rs77741796 | NA | 0 |
| 6 | 12:80800001-80840000 | 12:81440001-81480000 | 1.77E-09 | HiC | GSE87112 | IMR90 | intra | rs117203215;rs77741796 | NA | 0 |
| 6 | 12:80800001-80840000 | 12:81480001-81520000 | 8.15E-18 | HiC | GSE87112 | IMR90 | intra | rs117203215;rs77741796 | NA | 0 |
| 6 | 12:80800001-80840000 | 12:81520001-81560000 | 1.94E-09 | HiC | GSE87112 | IMR90 | intra | rs117203215;rs77741796 | NA | 0 |
| 6 | 12:80800001-80840000 | 12:81600001-81640000 | 4.49E-31 | HiC | GSE87112 | IMR90 | intra | rs117203215;rs77741796 | NA | 0 |
| 6 | 12:80800001-80840000 | 12:81640001-81680000 | 2.06E-31 | HiC | GSE87112 | IMR90 | intra | rs117203215;rs77741796 | NA | 0 |
| 6 | 12:80840001-80880000 | 12:80920001-80960000 | 5.45E-08 | HiC | GSE87112 | IMR90 | intra | rs200589835 | NA | 0 |
| 6 | 12:80840001-80880000 | 12:81000001-81040000 | 1.99E-10 | HiC | GSE87112 | IMR90 | intra | rs200589835 | NA | 0 |
| 6 | 12:80840001-80880000 | 12:81080001-81120000 | 6.33E-10 | HiC | GSE87112 | IMR90 | intra | rs200589835 | ENSG00000111046:ENSG00000111049 | 0 |
| 6 | 12:80840001-80880000 | 12:81120001-81160000 | 1.30E-13 | HiC | GSE87112 | IMR90 | intra | rs200589835 | NA | 0 |
| 6 | 12:80840001-80880000 | 12:81240001-81280000 | 7.76E-11 | HiC | GSE87112 | IMR90 | intra | rs200589835 | NA | 0 |
| 7 | 12:127760001-127800000 | 12:127800001-127840000 | 5.16E-71 | HiC | GSE87112 | IMR90 | intra | rs1810088;rs367839627;rs146043604;rs143439170;rs10773382;rs10847307;rs35154640;rs1552386;rs7314186;rs1979065;rs5801722;rs10744302;12:127775513:A:C;rs10734938;rs35024927;rs10773387;rs978813;rs2348321;rs10847321;rs10847323;rs6489163 | NA | 0 |
| 7 | 12:127760001-127800000 | 12:127840001-127880000 | 2.45E-12 | HiC | GSE87112 | IMR90 | intra | rs1810088;rs367839627;rs146043604;rs143439170;rs10773382;rs10847307;rs35154640;rs1552386;rs7314186;rs1979065;rs5801722;rs10744302;12:127775513:A:C;rs10734938;rs35024927;rs10773387;rs978813;rs2348321;rs10847321;rs10847323;rs6489163 | NA | 0 |
| 7 | 12:127760001-127800000 | 12:127880001-127920000 | 2.65E-09 | HiC | GSE87112 | IMR90 | intra | rs1810088;rs367839627;rs146043604;rs143439170;rs10773382;rs10847307;rs35154640;rs1552386;rs7314186;rs1979065;rs5801722;rs10744302;12:127775513:A:C;rs10734938;rs35024927;rs10773387;rs978813;rs2348321;rs10847321;rs10847323;rs6489163 | NA | 0 |
| 7 | 12:127760001-127800000 | 12:128000001-128040000 | 1.23E-11 | HiC | GSE87112 | IMR90 | intra | rs1810088;rs367839627;rs146043604;rs143439170;rs10773382;rs10847307;rs35154640;rs1552386;rs7314186;rs1979065;rs5801722;rs10744302;12:127775513:A:C;rs10734938;rs35024927;rs10773387;rs978813;rs2348321;rs10847321;rs10847323;rs6489163 | NA | 0 |
| 7 | 12:127760001-127800000 | 12:128040001-128080000 | 2.28E-16 | HiC | GSE87112 | IMR90 | intra | rs1810088;rs367839627;rs146043604;rs143439170;rs10773382;rs10847307;rs35154640;rs1552386;rs7314186;rs1979065;rs5801722;rs10744302;12:127775513:A:C;rs10734938;rs35024927;rs10773387;rs978813;rs2348321;rs10847321;rs10847323;rs6489163 | NA | 0 |
| 7 | 12:127760001-127800000 | 12:128120001-128160000 | 2.27E-10 | HiC | GSE87112 | IMR90 | intra | rs1810088;rs367839627;rs146043604;rs143439170;rs10773382;rs10847307;rs35154640;rs1552386;rs7314186;rs1979065;rs5801722;rs10744302;12:127775513:A:C;rs10734938;rs35024927;rs10773387;rs978813;rs2348321;rs10847321;rs10847323;rs6489163 | NA | 0 |
| 7 | 12:127760001-127800000 | 12:128200001-128240000 | 4.06E-17 | HiC | GSE87112 | IMR90 | intra | rs1810088;rs367839627;rs146043604;rs143439170;rs10773382;rs10847307;rs35154640;rs1552386;rs7314186;rs1979065;rs5801722;rs10744302;12:127775513:A:C;rs10734938;rs35024927;rs10773387;rs978813;rs2348321;rs10847321;rs10847323;rs6489163 | NA | 0 |
| 7 | 12:127760001-127800000 | 12:128240001-128280000 | 3.82E-07 | HiC | GSE87112 | IMR90 | intra | rs1810088;rs367839627;rs146043604;rs143439170;rs10773382;rs10847307;rs35154640;rs1552386;rs7314186;rs1979065;rs5801722;rs10744302;12:127775513:A:C;rs10734938;rs35024927;rs10773387;rs978813;rs2348321;rs10847321;rs10847323;rs6489163 | NA | 0 |
| 7 | 12:127760001-127800000 | 12:128360001-128400000 | 7.31E-09 | HiC | GSE87112 | IMR90 | intra | rs1810088;rs367839627;rs146043604;rs143439170;rs10773382;rs10847307;rs35154640;rs1552386;rs7314186;rs1979065;rs5801722;rs10744302;12:127775513:A:C;rs10734938;rs35024927;rs10773387;rs978813;rs2348321;rs10847321;rs10847323;rs6489163 | NA | 0 |
| 7 | 12:127760001-127800000 | 12:128400001-128440000 | 2.23E-17 | HiC | GSE87112 | IMR90 | intra | rs1810088;rs367839627;rs146043604;rs143439170;rs10773382;rs10847307;rs35154640;rs1552386;rs7314186;rs1979065;rs5801722;rs10744302;12:127775513:A:C;rs10734938;rs35024927;rs10773387;rs978813;rs2348321;rs10847321;rs10847323;rs6489163 | NA | 0 |
| 7 | 12:127760001-127800000 | 12:128480001-128520000 | 2.12E-11 | HiC | GSE87112 | IMR90 | intra | rs1810088;rs367839627;rs146043604;rs143439170;rs10773382;rs10847307;rs35154640;rs1552386;rs7314186;rs1979065;rs5801722;rs10744302;12:127775513:A:C;rs10734938;rs35024927;rs10773387;rs978813;rs2348321;rs10847321;rs10847323;rs6489163 | NA | 0 |
| 7 | 12:127760001-127800000 | 12:128520001-128560000 | 8.69E-13 | HiC | GSE87112 | IMR90 | intra | rs1810088;rs367839627;rs146043604;rs143439170;rs10773382;rs10847307;rs35154640;rs1552386;rs7314186;rs1979065;rs5801722;rs10744302;12:127775513:A:C;rs10734938;rs35024927;rs10773387;rs978813;rs2348321;rs10847321;rs10847323;rs6489163 | NA | 0 |
| 7 | 12:127760001-127800000 | 12:128680001-128720000 | 6.80E-11 | HiC | GSE87112 | IMR90 | intra | rs1810088;rs367839627;rs146043604;rs143439170;rs10773382;rs10847307;rs35154640;rs1552386;rs7314186;rs1979065;rs5801722;rs10744302;12:127775513:A:C;rs10734938;rs35024927;rs10773387;rs978813;rs2348321;rs10847321;rs10847323;rs6489163 | NA | 0 |
| 7 | 12:127760001-127800000 | 12:128720001-128760000 | 7.28E-10 | HiC | GSE87112 | IMR90 | intra | rs1810088;rs367839627;rs146043604;rs143439170;rs10773382;rs10847307;rs35154640;rs1552386;rs7314186;rs1979065;rs5801722;rs10744302;12:127775513:A:C;rs10734938;rs35024927;rs10773387;rs978813;rs2348321;rs10847321;rs10847323;rs6489163 | ENSG00000181234 | 0 |
| 7 | 12:127760001-127800000 | 12:128840001-128880000 | 3.82E-09 | HiC | GSE87112 | IMR90 | intra | rs1810088;rs367839627;rs146043604;rs143439170;rs10773382;rs10847307;rs35154640;rs1552386;rs7314186;rs1979065;rs5801722;rs10744302;12:127775513:A:C;rs10734938;rs35024927;rs10773387;rs978813;rs2348321;rs10847321;rs10847323;rs6489163 | NA | 0 |
| 7 | 12:127760001-127800000 | 12:129600001-129640000 | 1.03E-08 | HiC | GSE87112 | IMR90 | intra | rs1810088;rs367839627;rs146043604;rs143439170;rs10773382;rs10847307;rs35154640;rs1552386;rs7314186;rs1979065;rs5801722;rs10744302;12:127775513:A:C;rs10734938;rs35024927;rs10773387;rs978813;rs2348321;rs10847321;rs10847323;rs6489163 | NA | 0 |
| 7 | 12:127760001-127800000 | 12:129640001-129680000 | 8.89E-17 | HiC | GSE87112 | IMR90 | intra | rs1810088;rs367839627;rs146043604;rs143439170;rs10773382;rs10847307;rs35154640;rs1552386;rs7314186;rs1979065;rs5801722;rs10744302;12:127775513:A:C;rs10734938;rs35024927;rs10773387;rs978813;rs2348321;rs10847321;rs10847323;rs6489163 | NA | 0 |
| 7 | 12:127760001-127800000 | 12:129760001-129800000 | 6.69E-09 | HiC | GSE87112 | IMR90 | intra | rs1810088;rs367839627;rs146043604;rs143439170;rs10773382;rs10847307;rs35154640;rs1552386;rs7314186;rs1979065;rs5801722;rs10744302;12:127775513:A:C;rs10734938;rs35024927;rs10773387;rs978813;rs2348321;rs10847321;rs10847323;rs6489163 | NA | 0 |
| 7 | 12:127760001-127800000 | 12:129800001-129840000 | 4.63E-09 | HiC | GSE87112 | IMR90 | intra | rs1810088;rs367839627;rs146043604;rs143439170;rs10773382;rs10847307;rs35154640;rs1552386;rs7314186;rs1979065;rs5801722;rs10744302;12:127775513:A:C;rs10734938;rs35024927;rs10773387;rs978813;rs2348321;rs10847321;rs10847323;rs6489163 | NA | 0 |
| 7 | 12:127760001-127800000 | 12:129880001-129920000 | 9.77E-07 | HiC | GSE87112 | IMR90 | intra | rs1810088;rs367839627;rs146043604;rs143439170;rs10773382;rs10847307;rs35154640;rs1552386;rs7314186;rs1979065;rs5801722;rs10744302;12:127775513:A:C;rs10734938;rs35024927;rs10773387;rs978813;rs2348321;rs10847321;rs10847323;rs6489163 | NA | 0 |
| 7 | 12:127760001-127800000 | 12:129960001-130000000 | 6.19E-35 | HiC | GSE87112 | IMR90 | intra | rs1810088;rs367839627;rs146043604;rs143439170;rs10773382;rs10847307;rs35154640;rs1552386;rs7314186;rs1979065;rs5801722;rs10744302;12:127775513:A:C;rs10734938;rs35024927;rs10773387;rs978813;rs2348321;rs10847321;rs10847323;rs6489163 | NA | 0 |
| 7 | 12:127760001-127800000 | 12:130000001-130040000 | 2.53E-18 | HiC | GSE87112 | IMR90 | intra | rs1810088;rs367839627;rs146043604;rs143439170;rs10773382;rs10847307;rs35154640;rs1552386;rs7314186;rs1979065;rs5801722;rs10744302;12:127775513:A:C;rs10734938;rs35024927;rs10773387;rs978813;rs2348321;rs10847321;rs10847323;rs6489163 | NA | 0 |
| 7 | 12:127760001-127800000 | 12:130040001-130080000 | 9.64E-08 | HiC | GSE87112 | IMR90 | intra | rs1810088;rs367839627;rs146043604;rs143439170;rs10773382;rs10847307;rs35154640;rs1552386;rs7314186;rs1979065;rs5801722;rs10744302;12:127775513:A:C;rs10734938;rs35024927;rs10773387;rs978813;rs2348321;rs10847321;rs10847323;rs6489163 | NA | 0 |
| 7 | 12:127760001-127800000 | 12:130160001-130200000 | 6.83E-10 | HiC | GSE87112 | IMR90 | intra | rs1810088;rs367839627;rs146043604;rs143439170;rs10773382;rs10847307;rs35154640;rs1552386;rs7314186;rs1979065;rs5801722;rs10744302;12:127775513:A:C;rs10734938;rs35024927;rs10773387;rs978813;rs2348321;rs10847321;rs10847323;rs6489163 | NA | 0 |
| 7 | 12:127760001-127800000 | 12:130200001-130240000 | 9.77E-07 | HiC | GSE87112 | IMR90 | intra | rs1810088;rs367839627;rs146043604;rs143439170;rs10773382;rs10847307;rs35154640;rs1552386;rs7314186;rs1979065;rs5801722;rs10744302;12:127775513:A:C;rs10734938;rs35024927;rs10773387;rs978813;rs2348321;rs10847321;rs10847323;rs6489163 | NA | 0 |
| 7 | 12:127760001-127800000 | 12:130240001-130280000 | 7.94E-13 | HiC | GSE87112 | IMR90 | intra | rs1810088;rs367839627;rs146043604;rs143439170;rs10773382;rs10847307;rs35154640;rs1552386;rs7314186;rs1979065;rs5801722;rs10744302;12:127775513:A:C;rs10734938;rs35024927;rs10773387;rs978813;rs2348321;rs10847321;rs10847323;rs6489163 | NA | 0 |
| 7 | 12:127760001-127800000 | 12:130360001-130400000 | 4.90E-11 | HiC | GSE87112 | IMR90 | intra | rs1810088;rs367839627;rs146043604;rs143439170;rs10773382;rs10847307;rs35154640;rs1552386;rs7314186;rs1979065;rs5801722;rs10744302;12:127775513:A:C;rs10734938;rs35024927;rs10773387;rs978813;rs2348321;rs10847321;rs10847323;rs6489163 | ENSG00000151952 | 0 |
| 7 | 12:127760001-127800000 | 12:130400001-130440000 | 2.32E-13 | HiC | GSE87112 | IMR90 | intra | rs1810088;rs367839627;rs146043604;rs143439170;rs10773382;rs10847307;rs35154640;rs1552386;rs7314186;rs1979065;rs5801722;rs10744302;12:127775513:A:C;rs10734938;rs35024927;rs10773387;rs978813;rs2348321;rs10847321;rs10847323;rs6489163 | NA | 0 |
| 7 | 12:127760001-127800000 | 12:130440001-130480000 | 4.72E-13 | HiC | GSE87112 | IMR90 | intra | rs1810088;rs367839627;rs146043604;rs143439170;rs10773382;rs10847307;rs35154640;rs1552386;rs7314186;rs1979065;rs5801722;rs10744302;12:127775513:A:C;rs10734938;rs35024927;rs10773387;rs978813;rs2348321;rs10847321;rs10847323;rs6489163 | NA | 0 |
| 7 | 12:127760001-127800000 | 12:130600001-130640000 | 1.99E-15 | HiC | GSE87112 | IMR90 | intra | rs1810088;rs367839627;rs146043604;rs143439170;rs10773382;rs10847307;rs35154640;rs1552386;rs7314186;rs1979065;rs5801722;rs10744302;12:127775513:A:C;rs10734938;rs35024927;rs10773387;rs978813;rs2348321;rs10847321;rs10847323;rs6489163 | NA | 0 |
| 7 | 12:127760001-127800000 | 12:130680001-130720000 | 1.33E-08 | HiC | GSE87112 | IMR90 | intra | rs1810088;rs367839627;rs146043604;rs143439170;rs10773382;rs10847307;rs35154640;rs1552386;rs7314186;rs1979065;rs5801722;rs10744302;12:127775513:A:C;rs10734938;rs35024927;rs10773387;rs978813;rs2348321;rs10847321;rs10847323;rs6489163 | NA | 0 |
| 1 | 3:37560001-37600000 | 3:37480001-37520000 | 6.21E-07 | HiC | GSE87112 | IMR90 | intra | rs74985154;rs3733138;rs3733140;rs75619156;rs17229924;rs2162356;rs74786716;rs77820597;rs112585731;rs915631;rs79149273;rs17230261;rs199607377;rs2162355;rs76952775;rs75469174;rs78210776;rs77162103;rs75217006;rs78292414 | ENSG00000144668 | 1 |
| 2 | 3:96560001-96600000 | 3:93800001-93840000 | 9.01E-09 | HiC | GSE87112 | IMR90 | intra | rs16836353;rs62262935;rs62262936;rs62262937;rs62262938;rs62262940;rs62262941;rs62262942;rs62262944;rs62262946;rs62262947;rs62262948;rs62262949;rs62262950;rs62262951 | NA | 0 |
| 2 | 3:96600001-96640000 | 3:93840001-93880000 | 2.34E-09 | HiC | GSE87112 | IMR90 | intra | rs62262952;rs62262953;rs116253310;rs76857958;rs111706803;rs62262955;rs62262957;rs62262958;rs62263676;rs187619159;rs62263677;rs62263678;rs574559070;rs534937763;rs62263680;rs62263682;rs62263684;rs16836982;rs16836986;rs112527011;rs62263685;rs62263686 | NA | 0 |
| 2 | 3:96520001-96560000 | 3:93880001-93920000 | 8.05E-16 | HiC | GSE87112 | IMR90 | intra | rs62262901;rs62262902;rs62262903;rs62262930;rs151165295;rs62262931;rs62262933;rs62262934;rs74641574;rs79645223;rs2318156;rs2318155;rs2318154 | NA | 0 |
| 2 | 3:96600001-96640000 | 3:93880001-93920000 | 3.02E-18 | HiC | GSE87112 | IMR90 | intra | rs62262952;rs62262953;rs116253310;rs76857958;rs111706803;rs62262955;rs62262957;rs62262958;rs62263676;rs187619159;rs62263677;rs62263678;rs574559070;rs534937763;rs62263680;rs62263682;rs62263684;rs16836982;rs16836986;rs112527011;rs62263685;rs62263686 | NA | 0 |
| 2 | 3:96680001-96720000 | 3:93880001-93920000 | 1.38E-19 | HiC | GSE87112 | IMR90 | intra | rs62263705;rs62263706;rs6762348;rs62263709;rs137983052;rs62263734;rs2856474;rs2612274;rs553308194 | NA | 0 |
| 2 | 3:96520001-96560000 | 3:93920001-93960000 | 9.25E-10 | HiC | GSE87112 | IMR90 | intra | rs62262901;rs62262902;rs62262903;rs62262930;rs151165295;rs62262931;rs62262933;rs62262934;rs74641574;rs79645223;rs2318156;rs2318155;rs2318154 | NA | 0 |
| 2 | 3:96560001-96600000 | 3:93920001-93960000 | 8.26E-08 | HiC | GSE87112 | IMR90 | intra | rs16836353;rs62262935;rs62262936;rs62262937;rs62262938;rs62262940;rs62262941;rs62262942;rs62262944;rs62262946;rs62262947;rs62262948;rs62262949;rs62262950;rs62262951 | NA | 0 |
| 2 | 3:96680001-96720000 | 3:93920001-93960000 | 3.34E-08 | HiC | GSE87112 | IMR90 | intra | rs62263705;rs62263706;rs6762348;rs62263709;rs137983052;rs62263734;rs2856474;rs2612274;rs553308194 | NA | 0 |
| 2 | 3:96520001-96560000 | 3:93960001-94000000 | 5.70E-27 | HiC | GSE87112 | IMR90 | intra | rs62262901;rs62262902;rs62262903;rs62262930;rs151165295;rs62262931;rs62262933;rs62262934;rs74641574;rs79645223;rs2318156;rs2318155;rs2318154 | NA | 0 |
| 2 | 3:96560001-96600000 | 3:93960001-94000000 | 6.17E-11 | HiC | GSE87112 | IMR90 | intra | rs16836353;rs62262935;rs62262936;rs62262937;rs62262938;rs62262940;rs62262941;rs62262942;rs62262944;rs62262946;rs62262947;rs62262948;rs62262949;rs62262950;rs62262951 | NA | 0 |
| 2 | 3:96600001-96640000 | 3:93960001-94000000 | 1.69E-12 | HiC | GSE87112 | IMR90 | intra | rs62262952;rs62262953;rs116253310;rs76857958;rs111706803;rs62262955;rs62262957;rs62262958;rs62263676;rs187619159;rs62263677;rs62263678;rs574559070;rs534937763;rs62263680;rs62263682;rs62263684;rs16836982;rs16836986;rs112527011;rs62263685;rs62263686 | NA | 0 |
| 2 | 3:96680001-96720000 | 3:93960001-94000000 | 1.16E-12 | HiC | GSE87112 | IMR90 | intra | rs62263705;rs62263706;rs6762348;rs62263709;rs137983052;rs62263734;rs2856474;rs2612274;rs553308194 | NA | 0 |
| 2 | 3:96520001-96560000 | 3:94000001-94040000 | 2.74E-32 | HiC | GSE87112 | IMR90 | intra | rs62262901;rs62262902;rs62262903;rs62262930;rs151165295;rs62262931;rs62262933;rs62262934;rs74641574;rs79645223;rs2318156;rs2318155;rs2318154 | NA | 0 |
| 2 | 3:96560001-96600000 | 3:94000001-94040000 | 8.78E-28 | HiC | GSE87112 | IMR90 | intra | rs16836353;rs62262935;rs62262936;rs62262937;rs62262938;rs62262940;rs62262941;rs62262942;rs62262944;rs62262946;rs62262947;rs62262948;rs62262949;rs62262950;rs62262951 | NA | 0 |
| 2 | 3:96600001-96640000 | 3:94000001-94040000 | 7.71E-38 | HiC | GSE87112 | IMR90 | intra | rs62262952;rs62262953;rs116253310;rs76857958;rs111706803;rs62262955;rs62262957;rs62262958;rs62263676;rs187619159;rs62263677;rs62263678;rs574559070;rs534937763;rs62263680;rs62263682;rs62263684;rs16836982;rs16836986;rs112527011;rs62263685;rs62263686 | NA | 0 |
| 2 | 3:96640001-96680000 | 3:94000001-94040000 | 8.26E-08 | HiC | GSE87112 | IMR90 | intra | rs62263687;rs62263693;rs2318066;rs62263694;rs74563548;rs62263696;rs185127539;rs145486125;rs62263697;rs148690862;rs62263699;rs149222683;rs111574505;rs62263701;rs62263702;rs62263703;rs75261331;rs62263704;rs189373463 | NA | 0 |
| 2 | 3:96680001-96720000 | 3:94000001-94040000 | 1.81E-38 | HiC | GSE87112 | IMR90 | intra | rs62263705;rs62263706;rs6762348;rs62263709;rs137983052;rs62263734;rs2856474;rs2612274;rs553308194 | NA | 0 |
| 2 | 3:96720001-96760000 | 3:94000001-94040000 | 8.72E-17 | HiC | GSE87112 | IMR90 | intra | rs2027816;rs2856463;rs2019818;rs115680726;rs112449918;rs565327317;rs2213251;rs2213252;rs2856466 | NA | 0 |
| 2 | 3:96520001-96560000 | 3:94040001-94080000 | 1.43E-18 | HiC | GSE87112 | IMR90 | intra | rs62262901;rs62262902;rs62262903;rs62262930;rs151165295;rs62262931;rs62262933;rs62262934;rs74641574;rs79645223;rs2318156;rs2318155;rs2318154 | NA | 0 |
| 2 | 3:96600001-96640000 | 3:94040001-94080000 | 1.76E-12 | HiC | GSE87112 | IMR90 | intra | rs62262952;rs62262953;rs116253310;rs76857958;rs111706803;rs62262955;rs62262957;rs62262958;rs62263676;rs187619159;rs62263677;rs62263678;rs574559070;rs534937763;rs62263680;rs62263682;rs62263684;rs16836982;rs16836986;rs112527011;rs62263685;rs62263686 | NA | 0 |
| 2 | 3:96680001-96720000 | 3:94040001-94080000 | 4.72E-10 | HiC | GSE87112 | IMR90 | intra | rs62263705;rs62263706;rs6762348;rs62263709;rs137983052;rs62263734;rs2856474;rs2612274;rs553308194 | NA | 0 |
| 2 | 3:96520001-96560000 | 3:94120001-94160000 | 1.51E-11 | HiC | GSE87112 | IMR90 | intra | rs62262901;rs62262902;rs62262903;rs62262930;rs151165295;rs62262931;rs62262933;rs62262934;rs74641574;rs79645223;rs2318156;rs2318155;rs2318154 | NA | 0 |
| 2 | 3:96560001-96600000 | 3:94120001-94160000 | 6.82E-09 | HiC | GSE87112 | IMR90 | intra | rs16836353;rs62262935;rs62262936;rs62262937;rs62262938;rs62262940;rs62262941;rs62262942;rs62262944;rs62262946;rs62262947;rs62262948;rs62262949;rs62262950;rs62262951 | NA | 0 |
| 2 | 3:96680001-96720000 | 3:94120001-94160000 | 7.30E-12 | HiC | GSE87112 | IMR90 | intra | rs62263705;rs62263706;rs6762348;rs62263709;rs137983052;rs62263734;rs2856474;rs2612274;rs553308194 | NA | 0 |
| 2 | 3:96600001-96640000 | 3:94200001-94240000 | 2.24E-13 | HiC | GSE87112 | IMR90 | intra | rs62262952;rs62262953;rs116253310;rs76857958;rs111706803;rs62262955;rs62262957;rs62262958;rs62263676;rs187619159;rs62263677;rs62263678;rs574559070;rs534937763;rs62263680;rs62263682;rs62263684;rs16836982;rs16836986;rs112527011;rs62263685;rs62263686 | NA | 0 |
| 2 | 3:96680001-96720000 | 3:94200001-94240000 | 1.57E-08 | HiC | GSE87112 | IMR90 | intra | rs62263705;rs62263706;rs6762348;rs62263709;rs137983052;rs62263734;rs2856474;rs2612274;rs553308194 | NA | 0 |
| 2 | 3:96520001-96560000 | 3:94240001-94280000 | 8.04E-13 | HiC | GSE87112 | IMR90 | intra | rs62262901;rs62262902;rs62262903;rs62262930;rs151165295;rs62262931;rs62262933;rs62262934;rs74641574;rs79645223;rs2318156;rs2318155;rs2318154 | NA | 0 |
| 2 | 3:96560001-96600000 | 3:94240001-94280000 | 5.68E-09 | HiC | GSE87112 | IMR90 | intra | rs16836353;rs62262935;rs62262936;rs62262937;rs62262938;rs62262940;rs62262941;rs62262942;rs62262944;rs62262946;rs62262947;rs62262948;rs62262949;rs62262950;rs62262951 | NA | 0 |
| 2 | 3:96680001-96720000 | 3:94240001-94280000 | 2.34E-08 | HiC | GSE87112 | IMR90 | intra | rs62263705;rs62263706;rs6762348;rs62263709;rs137983052;rs62263734;rs2856474;rs2612274;rs553308194 | NA | 0 |
| 2 | 3:96520001-96560000 | 3:94320001-94360000 | 3.36E-10 | HiC | GSE87112 | IMR90 | intra | rs62262901;rs62262902;rs62262903;rs62262930;rs151165295;rs62262931;rs62262933;rs62262934;rs74641574;rs79645223;rs2318156;rs2318155;rs2318154 | NA | 0 |
| 2 | 3:96560001-96600000 | 3:94320001-94360000 | 1.78E-11 | HiC | GSE87112 | IMR90 | intra | rs16836353;rs62262935;rs62262936;rs62262937;rs62262938;rs62262940;rs62262941;rs62262942;rs62262944;rs62262946;rs62262947;rs62262948;rs62262949;rs62262950;rs62262951 | NA | 0 |
| 2 | 3:96600001-96640000 | 3:94320001-94360000 | 6.42E-10 | HiC | GSE87112 | IMR90 | intra | rs62262952;rs62262953;rs116253310;rs76857958;rs111706803;rs62262955;rs62262957;rs62262958;rs62263676;rs187619159;rs62263677;rs62263678;rs574559070;rs534937763;rs62263680;rs62263682;rs62263684;rs16836982;rs16836986;rs112527011;rs62263685;rs62263686 | NA | 0 |
| 2 | 3:96680001-96720000 | 3:94320001-94360000 | 4.66E-08 | HiC | GSE87112 | IMR90 | intra | rs62263705;rs62263706;rs6762348;rs62263709;rs137983052;rs62263734;rs2856474;rs2612274;rs553308194 | NA | 0 |
| 2 | 3:96680001-96720000 | 3:94360001-94400000 | 5.68E-09 | HiC | GSE87112 | IMR90 | intra | rs62263705;rs62263706;rs6762348;rs62263709;rs137983052;rs62263734;rs2856474;rs2612274;rs553308194 | NA | 0 |
| 2 | 3:96600001-96640000 | 3:94400001-94440000 | 4.45E-08 | HiC | GSE87112 | IMR90 | intra | rs62262952;rs62262953;rs116253310;rs76857958;rs111706803;rs62262955;rs62262957;rs62262958;rs62263676;rs187619159;rs62263677;rs62263678;rs574559070;rs534937763;rs62263680;rs62263682;rs62263684;rs16836982;rs16836986;rs112527011;rs62263685;rs62263686 | NA | 0 |
| 2 | 3:96680001-96720000 | 3:94400001-94440000 | 7.76E-09 | HiC | GSE87112 | IMR90 | intra | rs62263705;rs62263706;rs6762348;rs62263709;rs137983052;rs62263734;rs2856474;rs2612274;rs553308194 | NA | 0 |
| 2 | 3:96680001-96720000 | 3:94480001-94520000 | 4.08E-09 | HiC | GSE87112 | IMR90 | intra | rs62263705;rs62263706;rs6762348;rs62263709;rs137983052;rs62263734;rs2856474;rs2612274;rs553308194 | NA | 0 |
| 2 | 3:96520001-96560000 | 3:94520001-94560000 | 1.70E-08 | HiC | GSE87112 | IMR90 | intra | rs62262901;rs62262902;rs62262903;rs62262930;rs151165295;rs62262931;rs62262933;rs62262934;rs74641574;rs79645223;rs2318156;rs2318155;rs2318154 | NA | 0 |
| 2 | 3:96560001-96600000 | 3:94520001-94560000 | 1.51E-15 | HiC | GSE87112 | IMR90 | intra | rs16836353;rs62262935;rs62262936;rs62262937;rs62262938;rs62262940;rs62262941;rs62262942;rs62262944;rs62262946;rs62262947;rs62262948;rs62262949;rs62262950;rs62262951 | NA | 0 |
| 2 | 3:96600001-96640000 | 3:94520001-94560000 | 2.38E-10 | HiC | GSE87112 | IMR90 | intra | rs62262952;rs62262953;rs116253310;rs76857958;rs111706803;rs62262955;rs62262957;rs62262958;rs62263676;rs187619159;rs62263677;rs62263678;rs574559070;rs534937763;rs62263680;rs62263682;rs62263684;rs16836982;rs16836986;rs112527011;rs62263685;rs62263686 | NA | 0 |
| 2 | 3:96520001-96560000 | 3:94560001-94600000 | 7.22E-08 | HiC | GSE87112 | IMR90 | intra | rs62262901;rs62262902;rs62262903;rs62262930;rs151165295;rs62262931;rs62262933;rs62262934;rs74641574;rs79645223;rs2318156;rs2318155;rs2318154 | NA | 0 |
| 2 | 3:96600001-96640000 | 3:94560001-94600000 | 4.04E-08 | HiC | GSE87112 | IMR90 | intra | rs62262952;rs62262953;rs116253310;rs76857958;rs111706803;rs62262955;rs62262957;rs62262958;rs62263676;rs187619159;rs62263677;rs62263678;rs574559070;rs534937763;rs62263680;rs62263682;rs62263684;rs16836982;rs16836986;rs112527011;rs62263685;rs62263686 | NA | 0 |
| 2 | 3:96680001-96720000 | 3:94560001-94600000 | 2.34E-08 | HiC | GSE87112 | IMR90 | intra | rs62263705;rs62263706;rs6762348;rs62263709;rs137983052;rs62263734;rs2856474;rs2612274;rs553308194 | NA | 0 |
| 2 | 3:96520001-96560000 | 3:94600001-94640000 | 7.79E-11 | HiC | GSE87112 | IMR90 | intra | rs62262901;rs62262902;rs62262903;rs62262930;rs151165295;rs62262931;rs62262933;rs62262934;rs74641574;rs79645223;rs2318156;rs2318155;rs2318154 | NA | 0 |
| 2 | 3:96680001-96720000 | 3:94600001-94640000 | 2.38E-10 | HiC | GSE87112 | IMR90 | intra | rs62263705;rs62263706;rs6762348;rs62263709;rs137983052;rs62263734;rs2856474;rs2612274;rs553308194 | NA | 0 |
| 2 | 3:96520001-96560000 | 3:94640001-94680000 | 6.47E-13 | HiC | GSE87112 | IMR90 | intra | rs62262901;rs62262902;rs62262903;rs62262930;rs151165295;rs62262931;rs62262933;rs62262934;rs74641574;rs79645223;rs2318156;rs2318155;rs2318154 | NA | 0 |
| 2 | 3:96560001-96600000 | 3:94640001-94680000 | 2.16E-11 | HiC | GSE87112 | IMR90 | intra | rs16836353;rs62262935;rs62262936;rs62262937;rs62262938;rs62262940;rs62262941;rs62262942;rs62262944;rs62262946;rs62262947;rs62262948;rs62262949;rs62262950;rs62262951 | NA | 0 |
| 2 | 3:96680001-96720000 | 3:94640001-94680000 | 1.46E-24 | HiC | GSE87112 | IMR90 | intra | rs62263705;rs62263706;rs6762348;rs62263709;rs137983052;rs62263734;rs2856474;rs2612274;rs553308194 | NA | 0 |
| 2 | 3:96520001-96560000 | 3:94680001-94720000 | 2.81E-13 | HiC | GSE87112 | IMR90 | intra | rs62262901;rs62262902;rs62262903;rs62262930;rs151165295;rs62262931;rs62262933;rs62262934;rs74641574;rs79645223;rs2318156;rs2318155;rs2318154 | NA | 0 |
| 2 | 3:96560001-96600000 | 3:94680001-94720000 | 4.08E-10 | HiC | GSE87112 | IMR90 | intra | rs16836353;rs62262935;rs62262936;rs62262937;rs62262938;rs62262940;rs62262941;rs62262942;rs62262944;rs62262946;rs62262947;rs62262948;rs62262949;rs62262950;rs62262951 | NA | 0 |
| 2 | 3:96600001-96640000 | 3:94680001-94720000 | 2.95E-07 | HiC | GSE87112 | IMR90 | intra | rs62262952;rs62262953;rs116253310;rs76857958;rs111706803;rs62262955;rs62262957;rs62262958;rs62263676;rs187619159;rs62263677;rs62263678;rs574559070;rs534937763;rs62263680;rs62263682;rs62263684;rs16836982;rs16836986;rs112527011;rs62263685;rs62263686 | NA | 0 |
| 2 | 3:96680001-96720000 | 3:94680001-94720000 | 5.37E-08 | HiC | GSE87112 | IMR90 | intra | rs62263705;rs62263706;rs6762348;rs62263709;rs137983052;rs62263734;rs2856474;rs2612274;rs553308194 | NA | 0 |
| 2 | 3:96520001-96560000 | 3:94800001-94840000 | 2.57E-10 | HiC | GSE87112 | IMR90 | intra | rs62262901;rs62262902;rs62262903;rs62262930;rs151165295;rs62262931;rs62262933;rs62262934;rs74641574;rs79645223;rs2318156;rs2318155;rs2318154 | NA | 0 |
| 2 | 3:96600001-96640000 | 3:94800001-94840000 | 3.27E-09 | HiC | GSE87112 | IMR90 | intra | rs62262952;rs62262953;rs116253310;rs76857958;rs111706803;rs62262955;rs62262957;rs62262958;rs62263676;rs187619159;rs62263677;rs62263678;rs574559070;rs534937763;rs62263680;rs62263682;rs62263684;rs16836982;rs16836986;rs112527011;rs62263685;rs62263686 | NA | 0 |
| 2 | 3:96520001-96560000 | 3:94840001-94880000 | 3.45E-20 | HiC | GSE87112 | IMR90 | intra | rs62262901;rs62262902;rs62262903;rs62262930;rs151165295;rs62262931;rs62262933;rs62262934;rs74641574;rs79645223;rs2318156;rs2318155;rs2318154 | NA | 0 |
| 2 | 3:96680001-96720000 | 3:94840001-94880000 | 1.48E-11 | HiC | GSE87112 | IMR90 | intra | rs62263705;rs62263706;rs6762348;rs62263709;rs137983052;rs62263734;rs2856474;rs2612274;rs553308194 | NA | 0 |
| 2 | 3:96520001-96560000 | 3:94920001-94960000 | 4.75E-08 | HiC | GSE87112 | IMR90 | intra | rs62262901;rs62262902;rs62262903;rs62262930;rs151165295;rs62262931;rs62262933;rs62262934;rs74641574;rs79645223;rs2318156;rs2318155;rs2318154 | NA | 0 |
| 2 | 3:96600001-96640000 | 3:94920001-94960000 | 4.64E-10 | HiC | GSE87112 | IMR90 | intra | rs62262952;rs62262953;rs116253310;rs76857958;rs111706803;rs62262955;rs62262957;rs62262958;rs62263676;rs187619159;rs62263677;rs62263678;rs574559070;rs534937763;rs62263680;rs62263682;rs62263684;rs16836982;rs16836986;rs112527011;rs62263685;rs62263686 | NA | 0 |
| 2 | 3:96680001-96720000 | 3:94920001-94960000 | 3.31E-12 | HiC | GSE87112 | IMR90 | intra | rs62263705;rs62263706;rs6762348;rs62263709;rs137983052;rs62263734;rs2856474;rs2612274;rs553308194 | NA | 0 |
| 2 | 3:96680001-96720000 | 3:94960001-95000000 | 6.57E-07 | HiC | GSE87112 | IMR90 | intra | rs62263705;rs62263706;rs6762348;rs62263709;rs137983052;rs62263734;rs2856474;rs2612274;rs553308194 | NA | 0 |
| 2 | 3:96600001-96640000 | 3:95000001-95040000 | 1.61E-08 | HiC | GSE87112 | IMR90 | intra | rs62262952;rs62262953;rs116253310;rs76857958;rs111706803;rs62262955;rs62262957;rs62262958;rs62263676;rs187619159;rs62263677;rs62263678;rs574559070;rs534937763;rs62263680;rs62263682;rs62263684;rs16836982;rs16836986;rs112527011;rs62263685;rs62263686 | NA | 0 |
| 2 | 3:96680001-96720000 | 3:95000001-95040000 | 3.73E-07 | HiC | GSE87112 | IMR90 | intra | rs62263705;rs62263706;rs6762348;rs62263709;rs137983052;rs62263734;rs2856474;rs2612274;rs553308194 | NA | 0 |
| 2 | 3:96520001-96560000 | 3:95040001-95080000 | 1.23E-13 | HiC | GSE87112 | IMR90 | intra | rs62262901;rs62262902;rs62262903;rs62262930;rs151165295;rs62262931;rs62262933;rs62262934;rs74641574;rs79645223;rs2318156;rs2318155;rs2318154 | NA | 0 |
| 2 | 3:96600001-96640000 | 3:95040001-95080000 | 1.05E-08 | HiC | GSE87112 | IMR90 | intra | rs62262952;rs62262953;rs116253310;rs76857958;rs111706803;rs62262955;rs62262957;rs62262958;rs62263676;rs187619159;rs62263677;rs62263678;rs574559070;rs534937763;rs62263680;rs62263682;rs62263684;rs16836982;rs16836986;rs112527011;rs62263685;rs62263686 | NA | 0 |
| 2 | 3:96520001-96560000 | 3:95120001-95160000 | 9.72E-14 | HiC | GSE87112 | IMR90 | intra | rs62262901;rs62262902;rs62262903;rs62262930;rs151165295;rs62262931;rs62262933;rs62262934;rs74641574;rs79645223;rs2318156;rs2318155;rs2318154 | NA | 0 |
| 2 | 3:96680001-96720000 | 3:95120001-95160000 | 5.93E-14 | HiC | GSE87112 | IMR90 | intra | rs62263705;rs62263706;rs6762348;rs62263709;rs137983052;rs62263734;rs2856474;rs2612274;rs553308194 | NA | 0 |
| 2 | 3:96520001-96560000 | 3:95200001-95240000 | 1.07E-09 | HiC | GSE87112 | IMR90 | intra | rs62262901;rs62262902;rs62262903;rs62262930;rs151165295;rs62262931;rs62262933;rs62262934;rs74641574;rs79645223;rs2318156;rs2318155;rs2318154 | NA | 0 |
| 2 | 3:96680001-96720000 | 3:95200001-95240000 | 3.39E-14 | HiC | GSE87112 | IMR90 | intra | rs62263705;rs62263706;rs6762348;rs62263709;rs137983052;rs62263734;rs2856474;rs2612274;rs553308194 | NA | 0 |
| 2 | 3:96680001-96720000 | 3:95240001-95280000 | 9.73E-08 | HiC | GSE87112 | IMR90 | intra | rs62263705;rs62263706;rs6762348;rs62263709;rs137983052;rs62263734;rs2856474;rs2612274;rs553308194 | NA | 0 |
| 2 | 3:96680001-96720000 | 3:95280001-95320000 | 5.69E-07 | HiC | GSE87112 | IMR90 | intra | rs62263705;rs62263706;rs6762348;rs62263709;rs137983052;rs62263734;rs2856474;rs2612274;rs553308194 | NA | 0 |
| 2 | 3:96520001-96560000 | 3:95400001-95440000 | 1.00E-09 | HiC | GSE87112 | IMR90 | intra | rs62262901;rs62262902;rs62262903;rs62262930;rs151165295;rs62262931;rs62262933;rs62262934;rs74641574;rs79645223;rs2318156;rs2318155;rs2318154 | NA | 0 |
| 4 | 5:108560001-108600000 | 5:107760001-107800000 | 9.10E-09 | HiC | GSE87112 | IMR90 | intra | rs35812497;rs75803484;rs75073283;rs76430100;rs75782477;rs77992445;rs61701247;rs17161655;rs115979287;rs17161659;rs151072743;rs17161665;rs6863688;5:108591279:C:T;5:108591279:A:T;rs1363213;rs1862201;rs1862202;rs1862203;rs5870359 | NA | 0 |
| 4 | 5:108600001-108640000 | 5:107760001-107800000 | 5.65E-07 | HiC | GSE87112 | IMR90 | intra | rs11744353;rs79776200;rs76069623;rs76043709;rs78729571;rs36123121;rs11748450;rs80234158;rs78146542;rs185072862;rs137867205;rs189006725;rs4388251;rs4438924;rs4541698;rs4388252;rs4392675;rs200252534;rs145520315;rs147699420;rs77803982;rs79469094;rs75077625;rs74379173;rs78664536;rs549536839;rs570631944;rs78784808;rs78340649;rs201569786;rs75649455;rs78814720;rs77561644;rs76565008;rs74436342;rs1592807;rs1592810;rs1592811;rs7700458;rs6594365;rs6863893;rs140163909;rs146837743;rs7705919;rs201046346;rs202152005;rs199869120;rs111726541;rs373673987;rs113026830;rs148211606;rs201619508;rs201169921;rs146644914;rs116649742;rs79893293;rs57629433;rs60335509;rs58453996;rs59466242;rs60878839;rs11743452;rs1833567;rs1833568;rs1833569;rs7721272;rs7721427;rs7721730;rs7721870;rs7722100;rs7704359;rs1895200;rs1895201;rs77974856;rs60905342;rs11746207;rs11739672;rs11749718;rs78518991;rs76243602;rs76383235;rs11741980;rs35243960;rs116462975;rs139801813;rs113103584;rs74727230;rs75919325;rs79624714;rs2080858;rs138092354;rs148285468;rs114455395;rs74850689;rs10463599 | NA | 0 |
| 4 | 5:108560001-108600000 | 5:107800001-107840000 | 2.04E-11 | HiC | GSE87112 | IMR90 | intra | rs35812497;rs75803484;rs75073283;rs76430100;rs75782477;rs77992445;rs61701247;rs17161655;rs115979287;rs17161659;rs151072743;rs17161665;rs6863688;5:108591279:C:T;5:108591279:A:T;rs1363213;rs1862201;rs1862202;rs1862203;rs5870359 | NA | 0 |
| 4 | 5:108600001-108640000 | 5:107800001-107840000 | 4.47E-11 | HiC | GSE87112 | IMR90 | intra | rs11744353;rs79776200;rs76069623;rs76043709;rs78729571;rs36123121;rs11748450;rs80234158;rs78146542;rs185072862;rs137867205;rs189006725;rs4388251;rs4438924;rs4541698;rs4388252;rs4392675;rs200252534;rs145520315;rs147699420;rs77803982;rs79469094;rs75077625;rs74379173;rs78664536;rs549536839;rs570631944;rs78784808;rs78340649;rs201569786;rs75649455;rs78814720;rs77561644;rs76565008;rs74436342;rs1592807;rs1592810;rs1592811;rs7700458;rs6594365;rs6863893;rs140163909;rs146837743;rs7705919;rs201046346;rs202152005;rs199869120;rs111726541;rs373673987;rs113026830;rs148211606;rs201619508;rs201169921;rs146644914;rs116649742;rs79893293;rs57629433;rs60335509;rs58453996;rs59466242;rs60878839;rs11743452;rs1833567;rs1833568;rs1833569;rs7721272;rs7721427;rs7721730;rs7721870;rs7722100;rs7704359;rs1895200;rs1895201;rs77974856;rs60905342;rs11746207;rs11739672;rs11749718;rs78518991;rs76243602;rs76383235;rs11741980;rs35243960;rs116462975;rs139801813;rs113103584;rs74727230;rs75919325;rs79624714;rs2080858;rs138092354;rs148285468;rs114455395;rs74850689;rs10463599 | NA | 0 |
| 4 | 5:108560001-108600000 | 5:107840001-107880000 | 6.30E-13 | HiC | GSE87112 | IMR90 | intra | rs35812497;rs75803484;rs75073283;rs76430100;rs75782477;rs77992445;rs61701247;rs17161655;rs115979287;rs17161659;rs151072743;rs17161665;rs6863688;5:108591279:C:T;5:108591279:A:T;rs1363213;rs1862201;rs1862202;rs1862203;rs5870359 | NA | 0 |
| 4 | 5:108560001-108600000 | 5:107920001-107960000 | 4.03E-14 | HiC | GSE87112 | IMR90 | intra | rs35812497;rs75803484;rs75073283;rs76430100;rs75782477;rs77992445;rs61701247;rs17161655;rs115979287;rs17161659;rs151072743;rs17161665;rs6863688;5:108591279:C:T;5:108591279:A:T;rs1363213;rs1862201;rs1862202;rs1862203;rs5870359 | NA | 0 |
| 4 | 5:108600001-108640000 | 5:107920001-107960000 | 8.98E-11 | HiC | GSE87112 | IMR90 | intra | rs11744353;rs79776200;rs76069623;rs76043709;rs78729571;rs36123121;rs11748450;rs80234158;rs78146542;rs185072862;rs137867205;rs189006725;rs4388251;rs4438924;rs4541698;rs4388252;rs4392675;rs200252534;rs145520315;rs147699420;rs77803982;rs79469094;rs75077625;rs74379173;rs78664536;rs549536839;rs570631944;rs78784808;rs78340649;rs201569786;rs75649455;rs78814720;rs77561644;rs76565008;rs74436342;rs1592807;rs1592810;rs1592811;rs7700458;rs6594365;rs6863893;rs140163909;rs146837743;rs7705919;rs201046346;rs202152005;rs199869120;rs111726541;rs373673987;rs113026830;rs148211606;rs201619508;rs201169921;rs146644914;rs116649742;rs79893293;rs57629433;rs60335509;rs58453996;rs59466242;rs60878839;rs11743452;rs1833567;rs1833568;rs1833569;rs7721272;rs7721427;rs7721730;rs7721870;rs7722100;rs7704359;rs1895200;rs1895201;rs77974856;rs60905342;rs11746207;rs11739672;rs11749718;rs78518991;rs76243602;rs76383235;rs11741980;rs35243960;rs116462975;rs139801813;rs113103584;rs74727230;rs75919325;rs79624714;rs2080858;rs138092354;rs148285468;rs114455395;rs74850689;rs10463599 | NA | 0 |
| 4 | 5:108560001-108600000 | 5:107960001-108000000 | 2.49E-26 | HiC | GSE87112 | IMR90 | intra | rs35812497;rs75803484;rs75073283;rs76430100;rs75782477;rs77992445;rs61701247;rs17161655;rs115979287;rs17161659;rs151072743;rs17161665;rs6863688;5:108591279:C:T;5:108591279:A:T;rs1363213;rs1862201;rs1862202;rs1862203;rs5870359 | NA | 0 |
| 4 | 5:108600001-108640000 | 5:107960001-108000000 | 2.31E-22 | HiC | GSE87112 | IMR90 | intra | rs11744353;rs79776200;rs76069623;rs76043709;rs78729571;rs36123121;rs11748450;rs80234158;rs78146542;rs185072862;rs137867205;rs189006725;rs4388251;rs4438924;rs4541698;rs4388252;rs4392675;rs200252534;rs145520315;rs147699420;rs77803982;rs79469094;rs75077625;rs74379173;rs78664536;rs549536839;rs570631944;rs78784808;rs78340649;rs201569786;rs75649455;rs78814720;rs77561644;rs76565008;rs74436342;rs1592807;rs1592810;rs1592811;rs7700458;rs6594365;rs6863893;rs140163909;rs146837743;rs7705919;rs201046346;rs202152005;rs199869120;rs111726541;rs373673987;rs113026830;rs148211606;rs201619508;rs201169921;rs146644914;rs116649742;rs79893293;rs57629433;rs60335509;rs58453996;rs59466242;rs60878839;rs11743452;rs1833567;rs1833568;rs1833569;rs7721272;rs7721427;rs7721730;rs7721870;rs7722100;rs7704359;rs1895200;rs1895201;rs77974856;rs60905342;rs11746207;rs11739672;rs11749718;rs78518991;rs76243602;rs76383235;rs11741980;rs35243960;rs116462975;rs139801813;rs113103584;rs74727230;rs75919325;rs79624714;rs2080858;rs138092354;rs148285468;rs114455395;rs74850689;rs10463599 | NA | 0 |
| 4 | 5:108560001-108600000 | 5:108000001-108040000 | 4.53E-09 | HiC | GSE87112 | IMR90 | intra | rs35812497;rs75803484;rs75073283;rs76430100;rs75782477;rs77992445;rs61701247;rs17161655;rs115979287;rs17161659;rs151072743;rs17161665;rs6863688;5:108591279:C:T;5:108591279:A:T;rs1363213;rs1862201;rs1862202;rs1862203;rs5870359 | NA | 0 |
| 4 | 5:108600001-108640000 | 5:108000001-108040000 | 2.34E-07 | HiC | GSE87112 | IMR90 | intra | rs11744353;rs79776200;rs76069623;rs76043709;rs78729571;rs36123121;rs11748450;rs80234158;rs78146542;rs185072862;rs137867205;rs189006725;rs4388251;rs4438924;rs4541698;rs4388252;rs4392675;rs200252534;rs145520315;rs147699420;rs77803982;rs79469094;rs75077625;rs74379173;rs78664536;rs549536839;rs570631944;rs78784808;rs78340649;rs201569786;rs75649455;rs78814720;rs77561644;rs76565008;rs74436342;rs1592807;rs1592810;rs1592811;rs7700458;rs6594365;rs6863893;rs140163909;rs146837743;rs7705919;rs201046346;rs202152005;rs199869120;rs111726541;rs373673987;rs113026830;rs148211606;rs201619508;rs201169921;rs146644914;rs116649742;rs79893293;rs57629433;rs60335509;rs58453996;rs59466242;rs60878839;rs11743452;rs1833567;rs1833568;rs1833569;rs7721272;rs7721427;rs7721730;rs7721870;rs7722100;rs7704359;rs1895200;rs1895201;rs77974856;rs60905342;rs11746207;rs11739672;rs11749718;rs78518991;rs76243602;rs76383235;rs11741980;rs35243960;rs116462975;rs139801813;rs113103584;rs74727230;rs75919325;rs79624714;rs2080858;rs138092354;rs148285468;rs114455395;rs74850689;rs10463599 | NA | 0 |
| 4 | 5:108560001-108600000 | 5:108040001-108080000 | 4.06E-10 | HiC | GSE87112 | IMR90 | intra | rs35812497;rs75803484;rs75073283;rs76430100;rs75782477;rs77992445;rs61701247;rs17161655;rs115979287;rs17161659;rs151072743;rs17161665;rs6863688;5:108591279:C:T;5:108591279:A:T;rs1363213;rs1862201;rs1862202;rs1862203;rs5870359 | NA | 0 |
| 4 | 5:108560001-108600000 | 5:108080001-108120000 | 8.73E-09 | HiC | GSE87112 | IMR90 | intra | rs35812497;rs75803484;rs75073283;rs76430100;rs75782477;rs77992445;rs61701247;rs17161655;rs115979287;rs17161659;rs151072743;rs17161665;rs6863688;5:108591279:C:T;5:108591279:A:T;rs1363213;rs1862201;rs1862202;rs1862203;rs5870359 | ENSG00000151422 | 0 |
| 4 | 5:108560001-108600000 | 5:108200001-108240000 | 4.10E-10 | HiC | GSE87112 | IMR90 | intra | rs35812497;rs75803484;rs75073283;rs76430100;rs75782477;rs77992445;rs61701247;rs17161655;rs115979287;rs17161659;rs151072743;rs17161665;rs6863688;5:108591279:C:T;5:108591279:A:T;rs1363213;rs1862201;rs1862202;rs1862203;rs5870359 | NA | 0 |
| 4 | 5:108600001-108640000 | 5:108200001-108240000 | 3.40E-07 | HiC | GSE87112 | IMR90 | intra | rs11744353;rs79776200;rs76069623;rs76043709;rs78729571;rs36123121;rs11748450;rs80234158;rs78146542;rs185072862;rs137867205;rs189006725;rs4388251;rs4438924;rs4541698;rs4388252;rs4392675;rs200252534;rs145520315;rs147699420;rs77803982;rs79469094;rs75077625;rs74379173;rs78664536;rs549536839;rs570631944;rs78784808;rs78340649;rs201569786;rs75649455;rs78814720;rs77561644;rs76565008;rs74436342;rs1592807;rs1592810;rs1592811;rs7700458;rs6594365;rs6863893;rs140163909;rs146837743;rs7705919;rs201046346;rs202152005;rs199869120;rs111726541;rs373673987;rs113026830;rs148211606;rs201619508;rs201169921;rs146644914;rs116649742;rs79893293;rs57629433;rs60335509;rs58453996;rs59466242;rs60878839;rs11743452;rs1833567;rs1833568;rs1833569;rs7721272;rs7721427;rs7721730;rs7721870;rs7722100;rs7704359;rs1895200;rs1895201;rs77974856;rs60905342;rs11746207;rs11739672;rs11749718;rs78518991;rs76243602;rs76383235;rs11741980;rs35243960;rs116462975;rs139801813;rs113103584;rs74727230;rs75919325;rs79624714;rs2080858;rs138092354;rs148285468;rs114455395;rs74850689;rs10463599 | NA | 0 |
| 4 | 5:108560001-108600000 | 5:108320001-108360000 | 3.04E-18 | HiC | GSE87112 | IMR90 | intra | rs35812497;rs75803484;rs75073283;rs76430100;rs75782477;rs77992445;rs61701247;rs17161655;rs115979287;rs17161659;rs151072743;rs17161665;rs6863688;5:108591279:C:T;5:108591279:A:T;rs1363213;rs1862201;rs1862202;rs1862203;rs5870359 | NA | 0 |
| 4 | 5:108560001-108600000 | 5:108360001-108400000 | 4.85E-07 | HiC | GSE87112 | IMR90 | intra | rs35812497;rs75803484;rs75073283;rs76430100;rs75782477;rs77992445;rs61701247;rs17161655;rs115979287;rs17161659;rs151072743;rs17161665;rs6863688;5:108591279:C:T;5:108591279:A:T;rs1363213;rs1862201;rs1862202;rs1862203;rs5870359 | NA | 0 |
| 4 | 5:108560001-108600000 | 5:108520001-108560000 | 2.84E-16 | HiC | GSE87112 | IMR90 | intra | rs35812497;rs75803484;rs75073283;rs76430100;rs75782477;rs77992445;rs61701247;rs17161655;rs115979287;rs17161659;rs151072743;rs17161665;rs6863688;5:108591279:C:T;5:108591279:A:T;rs1363213;rs1862201;rs1862202;rs1862203;rs5870359 | NA | 0 |
| 5 | 11:2520001-2560000 | 11:2160001-2200000 | 4.79E-10 | HiC | GSE87112 | IMR90 | intra | rs111815403 | ENSG00000167244:ENSG00000129965:ENSG00000254647:ENSG00000180176 | 0 |
| 5 | 11:2520001-2560000 | 11:2200001-2240000 | 8.06E-19 | HiC | GSE87112 | IMR90 | intra | rs111815403 | NA | 0 |
| 5 | 11:2520001-2560000 | 11:2240001-2280000 | 1.88E-31 | HiC | GSE87112 | IMR90 | intra | rs111815403 | NA | 0 |
| 5 | 11:2520001-2560000 | 11:2280001-2320000 | 1.77E-35 | HiC | GSE87112 | IMR90 | intra | rs111815403 | ENSG00000183734 | 1 |
| 6 | 12:80800001-80840000 | 12:78400001-78440000 | 2.29E-12 | HiC | GSE87112 | IMR90 | intra | rs117203215;rs77741796 | NA | 0 |
| 6 | 12:80800001-80840000 | 12:78480001-78520000 | 1.49E-09 | HiC | GSE87112 | IMR90 | intra | rs117203215;rs77741796 | NA | 0 |
| 6 | 12:80800001-80840000 | 12:78560001-78600000 | 4.26E-07 | HiC | GSE87112 | IMR90 | intra | rs117203215;rs77741796 | NA | 0 |
| 6 | 12:80640001-80680000 | 12:78640001-78680000 | 2.44E-07 | HiC | GSE87112 | IMR90 | intra | rs201272756;rs202070984 | NA | 0 |
| 6 | 12:80800001-80840000 | 12:78640001-78680000 | 7.41E-07 | HiC | GSE87112 | IMR90 | intra | rs117203215;rs77741796 | NA | 0 |
| 6 | 12:80800001-80840000 | 12:78960001-79000000 | 3.34E-07 | HiC | GSE87112 | IMR90 | intra | rs117203215;rs77741796 | NA | 0 |
| 6 | 12:80800001-80840000 | 12:79040001-79080000 | 2.07E-10 | HiC | GSE87112 | IMR90 | intra | rs117203215;rs77741796 | NA | 0 |
| 6 | 12:80800001-80840000 | 12:79240001-79280000 | 1.85E-08 | HiC | GSE87112 | IMR90 | intra | rs117203215;rs77741796 | ENSG00000067715 | 0 |
| 6 | 12:80800001-80840000 | 12:79320001-79360000 | 8.62E-11 | HiC | GSE87112 | IMR90 | intra | rs117203215;rs77741796 | NA | 0 |
| 6 | 12:80800001-80840000 | 12:79680001-79720000 | 8.58E-07 | HiC | GSE87112 | IMR90 | intra | rs117203215;rs77741796 | NA | 0 |
| 6 | 12:80640001-80680000 | 12:79760001-79800000 | 1.67E-25 | HiC | GSE87112 | IMR90 | intra | rs201272756;rs202070984 | NA | 0 |
| 6 | 12:80800001-80840000 | 12:79760001-79800000 | 1.14E-23 | HiC | GSE87112 | IMR90 | intra | rs117203215;rs77741796 | NA | 0 |
| 6 | 12:80640001-80680000 | 12:79800001-79840000 | 2.74E-08 | HiC | GSE87112 | IMR90 | intra | rs201272756;rs202070984 | NA | 0 |
| 6 | 12:80840001-80880000 | 12:79800001-79840000 | 3.65E-08 | HiC | GSE87112 | IMR90 | intra | rs200589835 | NA | 0 |
| 6 | 12:80640001-80680000 | 12:79920001-79960000 | 3.14E-12 | HiC | GSE87112 | IMR90 | intra | rs201272756;rs202070984 | NA | 0 |
| 6 | 12:80800001-80840000 | 12:79920001-79960000 | 1.80E-21 | HiC | GSE87112 | IMR90 | intra | rs117203215;rs77741796 | NA | 0 |
| 6 | 12:80840001-80880000 | 12:79920001-79960000 | 5.10E-08 | HiC | GSE87112 | IMR90 | intra | rs200589835 | NA | 0 |
| 6 | 12:80800001-80840000 | 12:79960001-80000000 | 1.49E-07 | HiC | GSE87112 | IMR90 | intra | rs117203215;rs77741796 | NA | 0 |
| 6 | 12:80640001-80680000 | 12:80040001-80080000 | 6.37E-10 | HiC | GSE87112 | IMR90 | intra | rs201272756;rs202070984 | NA | 0 |
| 6 | 12:80800001-80840000 | 12:80040001-80080000 | 4.31E-11 | HiC | GSE87112 | IMR90 | intra | rs117203215;rs77741796 | NA | 0 |
| 6 | 12:80800001-80840000 | 12:80120001-80160000 | 1.11E-09 | HiC | GSE87112 | IMR90 | intra | rs117203215;rs77741796 | NA | 0 |
| 6 | 12:80800001-80840000 | 12:80160001-80200000 | 4.26E-07 | HiC | GSE87112 | IMR90 | intra | rs117203215;rs77741796 | ENSG00000269531 | 0 |
| 6 | 12:80800001-80840000 | 12:80560001-80600000 | 4.37E-15 | HiC | GSE87112 | IMR90 | intra | rs117203215;rs77741796 | NA | 0 |
| 6 | 12:80800001-80840000 | 12:80680001-80720000 | 2.97E-07 | HiC | GSE87112 | IMR90 | intra | rs117203215;rs77741796 | NA | 0 |
| 6 | 12:80800001-80840000 | 12:80720001-80760000 | 6.07E-10 | HiC | GSE87112 | IMR90 | intra | rs117203215;rs77741796 | NA | 0 |
| 7 | 12:127760001-127800000 | 12:125640001-125680000 | 2.29E-09 | HiC | GSE87112 | IMR90 | intra | rs1810088;rs367839627;rs146043604;rs143439170;rs10773382;rs10847307;rs35154640;rs1552386;rs7314186;rs1979065;rs5801722;rs10744302;12:127775513:A:C;rs10734938;rs35024927;rs10773387;rs978813;rs2348321;rs10847321;rs10847323;rs6489163 | ENSG00000139364 | 0 |
| 7 | 12:127760001-127800000 | 12:125680001-125720000 | 2.96E-20 | HiC | GSE87112 | IMR90 | intra | rs1810088;rs367839627;rs146043604;rs143439170;rs10773382;rs10847307;rs35154640;rs1552386;rs7314186;rs1979065;rs5801722;rs10744302;12:127775513:A:C;rs10734938;rs35024927;rs10773387;rs978813;rs2348321;rs10847321;rs10847323;rs6489163 | NA | 0 |
| 7 | 12:127760001-127800000 | 12:125720001-125760000 | 5.91E-12 | HiC | GSE87112 | IMR90 | intra | rs1810088;rs367839627;rs146043604;rs143439170;rs10773382;rs10847307;rs35154640;rs1552386;rs7314186;rs1979065;rs5801722;rs10744302;12:127775513:A:C;rs10734938;rs35024927;rs10773387;rs978813;rs2348321;rs10847321;rs10847323;rs6489163 | NA | 0 |
| 7 | 12:127760001-127800000 | 12:125760001-125800000 | 3.09E-16 | HiC | GSE87112 | IMR90 | intra | rs1810088;rs367839627;rs146043604;rs143439170;rs10773382;rs10847307;rs35154640;rs1552386;rs7314186;rs1979065;rs5801722;rs10744302;12:127775513:A:C;rs10734938;rs35024927;rs10773387;rs978813;rs2348321;rs10847321;rs10847323;rs6489163 | NA | 0 |
| 7 | 12:127760001-127800000 | 12:125800001-125840000 | 9.94E-19 | HiC | GSE87112 | IMR90 | intra | rs1810088;rs367839627;rs146043604;rs143439170;rs10773382;rs10847307;rs35154640;rs1552386;rs7314186;rs1979065;rs5801722;rs10744302;12:127775513:A:C;rs10734938;rs35024927;rs10773387;rs978813;rs2348321;rs10847321;rs10847323;rs6489163 | NA | 0 |
| 7 | 12:127760001-127800000 | 12:125840001-125880000 | 1.50E-12 | HiC | GSE87112 | IMR90 | intra | rs1810088;rs367839627;rs146043604;rs143439170;rs10773382;rs10847307;rs35154640;rs1552386;rs7314186;rs1979065;rs5801722;rs10744302;12:127775513:A:C;rs10734938;rs35024927;rs10773387;rs978813;rs2348321;rs10847321;rs10847323;rs6489163 | NA | 0 |
| 7 | 12:127760001-127800000 | 12:125880001-125920000 | 2.25E-07 | HiC | GSE87112 | IMR90 | intra | rs1810088;rs367839627;rs146043604;rs143439170;rs10773382;rs10847307;rs35154640;rs1552386;rs7314186;rs1979065;rs5801722;rs10744302;12:127775513:A:C;rs10734938;rs35024927;rs10773387;rs978813;rs2348321;rs10847321;rs10847323;rs6489163 | NA | 0 |
| 7 | 12:127760001-127800000 | 12:125960001-126000000 | 1.29E-13 | HiC | GSE87112 | IMR90 | intra | rs1810088;rs367839627;rs146043604;rs143439170;rs10773382;rs10847307;rs35154640;rs1552386;rs7314186;rs1979065;rs5801722;rs10744302;12:127775513:A:C;rs10734938;rs35024927;rs10773387;rs978813;rs2348321;rs10847321;rs10847323;rs6489163 | NA | 0 |
| 7 | 12:127760001-127800000 | 12:126000001-126040000 | 3.50E-15 | HiC | GSE87112 | IMR90 | intra | rs1810088;rs367839627;rs146043604;rs143439170;rs10773382;rs10847307;rs35154640;rs1552386;rs7314186;rs1979065;rs5801722;rs10744302;12:127775513:A:C;rs10734938;rs35024927;rs10773387;rs978813;rs2348321;rs10847321;rs10847323;rs6489163 | NA | 0 |
| 7 | 12:127760001-127800000 | 12:126040001-126080000 | 2.13E-31 | HiC | GSE87112 | IMR90 | intra | rs1810088;rs367839627;rs146043604;rs143439170;rs10773382;rs10847307;rs35154640;rs1552386;rs7314186;rs1979065;rs5801722;rs10744302;12:127775513:A:C;rs10734938;rs35024927;rs10773387;rs978813;rs2348321;rs10847321;rs10847323;rs6489163 | NA | 0 |
| 7 | 12:127760001-127800000 | 12:126080001-126120000 | 4.34E-12 | HiC | GSE87112 | IMR90 | intra | rs1810088;rs367839627;rs146043604;rs143439170;rs10773382;rs10847307;rs35154640;rs1552386;rs7314186;rs1979065;rs5801722;rs10744302;12:127775513:A:C;rs10734938;rs35024927;rs10773387;rs978813;rs2348321;rs10847321;rs10847323;rs6489163 | NA | 0 |
| 7 | 12:127760001-127800000 | 12:126120001-126160000 | 2.07E-23 | HiC | GSE87112 | IMR90 | intra | rs1810088;rs367839627;rs146043604;rs143439170;rs10773382;rs10847307;rs35154640;rs1552386;rs7314186;rs1979065;rs5801722;rs10744302;12:127775513:A:C;rs10734938;rs35024927;rs10773387;rs978813;rs2348321;rs10847321;rs10847323;rs6489163 | NA | 0 |
| 7 | 12:127760001-127800000 | 12:126160001-126200000 | 5.79E-12 | HiC | GSE87112 | IMR90 | intra | rs1810088;rs367839627;rs146043604;rs143439170;rs10773382;rs10847307;rs35154640;rs1552386;rs7314186;rs1979065;rs5801722;rs10744302;12:127775513:A:C;rs10734938;rs35024927;rs10773387;rs978813;rs2348321;rs10847321;rs10847323;rs6489163 | NA | 0 |
| 7 | 12:127760001-127800000 | 12:126200001-126240000 | 1.39E-17 | HiC | GSE87112 | IMR90 | intra | rs1810088;rs367839627;rs146043604;rs143439170;rs10773382;rs10847307;rs35154640;rs1552386;rs7314186;rs1979065;rs5801722;rs10744302;12:127775513:A:C;rs10734938;rs35024927;rs10773387;rs978813;rs2348321;rs10847321;rs10847323;rs6489163 | NA | 0 |
| 7 | 12:127760001-127800000 | 12:126240001-126280000 | 4.56E-17 | HiC | GSE87112 | IMR90 | intra | rs1810088;rs367839627;rs146043604;rs143439170;rs10773382;rs10847307;rs35154640;rs1552386;rs7314186;rs1979065;rs5801722;rs10744302;12:127775513:A:C;rs10734938;rs35024927;rs10773387;rs978813;rs2348321;rs10847321;rs10847323;rs6489163 | NA | 0 |
| 7 | 12:127760001-127800000 | 12:126280001-126320000 | 5.34E-39 | HiC | GSE87112 | IMR90 | intra | rs1810088;rs367839627;rs146043604;rs143439170;rs10773382;rs10847307;rs35154640;rs1552386;rs7314186;rs1979065;rs5801722;rs10744302;12:127775513:A:C;rs10734938;rs35024927;rs10773387;rs978813;rs2348321;rs10847321;rs10847323;rs6489163 | NA | 0 |
| 7 | 12:127760001-127800000 | 12:126360001-126400000 | 7.97E-18 | HiC | GSE87112 | IMR90 | intra | rs1810088;rs367839627;rs146043604;rs143439170;rs10773382;rs10847307;rs35154640;rs1552386;rs7314186;rs1979065;rs5801722;rs10744302;12:127775513:A:C;rs10734938;rs35024927;rs10773387;rs978813;rs2348321;rs10847321;rs10847323;rs6489163 | NA | 0 |
| 7 | 12:127760001-127800000 | 12:126400001-126440000 | 1.38E-12 | HiC | GSE87112 | IMR90 | intra | rs1810088;rs367839627;rs146043604;rs143439170;rs10773382;rs10847307;rs35154640;rs1552386;rs7314186;rs1979065;rs5801722;rs10744302;12:127775513:A:C;rs10734938;rs35024927;rs10773387;rs978813;rs2348321;rs10847321;rs10847323;rs6489163 | NA | 0 |
| 7 | 12:127760001-127800000 | 12:126440001-126480000 | 2.85E-07 | HiC | GSE87112 | IMR90 | intra | rs1810088;rs367839627;rs146043604;rs143439170;rs10773382;rs10847307;rs35154640;rs1552386;rs7314186;rs1979065;rs5801722;rs10744302;12:127775513:A:C;rs10734938;rs35024927;rs10773387;rs978813;rs2348321;rs10847321;rs10847323;rs6489163 | NA | 0 |
| 7 | 12:127760001-127800000 | 12:126480001-126520000 | 1.03E-28 | HiC | GSE87112 | IMR90 | intra | rs1810088;rs367839627;rs146043604;rs143439170;rs10773382;rs10847307;rs35154640;rs1552386;rs7314186;rs1979065;rs5801722;rs10744302;12:127775513:A:C;rs10734938;rs35024927;rs10773387;rs978813;rs2348321;rs10847321;rs10847323;rs6489163 | NA | 0 |
| 7 | 12:127760001-127800000 | 12:126520001-126560000 | 2.48E-26 | HiC | GSE87112 | IMR90 | intra | rs1810088;rs367839627;rs146043604;rs143439170;rs10773382;rs10847307;rs35154640;rs1552386;rs7314186;rs1979065;rs5801722;rs10744302;12:127775513:A:C;rs10734938;rs35024927;rs10773387;rs978813;rs2348321;rs10847321;rs10847323;rs6489163 | NA | 0 |
| 7 | 12:127760001-127800000 | 12:126560001-126600000 | 9.64E-23 | HiC | GSE87112 | IMR90 | intra | rs1810088;rs367839627;rs146043604;rs143439170;rs10773382;rs10847307;rs35154640;rs1552386;rs7314186;rs1979065;rs5801722;rs10744302;12:127775513:A:C;rs10734938;rs35024927;rs10773387;rs978813;rs2348321;rs10847321;rs10847323;rs6489163 | NA | 0 |
| 7 | 12:127760001-127800000 | 12:126640001-126680000 | 4.46E-13 | HiC | GSE87112 | IMR90 | intra | rs1810088;rs367839627;rs146043604;rs143439170;rs10773382;rs10847307;rs35154640;rs1552386;rs7314186;rs1979065;rs5801722;rs10744302;12:127775513:A:C;rs10734938;rs35024927;rs10773387;rs978813;rs2348321;rs10847321;rs10847323;rs6489163 | NA | 0 |
| 7 | 12:127760001-127800000 | 12:126680001-126720000 | 1.10E-26 | HiC | GSE87112 | IMR90 | intra | rs1810088;rs367839627;rs146043604;rs143439170;rs10773382;rs10847307;rs35154640;rs1552386;rs7314186;rs1979065;rs5801722;rs10744302;12:127775513:A:C;rs10734938;rs35024927;rs10773387;rs978813;rs2348321;rs10847321;rs10847323;rs6489163 | NA | 0 |
| 7 | 12:127760001-127800000 | 12:126720001-126760000 | 9.55E-33 | HiC | GSE87112 | IMR90 | intra | rs1810088;rs367839627;rs146043604;rs143439170;rs10773382;rs10847307;rs35154640;rs1552386;rs7314186;rs1979065;rs5801722;rs10744302;12:127775513:A:C;rs10734938;rs35024927;rs10773387;rs978813;rs2348321;rs10847321;rs10847323;rs6489163 | NA | 0 |
| 7 | 12:127760001-127800000 | 12:126800001-126840000 | 4.14E-28 | HiC | GSE87112 | IMR90 | intra | rs1810088;rs367839627;rs146043604;rs143439170;rs10773382;rs10847307;rs35154640;rs1552386;rs7314186;rs1979065;rs5801722;rs10744302;12:127775513:A:C;rs10734938;rs35024927;rs10773387;rs978813;rs2348321;rs10847321;rs10847323;rs6489163 | NA | 0 |
| 7 | 12:127760001-127800000 | 12:126840001-126880000 | 2.85E-07 | HiC | GSE87112 | IMR90 | intra | rs1810088;rs367839627;rs146043604;rs143439170;rs10773382;rs10847307;rs35154640;rs1552386;rs7314186;rs1979065;rs5801722;rs10744302;12:127775513:A:C;rs10734938;rs35024927;rs10773387;rs978813;rs2348321;rs10847321;rs10847323;rs6489163 | NA | 0 |
| 7 | 12:127760001-127800000 | 12:126880001-126920000 | 2.60E-32 | HiC | GSE87112 | IMR90 | intra | rs1810088;rs367839627;rs146043604;rs143439170;rs10773382;rs10847307;rs35154640;rs1552386;rs7314186;rs1979065;rs5801722;rs10744302;12:127775513:A:C;rs10734938;rs35024927;rs10773387;rs978813;rs2348321;rs10847321;rs10847323;rs6489163 | NA | 0 |
| 7 | 12:127760001-127800000 | 12:126920001-126960000 | 2.74E-08 | HiC | GSE87112 | IMR90 | intra | rs1810088;rs367839627;rs146043604;rs143439170;rs10773382;rs10847307;rs35154640;rs1552386;rs7314186;rs1979065;rs5801722;rs10744302;12:127775513:A:C;rs10734938;rs35024927;rs10773387;rs978813;rs2348321;rs10847321;rs10847323;rs6489163 | NA | 0 |
| 7 | 12:127760001-127800000 | 12:126960001-127000000 | 1.06E-25 | HiC | GSE87112 | IMR90 | intra | rs1810088;rs367839627;rs146043604;rs143439170;rs10773382;rs10847307;rs35154640;rs1552386;rs7314186;rs1979065;rs5801722;rs10744302;12:127775513:A:C;rs10734938;rs35024927;rs10773387;rs978813;rs2348321;rs10847321;rs10847323;rs6489163 | NA | 0 |
| 7 | 12:127760001-127800000 | 12:127000001-127040000 | 1.69E-09 | HiC | GSE87112 | IMR90 | intra | rs1810088;rs367839627;rs146043604;rs143439170;rs10773382;rs10847307;rs35154640;rs1552386;rs7314186;rs1979065;rs5801722;rs10744302;12:127775513:A:C;rs10734938;rs35024927;rs10773387;rs978813;rs2348321;rs10847321;rs10847323;rs6489163 | NA | 0 |
| 7 | 12:127760001-127800000 | 12:127040001-127080000 | 5.38E-11 | HiC | GSE87112 | IMR90 | intra | rs1810088;rs367839627;rs146043604;rs143439170;rs10773382;rs10847307;rs35154640;rs1552386;rs7314186;rs1979065;rs5801722;rs10744302;12:127775513:A:C;rs10734938;rs35024927;rs10773387;rs978813;rs2348321;rs10847321;rs10847323;rs6489163 | NA | 0 |
| 7 | 12:127760001-127800000 | 12:127080001-127120000 | 8.82E-19 | HiC | GSE87112 | IMR90 | intra | rs1810088;rs367839627;rs146043604;rs143439170;rs10773382;rs10847307;rs35154640;rs1552386;rs7314186;rs1979065;rs5801722;rs10744302;12:127775513:A:C;rs10734938;rs35024927;rs10773387;rs978813;rs2348321;rs10847321;rs10847323;rs6489163 | NA | 0 |
| 7 | 12:127760001-127800000 | 12:127120001-127160000 | 4.26E-07 | HiC | GSE87112 | IMR90 | intra | rs1810088;rs367839627;rs146043604;rs143439170;rs10773382;rs10847307;rs35154640;rs1552386;rs7314186;rs1979065;rs5801722;rs10744302;12:127775513:A:C;rs10734938;rs35024927;rs10773387;rs978813;rs2348321;rs10847321;rs10847323;rs6489163 | NA | 0 |
| 7 | 12:127760001-127800000 | 12:127160001-127200000 | 3.27E-09 | HiC | GSE87112 | IMR90 | intra | rs1810088;rs367839627;rs146043604;rs143439170;rs10773382;rs10847307;rs35154640;rs1552386;rs7314186;rs1979065;rs5801722;rs10744302;12:127775513:A:C;rs10734938;rs35024927;rs10773387;rs978813;rs2348321;rs10847321;rs10847323;rs6489163 | NA | 0 |
| 7 | 12:127760001-127800000 | 12:127200001-127240000 | 8.32E-21 | HiC | GSE87112 | IMR90 | intra | rs1810088;rs367839627;rs146043604;rs143439170;rs10773382;rs10847307;rs35154640;rs1552386;rs7314186;rs1979065;rs5801722;rs10744302;12:127775513:A:C;rs10734938;rs35024927;rs10773387;rs978813;rs2348321;rs10847321;rs10847323;rs6489163 | NA | 0 |
| 7 | 12:127760001-127800000 | 12:127240001-127280000 | 6.05E-20 | HiC | GSE87112 | IMR90 | intra | rs1810088;rs367839627;rs146043604;rs143439170;rs10773382;rs10847307;rs35154640;rs1552386;rs7314186;rs1979065;rs5801722;rs10744302;12:127775513:A:C;rs10734938;rs35024927;rs10773387;rs978813;rs2348321;rs10847321;rs10847323;rs6489163 | NA | 0 |
| 7 | 12:127760001-127800000 | 12:127280001-127320000 | 6.88E-20 | HiC | GSE87112 | IMR90 | intra | rs1810088;rs367839627;rs146043604;rs143439170;rs10773382;rs10847307;rs35154640;rs1552386;rs7314186;rs1979065;rs5801722;rs10744302;12:127775513:A:C;rs10734938;rs35024927;rs10773387;rs978813;rs2348321;rs10847321;rs10847323;rs6489163 | NA | 0 |
| 7 | 12:127760001-127800000 | 12:127360001-127400000 | 5.63E-25 | HiC | GSE87112 | IMR90 | intra | rs1810088;rs367839627;rs146043604;rs143439170;rs10773382;rs10847307;rs35154640;rs1552386;rs7314186;rs1979065;rs5801722;rs10744302;12:127775513:A:C;rs10734938;rs35024927;rs10773387;rs978813;rs2348321;rs10847321;rs10847323;rs6489163 | NA | 0 |
| 7 | 12:127760001-127800000 | 12:127400001-127440000 | 5.18E-11 | HiC | GSE87112 | IMR90 | intra | rs1810088;rs367839627;rs146043604;rs143439170;rs10773382;rs10847307;rs35154640;rs1552386;rs7314186;rs1979065;rs5801722;rs10744302;12:127775513:A:C;rs10734938;rs35024927;rs10773387;rs978813;rs2348321;rs10847321;rs10847323;rs6489163 | NA | 0 |
| 7 | 12:127760001-127800000 | 12:127440001-127480000 | 5.22E-09 | HiC | GSE87112 | IMR90 | intra | rs1810088;rs367839627;rs146043604;rs143439170;rs10773382;rs10847307;rs35154640;rs1552386;rs7314186;rs1979065;rs5801722;rs10744302;12:127775513:A:C;rs10734938;rs35024927;rs10773387;rs978813;rs2348321;rs10847321;rs10847323;rs6489163 | NA | 0 |
| 7 | 12:127760001-127800000 | 12:127480001-127520000 | 3.49E-10 | HiC | GSE87112 | IMR90 | intra | rs1810088;rs367839627;rs146043604;rs143439170;rs10773382;rs10847307;rs35154640;rs1552386;rs7314186;rs1979065;rs5801722;rs10744302;12:127775513:A:C;rs10734938;rs35024927;rs10773387;rs978813;rs2348321;rs10847321;rs10847323;rs6489163 | NA | 0 |
| 7 | 12:127760001-127800000 | 12:127560001-127600000 | 1.27E-32 | HiC | GSE87112 | IMR90 | intra | rs1810088;rs367839627;rs146043604;rs143439170;rs10773382;rs10847307;rs35154640;rs1552386;rs7314186;rs1979065;rs5801722;rs10744302;12:127775513:A:C;rs10734938;rs35024927;rs10773387;rs978813;rs2348321;rs10847321;rs10847323;rs6489163 | NA | 0 |
| 7 | 12:127760001-127800000 | 12:127600001-127640000 | 2.17E-19 | HiC | GSE87112 | IMR90 | intra | rs1810088;rs367839627;rs146043604;rs143439170;rs10773382;rs10847307;rs35154640;rs1552386;rs7314186;rs1979065;rs5801722;rs10744302;12:127775513:A:C;rs10734938;rs35024927;rs10773387;rs978813;rs2348321;rs10847321;rs10847323;rs6489163 | NA | 0 |
| 7 | 12:127760001-127800000 | 12:127640001-127680000 | 1.87E-08 | HiC | GSE87112 | IMR90 | intra | rs1810088;rs367839627;rs146043604;rs143439170;rs10773382;rs10847307;rs35154640;rs1552386;rs7314186;rs1979065;rs5801722;rs10744302;12:127775513:A:C;rs10734938;rs35024927;rs10773387;rs978813;rs2348321;rs10847321;rs10847323;rs6489163 | NA | 0 |
| 7 | 12:127760001-127800000 | 12:127680001-127720000 | 9.68E-10 | HiC | GSE87112 | IMR90 | intra | rs1810088;rs367839627;rs146043604;rs143439170;rs10773382;rs10847307;rs35154640;rs1552386;rs7314186;rs1979065;rs5801722;rs10744302;12:127775513:A:C;rs10734938;rs35024927;rs10773387;rs978813;rs2348321;rs10847321;rs10847323;rs6489163 | NA | 0 |
| 7 | 12:127760001-127800000 | 12:127720001-127760000 | 5.03E-39 | HiC | GSE87112 | IMR90 | intra | rs1810088;rs367839627;rs146043604;rs143439170;rs10773382;rs10847307;rs35154640;rs1552386;rs7314186;rs1979065;rs5801722;rs10744302;12:127775513:A:C;rs10734938;rs35024927;rs10773387;rs978813;rs2348321;rs10847321;rs10847323;rs6489163 | NA | 0 |
| 1 | 3:37520001-37560000 | 3:37720001-37760000 | 5.11E-10 | HiC | GSE87112 | Mesenchymal_Stem_Cell | intra | rs113897538;rs2507941;rs17814364;rs112038297;rs78544469;rs77422813;rs17228684;rs928799;rs75425697;rs5848026;rs17814903;rs12054441 | NA | 0 |
| 1 | 3:37520001-37560000 | 3:37760001-37800000 | 1.97E-17 | HiC | GSE87112 | Mesenchymal_Stem_Cell | intra | rs113897538;rs2507941;rs17814364;rs112038297;rs78544469;rs77422813;rs17228684;rs928799;rs75425697;rs5848026;rs17814903;rs12054441 | NA | 0 |
| 1 | 3:37520001-37560000 | 3:37800001-37840000 | 6.57E-14 | HiC | GSE87112 | Mesenchymal_Stem_Cell | intra | rs113897538;rs2507941;rs17814364;rs112038297;rs78544469;rs77422813;rs17228684;rs928799;rs75425697;rs5848026;rs17814903;rs12054441 | NA | 0 |
| 1 | 3:37560001-37600000 | 3:37720001-37760000 | 5.69E-15 | HiC | GSE87112 | Mesenchymal_Stem_Cell | intra | rs74985154;rs3733138;rs3733140;rs75619156;rs17229924;rs2162356;rs74786716;rs77820597;rs112585731;rs915631;rs79149273;rs17230261;rs199607377;rs2162355;rs76952775;rs75469174;rs78210776;rs77162103;rs75217006;rs78292414 | NA | 0 |
| 1 | 3:37560001-37600000 | 3:37760001-37800000 | 5.61E-28 | HiC | GSE87112 | Mesenchymal_Stem_Cell | intra | rs74985154;rs3733138;rs3733140;rs75619156;rs17229924;rs2162356;rs74786716;rs77820597;rs112585731;rs915631;rs79149273;rs17230261;rs199607377;rs2162355;rs76952775;rs75469174;rs78210776;rs77162103;rs75217006;rs78292414 | NA | 0 |
| 1 | 3:37560001-37600000 | 3:37800001-37840000 | 1.71E-07 | HiC | GSE87112 | Mesenchymal_Stem_Cell | intra | rs74985154;rs3733138;rs3733140;rs75619156;rs17229924;rs2162356;rs74786716;rs77820597;rs112585731;rs915631;rs79149273;rs17230261;rs199607377;rs2162355;rs76952775;rs75469174;rs78210776;rs77162103;rs75217006;rs78292414 | NA | 0 |
| 2 | 3:96520001-96560000 | 3:97360001-97400000 | 1.38E-07 | HiC | GSE87112 | Mesenchymal_Stem_Cell | intra | rs62262901;rs62262902;rs62262903;rs62262930;rs151165295;rs62262931;rs62262933;rs62262934;rs74641574;rs79645223;rs2318156;rs2318155;rs2318154 | NA | 0 |
| 2 | 3:96520001-96560000 | 3:97720001-97760000 | 7.14E-12 | HiC | GSE87112 | Mesenchymal_Stem_Cell | intra | rs62262901;rs62262902;rs62262903;rs62262930;rs151165295;rs62262931;rs62262933;rs62262934;rs74641574;rs79645223;rs2318156;rs2318155;rs2318154 | NA | 0 |
| 2 | 3:96560001-96600000 | 3:97360001-97400000 | 8.21E-08 | HiC | GSE87112 | Mesenchymal_Stem_Cell | intra | rs16836353;rs62262935;rs62262936;rs62262937;rs62262938;rs62262940;rs62262941;rs62262942;rs62262944;rs62262946;rs62262947;rs62262948;rs62262949;rs62262950;rs62262951 | NA | 0 |
| 2 | 3:96560001-96600000 | 3:97400001-97440000 | 5.97E-07 | HiC | GSE87112 | Mesenchymal_Stem_Cell | intra | rs16836353;rs62262935;rs62262936;rs62262937;rs62262938;rs62262940;rs62262941;rs62262942;rs62262944;rs62262946;rs62262947;rs62262948;rs62262949;rs62262950;rs62262951 | NA | 0 |
| 2 | 3:96560001-96600000 | 3:97720001-97760000 | 7.23E-08 | HiC | GSE87112 | Mesenchymal_Stem_Cell | intra | rs16836353;rs62262935;rs62262936;rs62262937;rs62262938;rs62262940;rs62262941;rs62262942;rs62262944;rs62262946;rs62262947;rs62262948;rs62262949;rs62262950;rs62262951 | NA | 0 |
| 2 | 3:96600001-96640000 | 3:96680001-96720000 | 9.63E-08 | HiC | GSE87112 | Mesenchymal_Stem_Cell | intra | rs62262952;rs62262953;rs116253310;rs76857958;rs111706803;rs62262955;rs62262957;rs62262958;rs62263676;rs187619159;rs62263677;rs62263678;rs574559070;rs534937763;rs62263680;rs62263682;rs62263684;rs16836982;rs16836986;rs112527011;rs62263685;rs62263686 | NA | 0 |
| 2 | 3:96600001-96640000 | 3:97120001-97160000 | 6.92E-10 | HiC | GSE87112 | Mesenchymal_Stem_Cell | intra | rs62262952;rs62262953;rs116253310;rs76857958;rs111706803;rs62262955;rs62262957;rs62262958;rs62263676;rs187619159;rs62263677;rs62263678;rs574559070;rs534937763;rs62263680;rs62263682;rs62263684;rs16836982;rs16836986;rs112527011;rs62263685;rs62263686 | NA | 0 |
| 2 | 3:96600001-96640000 | 3:97200001-97240000 | 3.06E-08 | HiC | GSE87112 | Mesenchymal_Stem_Cell | intra | rs62262952;rs62262953;rs116253310;rs76857958;rs111706803;rs62262955;rs62262957;rs62262958;rs62263676;rs187619159;rs62263677;rs62263678;rs574559070;rs534937763;rs62263680;rs62263682;rs62263684;rs16836982;rs16836986;rs112527011;rs62263685;rs62263686 | NA | 0 |
| 2 | 3:96600001-96640000 | 3:97280001-97320000 | 1.26E-16 | HiC | GSE87112 | Mesenchymal_Stem_Cell | intra | rs62262952;rs62262953;rs116253310;rs76857958;rs111706803;rs62262955;rs62262957;rs62262958;rs62263676;rs187619159;rs62263677;rs62263678;rs574559070;rs534937763;rs62263680;rs62263682;rs62263684;rs16836982;rs16836986;rs112527011;rs62263685;rs62263686 | NA | 0 |
| 2 | 3:96600001-96640000 | 3:97360001-97400000 | 2.15E-15 | HiC | GSE87112 | Mesenchymal_Stem_Cell | intra | rs62262952;rs62262953;rs116253310;rs76857958;rs111706803;rs62262955;rs62262957;rs62262958;rs62263676;rs187619159;rs62263677;rs62263678;rs574559070;rs534937763;rs62263680;rs62263682;rs62263684;rs16836982;rs16836986;rs112527011;rs62263685;rs62263686 | NA | 0 |
| 2 | 3:96600001-96640000 | 3:97440001-97480000 | 5.97E-07 | HiC | GSE87112 | Mesenchymal_Stem_Cell | intra | rs62262952;rs62262953;rs116253310;rs76857958;rs111706803;rs62262955;rs62262957;rs62262958;rs62263676;rs187619159;rs62263677;rs62263678;rs574559070;rs534937763;rs62263680;rs62263682;rs62263684;rs16836982;rs16836986;rs112527011;rs62263685;rs62263686 | NA | 0 |
| 2 | 3:96680001-96720000 | 3:96920001-96960000 | 2.32E-11 | HiC | GSE87112 | Mesenchymal_Stem_Cell | intra | rs62263705;rs62263706;rs6762348;rs62263709;rs137983052;rs62263734;rs2856474;rs2612274;rs553308194 | NA | 0 |
| 2 | 3:96680001-96720000 | 3:97080001-97120000 | 3.17E-14 | HiC | GSE87112 | Mesenchymal_Stem_Cell | intra | rs62263705;rs62263706;rs6762348;rs62263709;rs137983052;rs62263734;rs2856474;rs2612274;rs553308194 | NA | 0 |
| 2 | 3:96680001-96720000 | 3:97120001-97160000 | 6.74E-19 | HiC | GSE87112 | Mesenchymal_Stem_Cell | intra | rs62263705;rs62263706;rs6762348;rs62263709;rs137983052;rs62263734;rs2856474;rs2612274;rs553308194 | NA | 0 |
| 2 | 3:96680001-96720000 | 3:97200001-97240000 | 1.70E-10 | HiC | GSE87112 | Mesenchymal_Stem_Cell | intra | rs62263705;rs62263706;rs6762348;rs62263709;rs137983052;rs62263734;rs2856474;rs2612274;rs553308194 | NA | 0 |
| 2 | 3:96680001-96720000 | 3:97240001-97280000 | 5.26E-08 | HiC | GSE87112 | Mesenchymal_Stem_Cell | intra | rs62263705;rs62263706;rs6762348;rs62263709;rs137983052;rs62263734;rs2856474;rs2612274;rs553308194 | NA | 0 |
| 2 | 3:96680001-96720000 | 3:97280001-97320000 | 2.25E-23 | HiC | GSE87112 | Mesenchymal_Stem_Cell | intra | rs62263705;rs62263706;rs6762348;rs62263709;rs137983052;rs62263734;rs2856474;rs2612274;rs553308194 | NA | 0 |
| 2 | 3:96680001-96720000 | 3:97360001-97400000 | 2.51E-18 | HiC | GSE87112 | Mesenchymal_Stem_Cell | intra | rs62263705;rs62263706;rs6762348;rs62263709;rs137983052;rs62263734;rs2856474;rs2612274;rs553308194 | NA | 0 |
| 2 | 3:96680001-96720000 | 3:97400001-97440000 | 6.89E-13 | HiC | GSE87112 | Mesenchymal_Stem_Cell | intra | rs62263705;rs62263706;rs6762348;rs62263709;rs137983052;rs62263734;rs2856474;rs2612274;rs553308194 | NA | 0 |
| 2 | 3:96680001-96720000 | 3:97720001-97760000 | 5.93E-15 | HiC | GSE87112 | Mesenchymal_Stem_Cell | intra | rs62263705;rs62263706;rs6762348;rs62263709;rs137983052;rs62263734;rs2856474;rs2612274;rs553308194 | NA | 0 |
| 2 | 3:96680001-96720000 | 3:97920001-97960000 | 7.57E-08 | HiC | GSE87112 | Mesenchymal_Stem_Cell | intra | rs62263705;rs62263706;rs6762348;rs62263709;rs137983052;rs62263734;rs2856474;rs2612274;rs553308194 | NA | 0 |
| 2 | 3:96680001-96720000 | 3:98000001-98040000 | 4.05E-08 | HiC | GSE87112 | Mesenchymal_Stem_Cell | intra | rs62263705;rs62263706;rs6762348;rs62263709;rs137983052;rs62263734;rs2856474;rs2612274;rs553308194 | ENSG00000197938 | 0 |
| 3 | 4:171440001-171480000 | 4:171480001-171520000 | 6.92E-29 | HiC | GSE87112 | Mesenchymal_Stem_Cell | intra | rs147274196 | NA | 0 |
| 3 | 4:171440001-171480000 | 4:171520001-171560000 | 3.45E-39 | HiC | GSE87112 | Mesenchymal_Stem_Cell | intra | rs147274196 | NA | 0 |
| 3 | 4:171440001-171480000 | 4:171600001-171640000 | 5.41E-07 | HiC | GSE87112 | Mesenchymal_Stem_Cell | intra | rs147274196 | NA | 0 |
| 3 | 4:171440001-171480000 | 4:171640001-171680000 | 4.31E-20 | HiC | GSE87112 | Mesenchymal_Stem_Cell | intra | rs147274196 | NA | 0 |
| 3 | 4:171440001-171480000 | 4:171760001-171800000 | 4.56E-17 | HiC | GSE87112 | Mesenchymal_Stem_Cell | intra | rs147274196 | NA | 0 |
| 3 | 4:171440001-171480000 | 4:171840001-171880000 | 3.69E-07 | HiC | GSE87112 | Mesenchymal_Stem_Cell | intra | rs147274196 | NA | 0 |
| 3 | 4:171440001-171480000 | 4:171880001-171920000 | 1.98E-07 | HiC | GSE87112 | Mesenchymal_Stem_Cell | intra | rs147274196 | NA | 0 |
| 3 | 4:171440001-171480000 | 4:171960001-172000000 | 7.20E-15 | HiC | GSE87112 | Mesenchymal_Stem_Cell | intra | rs147274196 | NA | 0 |
| 3 | 4:171440001-171480000 | 4:172080001-172120000 | 2.32E-15 | HiC | GSE87112 | Mesenchymal_Stem_Cell | intra | rs147274196 | NA | 0 |
| 3 | 4:171440001-171480000 | 4:172120001-172160000 | 8.64E-07 | HiC | GSE87112 | Mesenchymal_Stem_Cell | intra | rs147274196 | NA | 0 |
| 3 | 4:171440001-171480000 | 4:172200001-172240000 | 1.16E-09 | HiC | GSE87112 | Mesenchymal_Stem_Cell | intra | rs147274196 | NA | 0 |
| 3 | 4:171440001-171480000 | 4:172280001-172320000 | 1.98E-08 | HiC | GSE87112 | Mesenchymal_Stem_Cell | intra | rs147274196 | NA | 0 |
| 3 | 4:171440001-171480000 | 4:172360001-172400000 | 6.58E-09 | HiC | GSE87112 | Mesenchymal_Stem_Cell | intra | rs147274196 | NA | 0 |
| 3 | 4:171440001-171480000 | 4:172440001-172480000 | 8.67E-11 | HiC | GSE87112 | Mesenchymal_Stem_Cell | intra | rs147274196 | NA | 0 |
| 3 | 4:171440001-171480000 | 4:172480001-172520000 | 5.72E-24 | HiC | GSE87112 | Mesenchymal_Stem_Cell | intra | rs147274196 | NA | 0 |
| 3 | 4:171440001-171480000 | 4:172520001-172560000 | 6.34E-17 | HiC | GSE87112 | Mesenchymal_Stem_Cell | intra | rs147274196 | NA | 0 |
| 3 | 4:171440001-171480000 | 4:172560001-172600000 | 4.29E-09 | HiC | GSE87112 | Mesenchymal_Stem_Cell | intra | rs147274196 | NA | 0 |
| 3 | 4:171440001-171480000 | 4:172600001-172640000 | 6.23E-16 | HiC | GSE87112 | Mesenchymal_Stem_Cell | intra | rs147274196 | NA | 0 |
| 3 | 4:171440001-171480000 | 4:172640001-172680000 | 8.15E-09 | HiC | GSE87112 | Mesenchymal_Stem_Cell | intra | rs147274196 | NA | 0 |
| 3 | 4:171440001-171480000 | 4:172680001-172720000 | 2.06E-12 | HiC | GSE87112 | Mesenchymal_Stem_Cell | intra | rs147274196 | NA | 0 |
| 3 | 4:171440001-171480000 | 4:172720001-172760000 | 3.33E-09 | HiC | GSE87112 | Mesenchymal_Stem_Cell | intra | rs147274196 | ENSG00000174473 | 0 |
| 3 | 4:171440001-171480000 | 4:172760001-172800000 | 3.69E-08 | HiC | GSE87112 | Mesenchymal_Stem_Cell | intra | rs147274196 | NA | 0 |
| 3 | 4:171440001-171480000 | 4:172840001-172880000 | 1.67E-10 | HiC | GSE87112 | Mesenchymal_Stem_Cell | intra | rs147274196 | NA | 0 |
| 3 | 4:171440001-171480000 | 4:172880001-172920000 | 5.65E-14 | HiC | GSE87112 | Mesenchymal_Stem_Cell | intra | rs147274196 | NA | 0 |
| 3 | 4:171440001-171480000 | 4:172920001-172960000 | 1.15E-07 | HiC | GSE87112 | Mesenchymal_Stem_Cell | intra | rs147274196 | NA | 0 |
| 3 | 4:171440001-171480000 | 4:173040001-173080000 | 7.54E-19 | HiC | GSE87112 | Mesenchymal_Stem_Cell | intra | rs147274196 | NA | 0 |
| 3 | 4:171440001-171480000 | 4:173080001-173120000 | 6.52E-19 | HiC | GSE87112 | Mesenchymal_Stem_Cell | intra | rs147274196 | NA | 0 |
| 3 | 4:171440001-171480000 | 4:173120001-173160000 | 3.00E-13 | HiC | GSE87112 | Mesenchymal_Stem_Cell | intra | rs147274196 | NA | 0 |
| 3 | 4:171440001-171480000 | 4:173160001-173200000 | 3.95E-10 | HiC | GSE87112 | Mesenchymal_Stem_Cell | intra | rs147274196 | NA | 0 |
| 3 | 4:171440001-171480000 | 4:173200001-173240000 | 2.82E-07 | HiC | GSE87112 | Mesenchymal_Stem_Cell | intra | rs147274196 | NA | 0 |
| 3 | 4:171440001-171480000 | 4:173240001-173280000 | 3.90E-07 | HiC | GSE87112 | Mesenchymal_Stem_Cell | intra | rs147274196 | NA | 0 |
| 3 | 4:171440001-171480000 | 4:173280001-173320000 | 2.64E-08 | HiC | GSE87112 | Mesenchymal_Stem_Cell | intra | rs147274196 | NA | 0 |
| 3 | 4:171440001-171480000 | 4:173400001-173440000 | 6.27E-14 | HiC | GSE87112 | Mesenchymal_Stem_Cell | intra | rs147274196 | NA | 0 |
| 3 | 4:171440001-171480000 | 4:173440001-173480000 | 5.04E-07 | HiC | GSE87112 | Mesenchymal_Stem_Cell | intra | rs147274196 | NA | 0 |
| 3 | 4:171440001-171480000 | 4:173560001-173600000 | 5.26E-14 | HiC | GSE87112 | Mesenchymal_Stem_Cell | intra | rs147274196 | NA | 0 |
| 3 | 4:171440001-171480000 | 4:173600001-173640000 | 5.04E-20 | HiC | GSE87112 | Mesenchymal_Stem_Cell | intra | rs147274196 | NA | 0 |
| 3 | 4:171440001-171480000 | 4:173680001-173720000 | 5.28E-20 | HiC | GSE87112 | Mesenchymal_Stem_Cell | intra | rs147274196 | NA | 0 |
| 3 | 4:171440001-171480000 | 4:173720001-173760000 | 1.07E-15 | HiC | GSE87112 | Mesenchymal_Stem_Cell | intra | rs147274196 | NA | 0 |
| 3 | 4:171440001-171480000 | 4:173760001-173800000 | 2.50E-21 | HiC | GSE87112 | Mesenchymal_Stem_Cell | intra | rs147274196 | NA | 0 |
| 3 | 4:171440001-171480000 | 4:173800001-173840000 | 1.40E-15 | HiC | GSE87112 | Mesenchymal_Stem_Cell | intra | rs147274196 | NA | 0 |
| 3 | 4:171440001-171480000 | 4:173880001-173920000 | 2.05E-17 | HiC | GSE87112 | Mesenchymal_Stem_Cell | intra | rs147274196 | NA | 0 |
| 3 | 4:171440001-171480000 | 4:173960001-174000000 | 7.61E-07 | HiC | GSE87112 | Mesenchymal_Stem_Cell | intra | rs147274196 | NA | 0 |
| 3 | 4:171440001-171480000 | 4:174160001-174200000 | 9.21E-08 | HiC | GSE87112 | Mesenchymal_Stem_Cell | intra | rs147274196 | NA | 0 |
| 3 | 4:171440001-171480000 | 4:174200001-174240000 | 4.40E-09 | HiC | GSE87112 | Mesenchymal_Stem_Cell | intra | rs147274196 | NA | 0 |
| 3 | 4:171480001-171520000 | 4:171520001-171560000 | 1.33E-75 | HiC | GSE87112 | Mesenchymal_Stem_Cell | intra | rs148420952;rs76413646 | NA | 0 |
| 3 | 4:171480001-171520000 | 4:171560001-171600000 | 5.06E-12 | HiC | GSE87112 | Mesenchymal_Stem_Cell | intra | rs148420952;rs76413646 | NA | 0 |
| 3 | 4:171480001-171520000 | 4:171600001-171640000 | 9.68E-10 | HiC | GSE87112 | Mesenchymal_Stem_Cell | intra | rs148420952;rs76413646 | NA | 0 |
| 3 | 4:171480001-171520000 | 4:171640001-171680000 | 1.51E-25 | HiC | GSE87112 | Mesenchymal_Stem_Cell | intra | rs148420952;rs76413646 | NA | 0 |
| 3 | 4:171480001-171520000 | 4:171760001-171800000 | 8.29E-33 | HiC | GSE87112 | Mesenchymal_Stem_Cell | intra | rs148420952;rs76413646 | NA | 0 |
| 3 | 4:171480001-171520000 | 4:171800001-171840000 | 7.38E-08 | HiC | GSE87112 | Mesenchymal_Stem_Cell | intra | rs148420952;rs76413646 | NA | 0 |
| 3 | 4:171480001-171520000 | 4:171880001-171920000 | 3.69E-07 | HiC | GSE87112 | Mesenchymal_Stem_Cell | intra | rs148420952;rs76413646 | NA | 0 |
| 3 | 4:171480001-171520000 | 4:171960001-172000000 | 3.63E-23 | HiC | GSE87112 | Mesenchymal_Stem_Cell | intra | rs148420952;rs76413646 | NA | 0 |
| 3 | 4:171480001-171520000 | 4:172080001-172120000 | 2.96E-10 | HiC | GSE87112 | Mesenchymal_Stem_Cell | intra | rs148420952;rs76413646 | NA | 0 |
| 3 | 4:171480001-171520000 | 4:172360001-172400000 | 1.55E-12 | HiC | GSE87112 | Mesenchymal_Stem_Cell | intra | rs148420952;rs76413646 | NA | 0 |
| 3 | 4:171480001-171520000 | 4:172440001-172480000 | 2.63E-15 | HiC | GSE87112 | Mesenchymal_Stem_Cell | intra | rs148420952;rs76413646 | NA | 0 |
| 3 | 4:171480001-171520000 | 4:172480001-172520000 | 1.19E-13 | HiC | GSE87112 | Mesenchymal_Stem_Cell | intra | rs148420952;rs76413646 | NA | 0 |
| 3 | 4:171480001-171520000 | 4:172520001-172560000 | 7.55E-22 | HiC | GSE87112 | Mesenchymal_Stem_Cell | intra | rs148420952;rs76413646 | NA | 0 |
| 3 | 4:171480001-171520000 | 4:172560001-172600000 | 4.33E-08 | HiC | GSE87112 | Mesenchymal_Stem_Cell | intra | rs148420952;rs76413646 | NA | 0 |
| 3 | 4:171480001-171520000 | 4:172600001-172640000 | 1.69E-15 | HiC | GSE87112 | Mesenchymal_Stem_Cell | intra | rs148420952;rs76413646 | NA | 0 |
| 3 | 4:171480001-171520000 | 4:172640001-172680000 | 1.37E-08 | HiC | GSE87112 | Mesenchymal_Stem_Cell | intra | rs148420952;rs76413646 | NA | 0 |
| 3 | 4:171480001-171520000 | 4:172680001-172720000 | 7.83E-11 | HiC | GSE87112 | Mesenchymal_Stem_Cell | intra | rs148420952;rs76413646 | NA | 0 |
| 3 | 4:171480001-171520000 | 4:172720001-172760000 | 2.03E-09 | HiC | GSE87112 | Mesenchymal_Stem_Cell | intra | rs148420952;rs76413646 | ENSG00000174473 | 0 |
| 3 | 4:171480001-171520000 | 4:172760001-172800000 | 4.58E-15 | HiC | GSE87112 | Mesenchymal_Stem_Cell | intra | rs148420952;rs76413646 | NA | 0 |
| 3 | 4:171480001-171520000 | 4:172840001-172880000 | 1.71E-07 | HiC | GSE87112 | Mesenchymal_Stem_Cell | intra | rs148420952;rs76413646 | NA | 0 |
| 3 | 4:171480001-171520000 | 4:172880001-172920000 | 6.54E-17 | HiC | GSE87112 | Mesenchymal_Stem_Cell | intra | rs148420952;rs76413646 | NA | 0 |
| 3 | 4:171480001-171520000 | 4:172920001-172960000 | 1.75E-14 | HiC | GSE87112 | Mesenchymal_Stem_Cell | intra | rs148420952;rs76413646 | NA | 0 |
| 3 | 4:171480001-171520000 | 4:172960001-173000000 | 7.01E-07 | HiC | GSE87112 | Mesenchymal_Stem_Cell | intra | rs148420952;rs76413646 | NA | 0 |
| 3 | 4:171480001-171520000 | 4:173040001-173080000 | 2.96E-11 | HiC | GSE87112 | Mesenchymal_Stem_Cell | intra | rs148420952;rs76413646 | NA | 0 |
| 3 | 4:171480001-171520000 | 4:173080001-173120000 | 1.75E-35 | HiC | GSE87112 | Mesenchymal_Stem_Cell | intra | rs148420952;rs76413646 | NA | 0 |
| 3 | 4:171480001-171520000 | 4:173120001-173160000 | 9.31E-13 | HiC | GSE87112 | Mesenchymal_Stem_Cell | intra | rs148420952;rs76413646 | NA | 0 |
| 3 | 4:171480001-171520000 | 4:173160001-173200000 | 1.95E-15 | HiC | GSE87112 | Mesenchymal_Stem_Cell | intra | rs148420952;rs76413646 | NA | 0 |
| 3 | 4:171480001-171520000 | 4:173200001-173240000 | 1.19E-09 | HiC | GSE87112 | Mesenchymal_Stem_Cell | intra | rs148420952;rs76413646 | NA | 0 |
| 3 | 4:171480001-171520000 | 4:173400001-173440000 | 7.31E-09 | HiC | GSE87112 | Mesenchymal_Stem_Cell | intra | rs148420952;rs76413646 | NA | 0 |
| 3 | 4:171480001-171520000 | 4:173520001-173560000 | 1.10E-07 | HiC | GSE87112 | Mesenchymal_Stem_Cell | intra | rs148420952;rs76413646 | NA | 0 |
| 3 | 4:171480001-171520000 | 4:173560001-173600000 | 2.62E-14 | HiC | GSE87112 | Mesenchymal_Stem_Cell | intra | rs148420952;rs76413646 | NA | 0 |
| 3 | 4:171480001-171520000 | 4:173600001-173640000 | 1.03E-17 | HiC | GSE87112 | Mesenchymal_Stem_Cell | intra | rs148420952;rs76413646 | NA | 0 |
| 3 | 4:171480001-171520000 | 4:173680001-173720000 | 9.02E-13 | HiC | GSE87112 | Mesenchymal_Stem_Cell | intra | rs148420952;rs76413646 | NA | 0 |
| 3 | 4:171480001-171520000 | 4:173720001-173760000 | 5.38E-18 | HiC | GSE87112 | Mesenchymal_Stem_Cell | intra | rs148420952;rs76413646 | NA | 0 |
| 3 | 4:171480001-171520000 | 4:173760001-173800000 | 1.43E-27 | HiC | GSE87112 | Mesenchymal_Stem_Cell | intra | rs148420952;rs76413646 | NA | 0 |
| 3 | 4:171480001-171520000 | 4:173800001-173840000 | 1.34E-16 | HiC | GSE87112 | Mesenchymal_Stem_Cell | intra | rs148420952;rs76413646 | NA | 0 |
| 3 | 4:171480001-171520000 | 4:173880001-173920000 | 1.10E-22 | HiC | GSE87112 | Mesenchymal_Stem_Cell | intra | rs148420952;rs76413646 | NA | 0 |
| 3 | 4:171480001-171520000 | 4:174200001-174240000 | 3.00E-07 | HiC | GSE87112 | Mesenchymal_Stem_Cell | intra | rs148420952;rs76413646 | NA | 0 |
| 4 | 5:108560001-108600000 | 5:108600001-108640000 | 1.60E-13 | HiC | GSE87112 | Mesenchymal_Stem_Cell | intra | rs35812497;rs75803484;rs75073283;rs76430100;rs75782477;rs77992445;rs61701247;rs17161655;rs115979287;rs17161659;rs151072743;rs17161665;rs6863688;5:108591279:C:T;5:108591279:A:T;rs1363213;rs1862201;rs1862202;rs1862203;rs5870359 | NA | 0 |
| 4 | 5:108560001-108600000 | 5:108640001-108680000 | 1.29E-11 | HiC | GSE87112 | Mesenchymal_Stem_Cell | intra | rs35812497;rs75803484;rs75073283;rs76430100;rs75782477;rs77992445;rs61701247;rs17161655;rs115979287;rs17161659;rs151072743;rs17161665;rs6863688;5:108591279:C:T;5:108591279:A:T;rs1363213;rs1862201;rs1862202;rs1862203;rs5870359 | NA | 0 |
| 4 | 5:108560001-108600000 | 5:108680001-108720000 | 6.34E-16 | HiC | GSE87112 | Mesenchymal_Stem_Cell | intra | rs35812497;rs75803484;rs75073283;rs76430100;rs75782477;rs77992445;rs61701247;rs17161655;rs115979287;rs17161659;rs151072743;rs17161665;rs6863688;5:108591279:C:T;5:108591279:A:T;rs1363213;rs1862201;rs1862202;rs1862203;rs5870359 | NA | 0 |
| 4 | 5:108560001-108600000 | 5:108800001-108840000 | 4.33E-41 | HiC | GSE87112 | Mesenchymal_Stem_Cell | intra | rs35812497;rs75803484;rs75073283;rs76430100;rs75782477;rs77992445;rs61701247;rs17161655;rs115979287;rs17161659;rs151072743;rs17161665;rs6863688;5:108591279:C:T;5:108591279:A:T;rs1363213;rs1862201;rs1862202;rs1862203;rs5870359 | NA | 0 |
| 4 | 5:108560001-108600000 | 5:108920001-108960000 | 1.77E-14 | HiC | GSE87112 | Mesenchymal_Stem_Cell | intra | rs35812497;rs75803484;rs75073283;rs76430100;rs75782477;rs77992445;rs61701247;rs17161655;rs115979287;rs17161659;rs151072743;rs17161665;rs6863688;5:108591279:C:T;5:108591279:A:T;rs1363213;rs1862201;rs1862202;rs1862203;rs5870359 | NA | 0 |
| 4 | 5:108560001-108600000 | 5:109240001-109280000 | 2.39E-09 | HiC | GSE87112 | Mesenchymal_Stem_Cell | intra | rs35812497;rs75803484;rs75073283;rs76430100;rs75782477;rs77992445;rs61701247;rs17161655;rs115979287;rs17161659;rs151072743;rs17161665;rs6863688;5:108591279:C:T;5:108591279:A:T;rs1363213;rs1862201;rs1862202;rs1862203;rs5870359 | NA | 0 |
| 4 | 5:108560001-108600000 | 5:109320001-109360000 | 1.88E-07 | HiC | GSE87112 | Mesenchymal_Stem_Cell | intra | rs35812497;rs75803484;rs75073283;rs76430100;rs75782477;rs77992445;rs61701247;rs17161655;rs115979287;rs17161659;rs151072743;rs17161665;rs6863688;5:108591279:C:T;5:108591279:A:T;rs1363213;rs1862201;rs1862202;rs1862203;rs5870359 | NA | 0 |
| 4 | 5:108560001-108600000 | 5:109520001-109560000 | 2.42E-08 | HiC | GSE87112 | Mesenchymal_Stem_Cell | intra | rs35812497;rs75803484;rs75073283;rs76430100;rs75782477;rs77992445;rs61701247;rs17161655;rs115979287;rs17161659;rs151072743;rs17161665;rs6863688;5:108591279:C:T;5:108591279:A:T;rs1363213;rs1862201;rs1862202;rs1862203;rs5870359 | NA | 0 |
| 4 | 5:108560001-108600000 | 5:109560001-109600000 | 6.13E-08 | HiC | GSE87112 | Mesenchymal_Stem_Cell | intra | rs35812497;rs75803484;rs75073283;rs76430100;rs75782477;rs77992445;rs61701247;rs17161655;rs115979287;rs17161659;rs151072743;rs17161665;rs6863688;5:108591279:C:T;5:108591279:A:T;rs1363213;rs1862201;rs1862202;rs1862203;rs5870359 | NA | 0 |
| 4 | 5:108560001-108600000 | 5:109640001-109680000 | 3.06E-09 | HiC | GSE87112 | Mesenchymal_Stem_Cell | intra | rs35812497;rs75803484;rs75073283;rs76430100;rs75782477;rs77992445;rs61701247;rs17161655;rs115979287;rs17161659;rs151072743;rs17161665;rs6863688;5:108591279:C:T;5:108591279:A:T;rs1363213;rs1862201;rs1862202;rs1862203;rs5870359 | NA | 0 |
| 4 | 5:108560001-108600000 | 5:109680001-109720000 | 9.03E-15 | HiC | GSE87112 | Mesenchymal_Stem_Cell | intra | rs35812497;rs75803484;rs75073283;rs76430100;rs75782477;rs77992445;rs61701247;rs17161655;rs115979287;rs17161659;rs151072743;rs17161665;rs6863688;5:108591279:C:T;5:108591279:A:T;rs1363213;rs1862201;rs1862202;rs1862203;rs5870359 | NA | 0 |
| 4 | 5:108560001-108600000 | 5:109720001-109760000 | 3.38E-14 | HiC | GSE87112 | Mesenchymal_Stem_Cell | intra | rs35812497;rs75803484;rs75073283;rs76430100;rs75782477;rs77992445;rs61701247;rs17161655;rs115979287;rs17161659;rs151072743;rs17161665;rs6863688;5:108591279:C:T;5:108591279:A:T;rs1363213;rs1862201;rs1862202;rs1862203;rs5870359 | NA | 0 |
| 4 | 5:108560001-108600000 | 5:109840001-109880000 | 1.19E-14 | HiC | GSE87112 | Mesenchymal_Stem_Cell | intra | rs35812497;rs75803484;rs75073283;rs76430100;rs75782477;rs77992445;rs61701247;rs17161655;rs115979287;rs17161659;rs151072743;rs17161665;rs6863688;5:108591279:C:T;5:108591279:A:T;rs1363213;rs1862201;rs1862202;rs1862203;rs5870359 | NA | 0 |
| 4 | 5:108560001-108600000 | 5:109880001-109920000 | 5.01E-11 | HiC | GSE87112 | Mesenchymal_Stem_Cell | intra | rs35812497;rs75803484;rs75073283;rs76430100;rs75782477;rs77992445;rs61701247;rs17161655;rs115979287;rs17161659;rs151072743;rs17161665;rs6863688;5:108591279:C:T;5:108591279:A:T;rs1363213;rs1862201;rs1862202;rs1862203;rs5870359 | NA | 0 |
| 4 | 5:108560001-108600000 | 5:109920001-109960000 | 2.14E-18 | HiC | GSE87112 | Mesenchymal_Stem_Cell | intra | rs35812497;rs75803484;rs75073283;rs76430100;rs75782477;rs77992445;rs61701247;rs17161655;rs115979287;rs17161659;rs151072743;rs17161665;rs6863688;5:108591279:C:T;5:108591279:A:T;rs1363213;rs1862201;rs1862202;rs1862203;rs5870359 | NA | 0 |
| 4 | 5:108560001-108600000 | 5:109960001-110000000 | 2.60E-11 | HiC | GSE87112 | Mesenchymal_Stem_Cell | intra | rs35812497;rs75803484;rs75073283;rs76430100;rs75782477;rs77992445;rs61701247;rs17161655;rs115979287;rs17161659;rs151072743;rs17161665;rs6863688;5:108591279:C:T;5:108591279:A:T;rs1363213;rs1862201;rs1862202;rs1862203;rs5870359 | NA | 0 |
| 4 | 5:108560001-108600000 | 5:110040001-110080000 | 1.05E-08 | HiC | GSE87112 | Mesenchymal_Stem_Cell | intra | rs35812497;rs75803484;rs75073283;rs76430100;rs75782477;rs77992445;rs61701247;rs17161655;rs115979287;rs17161659;rs151072743;rs17161665;rs6863688;5:108591279:C:T;5:108591279:A:T;rs1363213;rs1862201;rs1862202;rs1862203;rs5870359 | ENSG00000186952:ENSG00000164209 | 0 |
| 4 | 5:108600001-108640000 | 5:108800001-108840000 | 1.56E-33 | HiC | GSE87112 | Mesenchymal_Stem_Cell | intra | rs11744353;rs79776200;rs76069623;rs76043709;rs78729571;rs36123121;rs11748450;rs80234158;rs78146542;rs185072862;rs137867205;rs189006725;rs4388251;rs4438924;rs4541698;rs4388252;rs4392675;rs200252534;rs145520315;rs147699420;rs77803982;rs79469094;rs75077625;rs74379173;rs78664536;rs549536839;rs570631944;rs78784808;rs78340649;rs201569786;rs75649455;rs78814720;rs77561644;rs76565008;rs74436342;rs1592807;rs1592810;rs1592811;rs7700458;rs6594365;rs6863893;rs140163909;rs146837743;rs7705919;rs201046346;rs202152005;rs199869120;rs111726541;rs373673987;rs113026830;rs148211606;rs201619508;rs201169921;rs146644914;rs116649742;rs79893293;rs57629433;rs60335509;rs58453996;rs59466242;rs60878839;rs11743452;rs1833567;rs1833568;rs1833569;rs7721272;rs7721427;rs7721730;rs7721870;rs7722100;rs7704359;rs1895200;rs1895201;rs77974856;rs60905342;rs11746207;rs11739672;rs11749718;rs78518991;rs76243602;rs76383235;rs11741980;rs35243960;rs116462975;rs139801813;rs113103584;rs74727230;rs75919325;rs79624714;rs2080858;rs138092354;rs148285468;rs114455395;rs74850689;rs10463599 | NA | 0 |
| 4 | 5:108600001-108640000 | 5:109920001-109960000 | 3.96E-10 | HiC | GSE87112 | Mesenchymal_Stem_Cell | intra | rs11744353;rs79776200;rs76069623;rs76043709;rs78729571;rs36123121;rs11748450;rs80234158;rs78146542;rs185072862;rs137867205;rs189006725;rs4388251;rs4438924;rs4541698;rs4388252;rs4392675;rs200252534;rs145520315;rs147699420;rs77803982;rs79469094;rs75077625;rs74379173;rs78664536;rs549536839;rs570631944;rs78784808;rs78340649;rs201569786;rs75649455;rs78814720;rs77561644;rs76565008;rs74436342;rs1592807;rs1592810;rs1592811;rs7700458;rs6594365;rs6863893;rs140163909;rs146837743;rs7705919;rs201046346;rs202152005;rs199869120;rs111726541;rs373673987;rs113026830;rs148211606;rs201619508;rs201169921;rs146644914;rs116649742;rs79893293;rs57629433;rs60335509;rs58453996;rs59466242;rs60878839;rs11743452;rs1833567;rs1833568;rs1833569;rs7721272;rs7721427;rs7721730;rs7721870;rs7722100;rs7704359;rs1895200;rs1895201;rs77974856;rs60905342;rs11746207;rs11739672;rs11749718;rs78518991;rs76243602;rs76383235;rs11741980;rs35243960;rs116462975;rs139801813;rs113103584;rs74727230;rs75919325;rs79624714;rs2080858;rs138092354;rs148285468;rs114455395;rs74850689;rs10463599 | NA | 0 |
| 4 | 5:108600001-108640000 | 5:109960001-110000000 | 8.07E-10 | HiC | GSE87112 | Mesenchymal_Stem_Cell | intra | rs11744353;rs79776200;rs76069623;rs76043709;rs78729571;rs36123121;rs11748450;rs80234158;rs78146542;rs185072862;rs137867205;rs189006725;rs4388251;rs4438924;rs4541698;rs4388252;rs4392675;rs200252534;rs145520315;rs147699420;rs77803982;rs79469094;rs75077625;rs74379173;rs78664536;rs549536839;rs570631944;rs78784808;rs78340649;rs201569786;rs75649455;rs78814720;rs77561644;rs76565008;rs74436342;rs1592807;rs1592810;rs1592811;rs7700458;rs6594365;rs6863893;rs140163909;rs146837743;rs7705919;rs201046346;rs202152005;rs199869120;rs111726541;rs373673987;rs113026830;rs148211606;rs201619508;rs201169921;rs146644914;rs116649742;rs79893293;rs57629433;rs60335509;rs58453996;rs59466242;rs60878839;rs11743452;rs1833567;rs1833568;rs1833569;rs7721272;rs7721427;rs7721730;rs7721870;rs7722100;rs7704359;rs1895200;rs1895201;rs77974856;rs60905342;rs11746207;rs11739672;rs11749718;rs78518991;rs76243602;rs76383235;rs11741980;rs35243960;rs116462975;rs139801813;rs113103584;rs74727230;rs75919325;rs79624714;rs2080858;rs138092354;rs148285468;rs114455395;rs74850689;rs10463599 | NA | 0 |
| 4 | 5:108600001-108640000 | 5:110040001-110080000 | 3.05E-08 | HiC | GSE87112 | Mesenchymal_Stem_Cell | intra | rs11744353;rs79776200;rs76069623;rs76043709;rs78729571;rs36123121;rs11748450;rs80234158;rs78146542;rs185072862;rs137867205;rs189006725;rs4388251;rs4438924;rs4541698;rs4388252;rs4392675;rs200252534;rs145520315;rs147699420;rs77803982;rs79469094;rs75077625;rs74379173;rs78664536;rs549536839;rs570631944;rs78784808;rs78340649;rs201569786;rs75649455;rs78814720;rs77561644;rs76565008;rs74436342;rs1592807;rs1592810;rs1592811;rs7700458;rs6594365;rs6863893;rs140163909;rs146837743;rs7705919;rs201046346;rs202152005;rs199869120;rs111726541;rs373673987;rs113026830;rs148211606;rs201619508;rs201169921;rs146644914;rs116649742;rs79893293;rs57629433;rs60335509;rs58453996;rs59466242;rs60878839;rs11743452;rs1833567;rs1833568;rs1833569;rs7721272;rs7721427;rs7721730;rs7721870;rs7722100;rs7704359;rs1895200;rs1895201;rs77974856;rs60905342;rs11746207;rs11739672;rs11749718;rs78518991;rs76243602;rs76383235;rs11741980;rs35243960;rs116462975;rs139801813;rs113103584;rs74727230;rs75919325;rs79624714;rs2080858;rs138092354;rs148285468;rs114455395;rs74850689;rs10463599 | ENSG00000186952:ENSG00000164209 | 0 |
| 5 | 11:2520001-2560000 | 11:2880001-2920000 | 1.09E-08 | HiC | GSE87112 | Mesenchymal_Stem_Cell | intra | rs111815403 | ENSG00000129757 | 0 |
| 5 | 11:2520001-2560000 | 11:2960001-3000000 | 3.00E-16 | HiC | GSE87112 | Mesenchymal_Stem_Cell | intra | rs111815403 | NA | 0 |
| 5 | 11:2520001-2560000 | 11:3360001-3400000 | 3.86E-10 | HiC | GSE87112 | Mesenchymal_Stem_Cell | intra | rs111815403 | ENSG00000005801 | 1 |
| 6 | 12:80800001-80840000 | 12:80880001-80920000 | 8.70E-29 | HiC | GSE87112 | Mesenchymal_Stem_Cell | intra | rs117203215;rs77741796 | NA | 0 |
| 6 | 12:80800001-80840000 | 12:80920001-80960000 | 1.35E-80 | HiC | GSE87112 | Mesenchymal_Stem_Cell | intra | rs117203215;rs77741796 | NA | 0 |
| 6 | 12:80800001-80840000 | 12:81000001-81040000 | 8.05E-57 | HiC | GSE87112 | Mesenchymal_Stem_Cell | intra | rs117203215;rs77741796 | NA | 0 |
| 6 | 12:80800001-80840000 | 12:81040001-81080000 | 1.26E-50 | HiC | GSE87112 | Mesenchymal_Stem_Cell | intra | rs117203215;rs77741796 | NA | 0 |
| 6 | 12:80800001-80840000 | 12:81080001-81120000 | 4.88E-68 | HiC | GSE87112 | Mesenchymal_Stem_Cell | intra | rs117203215;rs77741796 | ENSG00000111046:ENSG00000111049 | 0 |
| 6 | 12:80800001-80840000 | 12:81120001-81160000 | 4.66E-50 | HiC | GSE87112 | Mesenchymal_Stem_Cell | intra | rs117203215;rs77741796 | NA | 0 |
| 6 | 12:80800001-80840000 | 12:81160001-81200000 | 1.08E-37 | HiC | GSE87112 | Mesenchymal_Stem_Cell | intra | rs117203215;rs77741796 | NA | 0 |
| 6 | 12:80800001-80840000 | 12:81200001-81240000 | 1.22E-15 | HiC | GSE87112 | Mesenchymal_Stem_Cell | intra | rs117203215;rs77741796 | NA | 0 |
| 6 | 12:80800001-80840000 | 12:81240001-81280000 | 1.05E-58 | HiC | GSE87112 | Mesenchymal_Stem_Cell | intra | rs117203215;rs77741796 | NA | 0 |
| 6 | 12:80800001-80840000 | 12:81280001-81320000 | 1.34E-73 | HiC | GSE87112 | Mesenchymal_Stem_Cell | intra | rs117203215;rs77741796 | NA | 0 |
| 6 | 12:80800001-80840000 | 12:81320001-81360000 | 1.13E-29 | HiC | GSE87112 | Mesenchymal_Stem_Cell | intra | rs117203215;rs77741796 | ENSG00000111052:ENSG00000111058 | 0 |
| 6 | 12:80800001-80840000 | 12:81360001-81400000 | 1.89E-12 | HiC | GSE87112 | Mesenchymal_Stem_Cell | intra | rs117203215;rs77741796 | NA | 0 |
| 6 | 12:80800001-80840000 | 12:81400001-81440000 | 3.56E-28 | HiC | GSE87112 | Mesenchymal_Stem_Cell | intra | rs117203215;rs77741796 | NA | 0 |
| 6 | 12:80800001-80840000 | 12:81440001-81480000 | 6.68E-14 | HiC | GSE87112 | Mesenchymal_Stem_Cell | intra | rs117203215;rs77741796 | NA | 0 |
| 6 | 12:80800001-80840000 | 12:81480001-81520000 | 2.76E-67 | HiC | GSE87112 | Mesenchymal_Stem_Cell | intra | rs117203215;rs77741796 | NA | 0 |
| 6 | 12:80800001-80840000 | 12:81520001-81560000 | 3.55E-26 | HiC | GSE87112 | Mesenchymal_Stem_Cell | intra | rs117203215;rs77741796 | NA | 0 |
| 6 | 12:80800001-80840000 | 12:81560001-81600000 | 1.24E-12 | HiC | GSE87112 | Mesenchymal_Stem_Cell | intra | rs117203215;rs77741796 | NA | 0 |
| 6 | 12:80800001-80840000 | 12:81600001-81640000 | 6.02E-74 | HiC | GSE87112 | Mesenchymal_Stem_Cell | intra | rs117203215;rs77741796 | NA | 0 |
| 6 | 12:80800001-80840000 | 12:81640001-81680000 | 3.60E-100 | HiC | GSE87112 | Mesenchymal_Stem_Cell | intra | rs117203215;rs77741796 | NA | 0 |
| 6 | 12:80800001-80840000 | 12:81680001-81720000 | 1.37E-31 | HiC | GSE87112 | Mesenchymal_Stem_Cell | intra | rs117203215;rs77741796 | NA | 0 |
| 6 | 12:80800001-80840000 | 12:81760001-81800000 | 3.12E-17 | HiC | GSE87112 | Mesenchymal_Stem_Cell | intra | rs117203215;rs77741796 | NA | 0 |
| 6 | 12:80800001-80840000 | 12:81840001-81880000 | 4.73E-09 | HiC | GSE87112 | Mesenchymal_Stem_Cell | intra | rs117203215;rs77741796 | NA | 0 |
| 6 | 12:80800001-80840000 | 12:81920001-81960000 | 2.98E-14 | HiC | GSE87112 | Mesenchymal_Stem_Cell | intra | rs117203215;rs77741796 | NA | 0 |
| 6 | 12:80800001-80840000 | 12:81960001-82000000 | 4.02E-13 | HiC | GSE87112 | Mesenchymal_Stem_Cell | intra | rs117203215;rs77741796 | NA | 0 |
| 6 | 12:80800001-80840000 | 12:82000001-82040000 | 3.31E-09 | HiC | GSE87112 | Mesenchymal_Stem_Cell | intra | rs117203215;rs77741796 | NA | 0 |
| 6 | 12:80800001-80840000 | 12:82080001-82120000 | 4.08E-16 | HiC | GSE87112 | Mesenchymal_Stem_Cell | intra | rs117203215;rs77741796 | NA | 0 |
| 6 | 12:80800001-80840000 | 12:82680001-82720000 | 1.35E-08 | HiC | GSE87112 | Mesenchymal_Stem_Cell | intra | rs117203215;rs77741796 | NA | 0 |
| 6 | 12:80840001-80880000 | 12:80920001-80960000 | 8.62E-14 | HiC | GSE87112 | Mesenchymal_Stem_Cell | intra | rs200589835 | NA | 0 |
| 6 | 12:80840001-80880000 | 12:81000001-81040000 | 5.06E-15 | HiC | GSE87112 | Mesenchymal_Stem_Cell | intra | rs200589835 | NA | 0 |
| 6 | 12:80840001-80880000 | 12:81080001-81120000 | 7.98E-09 | HiC | GSE87112 | Mesenchymal_Stem_Cell | intra | rs200589835 | ENSG00000111046:ENSG00000111049 | 0 |
| 6 | 12:80840001-80880000 | 12:81120001-81160000 | 1.01E-09 | HiC | GSE87112 | Mesenchymal_Stem_Cell | intra | rs200589835 | NA | 0 |
| 6 | 12:80840001-80880000 | 12:81160001-81200000 | 5.41E-08 | HiC | GSE87112 | Mesenchymal_Stem_Cell | intra | rs200589835 | NA | 0 |
| 6 | 12:80840001-80880000 | 12:81240001-81280000 | 2.71E-13 | HiC | GSE87112 | Mesenchymal_Stem_Cell | intra | rs200589835 | NA | 0 |
| 6 | 12:80840001-80880000 | 12:81280001-81320000 | 3.61E-12 | HiC | GSE87112 | Mesenchymal_Stem_Cell | intra | rs200589835 | NA | 0 |
| 6 | 12:80840001-80880000 | 12:81480001-81520000 | 6.68E-14 | HiC | GSE87112 | Mesenchymal_Stem_Cell | intra | rs200589835 | NA | 0 |
| 6 | 12:80840001-80880000 | 12:81600001-81640000 | 1.72E-16 | HiC | GSE87112 | Mesenchymal_Stem_Cell | intra | rs200589835 | NA | 0 |
| 6 | 12:80840001-80880000 | 12:81640001-81680000 | 4.91E-15 | HiC | GSE87112 | Mesenchymal_Stem_Cell | intra | rs200589835 | NA | 0 |
| 7 | 12:127760001-127800000 | 12:127800001-127840000 | 3.09E-114 | HiC | GSE87112 | Mesenchymal_Stem_Cell | intra | rs1810088;rs367839627;rs146043604;rs143439170;rs10773382;rs10847307;rs35154640;rs1552386;rs7314186;rs1979065;rs5801722;rs10744302;12:127775513:A:C;rs10734938;rs35024927;rs10773387;rs978813;rs2348321;rs10847321;rs10847323;rs6489163 | NA | 0 |
| 7 | 12:127760001-127800000 | 12:127840001-127880000 | 8.77E-25 | HiC | GSE87112 | Mesenchymal_Stem_Cell | intra | rs1810088;rs367839627;rs146043604;rs143439170;rs10773382;rs10847307;rs35154640;rs1552386;rs7314186;rs1979065;rs5801722;rs10744302;12:127775513:A:C;rs10734938;rs35024927;rs10773387;rs978813;rs2348321;rs10847321;rs10847323;rs6489163 | NA | 0 |
| 7 | 12:127760001-127800000 | 12:127880001-127920000 | 8.38E-15 | HiC | GSE87112 | Mesenchymal_Stem_Cell | intra | rs1810088;rs367839627;rs146043604;rs143439170;rs10773382;rs10847307;rs35154640;rs1552386;rs7314186;rs1979065;rs5801722;rs10744302;12:127775513:A:C;rs10734938;rs35024927;rs10773387;rs978813;rs2348321;rs10847321;rs10847323;rs6489163 | NA | 0 |
| 7 | 12:127760001-127800000 | 12:127960001-128000000 | 5.12E-17 | HiC | GSE87112 | Mesenchymal_Stem_Cell | intra | rs1810088;rs367839627;rs146043604;rs143439170;rs10773382;rs10847307;rs35154640;rs1552386;rs7314186;rs1979065;rs5801722;rs10744302;12:127775513:A:C;rs10734938;rs35024927;rs10773387;rs978813;rs2348321;rs10847321;rs10847323;rs6489163 | NA | 0 |
| 7 | 12:127760001-127800000 | 12:128000001-128040000 | 1.03E-23 | HiC | GSE87112 | Mesenchymal_Stem_Cell | intra | rs1810088;rs367839627;rs146043604;rs143439170;rs10773382;rs10847307;rs35154640;rs1552386;rs7314186;rs1979065;rs5801722;rs10744302;12:127775513:A:C;rs10734938;rs35024927;rs10773387;rs978813;rs2348321;rs10847321;rs10847323;rs6489163 | NA | 0 |
| 7 | 12:127760001-127800000 | 12:128040001-128080000 | 6.95E-22 | HiC | GSE87112 | Mesenchymal_Stem_Cell | intra | rs1810088;rs367839627;rs146043604;rs143439170;rs10773382;rs10847307;rs35154640;rs1552386;rs7314186;rs1979065;rs5801722;rs10744302;12:127775513:A:C;rs10734938;rs35024927;rs10773387;rs978813;rs2348321;rs10847321;rs10847323;rs6489163 | NA | 0 |
| 7 | 12:127760001-127800000 | 12:128120001-128160000 | 8.83E-13 | HiC | GSE87112 | Mesenchymal_Stem_Cell | intra | rs1810088;rs367839627;rs146043604;rs143439170;rs10773382;rs10847307;rs35154640;rs1552386;rs7314186;rs1979065;rs5801722;rs10744302;12:127775513:A:C;rs10734938;rs35024927;rs10773387;rs978813;rs2348321;rs10847321;rs10847323;rs6489163 | NA | 0 |
| 7 | 12:127760001-127800000 | 12:128200001-128240000 | 7.52E-09 | HiC | GSE87112 | Mesenchymal_Stem_Cell | intra | rs1810088;rs367839627;rs146043604;rs143439170;rs10773382;rs10847307;rs35154640;rs1552386;rs7314186;rs1979065;rs5801722;rs10744302;12:127775513:A:C;rs10734938;rs35024927;rs10773387;rs978813;rs2348321;rs10847321;rs10847323;rs6489163 | NA | 0 |
| 7 | 12:127760001-127800000 | 12:128240001-128280000 | 4.46E-08 | HiC | GSE87112 | Mesenchymal_Stem_Cell | intra | rs1810088;rs367839627;rs146043604;rs143439170;rs10773382;rs10847307;rs35154640;rs1552386;rs7314186;rs1979065;rs5801722;rs10744302;12:127775513:A:C;rs10734938;rs35024927;rs10773387;rs978813;rs2348321;rs10847321;rs10847323;rs6489163 | NA | 0 |
| 7 | 12:127760001-127800000 | 12:128360001-128400000 | 1.92E-13 | HiC | GSE87112 | Mesenchymal_Stem_Cell | intra | rs1810088;rs367839627;rs146043604;rs143439170;rs10773382;rs10847307;rs35154640;rs1552386;rs7314186;rs1979065;rs5801722;rs10744302;12:127775513:A:C;rs10734938;rs35024927;rs10773387;rs978813;rs2348321;rs10847321;rs10847323;rs6489163 | NA | 0 |
| 7 | 12:127760001-127800000 | 12:128400001-128440000 | 1.85E-37 | HiC | GSE87112 | Mesenchymal_Stem_Cell | intra | rs1810088;rs367839627;rs146043604;rs143439170;rs10773382;rs10847307;rs35154640;rs1552386;rs7314186;rs1979065;rs5801722;rs10744302;12:127775513:A:C;rs10734938;rs35024927;rs10773387;rs978813;rs2348321;rs10847321;rs10847323;rs6489163 | NA | 0 |
| 7 | 12:127760001-127800000 | 12:128480001-128520000 | 1.26E-20 | HiC | GSE87112 | Mesenchymal_Stem_Cell | intra | rs1810088;rs367839627;rs146043604;rs143439170;rs10773382;rs10847307;rs35154640;rs1552386;rs7314186;rs1979065;rs5801722;rs10744302;12:127775513:A:C;rs10734938;rs35024927;rs10773387;rs978813;rs2348321;rs10847321;rs10847323;rs6489163 | NA | 0 |
| 7 | 12:127760001-127800000 | 12:128520001-128560000 | 1.05E-29 | HiC | GSE87112 | Mesenchymal_Stem_Cell | intra | rs1810088;rs367839627;rs146043604;rs143439170;rs10773382;rs10847307;rs35154640;rs1552386;rs7314186;rs1979065;rs5801722;rs10744302;12:127775513:A:C;rs10734938;rs35024927;rs10773387;rs978813;rs2348321;rs10847321;rs10847323;rs6489163 | NA | 0 |
| 7 | 12:127760001-127800000 | 12:128600001-128640000 | 6.15E-14 | HiC | GSE87112 | Mesenchymal_Stem_Cell | intra | rs1810088;rs367839627;rs146043604;rs143439170;rs10773382;rs10847307;rs35154640;rs1552386;rs7314186;rs1979065;rs5801722;rs10744302;12:127775513:A:C;rs10734938;rs35024927;rs10773387;rs978813;rs2348321;rs10847321;rs10847323;rs6489163 | NA | 0 |
| 7 | 12:127760001-127800000 | 12:128640001-128680000 | 6.40E-10 | HiC | GSE87112 | Mesenchymal_Stem_Cell | intra | rs1810088;rs367839627;rs146043604;rs143439170;rs10773382;rs10847307;rs35154640;rs1552386;rs7314186;rs1979065;rs5801722;rs10744302;12:127775513:A:C;rs10734938;rs35024927;rs10773387;rs978813;rs2348321;rs10847321;rs10847323;rs6489163 | NA | 0 |
| 7 | 12:127760001-127800000 | 12:128720001-128760000 | 5.74E-10 | HiC | GSE87112 | Mesenchymal_Stem_Cell | intra | rs1810088;rs367839627;rs146043604;rs143439170;rs10773382;rs10847307;rs35154640;rs1552386;rs7314186;rs1979065;rs5801722;rs10744302;12:127775513:A:C;rs10734938;rs35024927;rs10773387;rs978813;rs2348321;rs10847321;rs10847323;rs6489163 | ENSG00000181234 | 0 |
| 7 | 12:127760001-127800000 | 12:128840001-128880000 | 1.81E-18 | HiC | GSE87112 | Mesenchymal_Stem_Cell | intra | rs1810088;rs367839627;rs146043604;rs143439170;rs10773382;rs10847307;rs35154640;rs1552386;rs7314186;rs1979065;rs5801722;rs10744302;12:127775513:A:C;rs10734938;rs35024927;rs10773387;rs978813;rs2348321;rs10847321;rs10847323;rs6489163 | NA | 0 |
| 7 | 12:127760001-127800000 | 12:129080001-129120000 | 4.86E-07 | HiC | GSE87112 | Mesenchymal_Stem_Cell | intra | rs1810088;rs367839627;rs146043604;rs143439170;rs10773382;rs10847307;rs35154640;rs1552386;rs7314186;rs1979065;rs5801722;rs10744302;12:127775513:A:C;rs10734938;rs35024927;rs10773387;rs978813;rs2348321;rs10847321;rs10847323;rs6489163 | NA | 0 |
| 7 | 12:127760001-127800000 | 12:129400001-129440000 | 1.64E-07 | HiC | GSE87112 | Mesenchymal_Stem_Cell | intra | rs1810088;rs367839627;rs146043604;rs143439170;rs10773382;rs10847307;rs35154640;rs1552386;rs7314186;rs1979065;rs5801722;rs10744302;12:127775513:A:C;rs10734938;rs35024927;rs10773387;rs978813;rs2348321;rs10847321;rs10847323;rs6489163 | NA | 0 |
| 7 | 12:127760001-127800000 | 12:129480001-129520000 | 4.23E-08 | HiC | GSE87112 | Mesenchymal_Stem_Cell | intra | rs1810088;rs367839627;rs146043604;rs143439170;rs10773382;rs10847307;rs35154640;rs1552386;rs7314186;rs1979065;rs5801722;rs10744302;12:127775513:A:C;rs10734938;rs35024927;rs10773387;rs978813;rs2348321;rs10847321;rs10847323;rs6489163 | NA | 0 |
| 7 | 12:127760001-127800000 | 12:129520001-129560000 | 2.12E-09 | HiC | GSE87112 | Mesenchymal_Stem_Cell | intra | rs1810088;rs367839627;rs146043604;rs143439170;rs10773382;rs10847307;rs35154640;rs1552386;rs7314186;rs1979065;rs5801722;rs10744302;12:127775513:A:C;rs10734938;rs35024927;rs10773387;rs978813;rs2348321;rs10847321;rs10847323;rs6489163 | NA | 0 |
| 7 | 12:127760001-127800000 | 12:129600001-129640000 | 7.21E-08 | HiC | GSE87112 | Mesenchymal_Stem_Cell | intra | rs1810088;rs367839627;rs146043604;rs143439170;rs10773382;rs10847307;rs35154640;rs1552386;rs7314186;rs1979065;rs5801722;rs10744302;12:127775513:A:C;rs10734938;rs35024927;rs10773387;rs978813;rs2348321;rs10847321;rs10847323;rs6489163 | NA | 0 |
| 7 | 12:127760001-127800000 | 12:129640001-129680000 | 3.33E-10 | HiC | GSE87112 | Mesenchymal_Stem_Cell | intra | rs1810088;rs367839627;rs146043604;rs143439170;rs10773382;rs10847307;rs35154640;rs1552386;rs7314186;rs1979065;rs5801722;rs10744302;12:127775513:A:C;rs10734938;rs35024927;rs10773387;rs978813;rs2348321;rs10847321;rs10847323;rs6489163 | NA | 0 |
| 7 | 12:127760001-127800000 | 12:129760001-129800000 | 9.52E-10 | HiC | GSE87112 | Mesenchymal_Stem_Cell | intra | rs1810088;rs367839627;rs146043604;rs143439170;rs10773382;rs10847307;rs35154640;rs1552386;rs7314186;rs1979065;rs5801722;rs10744302;12:127775513:A:C;rs10734938;rs35024927;rs10773387;rs978813;rs2348321;rs10847321;rs10847323;rs6489163 | NA | 0 |
| 7 | 12:127760001-127800000 | 12:129960001-130000000 | 3.22E-11 | HiC | GSE87112 | Mesenchymal_Stem_Cell | intra | rs1810088;rs367839627;rs146043604;rs143439170;rs10773382;rs10847307;rs35154640;rs1552386;rs7314186;rs1979065;rs5801722;rs10744302;12:127775513:A:C;rs10734938;rs35024927;rs10773387;rs978813;rs2348321;rs10847321;rs10847323;rs6489163 | NA | 0 |
| 7 | 12:127760001-127800000 | 12:130000001-130040000 | 2.93E-13 | HiC | GSE87112 | Mesenchymal_Stem_Cell | intra | rs1810088;rs367839627;rs146043604;rs143439170;rs10773382;rs10847307;rs35154640;rs1552386;rs7314186;rs1979065;rs5801722;rs10744302;12:127775513:A:C;rs10734938;rs35024927;rs10773387;rs978813;rs2348321;rs10847321;rs10847323;rs6489163 | NA | 0 |
| 7 | 12:127760001-127800000 | 12:130120001-130160000 | 7.94E-07 | HiC | GSE87112 | Mesenchymal_Stem_Cell | intra | rs1810088;rs367839627;rs146043604;rs143439170;rs10773382;rs10847307;rs35154640;rs1552386;rs7314186;rs1979065;rs5801722;rs10744302;12:127775513:A:C;rs10734938;rs35024927;rs10773387;rs978813;rs2348321;rs10847321;rs10847323;rs6489163 | NA | 0 |
| 7 | 12:127760001-127800000 | 12:130160001-130200000 | 2.00E-13 | HiC | GSE87112 | Mesenchymal_Stem_Cell | intra | rs1810088;rs367839627;rs146043604;rs143439170;rs10773382;rs10847307;rs35154640;rs1552386;rs7314186;rs1979065;rs5801722;rs10744302;12:127775513:A:C;rs10734938;rs35024927;rs10773387;rs978813;rs2348321;rs10847321;rs10847323;rs6489163 | NA | 0 |
| 7 | 12:127760001-127800000 | 12:130240001-130280000 | 1.10E-16 | HiC | GSE87112 | Mesenchymal_Stem_Cell | intra | rs1810088;rs367839627;rs146043604;rs143439170;rs10773382;rs10847307;rs35154640;rs1552386;rs7314186;rs1979065;rs5801722;rs10744302;12:127775513:A:C;rs10734938;rs35024927;rs10773387;rs978813;rs2348321;rs10847321;rs10847323;rs6489163 | NA | 0 |
| 7 | 12:127760001-127800000 | 12:130400001-130440000 | 1.44E-07 | HiC | GSE87112 | Mesenchymal_Stem_Cell | intra | rs1810088;rs367839627;rs146043604;rs143439170;rs10773382;rs10847307;rs35154640;rs1552386;rs7314186;rs1979065;rs5801722;rs10744302;12:127775513:A:C;rs10734938;rs35024927;rs10773387;rs978813;rs2348321;rs10847321;rs10847323;rs6489163 | NA | 0 |
| 7 | 12:127760001-127800000 | 12:130440001-130480000 | 2.05E-16 | HiC | GSE87112 | Mesenchymal_Stem_Cell | intra | rs1810088;rs367839627;rs146043604;rs143439170;rs10773382;rs10847307;rs35154640;rs1552386;rs7314186;rs1979065;rs5801722;rs10744302;12:127775513:A:C;rs10734938;rs35024927;rs10773387;rs978813;rs2348321;rs10847321;rs10847323;rs6489163 | NA | 0 |
| 7 | 12:127760001-127800000 | 12:130480001-130520000 | 5.08E-07 | HiC | GSE87112 | Mesenchymal_Stem_Cell | intra | rs1810088;rs367839627;rs146043604;rs143439170;rs10773382;rs10847307;rs35154640;rs1552386;rs7314186;rs1979065;rs5801722;rs10744302;12:127775513:A:C;rs10734938;rs35024927;rs10773387;rs978813;rs2348321;rs10847321;rs10847323;rs6489163 | NA | 0 |
| 7 | 12:127760001-127800000 | 12:130640001-130680000 | 1.01E-07 | HiC | GSE87112 | Mesenchymal_Stem_Cell | intra | rs1810088;rs367839627;rs146043604;rs143439170;rs10773382;rs10847307;rs35154640;rs1552386;rs7314186;rs1979065;rs5801722;rs10744302;12:127775513:A:C;rs10734938;rs35024927;rs10773387;rs978813;rs2348321;rs10847321;rs10847323;rs6489163 | ENSG00000111432 | 0 |
| 7 | 12:127760001-127800000 | 12:130680001-130720000 | 7.88E-20 | HiC | GSE87112 | Mesenchymal_Stem_Cell | intra | rs1810088;rs367839627;rs146043604;rs143439170;rs10773382;rs10847307;rs35154640;rs1552386;rs7314186;rs1979065;rs5801722;rs10744302;12:127775513:A:C;rs10734938;rs35024927;rs10773387;rs978813;rs2348321;rs10847321;rs10847323;rs6489163 | NA | 0 |
| 7 | 12:127760001-127800000 | 12:130720001-130760000 | 2.54E-08 | HiC | GSE87112 | Mesenchymal_Stem_Cell | intra | rs1810088;rs367839627;rs146043604;rs143439170;rs10773382;rs10847307;rs35154640;rs1552386;rs7314186;rs1979065;rs5801722;rs10744302;12:127775513:A:C;rs10734938;rs35024927;rs10773387;rs978813;rs2348321;rs10847321;rs10847323;rs6489163 | NA | 0 |
| 2 | 3:96680001-96720000 | 3:93680001-93720000 | 2.45E-08 | HiC | GSE87112 | Mesenchymal_Stem_Cell | intra | rs62263705;rs62263706;rs6762348;rs62263709;rs137983052;rs62263734;rs2856474;rs2612274;rs553308194 | ENSG00000184500:ENSG00000169379 | 0 |
| 2 | 3:96520001-96560000 | 3:93720001-93760000 | 3.43E-07 | HiC | GSE87112 | Mesenchymal_Stem_Cell | intra | rs62262901;rs62262902;rs62262903;rs62262930;rs151165295;rs62262931;rs62262933;rs62262934;rs74641574;rs79645223;rs2318156;rs2318155;rs2318154 | ENSG00000178750 | 0 |
| 2 | 3:96680001-96720000 | 3:93720001-93760000 | 9.15E-08 | HiC | GSE87112 | Mesenchymal_Stem_Cell | intra | rs62263705;rs62263706;rs6762348;rs62263709;rs137983052;rs62263734;rs2856474;rs2612274;rs553308194 | ENSG00000178750 | 0 |
| 2 | 3:96520001-96560000 | 3:93800001-93840000 | 1.82E-34 | HiC | GSE87112 | Mesenchymal_Stem_Cell | intra | rs62262901;rs62262902;rs62262903;rs62262930;rs151165295;rs62262931;rs62262933;rs62262934;rs74641574;rs79645223;rs2318156;rs2318155;rs2318154 | NA | 0 |
| 2 | 3:96560001-96600000 | 3:93800001-93840000 | 3.00E-12 | HiC | GSE87112 | Mesenchymal_Stem_Cell | intra | rs16836353;rs62262935;rs62262936;rs62262937;rs62262938;rs62262940;rs62262941;rs62262942;rs62262944;rs62262946;rs62262947;rs62262948;rs62262949;rs62262950;rs62262951 | NA | 0 |
| 2 | 3:96600001-96640000 | 3:93800001-93840000 | 1.69E-17 | HiC | GSE87112 | Mesenchymal_Stem_Cell | intra | rs62262952;rs62262953;rs116253310;rs76857958;rs111706803;rs62262955;rs62262957;rs62262958;rs62263676;rs187619159;rs62263677;rs62263678;rs574559070;rs534937763;rs62263680;rs62263682;rs62263684;rs16836982;rs16836986;rs112527011;rs62263685;rs62263686 | NA | 0 |
| 2 | 3:96680001-96720000 | 3:93800001-93840000 | 3.72E-24 | HiC | GSE87112 | Mesenchymal_Stem_Cell | intra | rs62263705;rs62263706;rs6762348;rs62263709;rs137983052;rs62263734;rs2856474;rs2612274;rs553308194 | NA | 0 |
| 2 | 3:96520001-96560000 | 3:93840001-93880000 | 1.31E-15 | HiC | GSE87112 | Mesenchymal_Stem_Cell | intra | rs62262901;rs62262902;rs62262903;rs62262930;rs151165295;rs62262931;rs62262933;rs62262934;rs74641574;rs79645223;rs2318156;rs2318155;rs2318154 | NA | 0 |
| 2 | 3:96560001-96600000 | 3:93840001-93880000 | 1.43E-14 | HiC | GSE87112 | Mesenchymal_Stem_Cell | intra | rs16836353;rs62262935;rs62262936;rs62262937;rs62262938;rs62262940;rs62262941;rs62262942;rs62262944;rs62262946;rs62262947;rs62262948;rs62262949;rs62262950;rs62262951 | NA | 0 |
| 2 | 3:96600001-96640000 | 3:93840001-93880000 | 1.31E-11 | HiC | GSE87112 | Mesenchymal_Stem_Cell | intra | rs62262952;rs62262953;rs116253310;rs76857958;rs111706803;rs62262955;rs62262957;rs62262958;rs62263676;rs187619159;rs62263677;rs62263678;rs574559070;rs534937763;rs62263680;rs62263682;rs62263684;rs16836982;rs16836986;rs112527011;rs62263685;rs62263686 | NA | 0 |
| 2 | 3:96680001-96720000 | 3:93840001-93880000 | 2.72E-20 | HiC | GSE87112 | Mesenchymal_Stem_Cell | intra | rs62263705;rs62263706;rs6762348;rs62263709;rs137983052;rs62263734;rs2856474;rs2612274;rs553308194 | NA | 0 |
| 2 | 3:96520001-96560000 | 3:93880001-93920000 | 4.72E-57 | HiC | GSE87112 | Mesenchymal_Stem_Cell | intra | rs62262901;rs62262902;rs62262903;rs62262930;rs151165295;rs62262931;rs62262933;rs62262934;rs74641574;rs79645223;rs2318156;rs2318155;rs2318154 | NA | 0 |
| 2 | 3:96560001-96600000 | 3:93880001-93920000 | 1.03E-28 | HiC | GSE87112 | Mesenchymal_Stem_Cell | intra | rs16836353;rs62262935;rs62262936;rs62262937;rs62262938;rs62262940;rs62262941;rs62262942;rs62262944;rs62262946;rs62262947;rs62262948;rs62262949;rs62262950;rs62262951 | NA | 0 |
| 2 | 3:96600001-96640000 | 3:93880001-93920000 | 1.23E-45 | HiC | GSE87112 | Mesenchymal_Stem_Cell | intra | rs62262952;rs62262953;rs116253310;rs76857958;rs111706803;rs62262955;rs62262957;rs62262958;rs62263676;rs187619159;rs62263677;rs62263678;rs574559070;rs534937763;rs62263680;rs62263682;rs62263684;rs16836982;rs16836986;rs112527011;rs62263685;rs62263686 | NA | 0 |
| 2 | 3:96640001-96680000 | 3:93880001-93920000 | 5.60E-11 | HiC | GSE87112 | Mesenchymal_Stem_Cell | intra | rs62263687;rs62263693;rs2318066;rs62263694;rs74563548;rs62263696;rs185127539;rs145486125;rs62263697;rs148690862;rs62263699;rs149222683;rs111574505;rs62263701;rs62263702;rs62263703;rs75261331;rs62263704;rs189373463 | NA | 0 |
| 2 | 3:96680001-96720000 | 3:93880001-93920000 | 2.41E-44 | HiC | GSE87112 | Mesenchymal_Stem_Cell | intra | rs62263705;rs62263706;rs6762348;rs62263709;rs137983052;rs62263734;rs2856474;rs2612274;rs553308194 | NA | 0 |
| 2 | 3:96720001-96760000 | 3:93880001-93920000 | 3.52E-09 | HiC | GSE87112 | Mesenchymal_Stem_Cell | intra | rs2027816;rs2856463;rs2019818;rs115680726;rs112449918;rs565327317;rs2213251;rs2213252;rs2856466 | NA | 0 |
| 2 | 3:96520001-96560000 | 3:93920001-93960000 | 3.10E-22 | HiC | GSE87112 | Mesenchymal_Stem_Cell | intra | rs62262901;rs62262902;rs62262903;rs62262930;rs151165295;rs62262931;rs62262933;rs62262934;rs74641574;rs79645223;rs2318156;rs2318155;rs2318154 | NA | 0 |
| 2 | 3:96560001-96600000 | 3:93920001-93960000 | 7.11E-07 | HiC | GSE87112 | Mesenchymal_Stem_Cell | intra | rs16836353;rs62262935;rs62262936;rs62262937;rs62262938;rs62262940;rs62262941;rs62262942;rs62262944;rs62262946;rs62262947;rs62262948;rs62262949;rs62262950;rs62262951 | NA | 0 |
| 2 | 3:96600001-96640000 | 3:93920001-93960000 | 2.02E-10 | HiC | GSE87112 | Mesenchymal_Stem_Cell | intra | rs62262952;rs62262953;rs116253310;rs76857958;rs111706803;rs62262955;rs62262957;rs62262958;rs62263676;rs187619159;rs62263677;rs62263678;rs574559070;rs534937763;rs62263680;rs62263682;rs62263684;rs16836982;rs16836986;rs112527011;rs62263685;rs62263686 | NA | 0 |
| 2 | 3:96680001-96720000 | 3:93920001-93960000 | 3.03E-14 | HiC | GSE87112 | Mesenchymal_Stem_Cell | intra | rs62263705;rs62263706;rs6762348;rs62263709;rs137983052;rs62263734;rs2856474;rs2612274;rs553308194 | NA | 0 |
| 2 | 3:96720001-96760000 | 3:93920001-93960000 | 3.43E-07 | HiC | GSE87112 | Mesenchymal_Stem_Cell | intra | rs2027816;rs2856463;rs2019818;rs115680726;rs112449918;rs565327317;rs2213251;rs2213252;rs2856466 | NA | 0 |
| 2 | 3:96520001-96560000 | 3:93960001-94000000 | 1.33E-95 | HiC | GSE87112 | Mesenchymal_Stem_Cell | intra | rs62262901;rs62262902;rs62262903;rs62262930;rs151165295;rs62262931;rs62262933;rs62262934;rs74641574;rs79645223;rs2318156;rs2318155;rs2318154 | NA | 0 |
| 2 | 3:96560001-96600000 | 3:93960001-94000000 | 1.42E-24 | HiC | GSE87112 | Mesenchymal_Stem_Cell | intra | rs16836353;rs62262935;rs62262936;rs62262937;rs62262938;rs62262940;rs62262941;rs62262942;rs62262944;rs62262946;rs62262947;rs62262948;rs62262949;rs62262950;rs62262951 | NA | 0 |
| 2 | 3:96600001-96640000 | 3:93960001-94000000 | 4.76E-51 | HiC | GSE87112 | Mesenchymal_Stem_Cell | intra | rs62262952;rs62262953;rs116253310;rs76857958;rs111706803;rs62262955;rs62262957;rs62262958;rs62263676;rs187619159;rs62263677;rs62263678;rs574559070;rs534937763;rs62263680;rs62263682;rs62263684;rs16836982;rs16836986;rs112527011;rs62263685;rs62263686 | NA | 0 |
| 2 | 3:96680001-96720000 | 3:93960001-94000000 | 1.13E-44 | HiC | GSE87112 | Mesenchymal_Stem_Cell | intra | rs62263705;rs62263706;rs6762348;rs62263709;rs137983052;rs62263734;rs2856474;rs2612274;rs553308194 | NA | 0 |
| 2 | 3:96720001-96760000 | 3:93960001-94000000 | 3.03E-14 | HiC | GSE87112 | Mesenchymal_Stem_Cell | intra | rs2027816;rs2856463;rs2019818;rs115680726;rs112449918;rs565327317;rs2213251;rs2213252;rs2856466 | NA | 0 |
| 2 | 3:96520001-96560000 | 3:94000001-94040000 | 6.89E-190 | HiC | GSE87112 | Mesenchymal_Stem_Cell | intra | rs62262901;rs62262902;rs62262903;rs62262930;rs151165295;rs62262931;rs62262933;rs62262934;rs74641574;rs79645223;rs2318156;rs2318155;rs2318154 | NA | 0 |
| 2 | 3:96560001-96600000 | 3:94000001-94040000 | 4.37E-106 | HiC | GSE87112 | Mesenchymal_Stem_Cell | intra | rs16836353;rs62262935;rs62262936;rs62262937;rs62262938;rs62262940;rs62262941;rs62262942;rs62262944;rs62262946;rs62262947;rs62262948;rs62262949;rs62262950;rs62262951 | NA | 0 |
| 2 | 3:96600001-96640000 | 3:94000001-94040000 | 4.79E-124 | HiC | GSE87112 | Mesenchymal_Stem_Cell | intra | rs62262952;rs62262953;rs116253310;rs76857958;rs111706803;rs62262955;rs62262957;rs62262958;rs62263676;rs187619159;rs62263677;rs62263678;rs574559070;rs534937763;rs62263680;rs62263682;rs62263684;rs16836982;rs16836986;rs112527011;rs62263685;rs62263686 | NA | 0 |
| 2 | 3:96640001-96680000 | 3:94000001-94040000 | 1.43E-19 | HiC | GSE87112 | Mesenchymal_Stem_Cell | intra | rs62263687;rs62263693;rs2318066;rs62263694;rs74563548;rs62263696;rs185127539;rs145486125;rs62263697;rs148690862;rs62263699;rs149222683;rs111574505;rs62263701;rs62263702;rs62263703;rs75261331;rs62263704;rs189373463 | NA | 0 |
| 2 | 3:96680001-96720000 | 3:94000001-94040000 | 2.81E-114 | HiC | GSE87112 | Mesenchymal_Stem_Cell | intra | rs62263705;rs62263706;rs6762348;rs62263709;rs137983052;rs62263734;rs2856474;rs2612274;rs553308194 | NA | 0 |
| 2 | 3:96720001-96760000 | 3:94000001-94040000 | 3.10E-29 | HiC | GSE87112 | Mesenchymal_Stem_Cell | intra | rs2027816;rs2856463;rs2019818;rs115680726;rs112449918;rs565327317;rs2213251;rs2213252;rs2856466 | NA | 0 |
| 2 | 3:96520001-96560000 | 3:94040001-94080000 | 4.01E-87 | HiC | GSE87112 | Mesenchymal_Stem_Cell | intra | rs62262901;rs62262902;rs62262903;rs62262930;rs151165295;rs62262931;rs62262933;rs62262934;rs74641574;rs79645223;rs2318156;rs2318155;rs2318154 | NA | 0 |
| 2 | 3:96560001-96600000 | 3:94040001-94080000 | 1.62E-16 | HiC | GSE87112 | Mesenchymal_Stem_Cell | intra | rs16836353;rs62262935;rs62262936;rs62262937;rs62262938;rs62262940;rs62262941;rs62262942;rs62262944;rs62262946;rs62262947;rs62262948;rs62262949;rs62262950;rs62262951 | NA | 0 |
| 2 | 3:96600001-96640000 | 3:94040001-94080000 | 1.19E-22 | HiC | GSE87112 | Mesenchymal_Stem_Cell | intra | rs62262952;rs62262953;rs116253310;rs76857958;rs111706803;rs62262955;rs62262957;rs62262958;rs62263676;rs187619159;rs62263677;rs62263678;rs574559070;rs534937763;rs62263680;rs62263682;rs62263684;rs16836982;rs16836986;rs112527011;rs62263685;rs62263686 | NA | 0 |
| 2 | 3:96640001-96680000 | 3:94040001-94080000 | 2.18E-09 | HiC | GSE87112 | Mesenchymal_Stem_Cell | intra | rs62263687;rs62263693;rs2318066;rs62263694;rs74563548;rs62263696;rs185127539;rs145486125;rs62263697;rs148690862;rs62263699;rs149222683;rs111574505;rs62263701;rs62263702;rs62263703;rs75261331;rs62263704;rs189373463 | NA | 0 |
| 2 | 3:96680001-96720000 | 3:94040001-94080000 | 3.08E-28 | HiC | GSE87112 | Mesenchymal_Stem_Cell | intra | rs62263705;rs62263706;rs6762348;rs62263709;rs137983052;rs62263734;rs2856474;rs2612274;rs553308194 | NA | 0 |
| 2 | 3:96520001-96560000 | 3:94080001-94120000 | 4.35E-18 | HiC | GSE87112 | Mesenchymal_Stem_Cell | intra | rs62262901;rs62262902;rs62262903;rs62262930;rs151165295;rs62262931;rs62262933;rs62262934;rs74641574;rs79645223;rs2318156;rs2318155;rs2318154 | NA | 0 |
| 2 | 3:96520001-96560000 | 3:94120001-94160000 | 2.44E-57 | HiC | GSE87112 | Mesenchymal_Stem_Cell | intra | rs62262901;rs62262902;rs62262903;rs62262930;rs151165295;rs62262931;rs62262933;rs62262934;rs74641574;rs79645223;rs2318156;rs2318155;rs2318154 | NA | 0 |
| 2 | 3:96560001-96600000 | 3:94120001-94160000 | 3.09E-20 | HiC | GSE87112 | Mesenchymal_Stem_Cell | intra | rs16836353;rs62262935;rs62262936;rs62262937;rs62262938;rs62262940;rs62262941;rs62262942;rs62262944;rs62262946;rs62262947;rs62262948;rs62262949;rs62262950;rs62262951 | NA | 0 |
| 2 | 3:96600001-96640000 | 3:94120001-94160000 | 2.35E-12 | HiC | GSE87112 | Mesenchymal_Stem_Cell | intra | rs62262952;rs62262953;rs116253310;rs76857958;rs111706803;rs62262955;rs62262957;rs62262958;rs62263676;rs187619159;rs62263677;rs62263678;rs574559070;rs534937763;rs62263680;rs62263682;rs62263684;rs16836982;rs16836986;rs112527011;rs62263685;rs62263686 | NA | 0 |
| 2 | 3:96640001-96680000 | 3:94120001-94160000 | 5.14E-09 | HiC | GSE87112 | Mesenchymal_Stem_Cell | intra | rs62263687;rs62263693;rs2318066;rs62263694;rs74563548;rs62263696;rs185127539;rs145486125;rs62263697;rs148690862;rs62263699;rs149222683;rs111574505;rs62263701;rs62263702;rs62263703;rs75261331;rs62263704;rs189373463 | NA | 0 |
| 2 | 3:96680001-96720000 | 3:94120001-94160000 | 5.45E-25 | HiC | GSE87112 | Mesenchymal_Stem_Cell | intra | rs62263705;rs62263706;rs6762348;rs62263709;rs137983052;rs62263734;rs2856474;rs2612274;rs553308194 | NA | 0 |
| 2 | 3:96520001-96560000 | 3:94160001-94200000 | 2.15E-36 | HiC | GSE87112 | Mesenchymal_Stem_Cell | intra | rs62262901;rs62262902;rs62262903;rs62262930;rs151165295;rs62262931;rs62262933;rs62262934;rs74641574;rs79645223;rs2318156;rs2318155;rs2318154 | NA | 0 |
| 2 | 3:96560001-96600000 | 3:94160001-94200000 | 5.83E-12 | HiC | GSE87112 | Mesenchymal_Stem_Cell | intra | rs16836353;rs62262935;rs62262936;rs62262937;rs62262938;rs62262940;rs62262941;rs62262942;rs62262944;rs62262946;rs62262947;rs62262948;rs62262949;rs62262950;rs62262951 | NA | 0 |
| 2 | 3:96600001-96640000 | 3:94160001-94200000 | 3.09E-20 | HiC | GSE87112 | Mesenchymal_Stem_Cell | intra | rs62262952;rs62262953;rs116253310;rs76857958;rs111706803;rs62262955;rs62262957;rs62262958;rs62263676;rs187619159;rs62263677;rs62263678;rs574559070;rs534937763;rs62263680;rs62263682;rs62263684;rs16836982;rs16836986;rs112527011;rs62263685;rs62263686 | NA | 0 |
| 2 | 3:96680001-96720000 | 3:94160001-94200000 | 1.45E-21 | HiC | GSE87112 | Mesenchymal_Stem_Cell | intra | rs62263705;rs62263706;rs6762348;rs62263709;rs137983052;rs62263734;rs2856474;rs2612274;rs553308194 | NA | 0 |
| 2 | 3:96520001-96560000 | 3:94200001-94240000 | 3.72E-42 | HiC | GSE87112 | Mesenchymal_Stem_Cell | intra | rs62262901;rs62262902;rs62262903;rs62262930;rs151165295;rs62262931;rs62262933;rs62262934;rs74641574;rs79645223;rs2318156;rs2318155;rs2318154 | NA | 0 |
| 2 | 3:96560001-96600000 | 3:94200001-94240000 | 7.33E-08 | HiC | GSE87112 | Mesenchymal_Stem_Cell | intra | rs16836353;rs62262935;rs62262936;rs62262937;rs62262938;rs62262940;rs62262941;rs62262942;rs62262944;rs62262946;rs62262947;rs62262948;rs62262949;rs62262950;rs62262951 | NA | 0 |
| 2 | 3:96600001-96640000 | 3:94200001-94240000 | 1.80E-14 | HiC | GSE87112 | Mesenchymal_Stem_Cell | intra | rs62262952;rs62262953;rs116253310;rs76857958;rs111706803;rs62262955;rs62262957;rs62262958;rs62263676;rs187619159;rs62263677;rs62263678;rs574559070;rs534937763;rs62263680;rs62263682;rs62263684;rs16836982;rs16836986;rs112527011;rs62263685;rs62263686 | NA | 0 |
| 2 | 3:96680001-96720000 | 3:94200001-94240000 | 5.98E-17 | HiC | GSE87112 | Mesenchymal_Stem_Cell | intra | rs62263705;rs62263706;rs6762348;rs62263709;rs137983052;rs62263734;rs2856474;rs2612274;rs553308194 | NA | 0 |
| 2 | 3:96520001-96560000 | 3:94240001-94280000 | 5.73E-50 | HiC | GSE87112 | Mesenchymal_Stem_Cell | intra | rs62262901;rs62262902;rs62262903;rs62262930;rs151165295;rs62262931;rs62262933;rs62262934;rs74641574;rs79645223;rs2318156;rs2318155;rs2318154 | NA | 0 |
| 2 | 3:96560001-96600000 | 3:94240001-94280000 | 3.50E-12 | HiC | GSE87112 | Mesenchymal_Stem_Cell | intra | rs16836353;rs62262935;rs62262936;rs62262937;rs62262938;rs62262940;rs62262941;rs62262942;rs62262944;rs62262946;rs62262947;rs62262948;rs62262949;rs62262950;rs62262951 | NA | 0 |
| 2 | 3:96600001-96640000 | 3:94240001-94280000 | 1.08E-19 | HiC | GSE87112 | Mesenchymal_Stem_Cell | intra | rs62262952;rs62262953;rs116253310;rs76857958;rs111706803;rs62262955;rs62262957;rs62262958;rs62263676;rs187619159;rs62263677;rs62263678;rs574559070;rs534937763;rs62263680;rs62263682;rs62263684;rs16836982;rs16836986;rs112527011;rs62263685;rs62263686 | NA | 0 |
| 2 | 3:96680001-96720000 | 3:94240001-94280000 | 3.09E-20 | HiC | GSE87112 | Mesenchymal_Stem_Cell | intra | rs62263705;rs62263706;rs6762348;rs62263709;rs137983052;rs62263734;rs2856474;rs2612274;rs553308194 | NA | 0 |
| 2 | 3:96520001-96560000 | 3:94280001-94320000 | 7.04E-15 | HiC | GSE87112 | Mesenchymal_Stem_Cell | intra | rs62262901;rs62262902;rs62262903;rs62262930;rs151165295;rs62262931;rs62262933;rs62262934;rs74641574;rs79645223;rs2318156;rs2318155;rs2318154 | NA | 0 |
| 2 | 3:96600001-96640000 | 3:94280001-94320000 | 5.38E-11 | HiC | GSE87112 | Mesenchymal_Stem_Cell | intra | rs62262952;rs62262953;rs116253310;rs76857958;rs111706803;rs62262955;rs62262957;rs62262958;rs62263676;rs187619159;rs62263677;rs62263678;rs574559070;rs534937763;rs62263680;rs62263682;rs62263684;rs16836982;rs16836986;rs112527011;rs62263685;rs62263686 | NA | 0 |
| 2 | 3:96680001-96720000 | 3:94280001-94320000 | 3.84E-17 | HiC | GSE87112 | Mesenchymal_Stem_Cell | intra | rs62263705;rs62263706;rs6762348;rs62263709;rs137983052;rs62263734;rs2856474;rs2612274;rs553308194 | NA | 0 |
| 2 | 3:96520001-96560000 | 3:94320001-94360000 | 3.54E-22 | HiC | GSE87112 | Mesenchymal_Stem_Cell | intra | rs62262901;rs62262902;rs62262903;rs62262930;rs151165295;rs62262931;rs62262933;rs62262934;rs74641574;rs79645223;rs2318156;rs2318155;rs2318154 | NA | 0 |
| 2 | 3:96560001-96600000 | 3:94320001-94360000 | 1.59E-15 | HiC | GSE87112 | Mesenchymal_Stem_Cell | intra | rs16836353;rs62262935;rs62262936;rs62262937;rs62262938;rs62262940;rs62262941;rs62262942;rs62262944;rs62262946;rs62262947;rs62262948;rs62262949;rs62262950;rs62262951 | NA | 0 |
| 2 | 3:96600001-96640000 | 3:94320001-94360000 | 3.32E-13 | HiC | GSE87112 | Mesenchymal_Stem_Cell | intra | rs62262952;rs62262953;rs116253310;rs76857958;rs111706803;rs62262955;rs62262957;rs62262958;rs62263676;rs187619159;rs62263677;rs62263678;rs574559070;rs534937763;rs62263680;rs62263682;rs62263684;rs16836982;rs16836986;rs112527011;rs62263685;rs62263686 | NA | 0 |
| 2 | 3:96680001-96720000 | 3:94320001-94360000 | 6.69E-17 | HiC | GSE87112 | Mesenchymal_Stem_Cell | intra | rs62263705;rs62263706;rs6762348;rs62263709;rs137983052;rs62263734;rs2856474;rs2612274;rs553308194 | NA | 0 |
| 2 | 3:96520001-96560000 | 3:94360001-94400000 | 8.17E-25 | HiC | GSE87112 | Mesenchymal_Stem_Cell | intra | rs62262901;rs62262902;rs62262903;rs62262930;rs151165295;rs62262931;rs62262933;rs62262934;rs74641574;rs79645223;rs2318156;rs2318155;rs2318154 | NA | 0 |
| 2 | 3:96560001-96600000 | 3:94360001-94400000 | 2.41E-09 | HiC | GSE87112 | Mesenchymal_Stem_Cell | intra | rs16836353;rs62262935;rs62262936;rs62262937;rs62262938;rs62262940;rs62262941;rs62262942;rs62262944;rs62262946;rs62262947;rs62262948;rs62262949;rs62262950;rs62262951 | NA | 0 |
| 2 | 3:96600001-96640000 | 3:94360001-94400000 | 7.04E-15 | HiC | GSE87112 | Mesenchymal_Stem_Cell | intra | rs62262952;rs62262953;rs116253310;rs76857958;rs111706803;rs62262955;rs62262957;rs62262958;rs62263676;rs187619159;rs62263677;rs62263678;rs574559070;rs534937763;rs62263680;rs62263682;rs62263684;rs16836982;rs16836986;rs112527011;rs62263685;rs62263686 | NA | 0 |
| 2 | 3:96680001-96720000 | 3:94360001-94400000 | 5.38E-11 | HiC | GSE87112 | Mesenchymal_Stem_Cell | intra | rs62263705;rs62263706;rs6762348;rs62263709;rs137983052;rs62263734;rs2856474;rs2612274;rs553308194 | NA | 0 |
| 2 | 3:96520001-96560000 | 3:94400001-94440000 | 1.57E-21 | HiC | GSE87112 | Mesenchymal_Stem_Cell | intra | rs62262901;rs62262902;rs62262903;rs62262930;rs151165295;rs62262931;rs62262933;rs62262934;rs74641574;rs79645223;rs2318156;rs2318155;rs2318154 | NA | 0 |
| 2 | 3:96600001-96640000 | 3:94400001-94440000 | 8.21E-09 | HiC | GSE87112 | Mesenchymal_Stem_Cell | intra | rs62262952;rs62262953;rs116253310;rs76857958;rs111706803;rs62262955;rs62262957;rs62262958;rs62263676;rs187619159;rs62263677;rs62263678;rs574559070;rs534937763;rs62263680;rs62263682;rs62263684;rs16836982;rs16836986;rs112527011;rs62263685;rs62263686 | NA | 0 |
| 2 | 3:96680001-96720000 | 3:94400001-94440000 | 1.36E-12 | HiC | GSE87112 | Mesenchymal_Stem_Cell | intra | rs62263705;rs62263706;rs6762348;rs62263709;rs137983052;rs62263734;rs2856474;rs2612274;rs553308194 | NA | 0 |
| 2 | 3:96520001-96560000 | 3:94440001-94480000 | 8.82E-16 | HiC | GSE87112 | Mesenchymal_Stem_Cell | intra | rs62262901;rs62262902;rs62262903;rs62262930;rs151165295;rs62262931;rs62262933;rs62262934;rs74641574;rs79645223;rs2318156;rs2318155;rs2318154 | NA | 0 |
| 2 | 3:96600001-96640000 | 3:94440001-94480000 | 1.05E-09 | HiC | GSE87112 | Mesenchymal_Stem_Cell | intra | rs62262952;rs62262953;rs116253310;rs76857958;rs111706803;rs62262955;rs62262957;rs62262958;rs62263676;rs187619159;rs62263677;rs62263678;rs574559070;rs534937763;rs62263680;rs62263682;rs62263684;rs16836982;rs16836986;rs112527011;rs62263685;rs62263686 | NA | 0 |
| 2 | 3:96680001-96720000 | 3:94440001-94480000 | 1.29E-13 | HiC | GSE87112 | Mesenchymal_Stem_Cell | intra | rs62263705;rs62263706;rs6762348;rs62263709;rs137983052;rs62263734;rs2856474;rs2612274;rs553308194 | NA | 0 |
| 2 | 3:96520001-96560000 | 3:94480001-94520000 | 6.51E-26 | HiC | GSE87112 | Mesenchymal_Stem_Cell | intra | rs62262901;rs62262902;rs62262903;rs62262930;rs151165295;rs62262931;rs62262933;rs62262934;rs74641574;rs79645223;rs2318156;rs2318155;rs2318154 | NA | 0 |
| 2 | 3:96560001-96600000 | 3:94480001-94520000 | 1.09E-12 | HiC | GSE87112 | Mesenchymal_Stem_Cell | intra | rs16836353;rs62262935;rs62262936;rs62262937;rs62262938;rs62262940;rs62262941;rs62262942;rs62262944;rs62262946;rs62262947;rs62262948;rs62262949;rs62262950;rs62262951 | NA | 0 |
| 2 | 3:96600001-96640000 | 3:94480001-94520000 | 1.31E-10 | HiC | GSE87112 | Mesenchymal_Stem_Cell | intra | rs62262952;rs62262953;rs116253310;rs76857958;rs111706803;rs62262955;rs62262957;rs62262958;rs62263676;rs187619159;rs62263677;rs62263678;rs574559070;rs534937763;rs62263680;rs62263682;rs62263684;rs16836982;rs16836986;rs112527011;rs62263685;rs62263686 | NA | 0 |
| 2 | 3:96680001-96720000 | 3:94480001-94520000 | 1.20E-14 | HiC | GSE87112 | Mesenchymal_Stem_Cell | intra | rs62263705;rs62263706;rs6762348;rs62263709;rs137983052;rs62263734;rs2856474;rs2612274;rs553308194 | NA | 0 |
| 2 | 3:96520001-96560000 | 3:94520001-94560000 | 7.74E-60 | HiC | GSE87112 | Mesenchymal_Stem_Cell | intra | rs62262901;rs62262902;rs62262903;rs62262930;rs151165295;rs62262931;rs62262933;rs62262934;rs74641574;rs79645223;rs2318156;rs2318155;rs2318154 | NA | 0 |
| 2 | 3:96560001-96600000 | 3:94520001-94560000 | 5.80E-23 | HiC | GSE87112 | Mesenchymal_Stem_Cell | intra | rs16836353;rs62262935;rs62262936;rs62262937;rs62262938;rs62262940;rs62262941;rs62262942;rs62262944;rs62262946;rs62262947;rs62262948;rs62262949;rs62262950;rs62262951 | NA | 0 |
| 2 | 3:96600001-96640000 | 3:94520001-94560000 | 1.22E-28 | HiC | GSE87112 | Mesenchymal_Stem_Cell | intra | rs62262952;rs62262953;rs116253310;rs76857958;rs111706803;rs62262955;rs62262957;rs62262958;rs62263676;rs187619159;rs62263677;rs62263678;rs574559070;rs534937763;rs62263680;rs62263682;rs62263684;rs16836982;rs16836986;rs112527011;rs62263685;rs62263686 | NA | 0 |
| 2 | 3:96680001-96720000 | 3:94520001-94560000 | 1.08E-32 | HiC | GSE87112 | Mesenchymal_Stem_Cell | intra | rs62263705;rs62263706;rs6762348;rs62263709;rs137983052;rs62263734;rs2856474;rs2612274;rs553308194 | NA | 0 |
| 2 | 3:96520001-96560000 | 3:94560001-94600000 | 9.67E-21 | HiC | GSE87112 | Mesenchymal_Stem_Cell | intra | rs62262901;rs62262902;rs62262903;rs62262930;rs151165295;rs62262931;rs62262933;rs62262934;rs74641574;rs79645223;rs2318156;rs2318155;rs2318154 | NA | 0 |
| 2 | 3:96560001-96600000 | 3:94560001-94600000 | 2.42E-13 | HiC | GSE87112 | Mesenchymal_Stem_Cell | intra | rs16836353;rs62262935;rs62262936;rs62262937;rs62262938;rs62262940;rs62262941;rs62262942;rs62262944;rs62262946;rs62262947;rs62262948;rs62262949;rs62262950;rs62262951 | NA | 0 |
| 2 | 3:96680001-96720000 | 3:94560001-94600000 | 1.53E-13 | HiC | GSE87112 | Mesenchymal_Stem_Cell | intra | rs62263705;rs62263706;rs6762348;rs62263709;rs137983052;rs62263734;rs2856474;rs2612274;rs553308194 | NA | 0 |
| 2 | 3:96520001-96560000 | 3:94600001-94640000 | 8.83E-51 | HiC | GSE87112 | Mesenchymal_Stem_Cell | intra | rs62262901;rs62262902;rs62262903;rs62262930;rs151165295;rs62262931;rs62262933;rs62262934;rs74641574;rs79645223;rs2318156;rs2318155;rs2318154 | NA | 0 |
| 2 | 3:96560001-96600000 | 3:94600001-94640000 | 3.07E-14 | HiC | GSE87112 | Mesenchymal_Stem_Cell | intra | rs16836353;rs62262935;rs62262936;rs62262937;rs62262938;rs62262940;rs62262941;rs62262942;rs62262944;rs62262946;rs62262947;rs62262948;rs62262949;rs62262950;rs62262951 | NA | 0 |
| 2 | 3:96600001-96640000 | 3:94600001-94640000 | 2.80E-23 | HiC | GSE87112 | Mesenchymal_Stem_Cell | intra | rs62262952;rs62262953;rs116253310;rs76857958;rs111706803;rs62262955;rs62262957;rs62262958;rs62263676;rs187619159;rs62263677;rs62263678;rs574559070;rs534937763;rs62263680;rs62263682;rs62263684;rs16836982;rs16836986;rs112527011;rs62263685;rs62263686 | NA | 0 |
| 2 | 3:96680001-96720000 | 3:94600001-94640000 | 2.04E-18 | HiC | GSE87112 | Mesenchymal_Stem_Cell | intra | rs62263705;rs62263706;rs6762348;rs62263709;rs137983052;rs62263734;rs2856474;rs2612274;rs553308194 | NA | 0 |
| 2 | 3:96520001-96560000 | 3:94640001-94680000 | 1.22E-72 | HiC | GSE87112 | Mesenchymal_Stem_Cell | intra | rs62262901;rs62262902;rs62262903;rs62262930;rs151165295;rs62262931;rs62262933;rs62262934;rs74641574;rs79645223;rs2318156;rs2318155;rs2318154 | NA | 0 |
| 2 | 3:96560001-96600000 | 3:94640001-94680000 | 1.19E-17 | HiC | GSE87112 | Mesenchymal_Stem_Cell | intra | rs16836353;rs62262935;rs62262936;rs62262937;rs62262938;rs62262940;rs62262941;rs62262942;rs62262944;rs62262946;rs62262947;rs62262948;rs62262949;rs62262950;rs62262951 | NA | 0 |
| 2 | 3:96600001-96640000 | 3:94640001-94680000 | 8.98E-29 | HiC | GSE87112 | Mesenchymal_Stem_Cell | intra | rs62262952;rs62262953;rs116253310;rs76857958;rs111706803;rs62262955;rs62262957;rs62262958;rs62263676;rs187619159;rs62263677;rs62263678;rs574559070;rs534937763;rs62263680;rs62263682;rs62263684;rs16836982;rs16836986;rs112527011;rs62263685;rs62263686 | NA | 0 |
| 2 | 3:96680001-96720000 | 3:94640001-94680000 | 6.04E-33 | HiC | GSE87112 | Mesenchymal_Stem_Cell | intra | rs62263705;rs62263706;rs6762348;rs62263709;rs137983052;rs62263734;rs2856474;rs2612274;rs553308194 | NA | 0 |
| 2 | 3:96720001-96760000 | 3:94640001-94680000 | 7.53E-10 | HiC | GSE87112 | Mesenchymal_Stem_Cell | intra | rs2027816;rs2856463;rs2019818;rs115680726;rs112449918;rs565327317;rs2213251;rs2213252;rs2856466 | NA | 0 |
| 2 | 3:96520001-96560000 | 3:94680001-94720000 | 1.88E-46 | HiC | GSE87112 | Mesenchymal_Stem_Cell | intra | rs62262901;rs62262902;rs62262903;rs62262930;rs151165295;rs62262931;rs62262933;rs62262934;rs74641574;rs79645223;rs2318156;rs2318155;rs2318154 | NA | 0 |
| 2 | 3:96560001-96600000 | 3:94680001-94720000 | 1.53E-20 | HiC | GSE87112 | Mesenchymal_Stem_Cell | intra | rs16836353;rs62262935;rs62262936;rs62262937;rs62262938;rs62262940;rs62262941;rs62262942;rs62262944;rs62262946;rs62262947;rs62262948;rs62262949;rs62262950;rs62262951 | NA | 0 |
| 2 | 3:96600001-96640000 | 3:94680001-94720000 | 6.68E-33 | HiC | GSE87112 | Mesenchymal_Stem_Cell | intra | rs62262952;rs62262953;rs116253310;rs76857958;rs111706803;rs62262955;rs62262957;rs62262958;rs62263676;rs187619159;rs62263677;rs62263678;rs574559070;rs534937763;rs62263680;rs62263682;rs62263684;rs16836982;rs16836986;rs112527011;rs62263685;rs62263686 | NA | 0 |
| 2 | 3:96640001-96680000 | 3:94680001-94720000 | 1.11E-07 | HiC | GSE87112 | Mesenchymal_Stem_Cell | intra | rs62263687;rs62263693;rs2318066;rs62263694;rs74563548;rs62263696;rs185127539;rs145486125;rs62263697;rs148690862;rs62263699;rs149222683;rs111574505;rs62263701;rs62263702;rs62263703;rs75261331;rs62263704;rs189373463 | NA | 0 |
| 2 | 3:96680001-96720000 | 3:94680001-94720000 | 3.10E-33 | HiC | GSE87112 | Mesenchymal_Stem_Cell | intra | rs62263705;rs62263706;rs6762348;rs62263709;rs137983052;rs62263734;rs2856474;rs2612274;rs553308194 | NA | 0 |
| 2 | 3:96520001-96560000 | 3:94760001-94800000 | 1.64E-16 | HiC | GSE87112 | Mesenchymal_Stem_Cell | intra | rs62262901;rs62262902;rs62262903;rs62262930;rs151165295;rs62262931;rs62262933;rs62262934;rs74641574;rs79645223;rs2318156;rs2318155;rs2318154 | NA | 0 |
| 2 | 3:96680001-96720000 | 3:94760001-94800000 | 1.94E-09 | HiC | GSE87112 | Mesenchymal_Stem_Cell | intra | rs62263705;rs62263706;rs6762348;rs62263709;rs137983052;rs62263734;rs2856474;rs2612274;rs553308194 | NA | 0 |
| 2 | 3:96520001-96560000 | 3:94800001-94840000 | 1.38E-35 | HiC | GSE87112 | Mesenchymal_Stem_Cell | intra | rs62262901;rs62262902;rs62262903;rs62262930;rs151165295;rs62262931;rs62262933;rs62262934;rs74641574;rs79645223;rs2318156;rs2318155;rs2318154 | NA | 0 |
| 2 | 3:96600001-96640000 | 3:94800001-94840000 | 9.59E-07 | HiC | GSE87112 | Mesenchymal_Stem_Cell | intra | rs62262952;rs62262953;rs116253310;rs76857958;rs111706803;rs62262955;rs62262957;rs62262958;rs62263676;rs187619159;rs62263677;rs62263678;rs574559070;rs534937763;rs62263680;rs62263682;rs62263684;rs16836982;rs16836986;rs112527011;rs62263685;rs62263686 | NA | 0 |
| 2 | 3:96680001-96720000 | 3:94800001-94840000 | 1.54E-18 | HiC | GSE87112 | Mesenchymal_Stem_Cell | intra | rs62263705;rs62263706;rs6762348;rs62263709;rs137983052;rs62263734;rs2856474;rs2612274;rs553308194 | NA | 0 |
| 2 | 3:96520001-96560000 | 3:94840001-94880000 | 6.31E-31 | HiC | GSE87112 | Mesenchymal_Stem_Cell | intra | rs62262901;rs62262902;rs62262903;rs62262930;rs151165295;rs62262931;rs62262933;rs62262934;rs74641574;rs79645223;rs2318156;rs2318155;rs2318154 | NA | 0 |
| 2 | 3:96560001-96600000 | 3:94840001-94880000 | 7.93E-15 | HiC | GSE87112 | Mesenchymal_Stem_Cell | intra | rs16836353;rs62262935;rs62262936;rs62262937;rs62262938;rs62262940;rs62262941;rs62262942;rs62262944;rs62262946;rs62262947;rs62262948;rs62262949;rs62262950;rs62262951 | NA | 0 |
| 2 | 3:96600001-96640000 | 3:94840001-94880000 | 7.68E-26 | HiC | GSE87112 | Mesenchymal_Stem_Cell | intra | rs62262952;rs62262953;rs116253310;rs76857958;rs111706803;rs62262955;rs62262957;rs62262958;rs62263676;rs187619159;rs62263677;rs62263678;rs574559070;rs534937763;rs62263680;rs62263682;rs62263684;rs16836982;rs16836986;rs112527011;rs62263685;rs62263686 | NA | 0 |
| 2 | 3:96680001-96720000 | 3:94840001-94880000 | 4.32E-18 | HiC | GSE87112 | Mesenchymal_Stem_Cell | intra | rs62263705;rs62263706;rs6762348;rs62263709;rs137983052;rs62263734;rs2856474;rs2612274;rs553308194 | NA | 0 |
| 2 | 3:96520001-96560000 | 3:94880001-94920000 | 1.26E-10 | HiC | GSE87112 | Mesenchymal_Stem_Cell | intra | rs62262901;rs62262902;rs62262903;rs62262930;rs151165295;rs62262931;rs62262933;rs62262934;rs74641574;rs79645223;rs2318156;rs2318155;rs2318154 | NA | 0 |
| 2 | 3:96600001-96640000 | 3:94880001-94920000 | 7.42E-18 | HiC | GSE87112 | Mesenchymal_Stem_Cell | intra | rs62262952;rs62262953;rs116253310;rs76857958;rs111706803;rs62262955;rs62262957;rs62262958;rs62263676;rs187619159;rs62263677;rs62263678;rs574559070;rs534937763;rs62263680;rs62263682;rs62263684;rs16836982;rs16836986;rs112527011;rs62263685;rs62263686 | NA | 0 |
| 2 | 3:96680001-96720000 | 3:94880001-94920000 | 1.20E-07 | HiC | GSE87112 | Mesenchymal_Stem_Cell | intra | rs62263705;rs62263706;rs6762348;rs62263709;rs137983052;rs62263734;rs2856474;rs2612274;rs553308194 | NA | 0 |
| 2 | 3:96520001-96560000 | 3:94920001-94960000 | 6.27E-30 | HiC | GSE87112 | Mesenchymal_Stem_Cell | intra | rs62262901;rs62262902;rs62262903;rs62262930;rs151165295;rs62262931;rs62262933;rs62262934;rs74641574;rs79645223;rs2318156;rs2318155;rs2318154 | NA | 0 |
| 2 | 3:96560001-96600000 | 3:94920001-94960000 | 1.17E-11 | HiC | GSE87112 | Mesenchymal_Stem_Cell | intra | rs16836353;rs62262935;rs62262936;rs62262937;rs62262938;rs62262940;rs62262941;rs62262942;rs62262944;rs62262946;rs62262947;rs62262948;rs62262949;rs62262950;rs62262951 | NA | 0 |
| 2 | 3:96600001-96640000 | 3:94920001-94960000 | 2.66E-17 | HiC | GSE87112 | Mesenchymal_Stem_Cell | intra | rs62262952;rs62262953;rs116253310;rs76857958;rs111706803;rs62262955;rs62262957;rs62262958;rs62263676;rs187619159;rs62263677;rs62263678;rs574559070;rs534937763;rs62263680;rs62263682;rs62263684;rs16836982;rs16836986;rs112527011;rs62263685;rs62263686 | NA | 0 |
| 2 | 3:96680001-96720000 | 3:94920001-94960000 | 1.64E-16 | HiC | GSE87112 | Mesenchymal_Stem_Cell | intra | rs62263705;rs62263706;rs6762348;rs62263709;rs137983052;rs62263734;rs2856474;rs2612274;rs553308194 | NA | 0 |
| 2 | 3:96520001-96560000 | 3:94960001-95000000 | 2.12E-14 | HiC | GSE87112 | Mesenchymal_Stem_Cell | intra | rs62262901;rs62262902;rs62262903;rs62262930;rs151165295;rs62262931;rs62262933;rs62262934;rs74641574;rs79645223;rs2318156;rs2318155;rs2318154 | NA | 0 |
| 2 | 3:96680001-96720000 | 3:94960001-95000000 | 5.26E-12 | HiC | GSE87112 | Mesenchymal_Stem_Cell | intra | rs62263705;rs62263706;rs6762348;rs62263709;rs137983052;rs62263734;rs2856474;rs2612274;rs553308194 | NA | 0 |
| 2 | 3:96520001-96560000 | 3:95000001-95040000 | 3.76E-42 | HiC | GSE87112 | Mesenchymal_Stem_Cell | intra | rs62262901;rs62262902;rs62262903;rs62262930;rs151165295;rs62262931;rs62262933;rs62262934;rs74641574;rs79645223;rs2318156;rs2318155;rs2318154 | NA | 0 |
| 2 | 3:96560001-96600000 | 3:95000001-95040000 | 6.66E-08 | HiC | GSE87112 | Mesenchymal_Stem_Cell | intra | rs16836353;rs62262935;rs62262936;rs62262937;rs62262938;rs62262940;rs62262941;rs62262942;rs62262944;rs62262946;rs62262947;rs62262948;rs62262949;rs62262950;rs62262951 | NA | 0 |
| 2 | 3:96600001-96640000 | 3:95000001-95040000 | 1.05E-11 | HiC | GSE87112 | Mesenchymal_Stem_Cell | intra | rs62262952;rs62262953;rs116253310;rs76857958;rs111706803;rs62262955;rs62262957;rs62262958;rs62263676;rs187619159;rs62263677;rs62263678;rs574559070;rs534937763;rs62263680;rs62263682;rs62263684;rs16836982;rs16836986;rs112527011;rs62263685;rs62263686 | NA | 0 |
| 2 | 3:96680001-96720000 | 3:95000001-95040000 | 1.08E-16 | HiC | GSE87112 | Mesenchymal_Stem_Cell | intra | rs62263705;rs62263706;rs6762348;rs62263709;rs137983052;rs62263734;rs2856474;rs2612274;rs553308194 | NA | 0 |
| 2 | 3:96520001-96560000 | 3:95040001-95080000 | 4.98E-20 | HiC | GSE87112 | Mesenchymal_Stem_Cell | intra | rs62262901;rs62262902;rs62262903;rs62262930;rs151165295;rs62262931;rs62262933;rs62262934;rs74641574;rs79645223;rs2318156;rs2318155;rs2318154 | NA | 0 |
| 2 | 3:96600001-96640000 | 3:95040001-95080000 | 9.75E-10 | HiC | GSE87112 | Mesenchymal_Stem_Cell | intra | rs62262952;rs62262953;rs116253310;rs76857958;rs111706803;rs62262955;rs62262957;rs62262958;rs62263676;rs187619159;rs62263677;rs62263678;rs574559070;rs534937763;rs62263680;rs62263682;rs62263684;rs16836982;rs16836986;rs112527011;rs62263685;rs62263686 | NA | 0 |
| 2 | 3:96680001-96720000 | 3:95040001-95080000 | 2.84E-13 | HiC | GSE87112 | Mesenchymal_Stem_Cell | intra | rs62263705;rs62263706;rs6762348;rs62263709;rs137983052;rs62263734;rs2856474;rs2612274;rs553308194 | NA | 0 |
| 2 | 3:96520001-96560000 | 3:95120001-95160000 | 6.83E-46 | HiC | GSE87112 | Mesenchymal_Stem_Cell | intra | rs62262901;rs62262902;rs62262903;rs62262930;rs151165295;rs62262931;rs62262933;rs62262934;rs74641574;rs79645223;rs2318156;rs2318155;rs2318154 | NA | 0 |
| 2 | 3:96560001-96600000 | 3:95120001-95160000 | 2.40E-08 | HiC | GSE87112 | Mesenchymal_Stem_Cell | intra | rs16836353;rs62262935;rs62262936;rs62262937;rs62262938;rs62262940;rs62262941;rs62262942;rs62262944;rs62262946;rs62262947;rs62262948;rs62262949;rs62262950;rs62262951 | NA | 0 |
| 2 | 3:96600001-96640000 | 3:95120001-95160000 | 8.02E-19 | HiC | GSE87112 | Mesenchymal_Stem_Cell | intra | rs62262952;rs62262953;rs116253310;rs76857958;rs111706803;rs62262955;rs62262957;rs62262958;rs62263676;rs187619159;rs62263677;rs62263678;rs574559070;rs534937763;rs62263680;rs62263682;rs62263684;rs16836982;rs16836986;rs112527011;rs62263685;rs62263686 | NA | 0 |
| 2 | 3:96680001-96720000 | 3:95120001-95160000 | 1.80E-18 | HiC | GSE87112 | Mesenchymal_Stem_Cell | intra | rs62263705;rs62263706;rs6762348;rs62263709;rs137983052;rs62263734;rs2856474;rs2612274;rs553308194 | NA | 0 |
| 2 | 3:96520001-96560000 | 3:95160001-95200000 | 2.39E-11 | HiC | GSE87112 | Mesenchymal_Stem_Cell | intra | rs62262901;rs62262902;rs62262903;rs62262930;rs151165295;rs62262931;rs62262933;rs62262934;rs74641574;rs79645223;rs2318156;rs2318155;rs2318154 | NA | 0 |
| 2 | 3:96680001-96720000 | 3:95160001-95200000 | 9.51E-10 | HiC | GSE87112 | Mesenchymal_Stem_Cell | intra | rs62263705;rs62263706;rs6762348;rs62263709;rs137983052;rs62263734;rs2856474;rs2612274;rs553308194 | NA | 0 |
| 2 | 3:96520001-96560000 | 3:95200001-95240000 | 3.45E-48 | HiC | GSE87112 | Mesenchymal_Stem_Cell | intra | rs62262901;rs62262902;rs62262903;rs62262930;rs151165295;rs62262931;rs62262933;rs62262934;rs74641574;rs79645223;rs2318156;rs2318155;rs2318154 | NA | 0 |
| 2 | 3:96560001-96600000 | 3:95200001-95240000 | 2.39E-11 | HiC | GSE87112 | Mesenchymal_Stem_Cell | intra | rs16836353;rs62262935;rs62262936;rs62262937;rs62262938;rs62262940;rs62262941;rs62262942;rs62262944;rs62262946;rs62262947;rs62262948;rs62262949;rs62262950;rs62262951 | NA | 0 |
| 2 | 3:96600001-96640000 | 3:95200001-95240000 | 1.58E-14 | HiC | GSE87112 | Mesenchymal_Stem_Cell | intra | rs62262952;rs62262953;rs116253310;rs76857958;rs111706803;rs62262955;rs62262957;rs62262958;rs62263676;rs187619159;rs62263677;rs62263678;rs574559070;rs534937763;rs62263680;rs62263682;rs62263684;rs16836982;rs16836986;rs112527011;rs62263685;rs62263686 | NA | 0 |
| 2 | 3:96680001-96720000 | 3:95200001-95240000 | 8.18E-15 | HiC | GSE87112 | Mesenchymal_Stem_Cell | intra | rs62263705;rs62263706;rs6762348;rs62263709;rs137983052;rs62263734;rs2856474;rs2612274;rs553308194 | NA | 0 |
| 2 | 3:96520001-96560000 | 3:95240001-95280000 | 6.77E-29 | HiC | GSE87112 | Mesenchymal_Stem_Cell | intra | rs62262901;rs62262902;rs62262903;rs62262930;rs151165295;rs62262931;rs62262933;rs62262934;rs74641574;rs79645223;rs2318156;rs2318155;rs2318154 | NA | 0 |
| 2 | 3:96600001-96640000 | 3:95240001-95280000 | 5.90E-10 | HiC | GSE87112 | Mesenchymal_Stem_Cell | intra | rs62262952;rs62262953;rs116253310;rs76857958;rs111706803;rs62262955;rs62262957;rs62262958;rs62263676;rs187619159;rs62263677;rs62263678;rs574559070;rs534937763;rs62263680;rs62263682;rs62263684;rs16836982;rs16836986;rs112527011;rs62263685;rs62263686 | NA | 0 |
| 2 | 3:96680001-96720000 | 3:95240001-95280000 | 6.70E-17 | HiC | GSE87112 | Mesenchymal_Stem_Cell | intra | rs62263705;rs62263706;rs6762348;rs62263709;rs137983052;rs62263734;rs2856474;rs2612274;rs553308194 | NA | 0 |
| 2 | 3:96520001-96560000 | 3:95280001-95320000 | 8.84E-19 | HiC | GSE87112 | Mesenchymal_Stem_Cell | intra | rs62262901;rs62262902;rs62262903;rs62262930;rs151165295;rs62262931;rs62262933;rs62262934;rs74641574;rs79645223;rs2318156;rs2318155;rs2318154 | NA | 0 |
| 2 | 3:96600001-96640000 | 3:95280001-95320000 | 4.05E-08 | HiC | GSE87112 | Mesenchymal_Stem_Cell | intra | rs62262952;rs62262953;rs116253310;rs76857958;rs111706803;rs62262955;rs62262957;rs62262958;rs62263676;rs187619159;rs62263677;rs62263678;rs574559070;rs534937763;rs62263680;rs62263682;rs62263684;rs16836982;rs16836986;rs112527011;rs62263685;rs62263686 | NA | 0 |
| 2 | 3:96520001-96560000 | 3:95400001-95440000 | 4.12E-25 | HiC | GSE87112 | Mesenchymal_Stem_Cell | intra | rs62262901;rs62262902;rs62262903;rs62262930;rs151165295;rs62262931;rs62262933;rs62262934;rs74641574;rs79645223;rs2318156;rs2318155;rs2318154 | NA | 0 |
| 2 | 3:96600001-96640000 | 3:95400001-95440000 | 2.89E-09 | HiC | GSE87112 | Mesenchymal_Stem_Cell | intra | rs62262952;rs62262953;rs116253310;rs76857958;rs111706803;rs62262955;rs62262957;rs62262958;rs62263676;rs187619159;rs62263677;rs62263678;rs574559070;rs534937763;rs62263680;rs62263682;rs62263684;rs16836982;rs16836986;rs112527011;rs62263685;rs62263686 | NA | 0 |
| 2 | 3:96680001-96720000 | 3:95400001-95440000 | 7.15E-15 | HiC | GSE87112 | Mesenchymal_Stem_Cell | intra | rs62263705;rs62263706;rs6762348;rs62263709;rs137983052;rs62263734;rs2856474;rs2612274;rs553308194 | NA | 0 |
| 2 | 3:96520001-96560000 | 3:95480001-95520000 | 1.62E-07 | HiC | GSE87112 | Mesenchymal_Stem_Cell | intra | rs62262901;rs62262902;rs62262903;rs62262930;rs151165295;rs62262931;rs62262933;rs62262934;rs74641574;rs79645223;rs2318156;rs2318155;rs2318154 | NA | 0 |
| 2 | 3:96520001-96560000 | 3:95920001-95960000 | 9.49E-12 | HiC | GSE87112 | Mesenchymal_Stem_Cell | intra | rs62262901;rs62262902;rs62262903;rs62262930;rs151165295;rs62262931;rs62262933;rs62262934;rs74641574;rs79645223;rs2318156;rs2318155;rs2318154 | NA | 0 |
| 2 | 3:96680001-96720000 | 3:95920001-95960000 | 9.97E-07 | HiC | GSE87112 | Mesenchymal_Stem_Cell | intra | rs62263705;rs62263706;rs6762348;rs62263709;rs137983052;rs62263734;rs2856474;rs2612274;rs553308194 | NA | 0 |
| 2 | 3:96680001-96720000 | 3:96600001-96640000 | 9.63E-08 | HiC | GSE87112 | Mesenchymal_Stem_Cell | intra | rs62263705;rs62263706;rs6762348;rs62263709;rs137983052;rs62263734;rs2856474;rs2612274;rs553308194 | NA | 0 |
| 3 | 4:171440001-171480000 | 4:171160001-171200000 | 1.30E-33 | HiC | GSE87112 | Mesenchymal_Stem_Cell | intra | rs147274196 | NA | 0 |
| 3 | 4:171480001-171520000 | 4:171160001-171200000 | 5.83E-62 | HiC | GSE87112 | Mesenchymal_Stem_Cell | intra | rs148420952;rs76413646 | NA | 0 |
| 3 | 4:171440001-171480000 | 4:171200001-171240000 | 8.42E-34 | HiC | GSE87112 | Mesenchymal_Stem_Cell | intra | rs147274196 | NA | 0 |
| 3 | 4:171480001-171520000 | 4:171200001-171240000 | 1.30E-31 | HiC | GSE87112 | Mesenchymal_Stem_Cell | intra | rs148420952;rs76413646 | NA | 0 |
| 3 | 4:171440001-171480000 | 4:171240001-171280000 | 2.04E-14 | HiC | GSE87112 | Mesenchymal_Stem_Cell | intra | rs147274196 | NA | 0 |
| 3 | 4:171480001-171520000 | 4:171240001-171280000 | 9.80E-14 | HiC | GSE87112 | Mesenchymal_Stem_Cell | intra | rs148420952;rs76413646 | NA | 0 |
| 3 | 4:171440001-171480000 | 4:171280001-171320000 | 7.12E-20 | HiC | GSE87112 | Mesenchymal_Stem_Cell | intra | rs147274196 | NA | 0 |
| 3 | 4:171480001-171520000 | 4:171280001-171320000 | 6.63E-23 | HiC | GSE87112 | Mesenchymal_Stem_Cell | intra | rs148420952;rs76413646 | NA | 0 |
| 3 | 4:171480001-171520000 | 4:171320001-171360000 | 2.56E-11 | HiC | GSE87112 | Mesenchymal_Stem_Cell | intra | rs148420952;rs76413646 | NA | 0 |
| 3 | 4:171440001-171480000 | 4:171360001-171400000 | 8.85E-07 | HiC | GSE87112 | Mesenchymal_Stem_Cell | intra | rs147274196 | NA | 0 |
| 3 | 4:171440001-171480000 | 4:171400001-171440000 | 2.32E-36 | HiC | GSE87112 | Mesenchymal_Stem_Cell | intra | rs147274196 | NA | 0 |
| 3 | 4:171480001-171520000 | 4:171400001-171440000 | 1.59E-45 | HiC | GSE87112 | Mesenchymal_Stem_Cell | intra | rs148420952;rs76413646 | NA | 0 |
| 3 | 4:171480001-171520000 | 4:171440001-171480000 | 6.92E-29 | HiC | GSE87112 | Mesenchymal_Stem_Cell | intra | rs148420952;rs76413646 | NA | 0 |
| 4 | 5:108560001-108600000 | 5:107720001-107760000 | 3.83E-16 | HiC | GSE87112 | Mesenchymal_Stem_Cell | intra | rs35812497;rs75803484;rs75073283;rs76430100;rs75782477;rs77992445;rs61701247;rs17161655;rs115979287;rs17161659;rs151072743;rs17161665;rs6863688;5:108591279:C:T;5:108591279:A:T;rs1363213;rs1862201;rs1862202;rs1862203;rs5870359 | NA | 0 |
| 4 | 5:108600001-108640000 | 5:107720001-107760000 | 2.32E-08 | HiC | GSE87112 | Mesenchymal_Stem_Cell | intra | rs11744353;rs79776200;rs76069623;rs76043709;rs78729571;rs36123121;rs11748450;rs80234158;rs78146542;rs185072862;rs137867205;rs189006725;rs4388251;rs4438924;rs4541698;rs4388252;rs4392675;rs200252534;rs145520315;rs147699420;rs77803982;rs79469094;rs75077625;rs74379173;rs78664536;rs549536839;rs570631944;rs78784808;rs78340649;rs201569786;rs75649455;rs78814720;rs77561644;rs76565008;rs74436342;rs1592807;rs1592810;rs1592811;rs7700458;rs6594365;rs6863893;rs140163909;rs146837743;rs7705919;rs201046346;rs202152005;rs199869120;rs111726541;rs373673987;rs113026830;rs148211606;rs201619508;rs201169921;rs146644914;rs116649742;rs79893293;rs57629433;rs60335509;rs58453996;rs59466242;rs60878839;rs11743452;rs1833567;rs1833568;rs1833569;rs7721272;rs7721427;rs7721730;rs7721870;rs7722100;rs7704359;rs1895200;rs1895201;rs77974856;rs60905342;rs11746207;rs11739672;rs11749718;rs78518991;rs76243602;rs76383235;rs11741980;rs35243960;rs116462975;rs139801813;rs113103584;rs74727230;rs75919325;rs79624714;rs2080858;rs138092354;rs148285468;rs114455395;rs74850689;rs10463599 | NA | 0 |
| 4 | 5:108560001-108600000 | 5:107760001-107800000 | 2.32E-24 | HiC | GSE87112 | Mesenchymal_Stem_Cell | intra | rs35812497;rs75803484;rs75073283;rs76430100;rs75782477;rs77992445;rs61701247;rs17161655;rs115979287;rs17161659;rs151072743;rs17161665;rs6863688;5:108591279:C:T;5:108591279:A:T;rs1363213;rs1862201;rs1862202;rs1862203;rs5870359 | NA | 0 |
| 4 | 5:108600001-108640000 | 5:107760001-107800000 | 2.42E-25 | HiC | GSE87112 | Mesenchymal_Stem_Cell | intra | rs11744353;rs79776200;rs76069623;rs76043709;rs78729571;rs36123121;rs11748450;rs80234158;rs78146542;rs185072862;rs137867205;rs189006725;rs4388251;rs4438924;rs4541698;rs4388252;rs4392675;rs200252534;rs145520315;rs147699420;rs77803982;rs79469094;rs75077625;rs74379173;rs78664536;rs549536839;rs570631944;rs78784808;rs78340649;rs201569786;rs75649455;rs78814720;rs77561644;rs76565008;rs74436342;rs1592807;rs1592810;rs1592811;rs7700458;rs6594365;rs6863893;rs140163909;rs146837743;rs7705919;rs201046346;rs202152005;rs199869120;rs111726541;rs373673987;rs113026830;rs148211606;rs201619508;rs201169921;rs146644914;rs116649742;rs79893293;rs57629433;rs60335509;rs58453996;rs59466242;rs60878839;rs11743452;rs1833567;rs1833568;rs1833569;rs7721272;rs7721427;rs7721730;rs7721870;rs7722100;rs7704359;rs1895200;rs1895201;rs77974856;rs60905342;rs11746207;rs11739672;rs11749718;rs78518991;rs76243602;rs76383235;rs11741980;rs35243960;rs116462975;rs139801813;rs113103584;rs74727230;rs75919325;rs79624714;rs2080858;rs138092354;rs148285468;rs114455395;rs74850689;rs10463599 | NA | 0 |
| 4 | 5:108560001-108600000 | 5:107800001-107840000 | 8.10E-41 | HiC | GSE87112 | Mesenchymal_Stem_Cell | intra | rs35812497;rs75803484;rs75073283;rs76430100;rs75782477;rs77992445;rs61701247;rs17161655;rs115979287;rs17161659;rs151072743;rs17161665;rs6863688;5:108591279:C:T;5:108591279:A:T;rs1363213;rs1862201;rs1862202;rs1862203;rs5870359 | NA | 0 |
| 4 | 5:108600001-108640000 | 5:107800001-107840000 | 1.55E-33 | HiC | GSE87112 | Mesenchymal_Stem_Cell | intra | rs11744353;rs79776200;rs76069623;rs76043709;rs78729571;rs36123121;rs11748450;rs80234158;rs78146542;rs185072862;rs137867205;rs189006725;rs4388251;rs4438924;rs4541698;rs4388252;rs4392675;rs200252534;rs145520315;rs147699420;rs77803982;rs79469094;rs75077625;rs74379173;rs78664536;rs549536839;rs570631944;rs78784808;rs78340649;rs201569786;rs75649455;rs78814720;rs77561644;rs76565008;rs74436342;rs1592807;rs1592810;rs1592811;rs7700458;rs6594365;rs6863893;rs140163909;rs146837743;rs7705919;rs201046346;rs202152005;rs199869120;rs111726541;rs373673987;rs113026830;rs148211606;rs201619508;rs201169921;rs146644914;rs116649742;rs79893293;rs57629433;rs60335509;rs58453996;rs59466242;rs60878839;rs11743452;rs1833567;rs1833568;rs1833569;rs7721272;rs7721427;rs7721730;rs7721870;rs7722100;rs7704359;rs1895200;rs1895201;rs77974856;rs60905342;rs11746207;rs11739672;rs11749718;rs78518991;rs76243602;rs76383235;rs11741980;rs35243960;rs116462975;rs139801813;rs113103584;rs74727230;rs75919325;rs79624714;rs2080858;rs138092354;rs148285468;rs114455395;rs74850689;rs10463599 | NA | 0 |
| 4 | 5:108560001-108600000 | 5:107840001-107880000 | 3.21E-67 | HiC | GSE87112 | Mesenchymal_Stem_Cell | intra | rs35812497;rs75803484;rs75073283;rs76430100;rs75782477;rs77992445;rs61701247;rs17161655;rs115979287;rs17161659;rs151072743;rs17161665;rs6863688;5:108591279:C:T;5:108591279:A:T;rs1363213;rs1862201;rs1862202;rs1862203;rs5870359 | NA | 0 |
| 4 | 5:108600001-108640000 | 5:107840001-107880000 | 3.22E-40 | HiC | GSE87112 | Mesenchymal_Stem_Cell | intra | rs11744353;rs79776200;rs76069623;rs76043709;rs78729571;rs36123121;rs11748450;rs80234158;rs78146542;rs185072862;rs137867205;rs189006725;rs4388251;rs4438924;rs4541698;rs4388252;rs4392675;rs200252534;rs145520315;rs147699420;rs77803982;rs79469094;rs75077625;rs74379173;rs78664536;rs549536839;rs570631944;rs78784808;rs78340649;rs201569786;rs75649455;rs78814720;rs77561644;rs76565008;rs74436342;rs1592807;rs1592810;rs1592811;rs7700458;rs6594365;rs6863893;rs140163909;rs146837743;rs7705919;rs201046346;rs202152005;rs199869120;rs111726541;rs373673987;rs113026830;rs148211606;rs201619508;rs201169921;rs146644914;rs116649742;rs79893293;rs57629433;rs60335509;rs58453996;rs59466242;rs60878839;rs11743452;rs1833567;rs1833568;rs1833569;rs7721272;rs7721427;rs7721730;rs7721870;rs7722100;rs7704359;rs1895200;rs1895201;rs77974856;rs60905342;rs11746207;rs11739672;rs11749718;rs78518991;rs76243602;rs76383235;rs11741980;rs35243960;rs116462975;rs139801813;rs113103584;rs74727230;rs75919325;rs79624714;rs2080858;rs138092354;rs148285468;rs114455395;rs74850689;rs10463599 | NA | 0 |
| 4 | 5:108560001-108600000 | 5:107880001-107920000 | 2.04E-26 | HiC | GSE87112 | Mesenchymal_Stem_Cell | intra | rs35812497;rs75803484;rs75073283;rs76430100;rs75782477;rs77992445;rs61701247;rs17161655;rs115979287;rs17161659;rs151072743;rs17161665;rs6863688;5:108591279:C:T;5:108591279:A:T;rs1363213;rs1862201;rs1862202;rs1862203;rs5870359 | NA | 0 |
| 4 | 5:108600001-108640000 | 5:107880001-107920000 | 5.38E-24 | HiC | GSE87112 | Mesenchymal_Stem_Cell | intra | rs11744353;rs79776200;rs76069623;rs76043709;rs78729571;rs36123121;rs11748450;rs80234158;rs78146542;rs185072862;rs137867205;rs189006725;rs4388251;rs4438924;rs4541698;rs4388252;rs4392675;rs200252534;rs145520315;rs147699420;rs77803982;rs79469094;rs75077625;rs74379173;rs78664536;rs549536839;rs570631944;rs78784808;rs78340649;rs201569786;rs75649455;rs78814720;rs77561644;rs76565008;rs74436342;rs1592807;rs1592810;rs1592811;rs7700458;rs6594365;rs6863893;rs140163909;rs146837743;rs7705919;rs201046346;rs202152005;rs199869120;rs111726541;rs373673987;rs113026830;rs148211606;rs201619508;rs201169921;rs146644914;rs116649742;rs79893293;rs57629433;rs60335509;rs58453996;rs59466242;rs60878839;rs11743452;rs1833567;rs1833568;rs1833569;rs7721272;rs7721427;rs7721730;rs7721870;rs7722100;rs7704359;rs1895200;rs1895201;rs77974856;rs60905342;rs11746207;rs11739672;rs11749718;rs78518991;rs76243602;rs76383235;rs11741980;rs35243960;rs116462975;rs139801813;rs113103584;rs74727230;rs75919325;rs79624714;rs2080858;rs138092354;rs148285468;rs114455395;rs74850689;rs10463599 | NA | 0 |
| 4 | 5:108560001-108600000 | 5:107920001-107960000 | 5.10E-49 | HiC | GSE87112 | Mesenchymal_Stem_Cell | intra | rs35812497;rs75803484;rs75073283;rs76430100;rs75782477;rs77992445;rs61701247;rs17161655;rs115979287;rs17161659;rs151072743;rs17161665;rs6863688;5:108591279:C:T;5:108591279:A:T;rs1363213;rs1862201;rs1862202;rs1862203;rs5870359 | NA | 0 |
| 4 | 5:108600001-108640000 | 5:107920001-107960000 | 6.15E-29 | HiC | GSE87112 | Mesenchymal_Stem_Cell | intra | rs11744353;rs79776200;rs76069623;rs76043709;rs78729571;rs36123121;rs11748450;rs80234158;rs78146542;rs185072862;rs137867205;rs189006725;rs4388251;rs4438924;rs4541698;rs4388252;rs4392675;rs200252534;rs145520315;rs147699420;rs77803982;rs79469094;rs75077625;rs74379173;rs78664536;rs549536839;rs570631944;rs78784808;rs78340649;rs201569786;rs75649455;rs78814720;rs77561644;rs76565008;rs74436342;rs1592807;rs1592810;rs1592811;rs7700458;rs6594365;rs6863893;rs140163909;rs146837743;rs7705919;rs201046346;rs202152005;rs199869120;rs111726541;rs373673987;rs113026830;rs148211606;rs201619508;rs201169921;rs146644914;rs116649742;rs79893293;rs57629433;rs60335509;rs58453996;rs59466242;rs60878839;rs11743452;rs1833567;rs1833568;rs1833569;rs7721272;rs7721427;rs7721730;rs7721870;rs7722100;rs7704359;rs1895200;rs1895201;rs77974856;rs60905342;rs11746207;rs11739672;rs11749718;rs78518991;rs76243602;rs76383235;rs11741980;rs35243960;rs116462975;rs139801813;rs113103584;rs74727230;rs75919325;rs79624714;rs2080858;rs138092354;rs148285468;rs114455395;rs74850689;rs10463599 | NA | 0 |
| 4 | 5:108560001-108600000 | 5:107960001-108000000 | 4.84E-145 | HiC | GSE87112 | Mesenchymal_Stem_Cell | intra | rs35812497;rs75803484;rs75073283;rs76430100;rs75782477;rs77992445;rs61701247;rs17161655;rs115979287;rs17161659;rs151072743;rs17161665;rs6863688;5:108591279:C:T;5:108591279:A:T;rs1363213;rs1862201;rs1862202;rs1862203;rs5870359 | NA | 0 |
| 4 | 5:108600001-108640000 | 5:107960001-108000000 | 7.41E-83 | HiC | GSE87112 | Mesenchymal_Stem_Cell | intra | rs11744353;rs79776200;rs76069623;rs76043709;rs78729571;rs36123121;rs11748450;rs80234158;rs78146542;rs185072862;rs137867205;rs189006725;rs4388251;rs4438924;rs4541698;rs4388252;rs4392675;rs200252534;rs145520315;rs147699420;rs77803982;rs79469094;rs75077625;rs74379173;rs78664536;rs549536839;rs570631944;rs78784808;rs78340649;rs201569786;rs75649455;rs78814720;rs77561644;rs76565008;rs74436342;rs1592807;rs1592810;rs1592811;rs7700458;rs6594365;rs6863893;rs140163909;rs146837743;rs7705919;rs201046346;rs202152005;rs199869120;rs111726541;rs373673987;rs113026830;rs148211606;rs201619508;rs201169921;rs146644914;rs116649742;rs79893293;rs57629433;rs60335509;rs58453996;rs59466242;rs60878839;rs11743452;rs1833567;rs1833568;rs1833569;rs7721272;rs7721427;rs7721730;rs7721870;rs7722100;rs7704359;rs1895200;rs1895201;rs77974856;rs60905342;rs11746207;rs11739672;rs11749718;rs78518991;rs76243602;rs76383235;rs11741980;rs35243960;rs116462975;rs139801813;rs113103584;rs74727230;rs75919325;rs79624714;rs2080858;rs138092354;rs148285468;rs114455395;rs74850689;rs10463599 | NA | 0 |
| 4 | 5:108560001-108600000 | 5:108000001-108040000 | 4.25E-24 | HiC | GSE87112 | Mesenchymal_Stem_Cell | intra | rs35812497;rs75803484;rs75073283;rs76430100;rs75782477;rs77992445;rs61701247;rs17161655;rs115979287;rs17161659;rs151072743;rs17161665;rs6863688;5:108591279:C:T;5:108591279:A:T;rs1363213;rs1862201;rs1862202;rs1862203;rs5870359 | NA | 0 |
| 4 | 5:108600001-108640000 | 5:108000001-108040000 | 5.29E-18 | HiC | GSE87112 | Mesenchymal_Stem_Cell | intra | rs11744353;rs79776200;rs76069623;rs76043709;rs78729571;rs36123121;rs11748450;rs80234158;rs78146542;rs185072862;rs137867205;rs189006725;rs4388251;rs4438924;rs4541698;rs4388252;rs4392675;rs200252534;rs145520315;rs147699420;rs77803982;rs79469094;rs75077625;rs74379173;rs78664536;rs549536839;rs570631944;rs78784808;rs78340649;rs201569786;rs75649455;rs78814720;rs77561644;rs76565008;rs74436342;rs1592807;rs1592810;rs1592811;rs7700458;rs6594365;rs6863893;rs140163909;rs146837743;rs7705919;rs201046346;rs202152005;rs199869120;rs111726541;rs373673987;rs113026830;rs148211606;rs201619508;rs201169921;rs146644914;rs116649742;rs79893293;rs57629433;rs60335509;rs58453996;rs59466242;rs60878839;rs11743452;rs1833567;rs1833568;rs1833569;rs7721272;rs7721427;rs7721730;rs7721870;rs7722100;rs7704359;rs1895200;rs1895201;rs77974856;rs60905342;rs11746207;rs11739672;rs11749718;rs78518991;rs76243602;rs76383235;rs11741980;rs35243960;rs116462975;rs139801813;rs113103584;rs74727230;rs75919325;rs79624714;rs2080858;rs138092354;rs148285468;rs114455395;rs74850689;rs10463599 | NA | 0 |
| 4 | 5:108560001-108600000 | 5:108040001-108080000 | 1.95E-20 | HiC | GSE87112 | Mesenchymal_Stem_Cell | intra | rs35812497;rs75803484;rs75073283;rs76430100;rs75782477;rs77992445;rs61701247;rs17161655;rs115979287;rs17161659;rs151072743;rs17161665;rs6863688;5:108591279:C:T;5:108591279:A:T;rs1363213;rs1862201;rs1862202;rs1862203;rs5870359 | NA | 0 |
| 4 | 5:108600001-108640000 | 5:108040001-108080000 | 7.59E-16 | HiC | GSE87112 | Mesenchymal_Stem_Cell | intra | rs11744353;rs79776200;rs76069623;rs76043709;rs78729571;rs36123121;rs11748450;rs80234158;rs78146542;rs185072862;rs137867205;rs189006725;rs4388251;rs4438924;rs4541698;rs4388252;rs4392675;rs200252534;rs145520315;rs147699420;rs77803982;rs79469094;rs75077625;rs74379173;rs78664536;rs549536839;rs570631944;rs78784808;rs78340649;rs201569786;rs75649455;rs78814720;rs77561644;rs76565008;rs74436342;rs1592807;rs1592810;rs1592811;rs7700458;rs6594365;rs6863893;rs140163909;rs146837743;rs7705919;rs201046346;rs202152005;rs199869120;rs111726541;rs373673987;rs113026830;rs148211606;rs201619508;rs201169921;rs146644914;rs116649742;rs79893293;rs57629433;rs60335509;rs58453996;rs59466242;rs60878839;rs11743452;rs1833567;rs1833568;rs1833569;rs7721272;rs7721427;rs7721730;rs7721870;rs7722100;rs7704359;rs1895200;rs1895201;rs77974856;rs60905342;rs11746207;rs11739672;rs11749718;rs78518991;rs76243602;rs76383235;rs11741980;rs35243960;rs116462975;rs139801813;rs113103584;rs74727230;rs75919325;rs79624714;rs2080858;rs138092354;rs148285468;rs114455395;rs74850689;rs10463599 | NA | 0 |
| 4 | 5:108560001-108600000 | 5:108080001-108120000 | 1.97E-27 | HiC | GSE87112 | Mesenchymal_Stem_Cell | intra | rs35812497;rs75803484;rs75073283;rs76430100;rs75782477;rs77992445;rs61701247;rs17161655;rs115979287;rs17161659;rs151072743;rs17161665;rs6863688;5:108591279:C:T;5:108591279:A:T;rs1363213;rs1862201;rs1862202;rs1862203;rs5870359 | ENSG00000151422 | 0 |
| 4 | 5:108600001-108640000 | 5:108080001-108120000 | 2.54E-13 | HiC | GSE87112 | Mesenchymal_Stem_Cell | intra | rs11744353;rs79776200;rs76069623;rs76043709;rs78729571;rs36123121;rs11748450;rs80234158;rs78146542;rs185072862;rs137867205;rs189006725;rs4388251;rs4438924;rs4541698;rs4388252;rs4392675;rs200252534;rs145520315;rs147699420;rs77803982;rs79469094;rs75077625;rs74379173;rs78664536;rs549536839;rs570631944;rs78784808;rs78340649;rs201569786;rs75649455;rs78814720;rs77561644;rs76565008;rs74436342;rs1592807;rs1592810;rs1592811;rs7700458;rs6594365;rs6863893;rs140163909;rs146837743;rs7705919;rs201046346;rs202152005;rs199869120;rs111726541;rs373673987;rs113026830;rs148211606;rs201619508;rs201169921;rs146644914;rs116649742;rs79893293;rs57629433;rs60335509;rs58453996;rs59466242;rs60878839;rs11743452;rs1833567;rs1833568;rs1833569;rs7721272;rs7721427;rs7721730;rs7721870;rs7722100;rs7704359;rs1895200;rs1895201;rs77974856;rs60905342;rs11746207;rs11739672;rs11749718;rs78518991;rs76243602;rs76383235;rs11741980;rs35243960;rs116462975;rs139801813;rs113103584;rs74727230;rs75919325;rs79624714;rs2080858;rs138092354;rs148285468;rs114455395;rs74850689;rs10463599 | ENSG00000151422 | 0 |
| 4 | 5:108560001-108600000 | 5:108120001-108160000 | 5.71E-17 | HiC | GSE87112 | Mesenchymal_Stem_Cell | intra | rs35812497;rs75803484;rs75073283;rs76430100;rs75782477;rs77992445;rs61701247;rs17161655;rs115979287;rs17161659;rs151072743;rs17161665;rs6863688;5:108591279:C:T;5:108591279:A:T;rs1363213;rs1862201;rs1862202;rs1862203;rs5870359 | NA | 0 |
| 4 | 5:108560001-108600000 | 5:108160001-108200000 | 8.08E-22 | HiC | GSE87112 | Mesenchymal_Stem_Cell | intra | rs35812497;rs75803484;rs75073283;rs76430100;rs75782477;rs77992445;rs61701247;rs17161655;rs115979287;rs17161659;rs151072743;rs17161665;rs6863688;5:108591279:C:T;5:108591279:A:T;rs1363213;rs1862201;rs1862202;rs1862203;rs5870359 | NA | 0 |
| 4 | 5:108600001-108640000 | 5:108160001-108200000 | 6.69E-15 | HiC | GSE87112 | Mesenchymal_Stem_Cell | intra | rs11744353;rs79776200;rs76069623;rs76043709;rs78729571;rs36123121;rs11748450;rs80234158;rs78146542;rs185072862;rs137867205;rs189006725;rs4388251;rs4438924;rs4541698;rs4388252;rs4392675;rs200252534;rs145520315;rs147699420;rs77803982;rs79469094;rs75077625;rs74379173;rs78664536;rs549536839;rs570631944;rs78784808;rs78340649;rs201569786;rs75649455;rs78814720;rs77561644;rs76565008;rs74436342;rs1592807;rs1592810;rs1592811;rs7700458;rs6594365;rs6863893;rs140163909;rs146837743;rs7705919;rs201046346;rs202152005;rs199869120;rs111726541;rs373673987;rs113026830;rs148211606;rs201619508;rs201169921;rs146644914;rs116649742;rs79893293;rs57629433;rs60335509;rs58453996;rs59466242;rs60878839;rs11743452;rs1833567;rs1833568;rs1833569;rs7721272;rs7721427;rs7721730;rs7721870;rs7722100;rs7704359;rs1895200;rs1895201;rs77974856;rs60905342;rs11746207;rs11739672;rs11749718;rs78518991;rs76243602;rs76383235;rs11741980;rs35243960;rs116462975;rs139801813;rs113103584;rs74727230;rs75919325;rs79624714;rs2080858;rs138092354;rs148285468;rs114455395;rs74850689;rs10463599 | NA | 0 |
| 4 | 5:108560001-108600000 | 5:108200001-108240000 | 1.38E-46 | HiC | GSE87112 | Mesenchymal_Stem_Cell | intra | rs35812497;rs75803484;rs75073283;rs76430100;rs75782477;rs77992445;rs61701247;rs17161655;rs115979287;rs17161659;rs151072743;rs17161665;rs6863688;5:108591279:C:T;5:108591279:A:T;rs1363213;rs1862201;rs1862202;rs1862203;rs5870359 | NA | 0 |
| 4 | 5:108600001-108640000 | 5:108200001-108240000 | 2.42E-30 | HiC | GSE87112 | Mesenchymal_Stem_Cell | intra | rs11744353;rs79776200;rs76069623;rs76043709;rs78729571;rs36123121;rs11748450;rs80234158;rs78146542;rs185072862;rs137867205;rs189006725;rs4388251;rs4438924;rs4541698;rs4388252;rs4392675;rs200252534;rs145520315;rs147699420;rs77803982;rs79469094;rs75077625;rs74379173;rs78664536;rs549536839;rs570631944;rs78784808;rs78340649;rs201569786;rs75649455;rs78814720;rs77561644;rs76565008;rs74436342;rs1592807;rs1592810;rs1592811;rs7700458;rs6594365;rs6863893;rs140163909;rs146837743;rs7705919;rs201046346;rs202152005;rs199869120;rs111726541;rs373673987;rs113026830;rs148211606;rs201619508;rs201169921;rs146644914;rs116649742;rs79893293;rs57629433;rs60335509;rs58453996;rs59466242;rs60878839;rs11743452;rs1833567;rs1833568;rs1833569;rs7721272;rs7721427;rs7721730;rs7721870;rs7722100;rs7704359;rs1895200;rs1895201;rs77974856;rs60905342;rs11746207;rs11739672;rs11749718;rs78518991;rs76243602;rs76383235;rs11741980;rs35243960;rs116462975;rs139801813;rs113103584;rs74727230;rs75919325;rs79624714;rs2080858;rs138092354;rs148285468;rs114455395;rs74850689;rs10463599 | NA | 0 |
| 4 | 5:108560001-108600000 | 5:108240001-108280000 | 7.27E-22 | HiC | GSE87112 | Mesenchymal_Stem_Cell | intra | rs35812497;rs75803484;rs75073283;rs76430100;rs75782477;rs77992445;rs61701247;rs17161655;rs115979287;rs17161659;rs151072743;rs17161665;rs6863688;5:108591279:C:T;5:108591279:A:T;rs1363213;rs1862201;rs1862202;rs1862203;rs5870359 | NA | 0 |
| 4 | 5:108600001-108640000 | 5:108240001-108280000 | 7.24E-14 | HiC | GSE87112 | Mesenchymal_Stem_Cell | intra | rs11744353;rs79776200;rs76069623;rs76043709;rs78729571;rs36123121;rs11748450;rs80234158;rs78146542;rs185072862;rs137867205;rs189006725;rs4388251;rs4438924;rs4541698;rs4388252;rs4392675;rs200252534;rs145520315;rs147699420;rs77803982;rs79469094;rs75077625;rs74379173;rs78664536;rs549536839;rs570631944;rs78784808;rs78340649;rs201569786;rs75649455;rs78814720;rs77561644;rs76565008;rs74436342;rs1592807;rs1592810;rs1592811;rs7700458;rs6594365;rs6863893;rs140163909;rs146837743;rs7705919;rs201046346;rs202152005;rs199869120;rs111726541;rs373673987;rs113026830;rs148211606;rs201619508;rs201169921;rs146644914;rs116649742;rs79893293;rs57629433;rs60335509;rs58453996;rs59466242;rs60878839;rs11743452;rs1833567;rs1833568;rs1833569;rs7721272;rs7721427;rs7721730;rs7721870;rs7722100;rs7704359;rs1895200;rs1895201;rs77974856;rs60905342;rs11746207;rs11739672;rs11749718;rs78518991;rs76243602;rs76383235;rs11741980;rs35243960;rs116462975;rs139801813;rs113103584;rs74727230;rs75919325;rs79624714;rs2080858;rs138092354;rs148285468;rs114455395;rs74850689;rs10463599 | NA | 0 |
| 4 | 5:108560001-108600000 | 5:108280001-108320000 | 7.66E-11 | HiC | GSE87112 | Mesenchymal_Stem_Cell | intra | rs35812497;rs75803484;rs75073283;rs76430100;rs75782477;rs77992445;rs61701247;rs17161655;rs115979287;rs17161659;rs151072743;rs17161665;rs6863688;5:108591279:C:T;5:108591279:A:T;rs1363213;rs1862201;rs1862202;rs1862203;rs5870359 | NA | 0 |
| 4 | 5:108560001-108600000 | 5:108320001-108360000 | 8.16E-40 | HiC | GSE87112 | Mesenchymal_Stem_Cell | intra | rs35812497;rs75803484;rs75073283;rs76430100;rs75782477;rs77992445;rs61701247;rs17161655;rs115979287;rs17161659;rs151072743;rs17161665;rs6863688;5:108591279:C:T;5:108591279:A:T;rs1363213;rs1862201;rs1862202;rs1862203;rs5870359 | NA | 0 |
| 4 | 5:108600001-108640000 | 5:108320001-108360000 | 2.70E-21 | HiC | GSE87112 | Mesenchymal_Stem_Cell | intra | rs11744353;rs79776200;rs76069623;rs76043709;rs78729571;rs36123121;rs11748450;rs80234158;rs78146542;rs185072862;rs137867205;rs189006725;rs4388251;rs4438924;rs4541698;rs4388252;rs4392675;rs200252534;rs145520315;rs147699420;rs77803982;rs79469094;rs75077625;rs74379173;rs78664536;rs549536839;rs570631944;rs78784808;rs78340649;rs201569786;rs75649455;rs78814720;rs77561644;rs76565008;rs74436342;rs1592807;rs1592810;rs1592811;rs7700458;rs6594365;rs6863893;rs140163909;rs146837743;rs7705919;rs201046346;rs202152005;rs199869120;rs111726541;rs373673987;rs113026830;rs148211606;rs201619508;rs201169921;rs146644914;rs116649742;rs79893293;rs57629433;rs60335509;rs58453996;rs59466242;rs60878839;rs11743452;rs1833567;rs1833568;rs1833569;rs7721272;rs7721427;rs7721730;rs7721870;rs7722100;rs7704359;rs1895200;rs1895201;rs77974856;rs60905342;rs11746207;rs11739672;rs11749718;rs78518991;rs76243602;rs76383235;rs11741980;rs35243960;rs116462975;rs139801813;rs113103584;rs74727230;rs75919325;rs79624714;rs2080858;rs138092354;rs148285468;rs114455395;rs74850689;rs10463599 | NA | 0 |
| 4 | 5:108560001-108600000 | 5:108360001-108400000 | 2.08E-32 | HiC | GSE87112 | Mesenchymal_Stem_Cell | intra | rs35812497;rs75803484;rs75073283;rs76430100;rs75782477;rs77992445;rs61701247;rs17161655;rs115979287;rs17161659;rs151072743;rs17161665;rs6863688;5:108591279:C:T;5:108591279:A:T;rs1363213;rs1862201;rs1862202;rs1862203;rs5870359 | NA | 0 |
| 4 | 5:108600001-108640000 | 5:108360001-108400000 | 2.56E-22 | HiC | GSE87112 | Mesenchymal_Stem_Cell | intra | rs11744353;rs79776200;rs76069623;rs76043709;rs78729571;rs36123121;rs11748450;rs80234158;rs78146542;rs185072862;rs137867205;rs189006725;rs4388251;rs4438924;rs4541698;rs4388252;rs4392675;rs200252534;rs145520315;rs147699420;rs77803982;rs79469094;rs75077625;rs74379173;rs78664536;rs549536839;rs570631944;rs78784808;rs78340649;rs201569786;rs75649455;rs78814720;rs77561644;rs76565008;rs74436342;rs1592807;rs1592810;rs1592811;rs7700458;rs6594365;rs6863893;rs140163909;rs146837743;rs7705919;rs201046346;rs202152005;rs199869120;rs111726541;rs373673987;rs113026830;rs148211606;rs201619508;rs201169921;rs146644914;rs116649742;rs79893293;rs57629433;rs60335509;rs58453996;rs59466242;rs60878839;rs11743452;rs1833567;rs1833568;rs1833569;rs7721272;rs7721427;rs7721730;rs7721870;rs7722100;rs7704359;rs1895200;rs1895201;rs77974856;rs60905342;rs11746207;rs11739672;rs11749718;rs78518991;rs76243602;rs76383235;rs11741980;rs35243960;rs116462975;rs139801813;rs113103584;rs74727230;rs75919325;rs79624714;rs2080858;rs138092354;rs148285468;rs114455395;rs74850689;rs10463599 | NA | 0 |
| 4 | 5:108560001-108600000 | 5:108400001-108440000 | 4.50E-10 | HiC | GSE87112 | Mesenchymal_Stem_Cell | intra | rs35812497;rs75803484;rs75073283;rs76430100;rs75782477;rs77992445;rs61701247;rs17161655;rs115979287;rs17161659;rs151072743;rs17161665;rs6863688;5:108591279:C:T;5:108591279:A:T;rs1363213;rs1862201;rs1862202;rs1862203;rs5870359 | NA | 0 |
| 4 | 5:108560001-108600000 | 5:108480001-108520000 | 2.72E-57 | HiC | GSE87112 | Mesenchymal_Stem_Cell | intra | rs35812497;rs75803484;rs75073283;rs76430100;rs75782477;rs77992445;rs61701247;rs17161655;rs115979287;rs17161659;rs151072743;rs17161665;rs6863688;5:108591279:C:T;5:108591279:A:T;rs1363213;rs1862201;rs1862202;rs1862203;rs5870359 | NA | 0 |
| 4 | 5:108600001-108640000 | 5:108480001-108520000 | 1.13E-23 | HiC | GSE87112 | Mesenchymal_Stem_Cell | intra | rs11744353;rs79776200;rs76069623;rs76043709;rs78729571;rs36123121;rs11748450;rs80234158;rs78146542;rs185072862;rs137867205;rs189006725;rs4388251;rs4438924;rs4541698;rs4388252;rs4392675;rs200252534;rs145520315;rs147699420;rs77803982;rs79469094;rs75077625;rs74379173;rs78664536;rs549536839;rs570631944;rs78784808;rs78340649;rs201569786;rs75649455;rs78814720;rs77561644;rs76565008;rs74436342;rs1592807;rs1592810;rs1592811;rs7700458;rs6594365;rs6863893;rs140163909;rs146837743;rs7705919;rs201046346;rs202152005;rs199869120;rs111726541;rs373673987;rs113026830;rs148211606;rs201619508;rs201169921;rs146644914;rs116649742;rs79893293;rs57629433;rs60335509;rs58453996;rs59466242;rs60878839;rs11743452;rs1833567;rs1833568;rs1833569;rs7721272;rs7721427;rs7721730;rs7721870;rs7722100;rs7704359;rs1895200;rs1895201;rs77974856;rs60905342;rs11746207;rs11739672;rs11749718;rs78518991;rs76243602;rs76383235;rs11741980;rs35243960;rs116462975;rs139801813;rs113103584;rs74727230;rs75919325;rs79624714;rs2080858;rs138092354;rs148285468;rs114455395;rs74850689;rs10463599 | NA | 0 |
| 4 | 5:108560001-108600000 | 5:108520001-108560000 | 2.48E-48 | HiC | GSE87112 | Mesenchymal_Stem_Cell | intra | rs35812497;rs75803484;rs75073283;rs76430100;rs75782477;rs77992445;rs61701247;rs17161655;rs115979287;rs17161659;rs151072743;rs17161665;rs6863688;5:108591279:C:T;5:108591279:A:T;rs1363213;rs1862201;rs1862202;rs1862203;rs5870359 | NA | 0 |
| 4 | 5:108600001-108640000 | 5:108520001-108560000 | 3.20E-09 | HiC | GSE87112 | Mesenchymal_Stem_Cell | intra | rs11744353;rs79776200;rs76069623;rs76043709;rs78729571;rs36123121;rs11748450;rs80234158;rs78146542;rs185072862;rs137867205;rs189006725;rs4388251;rs4438924;rs4541698;rs4388252;rs4392675;rs200252534;rs145520315;rs147699420;rs77803982;rs79469094;rs75077625;rs74379173;rs78664536;rs549536839;rs570631944;rs78784808;rs78340649;rs201569786;rs75649455;rs78814720;rs77561644;rs76565008;rs74436342;rs1592807;rs1592810;rs1592811;rs7700458;rs6594365;rs6863893;rs140163909;rs146837743;rs7705919;rs201046346;rs202152005;rs199869120;rs111726541;rs373673987;rs113026830;rs148211606;rs201619508;rs201169921;rs146644914;rs116649742;rs79893293;rs57629433;rs60335509;rs58453996;rs59466242;rs60878839;rs11743452;rs1833567;rs1833568;rs1833569;rs7721272;rs7721427;rs7721730;rs7721870;rs7722100;rs7704359;rs1895200;rs1895201;rs77974856;rs60905342;rs11746207;rs11739672;rs11749718;rs78518991;rs76243602;rs76383235;rs11741980;rs35243960;rs116462975;rs139801813;rs113103584;rs74727230;rs75919325;rs79624714;rs2080858;rs138092354;rs148285468;rs114455395;rs74850689;rs10463599 | NA | 0 |
| 4 | 5:108600001-108640000 | 5:108560001-108600000 | 1.60E-13 | HiC | GSE87112 | Mesenchymal_Stem_Cell | intra | rs11744353;rs79776200;rs76069623;rs76043709;rs78729571;rs36123121;rs11748450;rs80234158;rs78146542;rs185072862;rs137867205;rs189006725;rs4388251;rs4438924;rs4541698;rs4388252;rs4392675;rs200252534;rs145520315;rs147699420;rs77803982;rs79469094;rs75077625;rs74379173;rs78664536;rs549536839;rs570631944;rs78784808;rs78340649;rs201569786;rs75649455;rs78814720;rs77561644;rs76565008;rs74436342;rs1592807;rs1592810;rs1592811;rs7700458;rs6594365;rs6863893;rs140163909;rs146837743;rs7705919;rs201046346;rs202152005;rs199869120;rs111726541;rs373673987;rs113026830;rs148211606;rs201619508;rs201169921;rs146644914;rs116649742;rs79893293;rs57629433;rs60335509;rs58453996;rs59466242;rs60878839;rs11743452;rs1833567;rs1833568;rs1833569;rs7721272;rs7721427;rs7721730;rs7721870;rs7722100;rs7704359;rs1895200;rs1895201;rs77974856;rs60905342;rs11746207;rs11739672;rs11749718;rs78518991;rs76243602;rs76383235;rs11741980;rs35243960;rs116462975;rs139801813;rs113103584;rs74727230;rs75919325;rs79624714;rs2080858;rs138092354;rs148285468;rs114455395;rs74850689;rs10463599 | NA | 0 |
| 5 | 11:2520001-2560000 | 11:2200001-2240000 | 6.57E-07 | HiC | GSE87112 | Mesenchymal_Stem_Cell | intra | rs111815403 | NA | 0 |
| 5 | 11:2520001-2560000 | 11:2240001-2280000 | 2.98E-30 | HiC | GSE87112 | Mesenchymal_Stem_Cell | intra | rs111815403 | NA | 0 |
| 5 | 11:2520001-2560000 | 11:2280001-2320000 | 3.58E-11 | HiC | GSE87112 | Mesenchymal_Stem_Cell | intra | rs111815403 | ENSG00000183734 | 1 |
| 6 | 12:80800001-80840000 | 12:77840001-77880000 | 3.85E-07 | HiC | GSE87112 | Mesenchymal_Stem_Cell | intra | rs117203215;rs77741796 | NA | 0 |
| 6 | 12:80800001-80840000 | 12:77880001-77920000 | 2.54E-08 | HiC | GSE87112 | Mesenchymal_Stem_Cell | intra | rs117203215;rs77741796 | NA | 0 |
| 6 | 12:80800001-80840000 | 12:77960001-78000000 | 1.44E-09 | HiC | GSE87112 | Mesenchymal_Stem_Cell | intra | rs117203215;rs77741796 | NA | 0 |
| 6 | 12:80800001-80840000 | 12:78080001-78120000 | 5.56E-10 | HiC | GSE87112 | Mesenchymal_Stem_Cell | intra | rs117203215;rs77741796 | NA | 0 |
| 6 | 12:80800001-80840000 | 12:78560001-78600000 | 2.16E-11 | HiC | GSE87112 | Mesenchymal_Stem_Cell | intra | rs117203215;rs77741796 | NA | 0 |
| 6 | 12:80800001-80840000 | 12:78600001-78640000 | 5.15E-15 | HiC | GSE87112 | Mesenchymal_Stem_Cell | intra | rs117203215;rs77741796 | NA | 0 |
| 6 | 12:80800001-80840000 | 12:78640001-78680000 | 4.13E-21 | HiC | GSE87112 | Mesenchymal_Stem_Cell | intra | rs117203215;rs77741796 | NA | 0 |
| 6 | 12:80800001-80840000 | 12:78680001-78720000 | 4.57E-12 | HiC | GSE87112 | Mesenchymal_Stem_Cell | intra | rs117203215;rs77741796 | NA | 0 |
| 6 | 12:80800001-80840000 | 12:78720001-78760000 | 3.51E-33 | HiC | GSE87112 | Mesenchymal_Stem_Cell | intra | rs117203215;rs77741796 | NA | 0 |
| 6 | 12:80840001-80880000 | 12:78720001-78760000 | 1.83E-11 | HiC | GSE87112 | Mesenchymal_Stem_Cell | intra | rs200589835 | NA | 0 |
| 6 | 12:80800001-80840000 | 12:78760001-78800000 | 1.13E-11 | HiC | GSE87112 | Mesenchymal_Stem_Cell | intra | rs117203215;rs77741796 | NA | 0 |
| 6 | 12:80800001-80840000 | 12:78840001-78880000 | 1.76E-08 | HiC | GSE87112 | Mesenchymal_Stem_Cell | intra | rs117203215;rs77741796 | NA | 0 |
| 6 | 12:80800001-80840000 | 12:78880001-78920000 | 8.83E-08 | HiC | GSE87112 | Mesenchymal_Stem_Cell | intra | rs117203215;rs77741796 | NA | 0 |
| 6 | 12:80800001-80840000 | 12:78920001-78960000 | 1.18E-09 | HiC | GSE87112 | Mesenchymal_Stem_Cell | intra | rs117203215;rs77741796 | NA | 0 |
| 6 | 12:80800001-80840000 | 12:78960001-79000000 | 1.46E-16 | HiC | GSE87112 | Mesenchymal_Stem_Cell | intra | rs117203215;rs77741796 | NA | 0 |
| 6 | 12:80800001-80840000 | 12:79040001-79080000 | 1.40E-11 | HiC | GSE87112 | Mesenchymal_Stem_Cell | intra | rs117203215;rs77741796 | NA | 0 |
| 6 | 12:80800001-80840000 | 12:79160001-79200000 | 4.71E-07 | HiC | GSE87112 | Mesenchymal_Stem_Cell | intra | rs117203215;rs77741796 | NA | 0 |
| 6 | 12:80800001-80840000 | 12:79200001-79240000 | 1.48E-14 | HiC | GSE87112 | Mesenchymal_Stem_Cell | intra | rs117203215;rs77741796 | NA | 0 |
| 6 | 12:80800001-80840000 | 12:79240001-79280000 | 1.17E-17 | HiC | GSE87112 | Mesenchymal_Stem_Cell | intra | rs117203215;rs77741796 | ENSG00000067715 | 0 |
| 6 | 12:80800001-80840000 | 12:79320001-79360000 | 4.53E-24 | HiC | GSE87112 | Mesenchymal_Stem_Cell | intra | rs117203215;rs77741796 | NA | 0 |
| 6 | 12:80840001-80880000 | 12:79320001-79360000 | 2.54E-08 | HiC | GSE87112 | Mesenchymal_Stem_Cell | intra | rs200589835 | NA | 0 |
| 6 | 12:80800001-80840000 | 12:79480001-79520000 | 3.69E-14 | HiC | GSE87112 | Mesenchymal_Stem_Cell | intra | rs117203215;rs77741796 | NA | 0 |
| 6 | 12:80800001-80840000 | 12:79600001-79640000 | 1.52E-07 | HiC | GSE87112 | Mesenchymal_Stem_Cell | intra | rs117203215;rs77741796 | NA | 0 |
| 6 | 12:80800001-80840000 | 12:79640001-79680000 | 6.18E-39 | HiC | GSE87112 | Mesenchymal_Stem_Cell | intra | rs117203215;rs77741796 | NA | 0 |
| 6 | 12:80840001-80880000 | 12:79640001-79680000 | 2.34E-08 | HiC | GSE87112 | Mesenchymal_Stem_Cell | intra | rs200589835 | NA | 0 |
| 6 | 12:80800001-80840000 | 12:79680001-79720000 | 1.91E-18 | HiC | GSE87112 | Mesenchymal_Stem_Cell | intra | rs117203215;rs77741796 | NA | 0 |
| 6 | 12:80800001-80840000 | 12:79720001-79760000 | 2.87E-46 | HiC | GSE87112 | Mesenchymal_Stem_Cell | intra | rs117203215;rs77741796 | NA | 0 |
| 6 | 12:80640001-80680000 | 12:79760001-79800000 | 4.81E-28 | HiC | GSE87112 | Mesenchymal_Stem_Cell | intra | rs201272756;rs202070984 | NA | 0 |
| 6 | 12:80800001-80840000 | 12:79760001-79800000 | 6.25E-54 | HiC | GSE87112 | Mesenchymal_Stem_Cell | intra | rs117203215;rs77741796 | NA | 0 |
| 6 | 12:80840001-80880000 | 12:79760001-79800000 | 2.41E-09 | HiC | GSE87112 | Mesenchymal_Stem_Cell | intra | rs200589835 | NA | 0 |
| 6 | 12:80640001-80680000 | 12:79800001-79840000 | 1.20E-22 | HiC | GSE87112 | Mesenchymal_Stem_Cell | intra | rs201272756;rs202070984 | NA | 0 |
| 6 | 12:80800001-80840000 | 12:79800001-79840000 | 5.75E-41 | HiC | GSE87112 | Mesenchymal_Stem_Cell | intra | rs117203215;rs77741796 | NA | 0 |
| 6 | 12:80840001-80880000 | 12:79800001-79840000 | 7.01E-08 | HiC | GSE87112 | Mesenchymal_Stem_Cell | intra | rs200589835 | NA | 0 |
| 6 | 12:80640001-80680000 | 12:79920001-79960000 | 1.38E-22 | HiC | GSE87112 | Mesenchymal_Stem_Cell | intra | rs201272756;rs202070984 | NA | 0 |
| 6 | 12:80800001-80840000 | 12:79920001-79960000 | 1.53E-36 | HiC | GSE87112 | Mesenchymal_Stem_Cell | intra | rs117203215;rs77741796 | NA | 0 |
| 6 | 12:80800001-80840000 | 12:80040001-80080000 | 1.88E-13 | HiC | GSE87112 | Mesenchymal_Stem_Cell | intra | rs117203215;rs77741796 | NA | 0 |
| 6 | 12:80800001-80840000 | 12:80080001-80120000 | 1.64E-11 | HiC | GSE87112 | Mesenchymal_Stem_Cell | intra | rs117203215;rs77741796 | ENSG00000177425 | 0 |
| 6 | 12:80640001-80680000 | 12:80160001-80200000 | 4.53E-11 | HiC | GSE87112 | Mesenchymal_Stem_Cell | intra | rs201272756;rs202070984 | ENSG00000269531 | 0 |
| 6 | 12:80800001-80840000 | 12:80160001-80200000 | 3.34E-08 | HiC | GSE87112 | Mesenchymal_Stem_Cell | intra | rs117203215;rs77741796 | ENSG00000269531 | 0 |
| 6 | 12:80800001-80840000 | 12:80360001-80400000 | 3.02E-07 | HiC | GSE87112 | Mesenchymal_Stem_Cell | intra | rs117203215;rs77741796 | NA | 0 |
| 6 | 12:80640001-80680000 | 12:80560001-80600000 | 8.87E-09 | HiC | GSE87112 | Mesenchymal_Stem_Cell | intra | rs201272756;rs202070984 | NA | 0 |
| 6 | 12:80800001-80840000 | 12:80560001-80600000 | 9.51E-18 | HiC | GSE87112 | Mesenchymal_Stem_Cell | intra | rs117203215;rs77741796 | NA | 0 |
| 6 | 12:80800001-80840000 | 12:80600001-80640000 | 5.45E-10 | HiC | GSE87112 | Mesenchymal_Stem_Cell | intra | rs117203215;rs77741796 | ENSG00000165899 | 0 |
| 6 | 12:80800001-80840000 | 12:80680001-80720000 | 6.28E-40 | HiC | GSE87112 | Mesenchymal_Stem_Cell | intra | rs117203215;rs77741796 | NA | 0 |
| 6 | 12:80800001-80840000 | 12:80720001-80760000 | 1.23E-33 | HiC | GSE87112 | Mesenchymal_Stem_Cell | intra | rs117203215;rs77741796 | NA | 0 |
| 6 | 12:80800001-80840000 | 12:80760001-80800000 | 1.79E-09 | HiC | GSE87112 | Mesenchymal_Stem_Cell | intra | rs117203215;rs77741796 | ENSG00000139304 | 0 |
| 7 | 12:127760001-127800000 | 12:125560001-125600000 | 1.05E-13 | HiC | GSE87112 | Mesenchymal_Stem_Cell | intra | rs1810088;rs367839627;rs146043604;rs143439170;rs10773382;rs10847307;rs35154640;rs1552386;rs7314186;rs1979065;rs5801722;rs10744302;12:127775513:A:C;rs10734938;rs35024927;rs10773387;rs978813;rs2348321;rs10847321;rs10847323;rs6489163 | NA | 0 |
| 7 | 12:127760001-127800000 | 12:125640001-125680000 | 1.36E-16 | HiC | GSE87112 | Mesenchymal_Stem_Cell | intra | rs1810088;rs367839627;rs146043604;rs143439170;rs10773382;rs10847307;rs35154640;rs1552386;rs7314186;rs1979065;rs5801722;rs10744302;12:127775513:A:C;rs10734938;rs35024927;rs10773387;rs978813;rs2348321;rs10847321;rs10847323;rs6489163 | ENSG00000139364 | 0 |
| 7 | 12:127760001-127800000 | 12:125680001-125720000 | 4.96E-17 | HiC | GSE87112 | Mesenchymal_Stem_Cell | intra | rs1810088;rs367839627;rs146043604;rs143439170;rs10773382;rs10847307;rs35154640;rs1552386;rs7314186;rs1979065;rs5801722;rs10744302;12:127775513:A:C;rs10734938;rs35024927;rs10773387;rs978813;rs2348321;rs10847321;rs10847323;rs6489163 | NA | 0 |
| 7 | 12:127760001-127800000 | 12:125720001-125760000 | 1.94E-15 | HiC | GSE87112 | Mesenchymal_Stem_Cell | intra | rs1810088;rs367839627;rs146043604;rs143439170;rs10773382;rs10847307;rs35154640;rs1552386;rs7314186;rs1979065;rs5801722;rs10744302;12:127775513:A:C;rs10734938;rs35024927;rs10773387;rs978813;rs2348321;rs10847321;rs10847323;rs6489163 | NA | 0 |
| 7 | 12:127760001-127800000 | 12:125760001-125800000 | 9.09E-43 | HiC | GSE87112 | Mesenchymal_Stem_Cell | intra | rs1810088;rs367839627;rs146043604;rs143439170;rs10773382;rs10847307;rs35154640;rs1552386;rs7314186;rs1979065;rs5801722;rs10744302;12:127775513:A:C;rs10734938;rs35024927;rs10773387;rs978813;rs2348321;rs10847321;rs10847323;rs6489163 | NA | 0 |
| 7 | 12:127760001-127800000 | 12:125800001-125840000 | 1.13E-28 | HiC | GSE87112 | Mesenchymal_Stem_Cell | intra | rs1810088;rs367839627;rs146043604;rs143439170;rs10773382;rs10847307;rs35154640;rs1552386;rs7314186;rs1979065;rs5801722;rs10744302;12:127775513:A:C;rs10734938;rs35024927;rs10773387;rs978813;rs2348321;rs10847321;rs10847323;rs6489163 | NA | 0 |
| 7 | 12:127760001-127800000 | 12:125840001-125880000 | 1.03E-20 | HiC | GSE87112 | Mesenchymal_Stem_Cell | intra | rs1810088;rs367839627;rs146043604;rs143439170;rs10773382;rs10847307;rs35154640;rs1552386;rs7314186;rs1979065;rs5801722;rs10744302;12:127775513:A:C;rs10734938;rs35024927;rs10773387;rs978813;rs2348321;rs10847321;rs10847323;rs6489163 | NA | 0 |
| 7 | 12:127760001-127800000 | 12:125880001-125920000 | 9.23E-11 | HiC | GSE87112 | Mesenchymal_Stem_Cell | intra | rs1810088;rs367839627;rs146043604;rs143439170;rs10773382;rs10847307;rs35154640;rs1552386;rs7314186;rs1979065;rs5801722;rs10744302;12:127775513:A:C;rs10734938;rs35024927;rs10773387;rs978813;rs2348321;rs10847321;rs10847323;rs6489163 | NA | 0 |
| 7 | 12:127760001-127800000 | 12:125920001-125960000 | 3.00E-24 | HiC | GSE87112 | Mesenchymal_Stem_Cell | intra | rs1810088;rs367839627;rs146043604;rs143439170;rs10773382;rs10847307;rs35154640;rs1552386;rs7314186;rs1979065;rs5801722;rs10744302;12:127775513:A:C;rs10734938;rs35024927;rs10773387;rs978813;rs2348321;rs10847321;rs10847323;rs6489163 | NA | 0 |
| 7 | 12:127760001-127800000 | 12:125960001-126000000 | 1.39E-40 | HiC | GSE87112 | Mesenchymal_Stem_Cell | intra | rs1810088;rs367839627;rs146043604;rs143439170;rs10773382;rs10847307;rs35154640;rs1552386;rs7314186;rs1979065;rs5801722;rs10744302;12:127775513:A:C;rs10734938;rs35024927;rs10773387;rs978813;rs2348321;rs10847321;rs10847323;rs6489163 | NA | 0 |
| 7 | 12:127760001-127800000 | 12:126000001-126040000 | 4.54E-17 | HiC | GSE87112 | Mesenchymal_Stem_Cell | intra | rs1810088;rs367839627;rs146043604;rs143439170;rs10773382;rs10847307;rs35154640;rs1552386;rs7314186;rs1979065;rs5801722;rs10744302;12:127775513:A:C;rs10734938;rs35024927;rs10773387;rs978813;rs2348321;rs10847321;rs10847323;rs6489163 | NA | 0 |
| 7 | 12:127760001-127800000 | 12:126040001-126080000 | 2.43E-26 | HiC | GSE87112 | Mesenchymal_Stem_Cell | intra | rs1810088;rs367839627;rs146043604;rs143439170;rs10773382;rs10847307;rs35154640;rs1552386;rs7314186;rs1979065;rs5801722;rs10744302;12:127775513:A:C;rs10734938;rs35024927;rs10773387;rs978813;rs2348321;rs10847321;rs10847323;rs6489163 | NA | 0 |
| 7 | 12:127760001-127800000 | 12:126120001-126160000 | 3.90E-34 | HiC | GSE87112 | Mesenchymal_Stem_Cell | intra | rs1810088;rs367839627;rs146043604;rs143439170;rs10773382;rs10847307;rs35154640;rs1552386;rs7314186;rs1979065;rs5801722;rs10744302;12:127775513:A:C;rs10734938;rs35024927;rs10773387;rs978813;rs2348321;rs10847321;rs10847323;rs6489163 | NA | 0 |
| 7 | 12:127760001-127800000 | 12:126160001-126200000 | 1.48E-14 | HiC | GSE87112 | Mesenchymal_Stem_Cell | intra | rs1810088;rs367839627;rs146043604;rs143439170;rs10773382;rs10847307;rs35154640;rs1552386;rs7314186;rs1979065;rs5801722;rs10744302;12:127775513:A:C;rs10734938;rs35024927;rs10773387;rs978813;rs2348321;rs10847321;rs10847323;rs6489163 | NA | 0 |
| 7 | 12:127760001-127800000 | 12:126200001-126240000 | 3.94E-41 | HiC | GSE87112 | Mesenchymal_Stem_Cell | intra | rs1810088;rs367839627;rs146043604;rs143439170;rs10773382;rs10847307;rs35154640;rs1552386;rs7314186;rs1979065;rs5801722;rs10744302;12:127775513:A:C;rs10734938;rs35024927;rs10773387;rs978813;rs2348321;rs10847321;rs10847323;rs6489163 | NA | 0 |
| 7 | 12:127760001-127800000 | 12:126240001-126280000 | 6.68E-19 | HiC | GSE87112 | Mesenchymal_Stem_Cell | intra | rs1810088;rs367839627;rs146043604;rs143439170;rs10773382;rs10847307;rs35154640;rs1552386;rs7314186;rs1979065;rs5801722;rs10744302;12:127775513:A:C;rs10734938;rs35024927;rs10773387;rs978813;rs2348321;rs10847321;rs10847323;rs6489163 | NA | 0 |
| 7 | 12:127760001-127800000 | 12:126280001-126320000 | 3.44E-52 | HiC | GSE87112 | Mesenchymal_Stem_Cell | intra | rs1810088;rs367839627;rs146043604;rs143439170;rs10773382;rs10847307;rs35154640;rs1552386;rs7314186;rs1979065;rs5801722;rs10744302;12:127775513:A:C;rs10734938;rs35024927;rs10773387;rs978813;rs2348321;rs10847321;rs10847323;rs6489163 | NA | 0 |
| 7 | 12:127760001-127800000 | 12:126320001-126360000 | 1.03E-15 | HiC | GSE87112 | Mesenchymal_Stem_Cell | intra | rs1810088;rs367839627;rs146043604;rs143439170;rs10773382;rs10847307;rs35154640;rs1552386;rs7314186;rs1979065;rs5801722;rs10744302;12:127775513:A:C;rs10734938;rs35024927;rs10773387;rs978813;rs2348321;rs10847321;rs10847323;rs6489163 | NA | 0 |
| 7 | 12:127760001-127800000 | 12:126360001-126400000 | 1.11E-21 | HiC | GSE87112 | Mesenchymal_Stem_Cell | intra | rs1810088;rs367839627;rs146043604;rs143439170;rs10773382;rs10847307;rs35154640;rs1552386;rs7314186;rs1979065;rs5801722;rs10744302;12:127775513:A:C;rs10734938;rs35024927;rs10773387;rs978813;rs2348321;rs10847321;rs10847323;rs6489163 | NA | 0 |
| 7 | 12:127760001-127800000 | 12:126400001-126440000 | 1.07E-11 | HiC | GSE87112 | Mesenchymal_Stem_Cell | intra | rs1810088;rs367839627;rs146043604;rs143439170;rs10773382;rs10847307;rs35154640;rs1552386;rs7314186;rs1979065;rs5801722;rs10744302;12:127775513:A:C;rs10734938;rs35024927;rs10773387;rs978813;rs2348321;rs10847321;rs10847323;rs6489163 | NA | 0 |
| 7 | 12:127760001-127800000 | 12:126440001-126480000 | 3.00E-15 | HiC | GSE87112 | Mesenchymal_Stem_Cell | intra | rs1810088;rs367839627;rs146043604;rs143439170;rs10773382;rs10847307;rs35154640;rs1552386;rs7314186;rs1979065;rs5801722;rs10744302;12:127775513:A:C;rs10734938;rs35024927;rs10773387;rs978813;rs2348321;rs10847321;rs10847323;rs6489163 | NA | 0 |
| 7 | 12:127760001-127800000 | 12:126480001-126520000 | 1.27E-22 | HiC | GSE87112 | Mesenchymal_Stem_Cell | intra | rs1810088;rs367839627;rs146043604;rs143439170;rs10773382;rs10847307;rs35154640;rs1552386;rs7314186;rs1979065;rs5801722;rs10744302;12:127775513:A:C;rs10734938;rs35024927;rs10773387;rs978813;rs2348321;rs10847321;rs10847323;rs6489163 | NA | 0 |
| 7 | 12:127760001-127800000 | 12:126520001-126560000 | 3.99E-34 | HiC | GSE87112 | Mesenchymal_Stem_Cell | intra | rs1810088;rs367839627;rs146043604;rs143439170;rs10773382;rs10847307;rs35154640;rs1552386;rs7314186;rs1979065;rs5801722;rs10744302;12:127775513:A:C;rs10734938;rs35024927;rs10773387;rs978813;rs2348321;rs10847321;rs10847323;rs6489163 | NA | 0 |
| 7 | 12:127760001-127800000 | 12:126560001-126600000 | 9.93E-22 | HiC | GSE87112 | Mesenchymal_Stem_Cell | intra | rs1810088;rs367839627;rs146043604;rs143439170;rs10773382;rs10847307;rs35154640;rs1552386;rs7314186;rs1979065;rs5801722;rs10744302;12:127775513:A:C;rs10734938;rs35024927;rs10773387;rs978813;rs2348321;rs10847321;rs10847323;rs6489163 | NA | 0 |
| 7 | 12:127760001-127800000 | 12:126640001-126680000 | 7.69E-22 | HiC | GSE87112 | Mesenchymal_Stem_Cell | intra | rs1810088;rs367839627;rs146043604;rs143439170;rs10773382;rs10847307;rs35154640;rs1552386;rs7314186;rs1979065;rs5801722;rs10744302;12:127775513:A:C;rs10734938;rs35024927;rs10773387;rs978813;rs2348321;rs10847321;rs10847323;rs6489163 | NA | 0 |
| 7 | 12:127760001-127800000 | 12:126680001-126720000 | 1.26E-42 | HiC | GSE87112 | Mesenchymal_Stem_Cell | intra | rs1810088;rs367839627;rs146043604;rs143439170;rs10773382;rs10847307;rs35154640;rs1552386;rs7314186;rs1979065;rs5801722;rs10744302;12:127775513:A:C;rs10734938;rs35024927;rs10773387;rs978813;rs2348321;rs10847321;rs10847323;rs6489163 | NA | 0 |
| 7 | 12:127760001-127800000 | 12:126720001-126760000 | 2.11E-26 | HiC | GSE87112 | Mesenchymal_Stem_Cell | intra | rs1810088;rs367839627;rs146043604;rs143439170;rs10773382;rs10847307;rs35154640;rs1552386;rs7314186;rs1979065;rs5801722;rs10744302;12:127775513:A:C;rs10734938;rs35024927;rs10773387;rs978813;rs2348321;rs10847321;rs10847323;rs6489163 | NA | 0 |
| 7 | 12:127760001-127800000 | 12:126760001-126800000 | 3.03E-07 | HiC | GSE87112 | Mesenchymal_Stem_Cell | intra | rs1810088;rs367839627;rs146043604;rs143439170;rs10773382;rs10847307;rs35154640;rs1552386;rs7314186;rs1979065;rs5801722;rs10744302;12:127775513:A:C;rs10734938;rs35024927;rs10773387;rs978813;rs2348321;rs10847321;rs10847323;rs6489163 | NA | 0 |
| 7 | 12:127760001-127800000 | 12:126800001-126840000 | 9.99E-40 | HiC | GSE87112 | Mesenchymal_Stem_Cell | intra | rs1810088;rs367839627;rs146043604;rs143439170;rs10773382;rs10847307;rs35154640;rs1552386;rs7314186;rs1979065;rs5801722;rs10744302;12:127775513:A:C;rs10734938;rs35024927;rs10773387;rs978813;rs2348321;rs10847321;rs10847323;rs6489163 | NA | 0 |
| 7 | 12:127760001-127800000 | 12:126840001-126880000 | 5.82E-10 | HiC | GSE87112 | Mesenchymal_Stem_Cell | intra | rs1810088;rs367839627;rs146043604;rs143439170;rs10773382;rs10847307;rs35154640;rs1552386;rs7314186;rs1979065;rs5801722;rs10744302;12:127775513:A:C;rs10734938;rs35024927;rs10773387;rs978813;rs2348321;rs10847321;rs10847323;rs6489163 | NA | 0 |
| 7 | 12:127760001-127800000 | 12:126880001-126920000 | 6.78E-21 | HiC | GSE87112 | Mesenchymal_Stem_Cell | intra | rs1810088;rs367839627;rs146043604;rs143439170;rs10773382;rs10847307;rs35154640;rs1552386;rs7314186;rs1979065;rs5801722;rs10744302;12:127775513:A:C;rs10734938;rs35024927;rs10773387;rs978813;rs2348321;rs10847321;rs10847323;rs6489163 | NA | 0 |
| 7 | 12:127760001-127800000 | 12:126920001-126960000 | 4.25E-09 | HiC | GSE87112 | Mesenchymal_Stem_Cell | intra | rs1810088;rs367839627;rs146043604;rs143439170;rs10773382;rs10847307;rs35154640;rs1552386;rs7314186;rs1979065;rs5801722;rs10744302;12:127775513:A:C;rs10734938;rs35024927;rs10773387;rs978813;rs2348321;rs10847321;rs10847323;rs6489163 | NA | 0 |
| 7 | 12:127760001-127800000 | 12:126960001-127000000 | 2.88E-23 | HiC | GSE87112 | Mesenchymal_Stem_Cell | intra | rs1810088;rs367839627;rs146043604;rs143439170;rs10773382;rs10847307;rs35154640;rs1552386;rs7314186;rs1979065;rs5801722;rs10744302;12:127775513:A:C;rs10734938;rs35024927;rs10773387;rs978813;rs2348321;rs10847321;rs10847323;rs6489163 | NA | 0 |
| 7 | 12:127760001-127800000 | 12:127000001-127040000 | 3.70E-15 | HiC | GSE87112 | Mesenchymal_Stem_Cell | intra | rs1810088;rs367839627;rs146043604;rs143439170;rs10773382;rs10847307;rs35154640;rs1552386;rs7314186;rs1979065;rs5801722;rs10744302;12:127775513:A:C;rs10734938;rs35024927;rs10773387;rs978813;rs2348321;rs10847321;rs10847323;rs6489163 | NA | 0 |
| 7 | 12:127760001-127800000 | 12:127040001-127080000 | 5.74E-09 | HiC | GSE87112 | Mesenchymal_Stem_Cell | intra | rs1810088;rs367839627;rs146043604;rs143439170;rs10773382;rs10847307;rs35154640;rs1552386;rs7314186;rs1979065;rs5801722;rs10744302;12:127775513:A:C;rs10734938;rs35024927;rs10773387;rs978813;rs2348321;rs10847321;rs10847323;rs6489163 | NA | 0 |
| 7 | 12:127760001-127800000 | 12:127080001-127120000 | 3.81E-36 | HiC | GSE87112 | Mesenchymal_Stem_Cell | intra | rs1810088;rs367839627;rs146043604;rs143439170;rs10773382;rs10847307;rs35154640;rs1552386;rs7314186;rs1979065;rs5801722;rs10744302;12:127775513:A:C;rs10734938;rs35024927;rs10773387;rs978813;rs2348321;rs10847321;rs10847323;rs6489163 | NA | 0 |
| 7 | 12:127760001-127800000 | 12:127120001-127160000 | 6.87E-08 | HiC | GSE87112 | Mesenchymal_Stem_Cell | intra | rs1810088;rs367839627;rs146043604;rs143439170;rs10773382;rs10847307;rs35154640;rs1552386;rs7314186;rs1979065;rs5801722;rs10744302;12:127775513:A:C;rs10734938;rs35024927;rs10773387;rs978813;rs2348321;rs10847321;rs10847323;rs6489163 | NA | 0 |
| 7 | 12:127760001-127800000 | 12:127160001-127200000 | 3.28E-10 | HiC | GSE87112 | Mesenchymal_Stem_Cell | intra | rs1810088;rs367839627;rs146043604;rs143439170;rs10773382;rs10847307;rs35154640;rs1552386;rs7314186;rs1979065;rs5801722;rs10744302;12:127775513:A:C;rs10734938;rs35024927;rs10773387;rs978813;rs2348321;rs10847321;rs10847323;rs6489163 | NA | 0 |
| 7 | 12:127760001-127800000 | 12:127200001-127240000 | 1.36E-44 | HiC | GSE87112 | Mesenchymal_Stem_Cell | intra | rs1810088;rs367839627;rs146043604;rs143439170;rs10773382;rs10847307;rs35154640;rs1552386;rs7314186;rs1979065;rs5801722;rs10744302;12:127775513:A:C;rs10734938;rs35024927;rs10773387;rs978813;rs2348321;rs10847321;rs10847323;rs6489163 | NA | 0 |
| 7 | 12:127760001-127800000 | 12:127240001-127280000 | 9.93E-50 | HiC | GSE87112 | Mesenchymal_Stem_Cell | intra | rs1810088;rs367839627;rs146043604;rs143439170;rs10773382;rs10847307;rs35154640;rs1552386;rs7314186;rs1979065;rs5801722;rs10744302;12:127775513:A:C;rs10734938;rs35024927;rs10773387;rs978813;rs2348321;rs10847321;rs10847323;rs6489163 | NA | 0 |
| 7 | 12:127760001-127800000 | 12:127280001-127320000 | 4.33E-24 | HiC | GSE87112 | Mesenchymal_Stem_Cell | intra | rs1810088;rs367839627;rs146043604;rs143439170;rs10773382;rs10847307;rs35154640;rs1552386;rs7314186;rs1979065;rs5801722;rs10744302;12:127775513:A:C;rs10734938;rs35024927;rs10773387;rs978813;rs2348321;rs10847321;rs10847323;rs6489163 | NA | 0 |
| 7 | 12:127760001-127800000 | 12:127320001-127360000 | 6.17E-19 | HiC | GSE87112 | Mesenchymal_Stem_Cell | intra | rs1810088;rs367839627;rs146043604;rs143439170;rs10773382;rs10847307;rs35154640;rs1552386;rs7314186;rs1979065;rs5801722;rs10744302;12:127775513:A:C;rs10734938;rs35024927;rs10773387;rs978813;rs2348321;rs10847321;rs10847323;rs6489163 | NA | 0 |
| 7 | 12:127760001-127800000 | 12:127360001-127400000 | 7.09E-32 | HiC | GSE87112 | Mesenchymal_Stem_Cell | intra | rs1810088;rs367839627;rs146043604;rs143439170;rs10773382;rs10847307;rs35154640;rs1552386;rs7314186;rs1979065;rs5801722;rs10744302;12:127775513:A:C;rs10734938;rs35024927;rs10773387;rs978813;rs2348321;rs10847321;rs10847323;rs6489163 | NA | 0 |
| 7 | 12:127760001-127800000 | 12:127400001-127440000 | 1.46E-07 | HiC | GSE87112 | Mesenchymal_Stem_Cell | intra | rs1810088;rs367839627;rs146043604;rs143439170;rs10773382;rs10847307;rs35154640;rs1552386;rs7314186;rs1979065;rs5801722;rs10744302;12:127775513:A:C;rs10734938;rs35024927;rs10773387;rs978813;rs2348321;rs10847321;rs10847323;rs6489163 | NA | 0 |
| 7 | 12:127760001-127800000 | 12:127440001-127480000 | 1.29E-19 | HiC | GSE87112 | Mesenchymal_Stem_Cell | intra | rs1810088;rs367839627;rs146043604;rs143439170;rs10773382;rs10847307;rs35154640;rs1552386;rs7314186;rs1979065;rs5801722;rs10744302;12:127775513:A:C;rs10734938;rs35024927;rs10773387;rs978813;rs2348321;rs10847321;rs10847323;rs6489163 | NA | 0 |
| 7 | 12:127760001-127800000 | 12:127480001-127520000 | 1.74E-20 | HiC | GSE87112 | Mesenchymal_Stem_Cell | intra | rs1810088;rs367839627;rs146043604;rs143439170;rs10773382;rs10847307;rs35154640;rs1552386;rs7314186;rs1979065;rs5801722;rs10744302;12:127775513:A:C;rs10734938;rs35024927;rs10773387;rs978813;rs2348321;rs10847321;rs10847323;rs6489163 | NA | 0 |
| 7 | 12:127760001-127800000 | 12:127560001-127600000 | 2.63E-26 | HiC | GSE87112 | Mesenchymal_Stem_Cell | intra | rs1810088;rs367839627;rs146043604;rs143439170;rs10773382;rs10847307;rs35154640;rs1552386;rs7314186;rs1979065;rs5801722;rs10744302;12:127775513:A:C;rs10734938;rs35024927;rs10773387;rs978813;rs2348321;rs10847321;rs10847323;rs6489163 | NA | 0 |
| 7 | 12:127760001-127800000 | 12:127600001-127640000 | 4.59E-16 | HiC | GSE87112 | Mesenchymal_Stem_Cell | intra | rs1810088;rs367839627;rs146043604;rs143439170;rs10773382;rs10847307;rs35154640;rs1552386;rs7314186;rs1979065;rs5801722;rs10744302;12:127775513:A:C;rs10734938;rs35024927;rs10773387;rs978813;rs2348321;rs10847321;rs10847323;rs6489163 | NA | 0 |
| 7 | 12:127760001-127800000 | 12:127640001-127680000 | 4.37E-07 | HiC | GSE87112 | Mesenchymal_Stem_Cell | intra | rs1810088;rs367839627;rs146043604;rs143439170;rs10773382;rs10847307;rs35154640;rs1552386;rs7314186;rs1979065;rs5801722;rs10744302;12:127775513:A:C;rs10734938;rs35024927;rs10773387;rs978813;rs2348321;rs10847321;rs10847323;rs6489163 | NA | 0 |
| 7 | 12:127760001-127800000 | 12:127680001-127720000 | 5.36E-08 | HiC | GSE87112 | Mesenchymal_Stem_Cell | intra | rs1810088;rs367839627;rs146043604;rs143439170;rs10773382;rs10847307;rs35154640;rs1552386;rs7314186;rs1979065;rs5801722;rs10744302;12:127775513:A:C;rs10734938;rs35024927;rs10773387;rs978813;rs2348321;rs10847321;rs10847323;rs6489163 | NA | 0 |
| 7 | 12:127760001-127800000 | 12:127720001-127760000 | 2.67E-37 | HiC | GSE87112 | Mesenchymal_Stem_Cell | intra | rs1810088;rs367839627;rs146043604;rs143439170;rs10773382;rs10847307;rs35154640;rs1552386;rs7314186;rs1979065;rs5801722;rs10744302;12:127775513:A:C;rs10734938;rs35024927;rs10773387;rs978813;rs2348321;rs10847321;rs10847323;rs6489163 | NA | 0 |
| 1 | 3:37520001-37560000 | 3:37720001-37760000 | 1.01E-10 | HiC | GSE87112 | Mesendoderm | intra | rs113897538;rs2507941;rs17814364;rs112038297;rs78544469;rs77422813;rs17228684;rs928799;rs75425697;rs5848026;rs17814903;rs12054441 | NA | 0 |
| 1 | 3:37520001-37560000 | 3:37760001-37800000 | 1.40E-20 | HiC | GSE87112 | Mesendoderm | intra | rs113897538;rs2507941;rs17814364;rs112038297;rs78544469;rs77422813;rs17228684;rs928799;rs75425697;rs5848026;rs17814903;rs12054441 | NA | 0 |
| 1 | 3:37520001-37560000 | 3:37800001-37840000 | 4.22E-19 | HiC | GSE87112 | Mesendoderm | intra | rs113897538;rs2507941;rs17814364;rs112038297;rs78544469;rs77422813;rs17228684;rs928799;rs75425697;rs5848026;rs17814903;rs12054441 | NA | 0 |
| 1 | 3:37560001-37600000 | 3:37640001-37680000 | 2.24E-10 | HiC | GSE87112 | Mesendoderm | intra | rs74985154;rs3733138;rs3733140;rs75619156;rs17229924;rs2162356;rs74786716;rs77820597;rs112585731;rs915631;rs79149273;rs17230261;rs199607377;rs2162355;rs76952775;rs75469174;rs78210776;rs77162103;rs75217006;rs78292414 | NA | 0 |
| 1 | 3:37560001-37600000 | 3:37680001-37720000 | 3.30E-13 | HiC | GSE87112 | Mesendoderm | intra | rs74985154;rs3733138;rs3733140;rs75619156;rs17229924;rs2162356;rs74786716;rs77820597;rs112585731;rs915631;rs79149273;rs17230261;rs199607377;rs2162355;rs76952775;rs75469174;rs78210776;rs77162103;rs75217006;rs78292414 | NA | 0 |
| 1 | 3:37560001-37600000 | 3:37720001-37760000 | 2.10E-20 | HiC | GSE87112 | Mesendoderm | intra | rs74985154;rs3733138;rs3733140;rs75619156;rs17229924;rs2162356;rs74786716;rs77820597;rs112585731;rs915631;rs79149273;rs17230261;rs199607377;rs2162355;rs76952775;rs75469174;rs78210776;rs77162103;rs75217006;rs78292414 | NA | 0 |
| 1 | 3:37560001-37600000 | 3:37760001-37800000 | 1.07E-38 | HiC | GSE87112 | Mesendoderm | intra | rs74985154;rs3733138;rs3733140;rs75619156;rs17229924;rs2162356;rs74786716;rs77820597;rs112585731;rs915631;rs79149273;rs17230261;rs199607377;rs2162355;rs76952775;rs75469174;rs78210776;rs77162103;rs75217006;rs78292414 | NA | 0 |
| 1 | 3:37560001-37600000 | 3:37800001-37840000 | 1.37E-35 | HiC | GSE87112 | Mesendoderm | intra | rs74985154;rs3733138;rs3733140;rs75619156;rs17229924;rs2162356;rs74786716;rs77820597;rs112585731;rs915631;rs79149273;rs17230261;rs199607377;rs2162355;rs76952775;rs75469174;rs78210776;rs77162103;rs75217006;rs78292414 | NA | 0 |
| 1 | 3:37560001-37600000 | 3:37840001-37880000 | 5.27E-23 | HiC | GSE87112 | Mesendoderm | intra | rs74985154;rs3733138;rs3733140;rs75619156;rs17229924;rs2162356;rs74786716;rs77820597;rs112585731;rs915631;rs79149273;rs17230261;rs199607377;rs2162355;rs76952775;rs75469174;rs78210776;rs77162103;rs75217006;rs78292414 | NA | 0 |
| 1 | 3:37560001-37600000 | 3:37920001-37960000 | 9.13E-08 | HiC | GSE87112 | Mesendoderm | intra | rs74985154;rs3733138;rs3733140;rs75619156;rs17229924;rs2162356;rs74786716;rs77820597;rs112585731;rs915631;rs79149273;rs17230261;rs199607377;rs2162355;rs76952775;rs75469174;rs78210776;rs77162103;rs75217006;rs78292414 | NA | 0 |
| 1 | 3:37560001-37600000 | 3:38000001-38040000 | 1.35E-07 | HiC | GSE87112 | Mesendoderm | intra | rs74985154;rs3733138;rs3733140;rs75619156;rs17229924;rs2162356;rs74786716;rs77820597;rs112585731;rs915631;rs79149273;rs17230261;rs199607377;rs2162355;rs76952775;rs75469174;rs78210776;rs77162103;rs75217006;rs78292414 | ENSG00000136059 | 0 |
| 2 | 3:96520001-96560000 | 3:97080001-97120000 | 3.72E-07 | HiC | GSE87112 | Mesendoderm | intra | rs62262901;rs62262902;rs62262903;rs62262930;rs151165295;rs62262931;rs62262933;rs62262934;rs74641574;rs79645223;rs2318156;rs2318155;rs2318154 | NA | 0 |
| 2 | 3:96520001-96560000 | 3:97120001-97160000 | 3.70E-12 | HiC | GSE87112 | Mesendoderm | intra | rs62262901;rs62262902;rs62262903;rs62262930;rs151165295;rs62262931;rs62262933;rs62262934;rs74641574;rs79645223;rs2318156;rs2318155;rs2318154 | NA | 0 |
| 2 | 3:96520001-96560000 | 3:97200001-97240000 | 8.46E-07 | HiC | GSE87112 | Mesendoderm | intra | rs62262901;rs62262902;rs62262903;rs62262930;rs151165295;rs62262931;rs62262933;rs62262934;rs74641574;rs79645223;rs2318156;rs2318155;rs2318154 | NA | 0 |
| 2 | 3:96520001-96560000 | 3:97280001-97320000 | 4.32E-23 | HiC | GSE87112 | Mesendoderm | intra | rs62262901;rs62262902;rs62262903;rs62262930;rs151165295;rs62262931;rs62262933;rs62262934;rs74641574;rs79645223;rs2318156;rs2318155;rs2318154 | NA | 0 |
| 2 | 3:96520001-96560000 | 3:97320001-97360000 | 5.06E-10 | HiC | GSE87112 | Mesendoderm | intra | rs62262901;rs62262902;rs62262903;rs62262930;rs151165295;rs62262931;rs62262933;rs62262934;rs74641574;rs79645223;rs2318156;rs2318155;rs2318154 | NA | 0 |
| 2 | 3:96520001-96560000 | 3:97360001-97400000 | 1.86E-49 | HiC | GSE87112 | Mesendoderm | intra | rs62262901;rs62262902;rs62262903;rs62262930;rs151165295;rs62262931;rs62262933;rs62262934;rs74641574;rs79645223;rs2318156;rs2318155;rs2318154 | NA | 0 |
| 2 | 3:96520001-96560000 | 3:97400001-97440000 | 1.90E-42 | HiC | GSE87112 | Mesendoderm | intra | rs62262901;rs62262902;rs62262903;rs62262930;rs151165295;rs62262931;rs62262933;rs62262934;rs74641574;rs79645223;rs2318156;rs2318155;rs2318154 | NA | 0 |
| 2 | 3:96520001-96560000 | 3:97440001-97480000 | 4.82E-30 | HiC | GSE87112 | Mesendoderm | intra | rs62262901;rs62262902;rs62262903;rs62262930;rs151165295;rs62262931;rs62262933;rs62262934;rs74641574;rs79645223;rs2318156;rs2318155;rs2318154 | NA | 0 |
| 2 | 3:96520001-96560000 | 3:97480001-97520000 | 1.20E-12 | HiC | GSE87112 | Mesendoderm | intra | rs62262901;rs62262902;rs62262903;rs62262930;rs151165295;rs62262931;rs62262933;rs62262934;rs74641574;rs79645223;rs2318156;rs2318155;rs2318154 | ENSG00000113966 | 0 |
| 2 | 3:96520001-96560000 | 3:97520001-97560000 | 3.44E-18 | HiC | GSE87112 | Mesendoderm | intra | rs62262901;rs62262902;rs62262903;rs62262930;rs151165295;rs62262931;rs62262933;rs62262934;rs74641574;rs79645223;rs2318156;rs2318155;rs2318154 | ENSG00000233280 | 0 |
| 2 | 3:96520001-96560000 | 3:97560001-97600000 | 6.51E-09 | HiC | GSE87112 | Mesendoderm | intra | rs62262901;rs62262902;rs62262903;rs62262930;rs151165295;rs62262931;rs62262933;rs62262934;rs74641574;rs79645223;rs2318156;rs2318155;rs2318154 | ENSG00000080200 | 0 |
| 2 | 3:96520001-96560000 | 3:97600001-97640000 | 3.32E-08 | HiC | GSE87112 | Mesendoderm | intra | rs62262901;rs62262902;rs62262903;rs62262930;rs151165295;rs62262931;rs62262933;rs62262934;rs74641574;rs79645223;rs2318156;rs2318155;rs2318154 | NA | 0 |
| 2 | 3:96520001-96560000 | 3:97640001-97680000 | 5.54E-25 | HiC | GSE87112 | Mesendoderm | intra | rs62262901;rs62262902;rs62262903;rs62262930;rs151165295;rs62262931;rs62262933;rs62262934;rs74641574;rs79645223;rs2318156;rs2318155;rs2318154 | NA | 0 |
| 2 | 3:96520001-96560000 | 3:97680001-97720000 | 5.14E-09 | HiC | GSE87112 | Mesendoderm | intra | rs62262901;rs62262902;rs62262903;rs62262930;rs151165295;rs62262931;rs62262933;rs62262934;rs74641574;rs79645223;rs2318156;rs2318155;rs2318154 | ENSG00000170854 | 0 |
| 2 | 3:96520001-96560000 | 3:97720001-97760000 | 3.66E-20 | HiC | GSE87112 | Mesendoderm | intra | rs62262901;rs62262902;rs62262903;rs62262930;rs151165295;rs62262931;rs62262933;rs62262934;rs74641574;rs79645223;rs2318156;rs2318155;rs2318154 | NA | 0 |
| 2 | 3:96520001-96560000 | 3:98240001-98280000 | 5.56E-07 | HiC | GSE87112 | Mesendoderm | intra | rs62262901;rs62262902;rs62262903;rs62262930;rs151165295;rs62262931;rs62262933;rs62262934;rs74641574;rs79645223;rs2318156;rs2318155;rs2318154 | ENSG00000080822:ENSG00000154165 | 0 |
| 2 | 3:96560001-96600000 | 3:96920001-96960000 | 1.64E-17 | HiC | GSE87112 | Mesendoderm | intra | rs16836353;rs62262935;rs62262936;rs62262937;rs62262938;rs62262940;rs62262941;rs62262942;rs62262944;rs62262946;rs62262947;rs62262948;rs62262949;rs62262950;rs62262951 | NA | 0 |
| 2 | 3:96560001-96600000 | 3:97080001-97120000 | 4.52E-09 | HiC | GSE87112 | Mesendoderm | intra | rs16836353;rs62262935;rs62262936;rs62262937;rs62262938;rs62262940;rs62262941;rs62262942;rs62262944;rs62262946;rs62262947;rs62262948;rs62262949;rs62262950;rs62262951 | NA | 0 |
| 2 | 3:96560001-96600000 | 3:97120001-97160000 | 4.13E-20 | HiC | GSE87112 | Mesendoderm | intra | rs16836353;rs62262935;rs62262936;rs62262937;rs62262938;rs62262940;rs62262941;rs62262942;rs62262944;rs62262946;rs62262947;rs62262948;rs62262949;rs62262950;rs62262951 | NA | 0 |
| 2 | 3:96560001-96600000 | 3:97200001-97240000 | 8.26E-12 | HiC | GSE87112 | Mesendoderm | intra | rs16836353;rs62262935;rs62262936;rs62262937;rs62262938;rs62262940;rs62262941;rs62262942;rs62262944;rs62262946;rs62262947;rs62262948;rs62262949;rs62262950;rs62262951 | NA | 0 |
| 2 | 3:96560001-96600000 | 3:97240001-97280000 | 2.25E-07 | HiC | GSE87112 | Mesendoderm | intra | rs16836353;rs62262935;rs62262936;rs62262937;rs62262938;rs62262940;rs62262941;rs62262942;rs62262944;rs62262946;rs62262947;rs62262948;rs62262949;rs62262950;rs62262951 | NA | 0 |
| 2 | 3:96560001-96600000 | 3:97280001-97320000 | 3.94E-31 | HiC | GSE87112 | Mesendoderm | intra | rs16836353;rs62262935;rs62262936;rs62262937;rs62262938;rs62262940;rs62262941;rs62262942;rs62262944;rs62262946;rs62262947;rs62262948;rs62262949;rs62262950;rs62262951 | NA | 0 |
| 2 | 3:96560001-96600000 | 3:97320001-97360000 | 2.16E-18 | HiC | GSE87112 | Mesendoderm | intra | rs16836353;rs62262935;rs62262936;rs62262937;rs62262938;rs62262940;rs62262941;rs62262942;rs62262944;rs62262946;rs62262947;rs62262948;rs62262949;rs62262950;rs62262951 | NA | 0 |
| 2 | 3:96560001-96600000 | 3:97360001-97400000 | 1.08E-75 | HiC | GSE87112 | Mesendoderm | intra | rs16836353;rs62262935;rs62262936;rs62262937;rs62262938;rs62262940;rs62262941;rs62262942;rs62262944;rs62262946;rs62262947;rs62262948;rs62262949;rs62262950;rs62262951 | NA | 0 |
| 2 | 3:96560001-96600000 | 3:97400001-97440000 | 1.70E-53 | HiC | GSE87112 | Mesendoderm | intra | rs16836353;rs62262935;rs62262936;rs62262937;rs62262938;rs62262940;rs62262941;rs62262942;rs62262944;rs62262946;rs62262947;rs62262948;rs62262949;rs62262950;rs62262951 | NA | 0 |
| 2 | 3:96560001-96600000 | 3:97440001-97480000 | 9.64E-44 | HiC | GSE87112 | Mesendoderm | intra | rs16836353;rs62262935;rs62262936;rs62262937;rs62262938;rs62262940;rs62262941;rs62262942;rs62262944;rs62262946;rs62262947;rs62262948;rs62262949;rs62262950;rs62262951 | NA | 0 |
| 2 | 3:96560001-96600000 | 3:97480001-97520000 | 6.20E-15 | HiC | GSE87112 | Mesendoderm | intra | rs16836353;rs62262935;rs62262936;rs62262937;rs62262938;rs62262940;rs62262941;rs62262942;rs62262944;rs62262946;rs62262947;rs62262948;rs62262949;rs62262950;rs62262951 | ENSG00000113966 | 0 |
| 2 | 3:96560001-96600000 | 3:97520001-97560000 | 4.40E-13 | HiC | GSE87112 | Mesendoderm | intra | rs16836353;rs62262935;rs62262936;rs62262937;rs62262938;rs62262940;rs62262941;rs62262942;rs62262944;rs62262946;rs62262947;rs62262948;rs62262949;rs62262950;rs62262951 | ENSG00000233280 | 0 |
| 2 | 3:96560001-96600000 | 3:97640001-97680000 | 2.44E-17 | HiC | GSE87112 | Mesendoderm | intra | rs16836353;rs62262935;rs62262936;rs62262937;rs62262938;rs62262940;rs62262941;rs62262942;rs62262944;rs62262946;rs62262947;rs62262948;rs62262949;rs62262950;rs62262951 | NA | 0 |
| 2 | 3:96560001-96600000 | 3:97720001-97760000 | 1.94E-32 | HiC | GSE87112 | Mesendoderm | intra | rs16836353;rs62262935;rs62262936;rs62262937;rs62262938;rs62262940;rs62262941;rs62262942;rs62262944;rs62262946;rs62262947;rs62262948;rs62262949;rs62262950;rs62262951 | NA | 0 |
| 2 | 3:96600001-96640000 | 3:96680001-96720000 | 4.62E-38 | HiC | GSE87112 | Mesendoderm | intra | rs62262952;rs62262953;rs116253310;rs76857958;rs111706803;rs62262955;rs62262957;rs62262958;rs62263676;rs187619159;rs62263677;rs62263678;rs574559070;rs534937763;rs62263680;rs62263682;rs62263684;rs16836982;rs16836986;rs112527011;rs62263685;rs62263686 | NA | 0 |
| 2 | 3:96600001-96640000 | 3:96760001-96800000 | 1.30E-09 | HiC | GSE87112 | Mesendoderm | intra | rs62262952;rs62262953;rs116253310;rs76857958;rs111706803;rs62262955;rs62262957;rs62262958;rs62263676;rs187619159;rs62263677;rs62263678;rs574559070;rs534937763;rs62263680;rs62263682;rs62263684;rs16836982;rs16836986;rs112527011;rs62263685;rs62263686 | NA | 0 |
| 2 | 3:96600001-96640000 | 3:96800001-96840000 | 5.06E-08 | HiC | GSE87112 | Mesendoderm | intra | rs62262952;rs62262953;rs116253310;rs76857958;rs111706803;rs62262955;rs62262957;rs62262958;rs62263676;rs187619159;rs62263677;rs62263678;rs574559070;rs534937763;rs62263680;rs62263682;rs62263684;rs16836982;rs16836986;rs112527011;rs62263685;rs62263686 | NA | 0 |
| 2 | 3:96600001-96640000 | 3:96920001-96960000 | 1.07E-21 | HiC | GSE87112 | Mesendoderm | intra | rs62262952;rs62262953;rs116253310;rs76857958;rs111706803;rs62262955;rs62262957;rs62262958;rs62263676;rs187619159;rs62263677;rs62263678;rs574559070;rs534937763;rs62263680;rs62263682;rs62263684;rs16836982;rs16836986;rs112527011;rs62263685;rs62263686 | NA | 0 |
| 2 | 3:96600001-96640000 | 3:97000001-97040000 | 4.79E-21 | HiC | GSE87112 | Mesendoderm | intra | rs62262952;rs62262953;rs116253310;rs76857958;rs111706803;rs62262955;rs62262957;rs62262958;rs62263676;rs187619159;rs62263677;rs62263678;rs574559070;rs534937763;rs62263680;rs62263682;rs62263684;rs16836982;rs16836986;rs112527011;rs62263685;rs62263686 | NA | 0 |
| 2 | 3:96600001-96640000 | 3:97080001-97120000 | 2.85E-19 | HiC | GSE87112 | Mesendoderm | intra | rs62262952;rs62262953;rs116253310;rs76857958;rs111706803;rs62262955;rs62262957;rs62262958;rs62263676;rs187619159;rs62263677;rs62263678;rs574559070;rs534937763;rs62263680;rs62263682;rs62263684;rs16836982;rs16836986;rs112527011;rs62263685;rs62263686 | NA | 0 |
| 2 | 3:96600001-96640000 | 3:97120001-97160000 | 2.22E-31 | HiC | GSE87112 | Mesendoderm | intra | rs62262952;rs62262953;rs116253310;rs76857958;rs111706803;rs62262955;rs62262957;rs62262958;rs62263676;rs187619159;rs62263677;rs62263678;rs574559070;rs534937763;rs62263680;rs62263682;rs62263684;rs16836982;rs16836986;rs112527011;rs62263685;rs62263686 | NA | 0 |
| 2 | 3:96600001-96640000 | 3:97160001-97200000 | 2.91E-12 | HiC | GSE87112 | Mesendoderm | intra | rs62262952;rs62262953;rs116253310;rs76857958;rs111706803;rs62262955;rs62262957;rs62262958;rs62263676;rs187619159;rs62263677;rs62263678;rs574559070;rs534937763;rs62263680;rs62263682;rs62263684;rs16836982;rs16836986;rs112527011;rs62263685;rs62263686 | NA | 0 |
| 2 | 3:96600001-96640000 | 3:97200001-97240000 | 3.47E-16 | HiC | GSE87112 | Mesendoderm | intra | rs62262952;rs62262953;rs116253310;rs76857958;rs111706803;rs62262955;rs62262957;rs62262958;rs62263676;rs187619159;rs62263677;rs62263678;rs574559070;rs534937763;rs62263680;rs62263682;rs62263684;rs16836982;rs16836986;rs112527011;rs62263685;rs62263686 | NA | 0 |
| 2 | 3:96600001-96640000 | 3:97240001-97280000 | 7.06E-13 | HiC | GSE87112 | Mesendoderm | intra | rs62262952;rs62262953;rs116253310;rs76857958;rs111706803;rs62262955;rs62262957;rs62262958;rs62263676;rs187619159;rs62263677;rs62263678;rs574559070;rs534937763;rs62263680;rs62263682;rs62263684;rs16836982;rs16836986;rs112527011;rs62263685;rs62263686 | NA | 0 |
| 2 | 3:96600001-96640000 | 3:97280001-97320000 | 3.14E-38 | HiC | GSE87112 | Mesendoderm | intra | rs62262952;rs62262953;rs116253310;rs76857958;rs111706803;rs62262955;rs62262957;rs62262958;rs62263676;rs187619159;rs62263677;rs62263678;rs574559070;rs534937763;rs62263680;rs62263682;rs62263684;rs16836982;rs16836986;rs112527011;rs62263685;rs62263686 | NA | 0 |
| 2 | 3:96600001-96640000 | 3:97320001-97360000 | 1.53E-22 | HiC | GSE87112 | Mesendoderm | intra | rs62262952;rs62262953;rs116253310;rs76857958;rs111706803;rs62262955;rs62262957;rs62262958;rs62263676;rs187619159;rs62263677;rs62263678;rs574559070;rs534937763;rs62263680;rs62263682;rs62263684;rs16836982;rs16836986;rs112527011;rs62263685;rs62263686 | NA | 0 |
| 2 | 3:96600001-96640000 | 3:97360001-97400000 | 1.55E-62 | HiC | GSE87112 | Mesendoderm | intra | rs62262952;rs62262953;rs116253310;rs76857958;rs111706803;rs62262955;rs62262957;rs62262958;rs62263676;rs187619159;rs62263677;rs62263678;rs574559070;rs534937763;rs62263680;rs62263682;rs62263684;rs16836982;rs16836986;rs112527011;rs62263685;rs62263686 | NA | 0 |
| 2 | 3:96600001-96640000 | 3:97400001-97440000 | 4.89E-53 | HiC | GSE87112 | Mesendoderm | intra | rs62262952;rs62262953;rs116253310;rs76857958;rs111706803;rs62262955;rs62262957;rs62262958;rs62263676;rs187619159;rs62263677;rs62263678;rs574559070;rs534937763;rs62263680;rs62263682;rs62263684;rs16836982;rs16836986;rs112527011;rs62263685;rs62263686 | NA | 0 |
| 2 | 3:96600001-96640000 | 3:97440001-97480000 | 6.99E-24 | HiC | GSE87112 | Mesendoderm | intra | rs62262952;rs62262953;rs116253310;rs76857958;rs111706803;rs62262955;rs62262957;rs62262958;rs62263676;rs187619159;rs62263677;rs62263678;rs574559070;rs534937763;rs62263680;rs62263682;rs62263684;rs16836982;rs16836986;rs112527011;rs62263685;rs62263686 | NA | 0 |
| 2 | 3:96600001-96640000 | 3:97480001-97520000 | 3.53E-24 | HiC | GSE87112 | Mesendoderm | intra | rs62262952;rs62262953;rs116253310;rs76857958;rs111706803;rs62262955;rs62262957;rs62262958;rs62263676;rs187619159;rs62263677;rs62263678;rs574559070;rs534937763;rs62263680;rs62263682;rs62263684;rs16836982;rs16836986;rs112527011;rs62263685;rs62263686 | ENSG00000113966 | 0 |
| 2 | 3:96600001-96640000 | 3:97520001-97560000 | 8.79E-17 | HiC | GSE87112 | Mesendoderm | intra | rs62262952;rs62262953;rs116253310;rs76857958;rs111706803;rs62262955;rs62262957;rs62262958;rs62263676;rs187619159;rs62263677;rs62263678;rs574559070;rs534937763;rs62263680;rs62263682;rs62263684;rs16836982;rs16836986;rs112527011;rs62263685;rs62263686 | ENSG00000233280 | 0 |
| 2 | 3:96600001-96640000 | 3:97560001-97600000 | 1.48E-10 | HiC | GSE87112 | Mesendoderm | intra | rs62262952;rs62262953;rs116253310;rs76857958;rs111706803;rs62262955;rs62262957;rs62262958;rs62263676;rs187619159;rs62263677;rs62263678;rs574559070;rs534937763;rs62263680;rs62263682;rs62263684;rs16836982;rs16836986;rs112527011;rs62263685;rs62263686 | ENSG00000080200 | 0 |
| 2 | 3:96600001-96640000 | 3:97640001-97680000 | 1.82E-34 | HiC | GSE87112 | Mesendoderm | intra | rs62262952;rs62262953;rs116253310;rs76857958;rs111706803;rs62262955;rs62262957;rs62262958;rs62263676;rs187619159;rs62263677;rs62263678;rs574559070;rs534937763;rs62263680;rs62263682;rs62263684;rs16836982;rs16836986;rs112527011;rs62263685;rs62263686 | NA | 0 |
| 2 | 3:96600001-96640000 | 3:97720001-97760000 | 2.91E-31 | HiC | GSE87112 | Mesendoderm | intra | rs62262952;rs62262953;rs116253310;rs76857958;rs111706803;rs62262955;rs62262957;rs62262958;rs62263676;rs187619159;rs62263677;rs62263678;rs574559070;rs534937763;rs62263680;rs62263682;rs62263684;rs16836982;rs16836986;rs112527011;rs62263685;rs62263686 | NA | 0 |
| 2 | 3:96640001-96680000 | 3:97360001-97400000 | 5.86E-07 | HiC | GSE87112 | Mesendoderm | intra | rs62263687;rs62263693;rs2318066;rs62263694;rs74563548;rs62263696;rs185127539;rs145486125;rs62263697;rs148690862;rs62263699;rs149222683;rs111574505;rs62263701;rs62263702;rs62263703;rs75261331;rs62263704;rs189373463 | NA | 0 |
| 2 | 3:96680001-96720000 | 3:96760001-96800000 | 2.08E-14 | HiC | GSE87112 | Mesendoderm | intra | rs62263705;rs62263706;rs6762348;rs62263709;rs137983052;rs62263734;rs2856474;rs2612274;rs553308194 | NA | 0 |
| 2 | 3:96680001-96720000 | 3:96880001-96920000 | 7.47E-10 | HiC | GSE87112 | Mesendoderm | intra | rs62263705;rs62263706;rs6762348;rs62263709;rs137983052;rs62263734;rs2856474;rs2612274;rs553308194 | NA | 0 |
| 2 | 3:96680001-96720000 | 3:96920001-96960000 | 3.38E-28 | HiC | GSE87112 | Mesendoderm | intra | rs62263705;rs62263706;rs6762348;rs62263709;rs137983052;rs62263734;rs2856474;rs2612274;rs553308194 | NA | 0 |
| 2 | 3:96680001-96720000 | 3:96960001-97000000 | 3.01E-12 | HiC | GSE87112 | Mesendoderm | intra | rs62263705;rs62263706;rs6762348;rs62263709;rs137983052;rs62263734;rs2856474;rs2612274;rs553308194 | NA | 0 |
| 2 | 3:96680001-96720000 | 3:97000001-97040000 | 1.06E-18 | HiC | GSE87112 | Mesendoderm | intra | rs62263705;rs62263706;rs6762348;rs62263709;rs137983052;rs62263734;rs2856474;rs2612274;rs553308194 | NA | 0 |
| 2 | 3:96680001-96720000 | 3:97080001-97120000 | 1.95E-24 | HiC | GSE87112 | Mesendoderm | intra | rs62263705;rs62263706;rs6762348;rs62263709;rs137983052;rs62263734;rs2856474;rs2612274;rs553308194 | NA | 0 |
| 2 | 3:96680001-96720000 | 3:97120001-97160000 | 2.91E-40 | HiC | GSE87112 | Mesendoderm | intra | rs62263705;rs62263706;rs6762348;rs62263709;rs137983052;rs62263734;rs2856474;rs2612274;rs553308194 | NA | 0 |
| 2 | 3:96680001-96720000 | 3:97160001-97200000 | 2.50E-12 | HiC | GSE87112 | Mesendoderm | intra | rs62263705;rs62263706;rs6762348;rs62263709;rs137983052;rs62263734;rs2856474;rs2612274;rs553308194 | NA | 0 |
| 2 | 3:96680001-96720000 | 3:97200001-97240000 | 1.78E-25 | HiC | GSE87112 | Mesendoderm | intra | rs62263705;rs62263706;rs6762348;rs62263709;rs137983052;rs62263734;rs2856474;rs2612274;rs553308194 | NA | 0 |
| 2 | 3:96680001-96720000 | 3:97240001-97280000 | 2.54E-17 | HiC | GSE87112 | Mesendoderm | intra | rs62263705;rs62263706;rs6762348;rs62263709;rs137983052;rs62263734;rs2856474;rs2612274;rs553308194 | NA | 0 |
| 2 | 3:96680001-96720000 | 3:97280001-97320000 | 1.05E-57 | HiC | GSE87112 | Mesendoderm | intra | rs62263705;rs62263706;rs6762348;rs62263709;rs137983052;rs62263734;rs2856474;rs2612274;rs553308194 | NA | 0 |
| 2 | 3:96680001-96720000 | 3:97320001-97360000 | 1.46E-24 | HiC | GSE87112 | Mesendoderm | intra | rs62263705;rs62263706;rs6762348;rs62263709;rs137983052;rs62263734;rs2856474;rs2612274;rs553308194 | NA | 0 |
| 2 | 3:96680001-96720000 | 3:97360001-97400000 | 6.07E-89 | HiC | GSE87112 | Mesendoderm | intra | rs62263705;rs62263706;rs6762348;rs62263709;rs137983052;rs62263734;rs2856474;rs2612274;rs553308194 | NA | 0 |
| 2 | 3:96680001-96720000 | 3:97400001-97440000 | 1.31E-65 | HiC | GSE87112 | Mesendoderm | intra | rs62263705;rs62263706;rs6762348;rs62263709;rs137983052;rs62263734;rs2856474;rs2612274;rs553308194 | NA | 0 |
| 2 | 3:96680001-96720000 | 3:97440001-97480000 | 1.88E-36 | HiC | GSE87112 | Mesendoderm | intra | rs62263705;rs62263706;rs6762348;rs62263709;rs137983052;rs62263734;rs2856474;rs2612274;rs553308194 | NA | 0 |
| 2 | 3:96680001-96720000 | 3:97480001-97520000 | 1.66E-11 | HiC | GSE87112 | Mesendoderm | intra | rs62263705;rs62263706;rs6762348;rs62263709;rs137983052;rs62263734;rs2856474;rs2612274;rs553308194 | ENSG00000113966 | 0 |
| 2 | 3:96680001-96720000 | 3:97520001-97560000 | 3.57E-36 | HiC | GSE87112 | Mesendoderm | intra | rs62263705;rs62263706;rs6762348;rs62263709;rs137983052;rs62263734;rs2856474;rs2612274;rs553308194 | ENSG00000233280 | 0 |
| 2 | 3:96680001-96720000 | 3:97560001-97600000 | 1.26E-09 | HiC | GSE87112 | Mesendoderm | intra | rs62263705;rs62263706;rs6762348;rs62263709;rs137983052;rs62263734;rs2856474;rs2612274;rs553308194 | ENSG00000080200 | 0 |
| 2 | 3:96680001-96720000 | 3:97600001-97640000 | 2.18E-07 | HiC | GSE87112 | Mesendoderm | intra | rs62263705;rs62263706;rs6762348;rs62263709;rs137983052;rs62263734;rs2856474;rs2612274;rs553308194 | NA | 0 |
| 2 | 3:96680001-96720000 | 3:97640001-97680000 | 6.26E-31 | HiC | GSE87112 | Mesendoderm | intra | rs62263705;rs62263706;rs6762348;rs62263709;rs137983052;rs62263734;rs2856474;rs2612274;rs553308194 | NA | 0 |
| 2 | 3:96680001-96720000 | 3:97720001-97760000 | 1.98E-36 | HiC | GSE87112 | Mesendoderm | intra | rs62263705;rs62263706;rs6762348;rs62263709;rs137983052;rs62263734;rs2856474;rs2612274;rs553308194 | NA | 0 |
| 2 | 3:96680001-96720000 | 3:98240001-98280000 | 5.45E-10 | HiC | GSE87112 | Mesendoderm | intra | rs62263705;rs62263706;rs6762348;rs62263709;rs137983052;rs62263734;rs2856474;rs2612274;rs553308194 | ENSG00000080822:ENSG00000154165 | 0 |
| 2 | 3:96720001-96760000 | 3:97360001-97400000 | 7.06E-13 | HiC | GSE87112 | Mesendoderm | intra | rs2027816;rs2856463;rs2019818;rs115680726;rs112449918;rs565327317;rs2213251;rs2213252;rs2856466 | NA | 0 |
| 2 | 3:96720001-96760000 | 3:97400001-97440000 | 2.25E-07 | HiC | GSE87112 | Mesendoderm | intra | rs2027816;rs2856463;rs2019818;rs115680726;rs112449918;rs565327317;rs2213251;rs2213252;rs2856466 | NA | 0 |
| 2 | 3:96720001-96760000 | 3:97440001-97480000 | 2.98E-07 | HiC | GSE87112 | Mesendoderm | intra | rs2027816;rs2856463;rs2019818;rs115680726;rs112449918;rs565327317;rs2213251;rs2213252;rs2856466 | NA | 0 |
| 3 | 4:171440001-171480000 | 4:171480001-171520000 | 4.52E-23 | HiC | GSE87112 | Mesendoderm | intra | rs147274196 | NA | 0 |
| 3 | 4:171440001-171480000 | 4:171520001-171560000 | 2.30E-16 | HiC | GSE87112 | Mesendoderm | intra | rs147274196 | NA | 0 |
| 3 | 4:171440001-171480000 | 4:171560001-171600000 | 9.96E-09 | HiC | GSE87112 | Mesendoderm | intra | rs147274196 | NA | 0 |
| 3 | 4:171440001-171480000 | 4:171600001-171640000 | 7.95E-10 | HiC | GSE87112 | Mesendoderm | intra | rs147274196 | NA | 0 |
| 3 | 4:171440001-171480000 | 4:171640001-171680000 | 7.27E-28 | HiC | GSE87112 | Mesendoderm | intra | rs147274196 | NA | 0 |
| 3 | 4:171440001-171480000 | 4:171680001-171720000 | 5.52E-07 | HiC | GSE87112 | Mesendoderm | intra | rs147274196 | NA | 0 |
| 3 | 4:171440001-171480000 | 4:171760001-171800000 | 3.25E-17 | HiC | GSE87112 | Mesendoderm | intra | rs147274196 | NA | 0 |
| 3 | 4:171440001-171480000 | 4:171880001-171920000 | 8.51E-09 | HiC | GSE87112 | Mesendoderm | intra | rs147274196 | NA | 0 |
| 3 | 4:171440001-171480000 | 4:171960001-172000000 | 1.70E-19 | HiC | GSE87112 | Mesendoderm | intra | rs147274196 | NA | 0 |
| 3 | 4:171440001-171480000 | 4:172480001-172520000 | 1.52E-15 | HiC | GSE87112 | Mesendoderm | intra | rs147274196 | NA | 0 |
| 3 | 4:171440001-171480000 | 4:172520001-172560000 | 5.54E-13 | HiC | GSE87112 | Mesendoderm | intra | rs147274196 | NA | 0 |
| 3 | 4:171440001-171480000 | 4:172560001-172600000 | 1.24E-09 | HiC | GSE87112 | Mesendoderm | intra | rs147274196 | NA | 0 |
| 3 | 4:171440001-171480000 | 4:172600001-172640000 | 2.91E-12 | HiC | GSE87112 | Mesendoderm | intra | rs147274196 | NA | 0 |
| 3 | 4:171440001-171480000 | 4:173040001-173080000 | 1.69E-07 | HiC | GSE87112 | Mesendoderm | intra | rs147274196 | NA | 0 |
| 3 | 4:171440001-171480000 | 4:173080001-173120000 | 2.34E-08 | HiC | GSE87112 | Mesendoderm | intra | rs147274196 | NA | 0 |
| 3 | 4:171440001-171480000 | 4:173120001-173160000 | 2.61E-07 | HiC | GSE87112 | Mesendoderm | intra | rs147274196 | NA | 0 |
| 3 | 4:171440001-171480000 | 4:173760001-173800000 | 6.76E-07 | HiC | GSE87112 | Mesendoderm | intra | rs147274196 | NA | 0 |
| 3 | 4:171480001-171520000 | 4:171520001-171560000 | 5.07E-37 | HiC | GSE87112 | Mesendoderm | intra | rs148420952;rs76413646 | NA | 0 |
| 3 | 4:171480001-171520000 | 4:171560001-171600000 | 2.45E-11 | HiC | GSE87112 | Mesendoderm | intra | rs148420952;rs76413646 | NA | 0 |
| 3 | 4:171480001-171520000 | 4:171600001-171640000 | 2.21E-13 | HiC | GSE87112 | Mesendoderm | intra | rs148420952;rs76413646 | NA | 0 |
| 3 | 4:171480001-171520000 | 4:171640001-171680000 | 9.72E-37 | HiC | GSE87112 | Mesendoderm | intra | rs148420952;rs76413646 | NA | 0 |
| 3 | 4:171480001-171520000 | 4:171680001-171720000 | 3.06E-10 | HiC | GSE87112 | Mesendoderm | intra | rs148420952;rs76413646 | NA | 0 |
| 3 | 4:171480001-171520000 | 4:171760001-171800000 | 1.46E-43 | HiC | GSE87112 | Mesendoderm | intra | rs148420952;rs76413646 | NA | 0 |
| 3 | 4:171480001-171520000 | 4:171800001-171840000 | 2.98E-10 | HiC | GSE87112 | Mesendoderm | intra | rs148420952;rs76413646 | NA | 0 |
| 3 | 4:171480001-171520000 | 4:171840001-171880000 | 1.63E-07 | HiC | GSE87112 | Mesendoderm | intra | rs148420952;rs76413646 | NA | 0 |
| 3 | 4:171480001-171520000 | 4:171880001-171920000 | 2.45E-11 | HiC | GSE87112 | Mesendoderm | intra | rs148420952;rs76413646 | NA | 0 |
| 3 | 4:171480001-171520000 | 4:171960001-172000000 | 3.27E-19 | HiC | GSE87112 | Mesendoderm | intra | rs148420952;rs76413646 | NA | 0 |
| 3 | 4:171480001-171520000 | 4:172080001-172120000 | 3.98E-11 | HiC | GSE87112 | Mesendoderm | intra | rs148420952;rs76413646 | NA | 0 |
| 3 | 4:171480001-171520000 | 4:172480001-172520000 | 1.29E-17 | HiC | GSE87112 | Mesendoderm | intra | rs148420952;rs76413646 | NA | 0 |
| 3 | 4:171480001-171520000 | 4:172520001-172560000 | 3.33E-27 | HiC | GSE87112 | Mesendoderm | intra | rs148420952;rs76413646 | NA | 0 |
| 3 | 4:171480001-171520000 | 4:172560001-172600000 | 4.29E-12 | HiC | GSE87112 | Mesendoderm | intra | rs148420952;rs76413646 | NA | 0 |
| 3 | 4:171480001-171520000 | 4:172600001-172640000 | 1.24E-09 | HiC | GSE87112 | Mesendoderm | intra | rs148420952;rs76413646 | NA | 0 |
| 3 | 4:171480001-171520000 | 4:172680001-172720000 | 1.40E-07 | HiC | GSE87112 | Mesendoderm | intra | rs148420952;rs76413646 | NA | 0 |
| 3 | 4:171480001-171520000 | 4:172720001-172760000 | 7.40E-08 | HiC | GSE87112 | Mesendoderm | intra | rs148420952;rs76413646 | ENSG00000174473 | 0 |
| 3 | 4:171480001-171520000 | 4:172840001-172880000 | 7.44E-07 | HiC | GSE87112 | Mesendoderm | intra | rs148420952;rs76413646 | NA | 0 |
| 3 | 4:171480001-171520000 | 4:172960001-173000000 | 4.24E-07 | HiC | GSE87112 | Mesendoderm | intra | rs148420952;rs76413646 | NA | 0 |
| 3 | 4:171480001-171520000 | 4:173040001-173080000 | 2.06E-10 | HiC | GSE87112 | Mesendoderm | intra | rs148420952;rs76413646 | NA | 0 |
| 3 | 4:171480001-171520000 | 4:173080001-173120000 | 5.28E-16 | HiC | GSE87112 | Mesendoderm | intra | rs148420952;rs76413646 | NA | 0 |
| 3 | 4:171480001-171520000 | 4:173120001-173160000 | 5.71E-07 | HiC | GSE87112 | Mesendoderm | intra | rs148420952;rs76413646 | NA | 0 |
| 3 | 4:171480001-171520000 | 4:173880001-173920000 | 7.86E-07 | HiC | GSE87112 | Mesendoderm | intra | rs148420952;rs76413646 | NA | 0 |
| 4 | 5:108560001-108600000 | 5:108800001-108840000 | 2.79E-22 | HiC | GSE87112 | Mesendoderm | intra | rs35812497;rs75803484;rs75073283;rs76430100;rs75782477;rs77992445;rs61701247;rs17161655;rs115979287;rs17161659;rs151072743;rs17161665;rs6863688;5:108591279:C:T;5:108591279:A:T;rs1363213;rs1862201;rs1862202;rs1862203;rs5870359 | NA | 0 |
| 4 | 5:108560001-108600000 | 5:109160001-109200000 | 2.67E-07 | HiC | GSE87112 | Mesendoderm | intra | rs35812497;rs75803484;rs75073283;rs76430100;rs75782477;rs77992445;rs61701247;rs17161655;rs115979287;rs17161659;rs151072743;rs17161665;rs6863688;5:108591279:C:T;5:108591279:A:T;rs1363213;rs1862201;rs1862202;rs1862203;rs5870359 | NA | 0 |
| 4 | 5:108600001-108640000 | 5:108800001-108840000 | 5.24E-18 | HiC | GSE87112 | Mesendoderm | intra | rs11744353;rs79776200;rs76069623;rs76043709;rs78729571;rs36123121;rs11748450;rs80234158;rs78146542;rs185072862;rs137867205;rs189006725;rs4388251;rs4438924;rs4541698;rs4388252;rs4392675;rs200252534;rs145520315;rs147699420;rs77803982;rs79469094;rs75077625;rs74379173;rs78664536;rs549536839;rs570631944;rs78784808;rs78340649;rs201569786;rs75649455;rs78814720;rs77561644;rs76565008;rs74436342;rs1592807;rs1592810;rs1592811;rs7700458;rs6594365;rs6863893;rs140163909;rs146837743;rs7705919;rs201046346;rs202152005;rs199869120;rs111726541;rs373673987;rs113026830;rs148211606;rs201619508;rs201169921;rs146644914;rs116649742;rs79893293;rs57629433;rs60335509;rs58453996;rs59466242;rs60878839;rs11743452;rs1833567;rs1833568;rs1833569;rs7721272;rs7721427;rs7721730;rs7721870;rs7722100;rs7704359;rs1895200;rs1895201;rs77974856;rs60905342;rs11746207;rs11739672;rs11749718;rs78518991;rs76243602;rs76383235;rs11741980;rs35243960;rs116462975;rs139801813;rs113103584;rs74727230;rs75919325;rs79624714;rs2080858;rs138092354;rs148285468;rs114455395;rs74850689;rs10463599 | NA | 0 |
| 5 | 11:2520001-2560000 | 11:2760001-2800000 | 6.73E-09 | HiC | GSE87112 | Mesendoderm | intra | rs111815403 | NA | 0 |
| 5 | 11:2520001-2560000 | 11:2960001-3000000 | 6.43E-07 | HiC | GSE87112 | Mesendoderm | intra | rs111815403 | NA | 0 |
| 6 | 12:80800001-80840000 | 12:80840001-80880000 | 7.95E-09 | HiC | GSE87112 | Mesendoderm | intra | rs117203215;rs77741796 | NA | 0 |
| 6 | 12:80800001-80840000 | 12:80880001-80920000 | 2.24E-28 | HiC | GSE87112 | Mesendoderm | intra | rs117203215;rs77741796 | NA | 0 |
| 6 | 12:80800001-80840000 | 12:80920001-80960000 | 2.39E-109 | HiC | GSE87112 | Mesendoderm | intra | rs117203215;rs77741796 | NA | 0 |
| 6 | 12:80800001-80840000 | 12:80960001-81000000 | 1.40E-18 | HiC | GSE87112 | Mesendoderm | intra | rs117203215;rs77741796 | NA | 0 |
| 6 | 12:80800001-80840000 | 12:81000001-81040000 | 3.39E-107 | HiC | GSE87112 | Mesendoderm | intra | rs117203215;rs77741796 | NA | 0 |
| 6 | 12:80800001-80840000 | 12:81040001-81080000 | 8.36E-72 | HiC | GSE87112 | Mesendoderm | intra | rs117203215;rs77741796 | NA | 0 |
| 6 | 12:80800001-80840000 | 12:81080001-81120000 | 2.83E-103 | HiC | GSE87112 | Mesendoderm | intra | rs117203215;rs77741796 | ENSG00000111046:ENSG00000111049 | 0 |
| 6 | 12:80800001-80840000 | 12:81120001-81160000 | 3.09E-67 | HiC | GSE87112 | Mesendoderm | intra | rs117203215;rs77741796 | NA | 0 |
| 6 | 12:80800001-80840000 | 12:81160001-81200000 | 5.07E-24 | HiC | GSE87112 | Mesendoderm | intra | rs117203215;rs77741796 | NA | 0 |
| 6 | 12:80800001-80840000 | 12:81200001-81240000 | 8.27E-07 | HiC | GSE87112 | Mesendoderm | intra | rs117203215;rs77741796 | NA | 0 |
| 6 | 12:80800001-80840000 | 12:81240001-81280000 | 1.78E-26 | HiC | GSE87112 | Mesendoderm | intra | rs117203215;rs77741796 | NA | 0 |
| 6 | 12:80800001-80840000 | 12:81280001-81320000 | 8.27E-30 | HiC | GSE87112 | Mesendoderm | intra | rs117203215;rs77741796 | NA | 0 |
| 6 | 12:80800001-80840000 | 12:81320001-81360000 | 1.40E-36 | HiC | GSE87112 | Mesendoderm | intra | rs117203215;rs77741796 | ENSG00000111052:ENSG00000111058 | 0 |
| 6 | 12:80800001-80840000 | 12:81360001-81400000 | 6.35E-14 | HiC | GSE87112 | Mesendoderm | intra | rs117203215;rs77741796 | NA | 0 |
| 6 | 12:80800001-80840000 | 12:81400001-81440000 | 6.68E-20 | HiC | GSE87112 | Mesendoderm | intra | rs117203215;rs77741796 | NA | 0 |
| 6 | 12:80800001-80840000 | 12:81440001-81480000 | 3.63E-17 | HiC | GSE87112 | Mesendoderm | intra | rs117203215;rs77741796 | NA | 0 |
| 6 | 12:80800001-80840000 | 12:81480001-81520000 | 5.70E-66 | HiC | GSE87112 | Mesendoderm | intra | rs117203215;rs77741796 | NA | 0 |
| 6 | 12:80800001-80840000 | 12:81520001-81560000 | 1.68E-25 | HiC | GSE87112 | Mesendoderm | intra | rs117203215;rs77741796 | NA | 0 |
| 6 | 12:80800001-80840000 | 12:81600001-81640000 | 1.18E-67 | HiC | GSE87112 | Mesendoderm | intra | rs117203215;rs77741796 | NA | 0 |
| 6 | 12:80800001-80840000 | 12:81640001-81680000 | 4.58E-96 | HiC | GSE87112 | Mesendoderm | intra | rs117203215;rs77741796 | NA | 0 |
| 6 | 12:80800001-80840000 | 12:81680001-81720000 | 6.27E-42 | HiC | GSE87112 | Mesendoderm | intra | rs117203215;rs77741796 | NA | 0 |
| 6 | 12:80800001-80840000 | 12:81760001-81800000 | 6.22E-40 | HiC | GSE87112 | Mesendoderm | intra | rs117203215;rs77741796 | NA | 0 |
| 6 | 12:80800001-80840000 | 12:81800001-81840000 | 1.08E-07 | HiC | GSE87112 | Mesendoderm | intra | rs117203215;rs77741796 | NA | 0 |
| 6 | 12:80800001-80840000 | 12:81840001-81880000 | 5.01E-14 | HiC | GSE87112 | Mesendoderm | intra | rs117203215;rs77741796 | NA | 0 |
| 6 | 12:80800001-80840000 | 12:81920001-81960000 | 3.53E-19 | HiC | GSE87112 | Mesendoderm | intra | rs117203215;rs77741796 | NA | 0 |
| 6 | 12:80800001-80840000 | 12:81960001-82000000 | 1.83E-20 | HiC | GSE87112 | Mesendoderm | intra | rs117203215;rs77741796 | NA | 0 |
| 6 | 12:80800001-80840000 | 12:82000001-82040000 | 8.88E-07 | HiC | GSE87112 | Mesendoderm | intra | rs117203215;rs77741796 | NA | 0 |
| 6 | 12:80800001-80840000 | 12:82840001-82880000 | 6.49E-07 | HiC | GSE87112 | Mesendoderm | intra | rs117203215;rs77741796 | NA | 0 |
| 6 | 12:80840001-80880000 | 12:80920001-80960000 | 3.82E-25 | HiC | GSE87112 | Mesendoderm | intra | rs200589835 | NA | 0 |
| 6 | 12:80840001-80880000 | 12:81000001-81040000 | 1.76E-17 | HiC | GSE87112 | Mesendoderm | intra | rs200589835 | NA | 0 |
| 6 | 12:80840001-80880000 | 12:81040001-81080000 | 5.15E-08 | HiC | GSE87112 | Mesendoderm | intra | rs200589835 | NA | 0 |
| 6 | 12:80840001-80880000 | 12:81080001-81120000 | 3.72E-11 | HiC | GSE87112 | Mesendoderm | intra | rs200589835 | ENSG00000111046:ENSG00000111049 | 0 |
| 6 | 12:80840001-80880000 | 12:81120001-81160000 | 3.08E-08 | HiC | GSE87112 | Mesendoderm | intra | rs200589835 | NA | 0 |
| 6 | 12:80840001-80880000 | 12:81280001-81320000 | 4.00E-08 | HiC | GSE87112 | Mesendoderm | intra | rs200589835 | NA | 0 |
| 6 | 12:80840001-80880000 | 12:81480001-81520000 | 4.96E-13 | HiC | GSE87112 | Mesendoderm | intra | rs200589835 | NA | 0 |
| 6 | 12:80840001-80880000 | 12:81600001-81640000 | 1.10E-19 | HiC | GSE87112 | Mesendoderm | intra | rs200589835 | NA | 0 |
| 6 | 12:80840001-80880000 | 12:81640001-81680000 | 1.87E-34 | HiC | GSE87112 | Mesendoderm | intra | rs200589835 | NA | 0 |
| 6 | 12:80840001-80880000 | 12:81680001-81720000 | 2.72E-10 | HiC | GSE87112 | Mesendoderm | intra | rs200589835 | NA | 0 |
| 6 | 12:80840001-80880000 | 12:81760001-81800000 | 6.71E-14 | HiC | GSE87112 | Mesendoderm | intra | rs200589835 | NA | 0 |
| 7 | 12:127760001-127800000 | 12:127800001-127840000 | 1.39E-245 | HiC | GSE87112 | Mesendoderm | intra | rs1810088;rs367839627;rs146043604;rs143439170;rs10773382;rs10847307;rs35154640;rs1552386;rs7314186;rs1979065;rs5801722;rs10744302;12:127775513:A:C;rs10734938;rs35024927;rs10773387;rs978813;rs2348321;rs10847321;rs10847323;rs6489163 | NA | 0 |
| 7 | 12:127760001-127800000 | 12:127840001-127880000 | 1.06E-78 | HiC | GSE87112 | Mesendoderm | intra | rs1810088;rs367839627;rs146043604;rs143439170;rs10773382;rs10847307;rs35154640;rs1552386;rs7314186;rs1979065;rs5801722;rs10744302;12:127775513:A:C;rs10734938;rs35024927;rs10773387;rs978813;rs2348321;rs10847321;rs10847323;rs6489163 | NA | 0 |
| 7 | 12:127760001-127800000 | 12:127880001-127920000 | 7.21E-20 | HiC | GSE87112 | Mesendoderm | intra | rs1810088;rs367839627;rs146043604;rs143439170;rs10773382;rs10847307;rs35154640;rs1552386;rs7314186;rs1979065;rs5801722;rs10744302;12:127775513:A:C;rs10734938;rs35024927;rs10773387;rs978813;rs2348321;rs10847321;rs10847323;rs6489163 | NA | 0 |
| 7 | 12:127760001-127800000 | 12:128000001-128040000 | 1.72E-17 | HiC | GSE87112 | Mesendoderm | intra | rs1810088;rs367839627;rs146043604;rs143439170;rs10773382;rs10847307;rs35154640;rs1552386;rs7314186;rs1979065;rs5801722;rs10744302;12:127775513:A:C;rs10734938;rs35024927;rs10773387;rs978813;rs2348321;rs10847321;rs10847323;rs6489163 | NA | 0 |
| 7 | 12:127760001-127800000 | 12:128040001-128080000 | 4.14E-19 | HiC | GSE87112 | Mesendoderm | intra | rs1810088;rs367839627;rs146043604;rs143439170;rs10773382;rs10847307;rs35154640;rs1552386;rs7314186;rs1979065;rs5801722;rs10744302;12:127775513:A:C;rs10734938;rs35024927;rs10773387;rs978813;rs2348321;rs10847321;rs10847323;rs6489163 | NA | 0 |
| 7 | 12:127760001-127800000 | 12:128080001-128120000 | 5.19E-08 | HiC | GSE87112 | Mesendoderm | intra | rs1810088;rs367839627;rs146043604;rs143439170;rs10773382;rs10847307;rs35154640;rs1552386;rs7314186;rs1979065;rs5801722;rs10744302;12:127775513:A:C;rs10734938;rs35024927;rs10773387;rs978813;rs2348321;rs10847321;rs10847323;rs6489163 | NA | 0 |
| 7 | 12:127760001-127800000 | 12:128120001-128160000 | 1.20E-27 | HiC | GSE87112 | Mesendoderm | intra | rs1810088;rs367839627;rs146043604;rs143439170;rs10773382;rs10847307;rs35154640;rs1552386;rs7314186;rs1979065;rs5801722;rs10744302;12:127775513:A:C;rs10734938;rs35024927;rs10773387;rs978813;rs2348321;rs10847321;rs10847323;rs6489163 | NA | 0 |
| 7 | 12:127760001-127800000 | 12:128160001-128200000 | 2.74E-07 | HiC | GSE87112 | Mesendoderm | intra | rs1810088;rs367839627;rs146043604;rs143439170;rs10773382;rs10847307;rs35154640;rs1552386;rs7314186;rs1979065;rs5801722;rs10744302;12:127775513:A:C;rs10734938;rs35024927;rs10773387;rs978813;rs2348321;rs10847321;rs10847323;rs6489163 | NA | 0 |
| 7 | 12:127760001-127800000 | 12:128200001-128240000 | 3.25E-22 | HiC | GSE87112 | Mesendoderm | intra | rs1810088;rs367839627;rs146043604;rs143439170;rs10773382;rs10847307;rs35154640;rs1552386;rs7314186;rs1979065;rs5801722;rs10744302;12:127775513:A:C;rs10734938;rs35024927;rs10773387;rs978813;rs2348321;rs10847321;rs10847323;rs6489163 | NA | 0 |
| 7 | 12:127760001-127800000 | 12:128240001-128280000 | 1.42E-13 | HiC | GSE87112 | Mesendoderm | intra | rs1810088;rs367839627;rs146043604;rs143439170;rs10773382;rs10847307;rs35154640;rs1552386;rs7314186;rs1979065;rs5801722;rs10744302;12:127775513:A:C;rs10734938;rs35024927;rs10773387;rs978813;rs2348321;rs10847321;rs10847323;rs6489163 | NA | 0 |
| 7 | 12:127760001-127800000 | 12:128280001-128320000 | 6.94E-09 | HiC | GSE87112 | Mesendoderm | intra | rs1810088;rs367839627;rs146043604;rs143439170;rs10773382;rs10847307;rs35154640;rs1552386;rs7314186;rs1979065;rs5801722;rs10744302;12:127775513:A:C;rs10734938;rs35024927;rs10773387;rs978813;rs2348321;rs10847321;rs10847323;rs6489163 | NA | 0 |
| 7 | 12:127760001-127800000 | 12:128360001-128400000 | 6.68E-20 | HiC | GSE87112 | Mesendoderm | intra | rs1810088;rs367839627;rs146043604;rs143439170;rs10773382;rs10847307;rs35154640;rs1552386;rs7314186;rs1979065;rs5801722;rs10744302;12:127775513:A:C;rs10734938;rs35024927;rs10773387;rs978813;rs2348321;rs10847321;rs10847323;rs6489163 | NA | 0 |
| 7 | 12:127760001-127800000 | 12:128400001-128440000 | 4.43E-59 | HiC | GSE87112 | Mesendoderm | intra | rs1810088;rs367839627;rs146043604;rs143439170;rs10773382;rs10847307;rs35154640;rs1552386;rs7314186;rs1979065;rs5801722;rs10744302;12:127775513:A:C;rs10734938;rs35024927;rs10773387;rs978813;rs2348321;rs10847321;rs10847323;rs6489163 | NA | 0 |
| 7 | 12:127760001-127800000 | 12:128480001-128520000 | 4.93E-26 | HiC | GSE87112 | Mesendoderm | intra | rs1810088;rs367839627;rs146043604;rs143439170;rs10773382;rs10847307;rs35154640;rs1552386;rs7314186;rs1979065;rs5801722;rs10744302;12:127775513:A:C;rs10734938;rs35024927;rs10773387;rs978813;rs2348321;rs10847321;rs10847323;rs6489163 | NA | 0 |
| 7 | 12:127760001-127800000 | 12:128520001-128560000 | 2.15E-30 | HiC | GSE87112 | Mesendoderm | intra | rs1810088;rs367839627;rs146043604;rs143439170;rs10773382;rs10847307;rs35154640;rs1552386;rs7314186;rs1979065;rs5801722;rs10744302;12:127775513:A:C;rs10734938;rs35024927;rs10773387;rs978813;rs2348321;rs10847321;rs10847323;rs6489163 | NA | 0 |
| 7 | 12:127760001-127800000 | 12:128600001-128640000 | 5.16E-08 | HiC | GSE87112 | Mesendoderm | intra | rs1810088;rs367839627;rs146043604;rs143439170;rs10773382;rs10847307;rs35154640;rs1552386;rs7314186;rs1979065;rs5801722;rs10744302;12:127775513:A:C;rs10734938;rs35024927;rs10773387;rs978813;rs2348321;rs10847321;rs10847323;rs6489163 | NA | 0 |
| 7 | 12:127760001-127800000 | 12:128640001-128680000 | 1.09E-12 | HiC | GSE87112 | Mesendoderm | intra | rs1810088;rs367839627;rs146043604;rs143439170;rs10773382;rs10847307;rs35154640;rs1552386;rs7314186;rs1979065;rs5801722;rs10744302;12:127775513:A:C;rs10734938;rs35024927;rs10773387;rs978813;rs2348321;rs10847321;rs10847323;rs6489163 | NA | 0 |
| 7 | 12:127760001-127800000 | 12:128680001-128720000 | 3.27E-07 | HiC | GSE87112 | Mesendoderm | intra | rs1810088;rs367839627;rs146043604;rs143439170;rs10773382;rs10847307;rs35154640;rs1552386;rs7314186;rs1979065;rs5801722;rs10744302;12:127775513:A:C;rs10734938;rs35024927;rs10773387;rs978813;rs2348321;rs10847321;rs10847323;rs6489163 | NA | 0 |
| 1 | 3:37560001-37600000 | 3:37480001-37520000 | 2.86E-23 | HiC | GSE87112 | Mesendoderm | intra | rs74985154;rs3733138;rs3733140;rs75619156;rs17229924;rs2162356;rs74786716;rs77820597;rs112585731;rs915631;rs79149273;rs17230261;rs199607377;rs2162355;rs76952775;rs75469174;rs78210776;rs77162103;rs75217006;rs78292414 | ENSG00000144668 | 1 |
| 2 | 3:96520001-96560000 | 3:93720001-93760000 | 1.58E-09 | HiC | GSE87112 | Mesendoderm | intra | rs62262901;rs62262902;rs62262903;rs62262930;rs151165295;rs62262931;rs62262933;rs62262934;rs74641574;rs79645223;rs2318156;rs2318155;rs2318154 | ENSG00000178750 | 0 |
| 2 | 3:96520001-96560000 | 3:93800001-93840000 | 5.01E-38 | HiC | GSE87112 | Mesendoderm | intra | rs62262901;rs62262902;rs62262903;rs62262930;rs151165295;rs62262931;rs62262933;rs62262934;rs74641574;rs79645223;rs2318156;rs2318155;rs2318154 | NA | 0 |
| 2 | 3:96560001-96600000 | 3:93800001-93840000 | 3.18E-10 | HiC | GSE87112 | Mesendoderm | intra | rs16836353;rs62262935;rs62262936;rs62262937;rs62262938;rs62262940;rs62262941;rs62262942;rs62262944;rs62262946;rs62262947;rs62262948;rs62262949;rs62262950;rs62262951 | NA | 0 |
| 2 | 3:96600001-96640000 | 3:93800001-93840000 | 7.58E-09 | HiC | GSE87112 | Mesendoderm | intra | rs62262952;rs62262953;rs116253310;rs76857958;rs111706803;rs62262955;rs62262957;rs62262958;rs62263676;rs187619159;rs62263677;rs62263678;rs574559070;rs534937763;rs62263680;rs62263682;rs62263684;rs16836982;rs16836986;rs112527011;rs62263685;rs62263686 | NA | 0 |
| 2 | 3:96680001-96720000 | 3:93800001-93840000 | 3.59E-13 | HiC | GSE87112 | Mesendoderm | intra | rs62263705;rs62263706;rs6762348;rs62263709;rs137983052;rs62263734;rs2856474;rs2612274;rs553308194 | NA | 0 |
| 2 | 3:96520001-96560000 | 3:93840001-93880000 | 8.98E-14 | HiC | GSE87112 | Mesendoderm | intra | rs62262901;rs62262902;rs62262903;rs62262930;rs151165295;rs62262931;rs62262933;rs62262934;rs74641574;rs79645223;rs2318156;rs2318155;rs2318154 | NA | 0 |
| 2 | 3:96560001-96600000 | 3:93840001-93880000 | 3.47E-08 | HiC | GSE87112 | Mesendoderm | intra | rs16836353;rs62262935;rs62262936;rs62262937;rs62262938;rs62262940;rs62262941;rs62262942;rs62262944;rs62262946;rs62262947;rs62262948;rs62262949;rs62262950;rs62262951 | NA | 0 |
| 2 | 3:96600001-96640000 | 3:93840001-93880000 | 1.15E-11 | HiC | GSE87112 | Mesendoderm | intra | rs62262952;rs62262953;rs116253310;rs76857958;rs111706803;rs62262955;rs62262957;rs62262958;rs62263676;rs187619159;rs62263677;rs62263678;rs574559070;rs534937763;rs62263680;rs62263682;rs62263684;rs16836982;rs16836986;rs112527011;rs62263685;rs62263686 | NA | 0 |
| 2 | 3:96680001-96720000 | 3:93840001-93880000 | 3.47E-08 | HiC | GSE87112 | Mesendoderm | intra | rs62263705;rs62263706;rs6762348;rs62263709;rs137983052;rs62263734;rs2856474;rs2612274;rs553308194 | NA | 0 |
| 2 | 3:96520001-96560000 | 3:93880001-93920000 | 1.38E-38 | HiC | GSE87112 | Mesendoderm | intra | rs62262901;rs62262902;rs62262903;rs62262930;rs151165295;rs62262931;rs62262933;rs62262934;rs74641574;rs79645223;rs2318156;rs2318155;rs2318154 | NA | 0 |
| 2 | 3:96560001-96600000 | 3:93880001-93920000 | 8.98E-14 | HiC | GSE87112 | Mesendoderm | intra | rs16836353;rs62262935;rs62262936;rs62262937;rs62262938;rs62262940;rs62262941;rs62262942;rs62262944;rs62262946;rs62262947;rs62262948;rs62262949;rs62262950;rs62262951 | NA | 0 |
| 2 | 3:96600001-96640000 | 3:93880001-93920000 | 3.47E-08 | HiC | GSE87112 | Mesendoderm | intra | rs62262952;rs62262953;rs116253310;rs76857958;rs111706803;rs62262955;rs62262957;rs62262958;rs62263676;rs187619159;rs62263677;rs62263678;rs574559070;rs534937763;rs62263680;rs62263682;rs62263684;rs16836982;rs16836986;rs112527011;rs62263685;rs62263686 | NA | 0 |
| 2 | 3:96680001-96720000 | 3:93880001-93920000 | 2.17E-22 | HiC | GSE87112 | Mesendoderm | intra | rs62263705;rs62263706;rs6762348;rs62263709;rs137983052;rs62263734;rs2856474;rs2612274;rs553308194 | NA | 0 |
| 2 | 3:96520001-96560000 | 3:93920001-93960000 | 4.86E-13 | HiC | GSE87112 | Mesendoderm | intra | rs62262901;rs62262902;rs62262903;rs62262930;rs151165295;rs62262931;rs62262933;rs62262934;rs74641574;rs79645223;rs2318156;rs2318155;rs2318154 | NA | 0 |
| 2 | 3:96680001-96720000 | 3:93920001-93960000 | 6.41E-07 | HiC | GSE87112 | Mesendoderm | intra | rs62263705;rs62263706;rs6762348;rs62263709;rs137983052;rs62263734;rs2856474;rs2612274;rs553308194 | NA | 0 |
| 2 | 3:96520001-96560000 | 3:93960001-94000000 | 3.29E-19 | HiC | GSE87112 | Mesendoderm | intra | rs62262901;rs62262902;rs62262903;rs62262930;rs151165295;rs62262931;rs62262933;rs62262934;rs74641574;rs79645223;rs2318156;rs2318155;rs2318154 | NA | 0 |
| 2 | 3:96560001-96600000 | 3:93960001-94000000 | 1.25E-09 | HiC | GSE87112 | Mesendoderm | intra | rs16836353;rs62262935;rs62262936;rs62262937;rs62262938;rs62262940;rs62262941;rs62262942;rs62262944;rs62262946;rs62262947;rs62262948;rs62262949;rs62262950;rs62262951 | NA | 0 |
| 2 | 3:96680001-96720000 | 3:93960001-94000000 | 3.59E-13 | HiC | GSE87112 | Mesendoderm | intra | rs62263705;rs62263706;rs6762348;rs62263709;rs137983052;rs62263734;rs2856474;rs2612274;rs553308194 | NA | 0 |
| 2 | 3:96520001-96560000 | 3:94000001-94040000 | 4.22E-54 | HiC | GSE87112 | Mesendoderm | intra | rs62262901;rs62262902;rs62262903;rs62262930;rs151165295;rs62262931;rs62262933;rs62262934;rs74641574;rs79645223;rs2318156;rs2318155;rs2318154 | NA | 0 |
| 2 | 3:96560001-96600000 | 3:94000001-94040000 | 6.55E-26 | HiC | GSE87112 | Mesendoderm | intra | rs16836353;rs62262935;rs62262936;rs62262937;rs62262938;rs62262940;rs62262941;rs62262942;rs62262944;rs62262946;rs62262947;rs62262948;rs62262949;rs62262950;rs62262951 | NA | 0 |
| 2 | 3:96600001-96640000 | 3:94000001-94040000 | 5.65E-20 | HiC | GSE87112 | Mesendoderm | intra | rs62262952;rs62262953;rs116253310;rs76857958;rs111706803;rs62262955;rs62262957;rs62262958;rs62263676;rs187619159;rs62263677;rs62263678;rs574559070;rs534937763;rs62263680;rs62263682;rs62263684;rs16836982;rs16836986;rs112527011;rs62263685;rs62263686 | NA | 0 |
| 2 | 3:96680001-96720000 | 3:94000001-94040000 | 5.67E-22 | HiC | GSE87112 | Mesendoderm | intra | rs62263705;rs62263706;rs6762348;rs62263709;rs137983052;rs62263734;rs2856474;rs2612274;rs553308194 | NA | 0 |
| 2 | 3:96720001-96760000 | 3:94000001-94040000 | 9.81E-15 | HiC | GSE87112 | Mesendoderm | intra | rs2027816;rs2856463;rs2019818;rs115680726;rs112449918;rs565327317;rs2213251;rs2213252;rs2856466 | NA | 0 |
| 2 | 3:96520001-96560000 | 3:94040001-94080000 | 1.95E-24 | HiC | GSE87112 | Mesendoderm | intra | rs62262901;rs62262902;rs62262903;rs62262930;rs151165295;rs62262931;rs62262933;rs62262934;rs74641574;rs79645223;rs2318156;rs2318155;rs2318154 | NA | 0 |
| 2 | 3:96560001-96600000 | 3:94040001-94080000 | 4.95E-07 | HiC | GSE87112 | Mesendoderm | intra | rs16836353;rs62262935;rs62262936;rs62262937;rs62262938;rs62262940;rs62262941;rs62262942;rs62262944;rs62262946;rs62262947;rs62262948;rs62262949;rs62262950;rs62262951 | NA | 0 |
| 2 | 3:96680001-96720000 | 3:94040001-94080000 | 1.81E-14 | HiC | GSE87112 | Mesendoderm | intra | rs62263705;rs62263706;rs6762348;rs62263709;rs137983052;rs62263734;rs2856474;rs2612274;rs553308194 | NA | 0 |
| 2 | 3:96520001-96560000 | 3:94120001-94160000 | 2.18E-28 | HiC | GSE87112 | Mesendoderm | intra | rs62262901;rs62262902;rs62262903;rs62262930;rs151165295;rs62262931;rs62262933;rs62262934;rs74641574;rs79645223;rs2318156;rs2318155;rs2318154 | NA | 0 |
| 2 | 3:96560001-96600000 | 3:94120001-94160000 | 9.45E-09 | HiC | GSE87112 | Mesendoderm | intra | rs16836353;rs62262935;rs62262936;rs62262937;rs62262938;rs62262940;rs62262941;rs62262942;rs62262944;rs62262946;rs62262947;rs62262948;rs62262949;rs62262950;rs62262951 | NA | 0 |
| 2 | 3:96600001-96640000 | 3:94120001-94160000 | 9.49E-07 | HiC | GSE87112 | Mesendoderm | intra | rs62262952;rs62262953;rs116253310;rs76857958;rs111706803;rs62262955;rs62262957;rs62262958;rs62263676;rs187619159;rs62263677;rs62263678;rs574559070;rs534937763;rs62263680;rs62263682;rs62263684;rs16836982;rs16836986;rs112527011;rs62263685;rs62263686 | NA | 0 |
| 2 | 3:96680001-96720000 | 3:94120001-94160000 | 1.74E-22 | HiC | GSE87112 | Mesendoderm | intra | rs62263705;rs62263706;rs6762348;rs62263709;rs137983052;rs62263734;rs2856474;rs2612274;rs553308194 | NA | 0 |
| 2 | 3:96520001-96560000 | 3:94160001-94200000 | 8.80E-37 | HiC | GSE87112 | Mesendoderm | intra | rs62262901;rs62262902;rs62262903;rs62262930;rs151165295;rs62262931;rs62262933;rs62262934;rs74641574;rs79645223;rs2318156;rs2318155;rs2318154 | NA | 0 |
| 2 | 3:96600001-96640000 | 3:94160001-94200000 | 4.64E-16 | HiC | GSE87112 | Mesendoderm | intra | rs62262952;rs62262953;rs116253310;rs76857958;rs111706803;rs62262955;rs62262957;rs62262958;rs62263676;rs187619159;rs62263677;rs62263678;rs574559070;rs534937763;rs62263680;rs62263682;rs62263684;rs16836982;rs16836986;rs112527011;rs62263685;rs62263686 | NA | 0 |
| 2 | 3:96680001-96720000 | 3:94160001-94200000 | 2.29E-13 | HiC | GSE87112 | Mesendoderm | intra | rs62263705;rs62263706;rs6762348;rs62263709;rs137983052;rs62263734;rs2856474;rs2612274;rs553308194 | NA | 0 |
| 2 | 3:96520001-96560000 | 3:94200001-94240000 | 4.81E-15 | HiC | GSE87112 | Mesendoderm | intra | rs62262901;rs62262902;rs62262903;rs62262930;rs151165295;rs62262931;rs62262933;rs62262934;rs74641574;rs79645223;rs2318156;rs2318155;rs2318154 | NA | 0 |
| 2 | 3:96600001-96640000 | 3:94200001-94240000 | 2.28E-07 | HiC | GSE87112 | Mesendoderm | intra | rs62262952;rs62262953;rs116253310;rs76857958;rs111706803;rs62262955;rs62262957;rs62262958;rs62263676;rs187619159;rs62263677;rs62263678;rs574559070;rs534937763;rs62263680;rs62263682;rs62263684;rs16836982;rs16836986;rs112527011;rs62263685;rs62263686 | NA | 0 |
| 2 | 3:96520001-96560000 | 3:94240001-94280000 | 2.13E-21 | HiC | GSE87112 | Mesendoderm | intra | rs62262901;rs62262902;rs62262903;rs62262930;rs151165295;rs62262931;rs62262933;rs62262934;rs74641574;rs79645223;rs2318156;rs2318155;rs2318154 | NA | 0 |
| 2 | 3:96600001-96640000 | 3:94240001-94280000 | 2.84E-08 | HiC | GSE87112 | Mesendoderm | intra | rs62262952;rs62262953;rs116253310;rs76857958;rs111706803;rs62262955;rs62262957;rs62262958;rs62263676;rs187619159;rs62263677;rs62263678;rs574559070;rs534937763;rs62263680;rs62263682;rs62263684;rs16836982;rs16836986;rs112527011;rs62263685;rs62263686 | NA | 0 |
| 2 | 3:96520001-96560000 | 3:94280001-94320000 | 1.20E-10 | HiC | GSE87112 | Mesendoderm | intra | rs62262901;rs62262902;rs62262903;rs62262930;rs151165295;rs62262931;rs62262933;rs62262934;rs74641574;rs79645223;rs2318156;rs2318155;rs2318154 | NA | 0 |
| 2 | 3:96520001-96560000 | 3:94320001-94360000 | 7.20E-10 | HiC | GSE87112 | Mesendoderm | intra | rs62262901;rs62262902;rs62262903;rs62262930;rs151165295;rs62262931;rs62262933;rs62262934;rs74641574;rs79645223;rs2318156;rs2318155;rs2318154 | NA | 0 |
| 2 | 3:96520001-96560000 | 3:94360001-94400000 | 1.05E-09 | HiC | GSE87112 | Mesendoderm | intra | rs62262901;rs62262902;rs62262903;rs62262930;rs151165295;rs62262931;rs62262933;rs62262934;rs74641574;rs79645223;rs2318156;rs2318155;rs2318154 | NA | 0 |
| 2 | 3:96520001-96560000 | 3:94440001-94480000 | 8.16E-18 | HiC | GSE87112 | Mesendoderm | intra | rs62262901;rs62262902;rs62262903;rs62262930;rs151165295;rs62262931;rs62262933;rs62262934;rs74641574;rs79645223;rs2318156;rs2318155;rs2318154 | NA | 0 |
| 2 | 3:96520001-96560000 | 3:94480001-94520000 | 3.64E-16 | HiC | GSE87112 | Mesendoderm | intra | rs62262901;rs62262902;rs62262903;rs62262930;rs151165295;rs62262931;rs62262933;rs62262934;rs74641574;rs79645223;rs2318156;rs2318155;rs2318154 | NA | 0 |
| 2 | 3:96680001-96720000 | 3:94480001-94520000 | 2.77E-09 | HiC | GSE87112 | Mesendoderm | intra | rs62263705;rs62263706;rs6762348;rs62263709;rs137983052;rs62263734;rs2856474;rs2612274;rs553308194 | NA | 0 |
| 2 | 3:96520001-96560000 | 3:94520001-94560000 | 6.47E-16 | HiC | GSE87112 | Mesendoderm | intra | rs62262901;rs62262902;rs62262903;rs62262930;rs151165295;rs62262931;rs62262933;rs62262934;rs74641574;rs79645223;rs2318156;rs2318155;rs2318154 | NA | 0 |
| 2 | 3:96680001-96720000 | 3:94520001-94560000 | 4.89E-19 | HiC | GSE87112 | Mesendoderm | intra | rs62263705;rs62263706;rs6762348;rs62263709;rs137983052;rs62263734;rs2856474;rs2612274;rs553308194 | NA | 0 |
| 2 | 3:96520001-96560000 | 3:94560001-94600000 | 8.69E-12 | HiC | GSE87112 | Mesendoderm | intra | rs62262901;rs62262902;rs62262903;rs62262930;rs151165295;rs62262931;rs62262933;rs62262934;rs74641574;rs79645223;rs2318156;rs2318155;rs2318154 | NA | 0 |
| 2 | 3:96520001-96560000 | 3:94600001-94640000 | 2.04E-17 | HiC | GSE87112 | Mesendoderm | intra | rs62262901;rs62262902;rs62262903;rs62262930;rs151165295;rs62262931;rs62262933;rs62262934;rs74641574;rs79645223;rs2318156;rs2318155;rs2318154 | NA | 0 |
| 2 | 3:96520001-96560000 | 3:94640001-94680000 | 6.65E-20 | HiC | GSE87112 | Mesendoderm | intra | rs62262901;rs62262902;rs62262903;rs62262930;rs151165295;rs62262931;rs62262933;rs62262934;rs74641574;rs79645223;rs2318156;rs2318155;rs2318154 | NA | 0 |
| 2 | 3:96600001-96640000 | 3:94640001-94680000 | 2.80E-07 | HiC | GSE87112 | Mesendoderm | intra | rs62262952;rs62262953;rs116253310;rs76857958;rs111706803;rs62262955;rs62262957;rs62262958;rs62263676;rs187619159;rs62263677;rs62263678;rs574559070;rs534937763;rs62263680;rs62263682;rs62263684;rs16836982;rs16836986;rs112527011;rs62263685;rs62263686 | NA | 0 |
| 2 | 3:96680001-96720000 | 3:94640001-94680000 | 8.35E-15 | HiC | GSE87112 | Mesendoderm | intra | rs62263705;rs62263706;rs6762348;rs62263709;rs137983052;rs62263734;rs2856474;rs2612274;rs553308194 | NA | 0 |
| 2 | 3:96520001-96560000 | 3:94680001-94720000 | 2.08E-17 | HiC | GSE87112 | Mesendoderm | intra | rs62262901;rs62262902;rs62262903;rs62262930;rs151165295;rs62262931;rs62262933;rs62262934;rs74641574;rs79645223;rs2318156;rs2318155;rs2318154 | NA | 0 |
| 2 | 3:96560001-96600000 | 3:94680001-94720000 | 6.02E-08 | HiC | GSE87112 | Mesendoderm | intra | rs16836353;rs62262935;rs62262936;rs62262937;rs62262938;rs62262940;rs62262941;rs62262942;rs62262944;rs62262946;rs62262947;rs62262948;rs62262949;rs62262950;rs62262951 | NA | 0 |
| 2 | 3:96520001-96560000 | 3:94760001-94800000 | 3.00E-07 | HiC | GSE87112 | Mesendoderm | intra | rs62262901;rs62262902;rs62262903;rs62262930;rs151165295;rs62262931;rs62262933;rs62262934;rs74641574;rs79645223;rs2318156;rs2318155;rs2318154 | NA | 0 |
| 2 | 3:96520001-96560000 | 3:94800001-94840000 | 5.80E-08 | HiC | GSE87112 | Mesendoderm | intra | rs62262901;rs62262902;rs62262903;rs62262930;rs151165295;rs62262931;rs62262933;rs62262934;rs74641574;rs79645223;rs2318156;rs2318155;rs2318154 | NA | 0 |
| 2 | 3:96520001-96560000 | 3:94840001-94880000 | 3.17E-18 | HiC | GSE87112 | Mesendoderm | intra | rs62262901;rs62262902;rs62262903;rs62262930;rs151165295;rs62262931;rs62262933;rs62262934;rs74641574;rs79645223;rs2318156;rs2318155;rs2318154 | NA | 0 |
| 2 | 3:96680001-96720000 | 3:94840001-94880000 | 1.89E-10 | HiC | GSE87112 | Mesendoderm | intra | rs62263705;rs62263706;rs6762348;rs62263709;rs137983052;rs62263734;rs2856474;rs2612274;rs553308194 | NA | 0 |
| 2 | 3:96520001-96560000 | 3:94880001-94920000 | 8.49E-08 | HiC | GSE87112 | Mesendoderm | intra | rs62262901;rs62262902;rs62262903;rs62262930;rs151165295;rs62262931;rs62262933;rs62262934;rs74641574;rs79645223;rs2318156;rs2318155;rs2318154 | NA | 0 |
| 2 | 3:96520001-96560000 | 3:94920001-94960000 | 2.12E-08 | HiC | GSE87112 | Mesendoderm | intra | rs62262901;rs62262902;rs62262903;rs62262930;rs151165295;rs62262931;rs62262933;rs62262934;rs74641574;rs79645223;rs2318156;rs2318155;rs2318154 | NA | 0 |
| 2 | 3:96680001-96720000 | 3:94920001-94960000 | 5.51E-11 | HiC | GSE87112 | Mesendoderm | intra | rs62263705;rs62263706;rs6762348;rs62263709;rs137983052;rs62263734;rs2856474;rs2612274;rs553308194 | NA | 0 |
| 2 | 3:96520001-96560000 | 3:94960001-95000000 | 5.80E-09 | HiC | GSE87112 | Mesendoderm | intra | rs62262901;rs62262902;rs62262903;rs62262930;rs151165295;rs62262931;rs62262933;rs62262934;rs74641574;rs79645223;rs2318156;rs2318155;rs2318154 | NA | 0 |
| 2 | 3:96520001-96560000 | 3:95000001-95040000 | 4.99E-15 | HiC | GSE87112 | Mesendoderm | intra | rs62262901;rs62262902;rs62262903;rs62262930;rs151165295;rs62262931;rs62262933;rs62262934;rs74641574;rs79645223;rs2318156;rs2318155;rs2318154 | NA | 0 |
| 2 | 3:96680001-96720000 | 3:95000001-95040000 | 1.20E-07 | HiC | GSE87112 | Mesendoderm | intra | rs62263705;rs62263706;rs6762348;rs62263709;rs137983052;rs62263734;rs2856474;rs2612274;rs553308194 | NA | 0 |
| 2 | 3:96520001-96560000 | 3:95040001-95080000 | 5.83E-09 | HiC | GSE87112 | Mesendoderm | intra | rs62262901;rs62262902;rs62262903;rs62262930;rs151165295;rs62262931;rs62262933;rs62262934;rs74641574;rs79645223;rs2318156;rs2318155;rs2318154 | NA | 0 |
| 2 | 3:96520001-96560000 | 3:95120001-95160000 | 3.01E-11 | HiC | GSE87112 | Mesendoderm | intra | rs62262901;rs62262902;rs62262903;rs62262930;rs151165295;rs62262931;rs62262933;rs62262934;rs74641574;rs79645223;rs2318156;rs2318155;rs2318154 | NA | 0 |
| 2 | 3:96520001-96560000 | 3:95200001-95240000 | 5.71E-15 | HiC | GSE87112 | Mesendoderm | intra | rs62262901;rs62262902;rs62262903;rs62262930;rs151165295;rs62262931;rs62262933;rs62262934;rs74641574;rs79645223;rs2318156;rs2318155;rs2318154 | NA | 0 |
| 2 | 3:96680001-96720000 | 3:95240001-95280000 | 4.53E-07 | HiC | GSE87112 | Mesendoderm | intra | rs62263705;rs62263706;rs6762348;rs62263709;rs137983052;rs62263734;rs2856474;rs2612274;rs553308194 | NA | 0 |
| 2 | 3:96520001-96560000 | 3:95280001-95320000 | 3.47E-10 | HiC | GSE87112 | Mesendoderm | intra | rs62262901;rs62262902;rs62262903;rs62262930;rs151165295;rs62262931;rs62262933;rs62262934;rs74641574;rs79645223;rs2318156;rs2318155;rs2318154 | NA | 0 |
| 2 | 3:96520001-96560000 | 3:95400001-95440000 | 3.30E-19 | HiC | GSE87112 | Mesendoderm | intra | rs62262901;rs62262902;rs62262903;rs62262930;rs151165295;rs62262931;rs62262933;rs62262934;rs74641574;rs79645223;rs2318156;rs2318155;rs2318154 | NA | 0 |
| 2 | 3:96520001-96560000 | 3:95640001-95680000 | 3.38E-08 | HiC | GSE87112 | Mesendoderm | intra | rs62262901;rs62262902;rs62262903;rs62262930;rs151165295;rs62262931;rs62262933;rs62262934;rs74641574;rs79645223;rs2318156;rs2318155;rs2318154 | NA | 0 |
| 2 | 3:96520001-96560000 | 3:95680001-95720000 | 1.42E-13 | HiC | GSE87112 | Mesendoderm | intra | rs62262901;rs62262902;rs62262903;rs62262930;rs151165295;rs62262931;rs62262933;rs62262934;rs74641574;rs79645223;rs2318156;rs2318155;rs2318154 | NA | 0 |
| 2 | 3:96520001-96560000 | 3:95720001-95760000 | 1.66E-11 | HiC | GSE87112 | Mesendoderm | intra | rs62262901;rs62262902;rs62262903;rs62262930;rs151165295;rs62262931;rs62262933;rs62262934;rs74641574;rs79645223;rs2318156;rs2318155;rs2318154 | NA | 0 |
| 2 | 3:96520001-96560000 | 3:95920001-95960000 | 3.14E-09 | HiC | GSE87112 | Mesendoderm | intra | rs62262901;rs62262902;rs62262903;rs62262930;rs151165295;rs62262931;rs62262933;rs62262934;rs74641574;rs79645223;rs2318156;rs2318155;rs2318154 | NA | 0 |
| 2 | 3:96520001-96560000 | 3:95960001-96000000 | 1.08E-07 | HiC | GSE87112 | Mesendoderm | intra | rs62262901;rs62262902;rs62262903;rs62262930;rs151165295;rs62262931;rs62262933;rs62262934;rs74641574;rs79645223;rs2318156;rs2318155;rs2318154 | NA | 0 |
| 2 | 3:96520001-96560000 | 3:96040001-96080000 | 1.30E-14 | HiC | GSE87112 | Mesendoderm | intra | rs62262901;rs62262902;rs62262903;rs62262930;rs151165295;rs62262931;rs62262933;rs62262934;rs74641574;rs79645223;rs2318156;rs2318155;rs2318154 | NA | 0 |
| 2 | 3:96520001-96560000 | 3:96080001-96120000 | 2.35E-11 | HiC | GSE87112 | Mesendoderm | intra | rs62262901;rs62262902;rs62262903;rs62262930;rs151165295;rs62262931;rs62262933;rs62262934;rs74641574;rs79645223;rs2318156;rs2318155;rs2318154 | NA | 0 |
| 2 | 3:96520001-96560000 | 3:96120001-96160000 | 6.27E-08 | HiC | GSE87112 | Mesendoderm | intra | rs62262901;rs62262902;rs62262903;rs62262930;rs151165295;rs62262931;rs62262933;rs62262934;rs74641574;rs79645223;rs2318156;rs2318155;rs2318154 | NA | 0 |
| 2 | 3:96520001-96560000 | 3:96200001-96240000 | 9.35E-07 | HiC | GSE87112 | Mesendoderm | intra | rs62262901;rs62262902;rs62262903;rs62262930;rs151165295;rs62262931;rs62262933;rs62262934;rs74641574;rs79645223;rs2318156;rs2318155;rs2318154 | NA | 0 |
| 2 | 3:96680001-96720000 | 3:96600001-96640000 | 4.62E-38 | HiC | GSE87112 | Mesendoderm | intra | rs62263705;rs62263706;rs6762348;rs62263709;rs137983052;rs62263734;rs2856474;rs2612274;rs553308194 | NA | 0 |
| 3 | 4:171480001-171520000 | 4:171000001-171040000 | 3.41E-09 | HiC | GSE87112 | Mesendoderm | intra | rs148420952;rs76413646 | ENSG00000109576 | 0 |
| 3 | 4:171480001-171520000 | 4:171040001-171080000 | 1.40E-21 | HiC | GSE87112 | Mesendoderm | intra | rs148420952;rs76413646 | NA | 0 |
| 3 | 4:171480001-171520000 | 4:171080001-171120000 | 3.13E-08 | HiC | GSE87112 | Mesendoderm | intra | rs148420952;rs76413646 | NA | 0 |
| 3 | 4:171440001-171480000 | 4:171160001-171200000 | 5.18E-17 | HiC | GSE87112 | Mesendoderm | intra | rs147274196 | NA | 0 |
| 3 | 4:171480001-171520000 | 4:171160001-171200000 | 3.55E-32 | HiC | GSE87112 | Mesendoderm | intra | rs148420952;rs76413646 | NA | 0 |
| 3 | 4:171440001-171480000 | 4:171200001-171240000 | 1.29E-15 | HiC | GSE87112 | Mesendoderm | intra | rs147274196 | NA | 0 |
| 3 | 4:171480001-171520000 | 4:171200001-171240000 | 4.22E-25 | HiC | GSE87112 | Mesendoderm | intra | rs148420952;rs76413646 | NA | 0 |
| 3 | 4:171440001-171480000 | 4:171280001-171320000 | 8.04E-13 | HiC | GSE87112 | Mesendoderm | intra | rs147274196 | NA | 0 |
| 3 | 4:171480001-171520000 | 4:171280001-171320000 | 5.62E-20 | HiC | GSE87112 | Mesendoderm | intra | rs148420952;rs76413646 | NA | 0 |
| 3 | 4:171440001-171480000 | 4:171320001-171360000 | 7.05E-08 | HiC | GSE87112 | Mesendoderm | intra | rs147274196 | NA | 0 |
| 3 | 4:171480001-171520000 | 4:171320001-171360000 | 6.46E-08 | HiC | GSE87112 | Mesendoderm | intra | rs148420952;rs76413646 | NA | 0 |
| 3 | 4:171440001-171480000 | 4:171400001-171440000 | 2.33E-41 | HiC | GSE87112 | Mesendoderm | intra | rs147274196 | NA | 0 |
| 3 | 4:171480001-171520000 | 4:171400001-171440000 | 4.66E-28 | HiC | GSE87112 | Mesendoderm | intra | rs148420952;rs76413646 | NA | 0 |
| 3 | 4:171480001-171520000 | 4:171440001-171480000 | 4.52E-23 | HiC | GSE87112 | Mesendoderm | intra | rs148420952;rs76413646 | NA | 0 |
| 4 | 5:108560001-108600000 | 5:107720001-107760000 | 1.65E-14 | HiC | GSE87112 | Mesendoderm | intra | rs35812497;rs75803484;rs75073283;rs76430100;rs75782477;rs77992445;rs61701247;rs17161655;rs115979287;rs17161659;rs151072743;rs17161665;rs6863688;5:108591279:C:T;5:108591279:A:T;rs1363213;rs1862201;rs1862202;rs1862203;rs5870359 | NA | 0 |
| 4 | 5:108600001-108640000 | 5:107720001-107760000 | 7.82E-16 | HiC | GSE87112 | Mesendoderm | intra | rs11744353;rs79776200;rs76069623;rs76043709;rs78729571;rs36123121;rs11748450;rs80234158;rs78146542;rs185072862;rs137867205;rs189006725;rs4388251;rs4438924;rs4541698;rs4388252;rs4392675;rs200252534;rs145520315;rs147699420;rs77803982;rs79469094;rs75077625;rs74379173;rs78664536;rs549536839;rs570631944;rs78784808;rs78340649;rs201569786;rs75649455;rs78814720;rs77561644;rs76565008;rs74436342;rs1592807;rs1592810;rs1592811;rs7700458;rs6594365;rs6863893;rs140163909;rs146837743;rs7705919;rs201046346;rs202152005;rs199869120;rs111726541;rs373673987;rs113026830;rs148211606;rs201619508;rs201169921;rs146644914;rs116649742;rs79893293;rs57629433;rs60335509;rs58453996;rs59466242;rs60878839;rs11743452;rs1833567;rs1833568;rs1833569;rs7721272;rs7721427;rs7721730;rs7721870;rs7722100;rs7704359;rs1895200;rs1895201;rs77974856;rs60905342;rs11746207;rs11739672;rs11749718;rs78518991;rs76243602;rs76383235;rs11741980;rs35243960;rs116462975;rs139801813;rs113103584;rs74727230;rs75919325;rs79624714;rs2080858;rs138092354;rs148285468;rs114455395;rs74850689;rs10463599 | NA | 0 |
| 4 | 5:108560001-108600000 | 5:107760001-107800000 | 3.70E-40 | HiC | GSE87112 | Mesendoderm | intra | rs35812497;rs75803484;rs75073283;rs76430100;rs75782477;rs77992445;rs61701247;rs17161655;rs115979287;rs17161659;rs151072743;rs17161665;rs6863688;5:108591279:C:T;5:108591279:A:T;rs1363213;rs1862201;rs1862202;rs1862203;rs5870359 | NA | 0 |
| 4 | 5:108600001-108640000 | 5:107760001-107800000 | 2.70E-35 | HiC | GSE87112 | Mesendoderm | intra | rs11744353;rs79776200;rs76069623;rs76043709;rs78729571;rs36123121;rs11748450;rs80234158;rs78146542;rs185072862;rs137867205;rs189006725;rs4388251;rs4438924;rs4541698;rs4388252;rs4392675;rs200252534;rs145520315;rs147699420;rs77803982;rs79469094;rs75077625;rs74379173;rs78664536;rs549536839;rs570631944;rs78784808;rs78340649;rs201569786;rs75649455;rs78814720;rs77561644;rs76565008;rs74436342;rs1592807;rs1592810;rs1592811;rs7700458;rs6594365;rs6863893;rs140163909;rs146837743;rs7705919;rs201046346;rs202152005;rs199869120;rs111726541;rs373673987;rs113026830;rs148211606;rs201619508;rs201169921;rs146644914;rs116649742;rs79893293;rs57629433;rs60335509;rs58453996;rs59466242;rs60878839;rs11743452;rs1833567;rs1833568;rs1833569;rs7721272;rs7721427;rs7721730;rs7721870;rs7722100;rs7704359;rs1895200;rs1895201;rs77974856;rs60905342;rs11746207;rs11739672;rs11749718;rs78518991;rs76243602;rs76383235;rs11741980;rs35243960;rs116462975;rs139801813;rs113103584;rs74727230;rs75919325;rs79624714;rs2080858;rs138092354;rs148285468;rs114455395;rs74850689;rs10463599 | NA | 0 |
| 4 | 5:108560001-108600000 | 5:107800001-107840000 | 1.86E-22 | HiC | GSE87112 | Mesendoderm | intra | rs35812497;rs75803484;rs75073283;rs76430100;rs75782477;rs77992445;rs61701247;rs17161655;rs115979287;rs17161659;rs151072743;rs17161665;rs6863688;5:108591279:C:T;5:108591279:A:T;rs1363213;rs1862201;rs1862202;rs1862203;rs5870359 | NA | 0 |
[truncated: 166,125 more chars]
